# Inhibition of Soluble Epoxide Hydrolase Is Protective against the Multiomic Effects of a High Glycemic Diet on Brain Microvascular Inflammation and Cognitive Dysfunction

**DOI:** 10.3390/nu13113913

**Published:** 2021-11-01

**Authors:** Saivageethi Nuthikattu, Dragan Milenkovic, Jennifer E. Norman, John Rutledge, Amparo Villablanca

**Affiliations:** 1 Division of Cardiovascular Medicine, University of California, Davis, CA 95616, USA; snuthikattu@ucdavis.edu (S.N.); dragan.milenkovic@inra.fr (D.M.); jenorman@ucdavis.edu (J.E.N.); jcrutledge@ucdavis.edu (J.R.); 2 Department of Nutrition, University of California, Davis, CA 95616, USA; 3 Unité de Nutrition Humaine, INRA, Université Clermont Auvergne, CRNH Auvergne, F-63000 Clermont-Ferrand, France

**Keywords:** multi-omics, microvascular, brain, dementia, high glycemic diet, soluble epoxide hydrolase inhibitor, maless

## Abstract

Diet is a modifiable risk factor for cardiovascular disease (CVD) and dementia, yet relatively little is known about the effect of a high glycemic diet (HGD) on the brain’s microvasculature. The objective of our study was to determine the molecular effects of an HGD on hippocampal microvessels and cognitive function and determine if a soluble epoxide hydrolase (sEH) inhibitor (sEHI), known to be vasculoprotective and anti-inflammatory, modulates these effects. Wild type male mice were fed a low glycemic diet (LGD, 12% sucrose/weight) or an HGD (34% sucrose/weight) with/without the sEHI, trans-4-[4-(3-adamantan-1-yl-ureido)-cyclohexyloxy]-benzoic acid (t-AUCB), for 12 weeks. Brain hippocampal microvascular gene expression was assessed by microarray and data analyzed using a multi-omic approach for differential expression of protein and non-protein-coding genes, gene networks, functional pathways, and transcription factors. Global hippocampal microvascular gene expression was fundamentally different for mice fed the HGD vs. the LGD. The HGD response was characterized by differential expression of 608 genes involved in cell signaling, neurodegeneration, metabolism, and cell adhesion/inflammation/oxidation effects reversible by t-AUCB and hence sEH inhibitor correlated with protection against Alzheimer’s dementia. Ours is the first study to demonstrate that high dietary glycemia contributes to brain hippocampal microvascular inflammation through sEH.

## 1. Introduction

Dementias are the seventh leading cause of death globally and contribute significantly to health care costs [1]. Several studies suggest that a high-fat diet or Western diet (high fat and high glycemic content) can lead to reduced cognitive function [2,3,4,5,6]. It has also become increasingly recognized that vasculature plays an important role in the development of dementias [7]. Our group has previously demonstrated the multi-omic and lipotoxic effect of a Western diet on the brain microvasculature and its negative consequences on cognitive function in male and female mice [8,9,10,11]. While there have been numerous mechanistic studies focusing on cognitive function with high-fat diets, few studies have explored the impact of high glycemia in the absence of high levels of dietary fat. 

There is compelling epidemiological data to suggest that the effects of a high glycemic diet (HGD) on cognitive function and the brain are an important area of study. High blood glucose and high dietary glycemic load were both found to be related to poorer performance in perceptual speed and spatial ability [12]. An HGD has also been associated with a greater cerebral amyloid burden [13]. Further, consumption of a high glycemic index afternoon snack was associated with cognitive decline in apolipoprotein E4 allele carriers [14]. However, there is relatively sparse animal data on the effects of an HGD on the brain and cognitive function. In rats, an HGD had detrimental effects on memory [15,16], disrupted hypothalamic redox homeostasis [17], and increased hippocampal endoplasmic reticulum stress [18]. In a mouse model of Alzheimer’s disease, an HGD was found to increase neuroinflammation and cortical levels of Amyloid-β [19]. These studies suggest that an HGD is associated with cognitive impairment in animal models.

The impact of high glycemia on cognition may be reversible. One study in rats found that a high sucrose diet increased brain unesterified arachidonic acid and the activity of enzymes facilitating the release of arachidonic acid from phospholipids [20]. Arachidonic acid is metabolized by cytochrome P450 enzymes to epoxyeicosatrienoic acid isomers (EETs), which are short-lasting and locally active neuroprotective, vasodilatory, and anti-inflammatory [21] signaling molecules. Soluble epoxide hydrolase (sEH) is an enzyme that converts EETs into dihydroxyeicosatrienoic acids (DHETs), which have less biological activity [21,22]. Inhibiting sEH activity thus increases the amount of beneficial EETs [22]. Studies have implicated soluble epoxide hydrolase (sEH) in many disorders of the central nervous system, including Parkinson’s disease, white matter hyperintensities, vascular cognitive decline/impairment, and Alzheimer’s disease [22,23,24,25,26]. Inhibitors of sEH (sEHI) have been shown to be protective in animal models of stroke [27,28]. Inhibition of sEH is of great clinical interest as it has also been shown to reduce neuroinflammation and cognitive impairment in animal models of cerebral hypoperfusion and type 1 and type 2 diabetes mellitus [29,30,31,32,33]. Further, the use of sEHI and genetic knockout of the sEH gene reduces cognitive impairment in animal models of age-related cognitive decline and Alzheimer’s disease [26,34,35].

The hippocampus is central to the formation of memory [36], and dysfunction of the microvasculature can contribute to the development of dementia [37]. To our knowledge, no studies have been published to date examining the effects of an HGD on brain hippocampal microvascular gene expression. The objectives of this study were to use a male murine model to comprehensively characterize the effect of an HGD on neurovascular function through hippocampal microvascular multi-omics and to assess the impact of an HGD on cognitive function. We hypothesized that an HGD would result in injurious differential gene expression changes characterized by brain hippocampal microvascular oxidation, inflammation, and blood–brain barrier disruption. Further, we aimed to determine whether the deleterious genomic effects of an HGD could be mitigated by inhibiting sEH. 

## 2. Materials and Methods

### 2.1. Experimental Animals and Soluble Epoxide Hydrolase Inhibitor (sEHI) Treatment

19-week-old C57BL/6J wild type (WT) male mice (Jackson Laboratories, stock 000664) were fed a standard chow diet (catalog no 0915 from Envigo Teklad Diets, Madison, WI, USA) and allowed to acclimate for one week prior to beginning the study procedures. Mice were then fed for 12 weeks either a Low Glycemic Index diet (LGD, catalog no TD.08485 from Envigo Teklad Diets, Madison, WI, USA) composed of 13% fat, 19.1% protein, 67.9% carbohydrate, as percent kcal, containing 12% sucrose by weight, or a High Glycemic Index diet (HGD, catalog no. TD.05230, Envigo Teklad Diets, Madison, WI, USA) composed of 12.6% fat, 18.7% protein, 68.7% carbohydrate, as percent kcal, containing 34% sucrose by weight. Mice receiving each diet were given 10mg/L of soluble epoxide hydrolase inhibitor (sEHI), trans-4-[4-(3-adamantan-1-yl-ureido)-cyclohexyloxy]-benzoic acid (t-AUCB) (Cayman Chemical, Ann Arbor, MI, USA) containing 1% *v/v* polyethylene glycol 400 (PEG400) (Millipore, Burlington, MA, USA) in the drinking water for 12 weeks at which point mice were 32 weeks of age and sacrificed. Mice consumed approximately 7 to 7.5 mL of water each day, consistent with previously published work [38], and 2.5 to 3 mg of t-AUCB (sEHI) per kg per day. There were a total of four experimental treatment groups (*n* = 7 mice/gp): LGD alone, LGD with sEHI (LGD+sEHI), HGD alone, and HGD with sEHI (HGD+sEHI). Mice were randomly assigned to the dietary groups. 

Animals were housed one mouse per cage in a temperature- and humidity-controlled environment with a 12 h light/dark cycle in the University of California, Davis Mouse Biology Program. Body weight was measured at baseline and at the completion of the dietary intervention period, and activity, water, and food intake were monitored daily by vivarium staff. The research was conducted in conformity with the Public Health Service Policy on Humane Care and Use of Laboratory Animals and all protocols approved by the Institutional Animal Care and Use Committee of the University of California, Davis.

### 2.2. Serum Lipid, Glucose, and Insulin Assays

Mice were fasted overnight for 8 hours, and blood obtained by submandibular nick blood draw for the pre diet samples, and by ventricular puncture at the time of sacrifice following completion of the dietary feeding period for the post diet samples. Blood samples were stored at −80 °C. Lipid, glucose and insulin levels were measured in fasted serum samples. Total cholesterol (TC), high-density lipoprotein cholesterol (HDL), and low-density lipoprotein cholesterol (LDL) were measured using enzymatic assays from Fisher Diagnostics (Middleton, VA, USA), and precipitation separation from AbCam (Cambridge, MA, USA) adapted to a microplate format. Glucose was measured using enzymatic assays from Fisher Diagnostics (Middleton, VA, USA), and insulin was determined by electrochemiluminescence from Meso Scale Discovery (Rockville, MD, USA) according to the manufacturer’s instructions. All serum assays were performed by the UC Davis Mouse Metabolic Phenotyping Center (MMPC) on non-pooled serum samples. 

### 2.3. Isolation and Cryosection of Murine Brain Hippocampus

Following completion of the 12 week dietary feeding period, mice were anesthetized by intraperitoneal xylazine/ketamine and euthanized by exsanguination during the light phase of their light/dark cycle. Intact brains were rapidly removed under RNAse free conditions, cut into regions including the temporal lobe segment containing the hippocampus, and embedded using HistoPrep Frozen Tissue Embedding Media (Fisher Scientific, Pittsburgh, PA, USA). To identify the hippocampus and hippocampal neurons, brain sections in the medial aspect of the temporal lobe were stained with hematoxylin and visualized with microscopy as previously described [8]. The hippocampus was then coronally cryosectioned (8 µm, Leica Frigocut 2800n Cryostat, Leica Biosystems, Buffalo Grove, IL, USA) and placed on charged RNA-free PEN Membrane Glass slides, treated with RNAlater®-ICE (Life Technologies, Grand Island, NY, USA) to prevent RNA degradation, and stored at −80 °C until use. When ready for use, cryosections from the hippocampal segments were submerged in nuclease-free water and dehydrated in desiccant.

### 2.4. Laser Capture Microdissection (LCM) of Hippocampal Microvessels

For analysis of gene transcriptome of hippocampal brain microvessels, endothelial microvessels (<20um) were first identified in the hippocampal brain cryosections by alkaline phosphatase staining utilizing 5-bromo-4-chloro-3-indolyl phosphate/nitro blue tetrazolium chloride (BCIP/NBT) substrate as previously described [39]. Laser capture microdissection (LCM) was then used to isolate the microvascular endothelium in hippocampal cryosections by capture of the entire vessel wall under direct microscopic visualization using a Leica LMD6000 Laser Microdissection Microscope (Leica Microsystems, Wetzlar, Germany). Microvessels were not categorized by hippocampal region or subregion, although they primarily corresponded to endothelial enriched sections in hippocampus dorsal segments that would have included CA1 and CA3 regions. 

### 2.5. RNA Extraction from Laser Captured Brain Microvessels

Total RNA was extracted from the laser-captured hippocampal brain microvessels (300 per mice, 3 mice per experimental group) using an Arcturus PicoPure™ RNA Isolation Kit (Thermo Fisher Scientific, Santa Clara, CA, USA) according to the manufacturer’s instructions. The quality of the RNA from the LCM-derived vessels was assessed by Nanodrop. RNA quantification was performed according to Affymetrix RNA quantification kit with SYBR Green I and ROX™ Passive Reference Dye protocol (Affymetrix, Santa Clara, CA, USA).

### 2.6. Microarray Hybridization and Transcriptome Analysis

For transcriptomics analysis, we used Clariom D Mouse Array (one array per mouse), containing more than 7 million probes for protein-coding and protein non-coding genes such as micro RNAs (miRNAs), small nucleolar RNAs (snoRNAs), and long non-coding RNAs (LncRNAs) (Thermo Fisher, Santa Clara, CA, USA). RNA (122.3 pg) was used to prepare cRNA and sscDNA using GeneChip® WT Pico Kit (Thermo Fisher, Santa Clara, CA, USA). SscDNA (5.5 μg) was fragmented by uracil-DNA glycosylase (UDG) and apurinic/apyrimidinic endonuclease 1 (APE 1) and labeled by terminal deoxynucleotidyl transferase (TdT) using the DNA Labeling Reagent that is covalently linked to biotin. Fragmented and labeled sscDNA samples were then submitted to the UC Davis Genome Center shared resource core for hybridization, staining, and scanning using Thermo Fisher Scientific WT array hybridization protocol following the manufacturer’s protocol. Hybridization of fragmented and labeled sscDNA samples was performed using GeneChip™Hybridization Oven 645, and samples were then washed and stained using GeneChip™ Fluidics Station 450. The arrays were scanned using GeneChip™ Scanner 3000 7G (Thermo Fisher Scientific, Santa Clara, CA, USA). Quality control of the microarrays and data analysis was performed using Thermo Fisher Scientific Transcriptome Analysis Console software version 4.0.2. We have deposited the microarray data in GEO, and the accession number is GSE185057.

### 2.7. Bioinformatic Analysis

**Figure 1:**
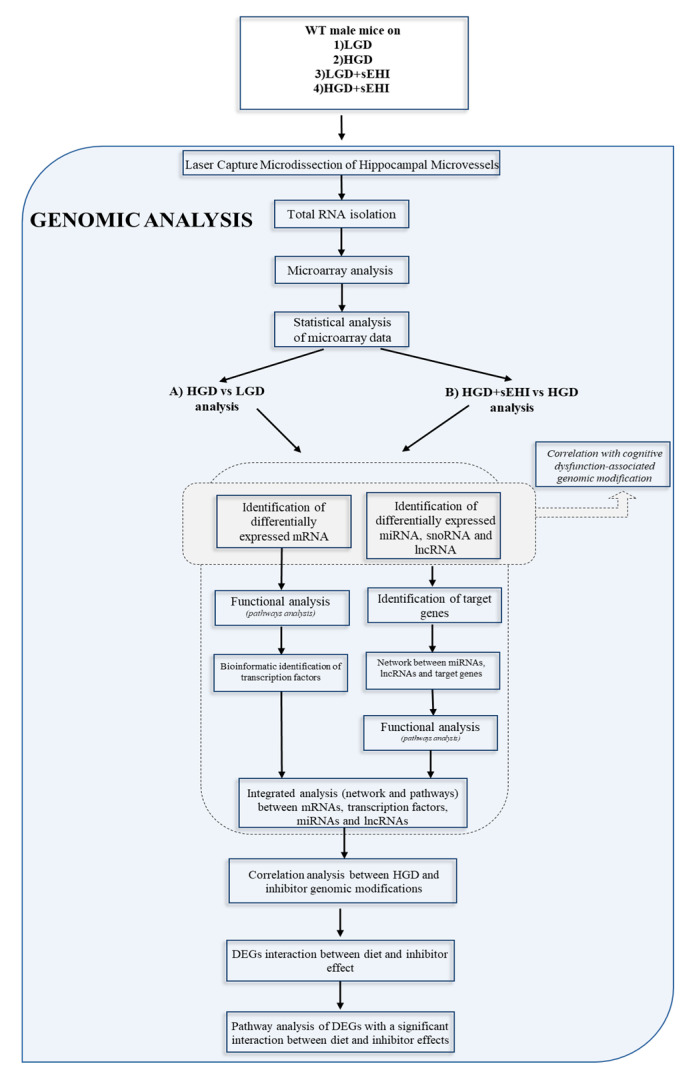
Research methodology flow chart. The flow chart shows the steps in the genomic analysis for: (A) the high glycemic diet compared to the low glycemic diet (HGD vs LGD), and (B) the high glycemic diet with the soluble epoxide hydrolase inhibitor (sEHI) compared to without the sEHI (HGD + sEHI vs HGD).

Bioinformatics analysis of differentially expressed genes was performed by two of the study investigators (SN and DM) using multiple software tools. We compared the following study groups: (A) HGD to LGD (to determine the effect of the HGD diet) and (B) HGD+sEHI to HGD (to determine the effect of the inhibitor on the HGD diet) as shown in the flow chart in Figure 1.

The Principal Component Analysis (PCA) plot of identified differentially expressed genes (DEG) was obtained through ClustVis [40]. miRNA targets of DEG were identified using Mienturnet [41]. LncRNAs of DEG were identified using LncRRIsearch [42] and Rtools CBRC [43]. Canonical pathway analysis was conducted using GeneTrial2 online database [44,45]. Networks were constructed and visualized using Cytoscape software (version 3.7.1) [46,47,48,49]. Data preparation was performed with the use of several R packages including splitstackshape [50], data.table [51], dplyr [52,53] and string [54,55]. Pathway networks were built for pathways enriched from a global pathway analysis, considering all omic layers components together. 

Transcription factor analyses were performed using Enrichr [56,57,58]. Hierarchical clustering and heat map representations of differentially expressed genes (DEG) were performed using PermutMatrix software [59,60]. 

We performed Pearson’s correlation analysis between the genes differentially expressed by the HGD vs. LGD and the HGD+sEHI vs. HGD. We used the ggpubr package in R [61] to obtain the correlation coefficient and the significance level, as well as the scatter plot with regression line and confidence interval. We then performed prediction of neurodegenerative disease trait by performing correlation analysis between the obtained changes in the expression of genes following the HGD vs. LGD or the HGD+sEHI vs. HGD and the gene expression profiles observed in patients with Alzheimer’s disease or cognitive disorders. The human genome data were extracted from the gene expression profile database, which was deposited in the GEO (gene expression omnibus) database. The identification of differentially expressed genes between patients and healthy volunteers was performed using GEO2R [62], an NCBI web tool that allows comparisons between two or more groups of samples in the GEO series to identify differentially expressed genes across the experimental conditions. The differentially expressed genes were screened according to *p*-values < 0.05. Pearson’s correlation analysis between the genes identified as differentially expressed, following the HGD vs. LGD or the HGD + sEHI vs. HGD, and patients with neurogenerative diseases was performed using ggpubr package in R [61].

Interaction of diet and inhibitor effects was performed using Thermo Fisher Scientific Transcriptome Analysis Console software version 4.0.2. Interaction was defined by a ≥2 or ≤−2 delta fold change and a *p*-value < 0.05 when comparing HGD+sEHI vs. LGD+sEHI to HGD vs. LGD.

### 2.8. Statistical Methods

For microarray, ANOVA ebayes (Thermo Fisher Scientific Transcriptome Analysis Console software, Santa Clara, CA, USA) were used for statistical analysis of microvessel transcriptomes. All genes from the microarray with *p* < 0.05 and ±2.0-fold change were considered as differentially expressed. Mean body weight and plasma lipid levels were expressed as means ± standard error of the mean (SEM), and significance was determined at *p* ≤ 0.05 using unpaired Student’s *t*-tests (GraphPad software, La Jolla, CA, USA).

## 3. Results

The dietary treatment resulted in the expected weight gain in the study mice as follows: The mean weight for male mice at 20 weeks of age, on the chow diet, prior to initiation of the study diets, was 30 g and increased significantly (*p* < 0.05) after 12 weeks in both diet groups (LGD mean 36 g, HGD mean 34g). The soluble epoxide hydrolase inhibitor (sEHI) had no effect on body weight. 

Total cholesterol (TC) levels at the end of the feeding period increased significantly (*p* < 0.05) in all the groups when compared to the baseline measurement at 20 weeks of age and did not statistically differ between the LGD (154.9 mg/dL) and HGD (146.2 mg/dL). The sEHI had no significant effect on total cholesterol levels. 

Glucose and insulin levels also increased significantly (*p* < 0.05) in both the LGD (416.1 mg/dL) and HGD (374.7 mg/dL) treatment groups but did not statistically differ between them. The sEHI had no significant effect on glucose and insulin levels. 

### 3.1. Effect of the High Glycemic Diet on the Hippocampal Microvascular Genome

#### 3.1.1. Global Gene Expression and Hierarchical Clustering

**Figure 2:**
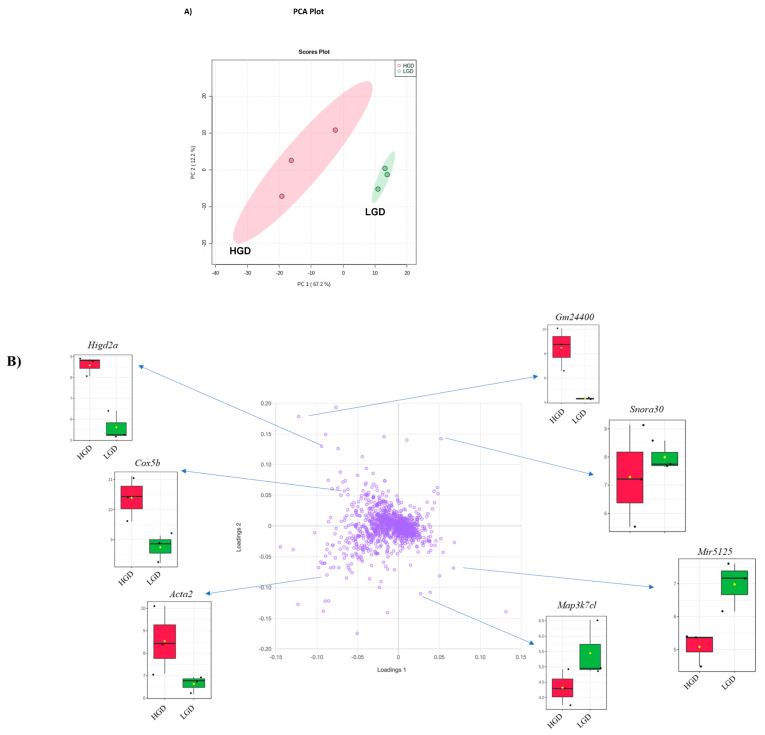
Principal Component Analysis (PCA) and loading plot of genes expressed in hippocampal microvessels of the high glycemic diet (HGD) and the low glycemic diet (LGD). (**A**) PCA scatter plot of the microarray data shows the trends of the expression profiles of the hippocampal microvasculature in the high glycemic diet (HGD, red circles) and low glycemic diet (LGD, green circles), respectively. The PCA plot captures the variance in a dataset in terms of principal components and displays the most significant of these on the x and y axes. The percentages of the total variation that are accounted for by the 1st and 2nd principal components are shown on the x- and y-axes labels. The data are shown for three biological replicates for each dietary group. (**B**) Loading plot of genes (violet circles) relevant to the separation of PCA. Blue arrows show box plots with expression levels of a few genes (*Higd2a, Gm24400, Snora30, Cox5b, Mir5125, Acta2, Map3k7cl, Snora30* and *Map3k7cl*) that contribute to separation of the two dietary groups (HGD, LGD). In the box plots, the black dots represent gene expression levels, the notch indicates 95% confidence interval around the median of each group, and yellow diamond indicates the average gene expression of each group.

To define the molecular mechanisms in brain hippocampal microvessels in response to the HGD, we began by assessing global gene expression using principal component analysis (PCA), a genetic distance visualization tool that shows relatedness between populations. PCA plot analysis showed that the global gene expression profiles of mice on the HDG and LGD were distinctly different from each other (Figure 2A). Using loading plot analysis, we further defined that the genes relevant to the separation of the PCA had opposite expression patterns with the HGD vs the LGD diet. Plots for a few genes (*Higd2a*, *Gm24400*, *Snora30*, *Cox5b*, *Mir5125*, *Acta2*, and *Map3k7cl*) that contributed to separation of the two dietary groups are provided as examples in Figure 2B.

**Figure 3:**
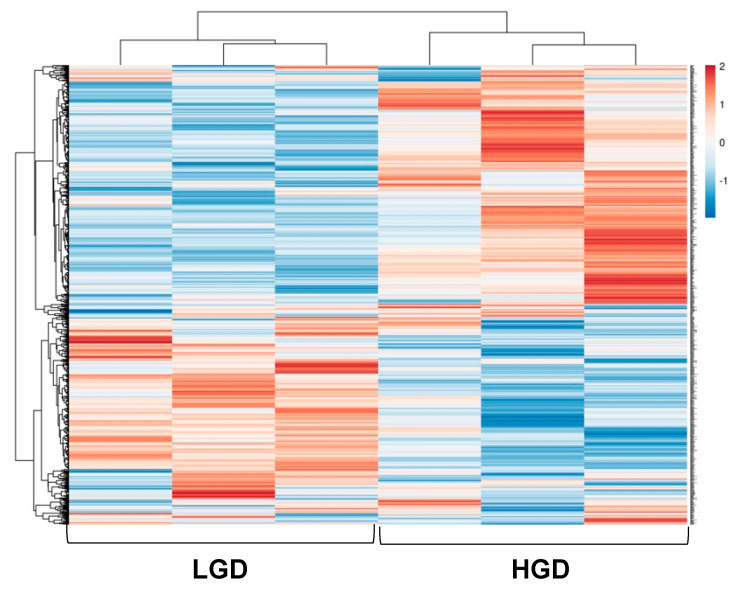
Hierarchical clustering of differentially expressed genes in hippocampal microvessels of the low glycemic diet (LGD) and the high glycemic diet (HGD). Upregulated genes are in red and downregulated genes are in blue. The data are shown for three biological replicates for each dietary group.

We then performed hierarchical clustering of global gene expression profiles. Hierarchical clustering groups similar data points together and then organizes the clusters into a hierarchy. Using this strategy, we further confirmed that genes with higher levels of expression with the HGD had lower levels of expression with the LGD, and vice versa (Figure 3). Thus, the effect of the HGD on global hippocampal microvascular gene expression was fundamentally opposite compared to the LGD.

#### 3.1.2. Differential Gene Expression

To study the effect of the HGD on gene expression, we compared the HGD to the LGD (flow chart Figure 1A). Statistical analysis of microarray data revealed 608 differentially expressed genes (DEGs) in hippocampal microvessels following the HGD, with the majority of the DEGs being up-regulated by the HGD (468 genes up-regulated vs. 140 genes down-regulated) when compared to the LGD, Supplemental Figure S1. The fold-change varied from 2.0 to 25.94 for up-regulated genes and from −16.65 to −2.01 for down-regulated genes (see Supplemental Table S1 for a complete listing of the DEGs). To our knowledge, we also show for the first time that the HGD regulates the expression of protein-coding genes (221) and non-coding genes (78) in male murine hippocampal microvessels (Supplemental Figure S2), including 32 long non-coding RNAs (lncRNAs), 25 microRNAs (miRNAs), and 21 small nucleolar RNAs (snoRNAs). The remaining 309 DEGs were other genes (pseudogenes, ribosomal RNAs (rRNAs), unassigned genes, and multiple-complex genes).

#### 3.1.3. Pathways and Networks for Coding and Non-Coding Differentially Expressed Genes

Next, we performed bioinformatic analysis to find cellular pathways involving differentially expressed (DE) protein-coding genes. Among the 56 cellular pathways we identified were those involved in the regulation of neurodegenerative diseases (e.g., Alzheimer’s disease), pathways involved in the regulation of cellular energy pathways (e.g., oxidative phosphorylation), and cellular metabolism (e.g., fatty acid metabolism). We also observed several cellular signaling pathways, cell adhesion pathways, as well as other pathways such as cell cycle or protein processing in endoplasmic reticulum (Supplemental Figure S3). Therefore, the HGD led to genomic modification in brain microvasculature pathways primarily by modulating the expression of genes involved in neurodegeneration, metabolism, and cell signaling. We also performed bioinformatics analyses of DEGs to identify potential transcription factors (TFs) whose activity could be modulated by the HGD and result in the observed genomic effects. Enrichr database analysis of the top 25 TFs and the DEGs regulated by them is shown in Supplemental Figure S4. The most statistically significant transcription factors were STAT3 (Signal Transducer and Activator of Transcription 3) involved in focal adhesion, DNMT1 (DNA Methyl Transferase 1), and PPARA (Peroxisome Proliferator-Activated Receptor Alpha) involved in Alzheimer’s disease (Supplemental Table S2). 

Microarray analysis also revealed that the HGD could induce differential expression of non-coding RNAs (miRNAs, lncRNAs, and snoRNAs). Of the 25 DE miRNAs in HGD vs. LGD (Supplemental Table S3), 12 were down-regulated (fold change −5.81 to −2.01) and 13 up-regulated (fold change 2.01 to 25.94). Using MIENTURNET software and database interrogation, we identified 442 potential target genes for 17 of the 25 miRNAs. The network of interactions between these miRNAs and their target genes is presented in Supplemental Figure S5. While most genes were the target of a single miRNA, we showed redundancy in that some genes were targets of two or three different miRNAs. Pathway analyses of miRNA target genes revealed they were involved in pathways regulating cell transduction, cell–cell adhesion, permeability, and neurofunction (Supplemental Figure S6). Eleven of the miRNA target genes pathways were in common with the protein-coding DEG pathways, such as Hypoxia-inducible factor 1 (HIF-1) signaling involved in endothelial cell function. Pathways unique to miRNA targets were primarily related to inflammation and cell signaling, whereas protein-coding DEGs specific pathways were primarily involved in cellular metabolism. 

Together with miRNAs, our analysis also revealed 32 DE lncRNAs (Supplemental Table S4) following the HGD. Among these, 4 were down-regulated (fold-change −16.65 to −2.02), and 28 were up-regulated (fold-change 2.02 to 5.34). Using LncRRIsearch and Rtools CBRC databases, we were able to identify 458 potential target genes for 5 of the 32 lncRNAs (Supplemental Figure S7). Pathway analysis of these target genes showed that they were involved in pathways such as nitric oxide signaling, N-cadherin that regulate vascular endothelial function, as well as Alzheimer’s disease (Supplemental Figure S8), which was also one of the 7 pathways in common with the miRNA targets pathways. 

We also studied the expression of snoRNAs in the HGD compared to LGD. Among the 21 DE snoRNAs, 9 were up-regulated (fold change 2.36 to 19.44), and 12 were down-regulated (fold change −10.37 to −2.07) (Supplemental Table S5). A literature review did not identify any known target genes for the DE snoRNAs.

#### 3.1.4. Integrated Analysis of Differentially Expressed Genes, Key Pathways and Networks

**Figure 4:**
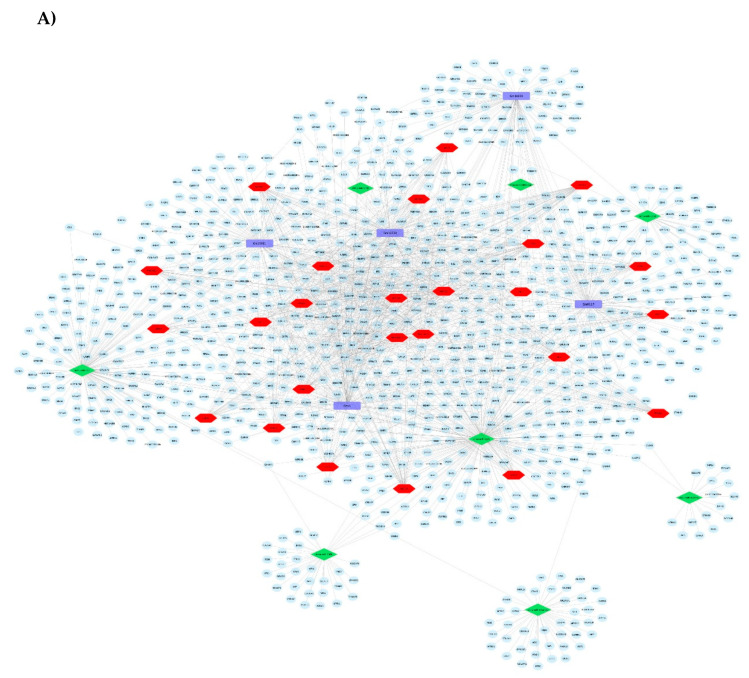

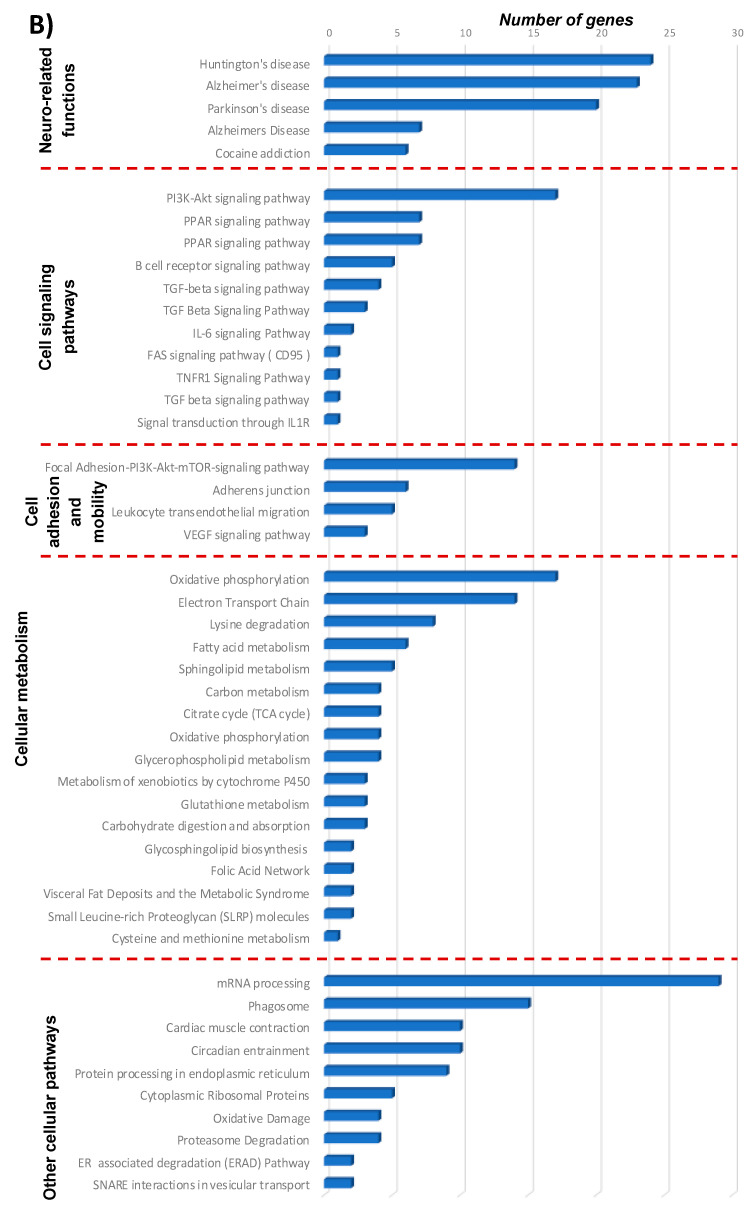

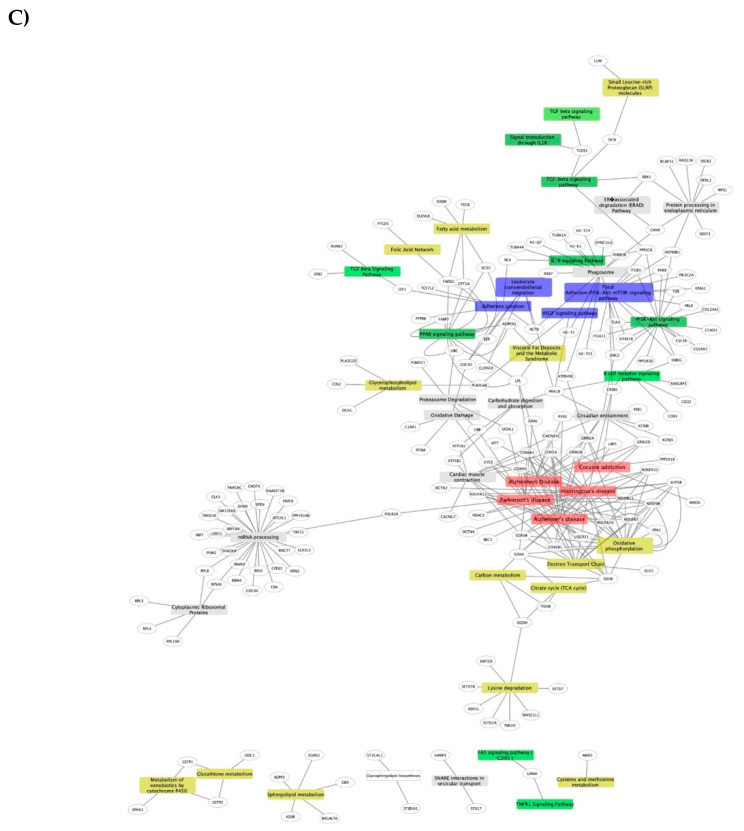

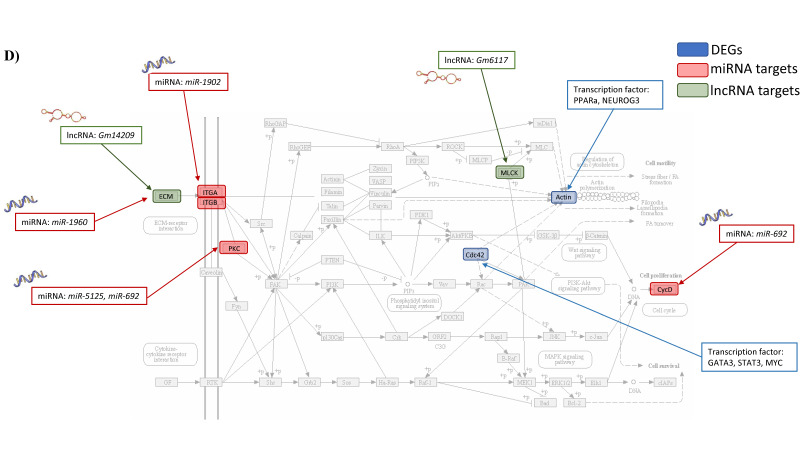

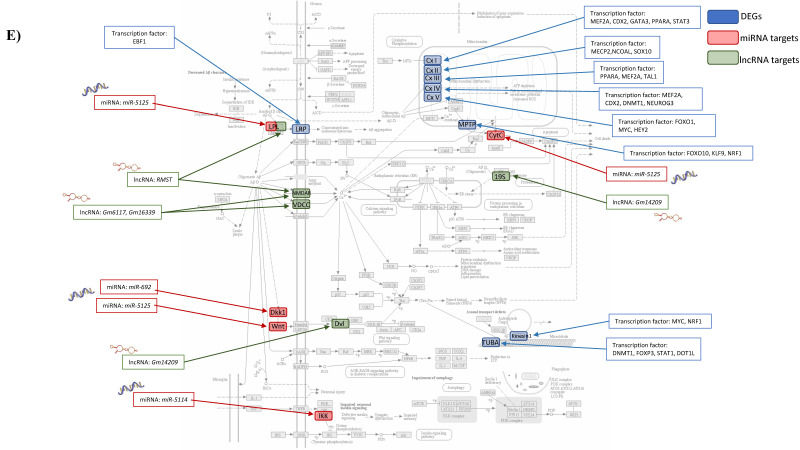
Effect of the high glycemic diet (HGD) on cellular pathways of differentially expressed protein-coding genes, miRNA targets, and LncRNA targets in the hippocampal microvasculature. (**A**) Network of differentially expressed protein-coding genes (grey circles), transcription factors (red hexagons), miRNAs (green diamonds) and their targets (blue circles), LncRNAs (purple rectangles) and their targets (blue circles) in the hippocampal microvessels for the high glycemic diet (HGD) compared to the low glycemic diet (LGD). (**B**) Histogram of a subset of the relevant pathways of differentially expressed protein-coding genes, miRNA targets, and LncRNA targets in the hippocampal microvasculature for the HGD vs LGD. The data are shown for three biological replicates for each dietary group. Statistically significant pathways (*p* < 0.05) were identified using Genetrial2 online database and grouped by cellular function. (**C**) Network of important cellular pathways shown in (**B**) and their genes. Pathways are shown in boxes and color coded based on cellular function such as neuro-related (red), cell signaling (green), cell adhesion and mobility (purple), cellular metabolism (yellow), and other cellular pathways (grey). White circles are differentially expressed genes (DEGs) or target genes of miRNAs and lncRNAs. Integrated analysis of (**D**) Focal adhesion and (**E**) Alzheimer’s disease pathways. Blue=DEGs with potential transcription factors (TFs); Red= differentially expressed miRNAs and their targets; Green= differentially expressed lncRNAs and their targets.

Following the individual omic analysis, we performed integrated analysis of all the identified DEGs including mRNAs, miRNAs and their targets, lncRNAs and their targets, and the identified potential transcription factors. This analysis allowed us to obtain networks of interactions (Figure 4A), and showed that in comparison to the LGD, the HGD significantly impacted the expression levels of different RNA types which interact and form a large molecular network. This molecular network can have a significant effect on cellular functions. Therefore, in order to determine if there was a pattern of functional coordination in the molecular differential expression pattern of the HGD on brain microvessels, we performed integrated pathway and network analysis using DE mRNAs, miRNAs and lncRNAs targets for the HGD compared to LGD. This analysis indeed revealed differential regulation of 5 key cellular pathways including for neurodegenerative diseases (such as Alzheimer’s disease), cell signaling pathways (such as PPAR signaling and phosphoinositide-3-kinase-protein kinase B (PI3K-Akt) signaling), cell adhesion and mobility (including focal adhesion), cellular metabolism (including oxidative phosphorylation and electron transport chain), and other cellular pathways (such as mRNA processing and oxidative damage) (Figure 4B and 4C). Integrated pathways for DEGs, transcription factors, miRNAs and their targets, and lncRNAs and their targets are shown for the focal adhesion pathway (Figure 4D) and the Alzheimer’s disease pathway (Figure 4E). Pathways are discussed in further detail in the Discussion section.

### 3.2. Effect of the Soluble Epoxide Hydrolase Inhibitor (sEHI) on the Hippocampal Microvascular Genome of Mice fed the High Glycemic Diet 

**Figure 5:**
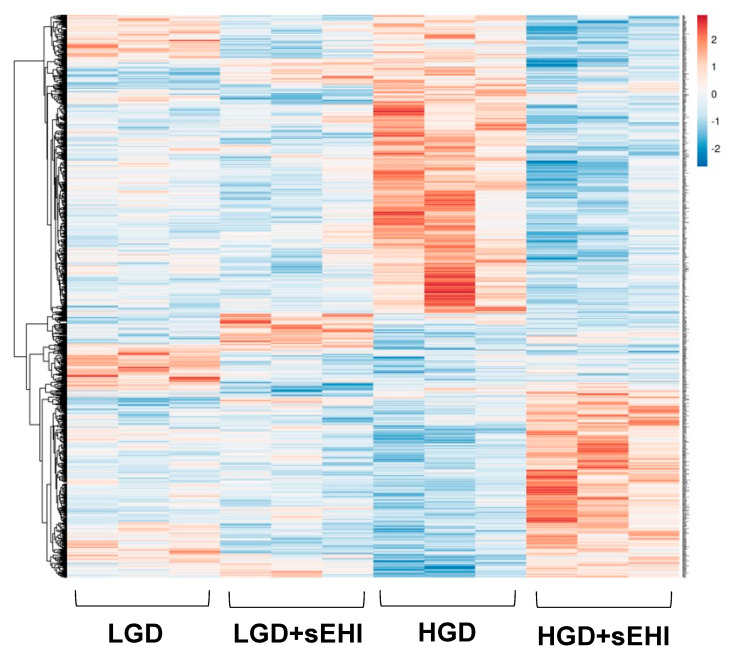
Hierarchical clustering of differentially expressed genes in hippocampal microvessels for the low glycemic diet (LGD) and the high glycemic diet (HGD) with and without soluble epoxide hydrolase inhibitor (sEHI). Hierarchical clustering of differentially expressed gene profiles in hippocampal microvessels of four experimental treatment groups: low glycemic diet (LGD), LGD with soluble epoxide hydrolase inhibitor (LGD+sEHI), high glycemic diet (HGD), and HGD with sEHI (HGD+sEHI). The data are shown for three biological replicates for each dietary group. Up-regulated genes are in red, and down-regulated genes are in blue. In the presence or absence of inhibitor, LGD groups had similar gene expression profiles while HGD groups had opposite gene expression profiles.

In order to determine whether the soluble epoxide hydrolase inhibitor (sEHI) could inhibit the seemingly deleterious molecular effects of the HGD on hippocampal microvessels (upregulation of genes in pathways such as PPAR signaling, PI3K-Akt signaling that play an important role in oxidative stress, inflammation and Alzheimer’s disease), we again performed hierarchical clustering of global gene expression profiles for the LGD and the HGD in the presence of the inhibitor (Figure 5). Since the gene expression profile of the LGD group did not substantially differ from the LGD with the inhibitor (Figure 5), the effect of the inhibitor was primarily analyzed in reference to the HGD in our analytic comparisons. Interestingly, in the presence of the sEHI, the gene expression profile of the HGD on hippocampal microvessels was nearly completely reversed and similar to that on the LGD.

**Figure 6:**
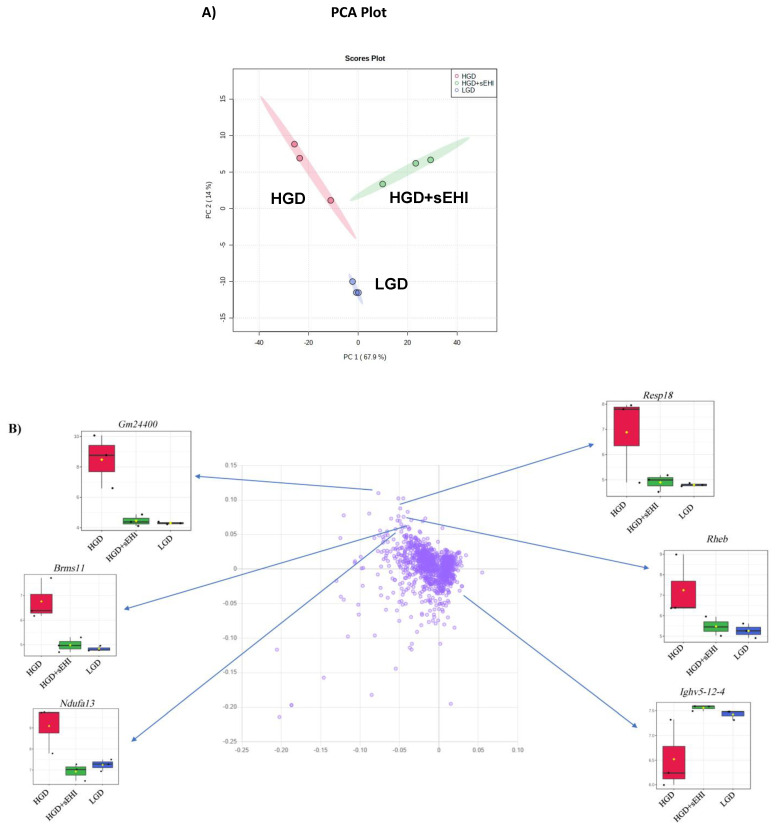
Principal Component Analysis (PCA) and loading plot of genes expressed in hippocampal microvessels for the high glycemic diet (HGD) with and without soluble epoxide hydrolase inhibitor (sEHI). (**A**) PCA scatter plot of the microarray data shows the trends of the expression profiles of the hippocampal microvasculature of the high glycemic diet with (HGD+sEHI, green circles) and without (HGD, red circles) the soluble epoxide hydrolase inhibitor compared to the low glycemic diet (LGD, blue circles). The PCA plot captures the variance in a dataset in terms of principal components and displays the most significant of these on the x and y axes. The percentages of the total variation that are accounted for by the 1st and 2nd principal components are shown on the x- and y-axes labels. The data are shown for three biological replicates for each dietary group. (**B**) Loading plot of genes (violet circles) relevant to the separation of PCA. Blue arrows show box plots with the expression levels of a few genes (*Gm24400, Resp18, Rheb, Brms1l, Ndufa13, Ighv5-12-4*) that contribute to separation of the three dietary groups (HGD, HGD+sEHI, LGD). In the box plots, the black dots represent gene expression levels, the notch indicates 95% confidence interval around the median of each group, and the yellow diamond indicates the average gene expression of each group.

Using PCA analysis, we were further able to show that the gene expression profile of hippocampal microvessels following the HGD was distinctly different from that of the HGD+sEHI (Figure 6A). We used loading plot to identify genes important to the separation of PCA (Figure 6B). Genes such as *Gm24400, Resp18, Rheb, Brms1l, Ndufa13,* and *Ighv5-12-4* showed opposite expression in the HGD with inhibitor when compared to the HGD alone.

**Figure 7:**
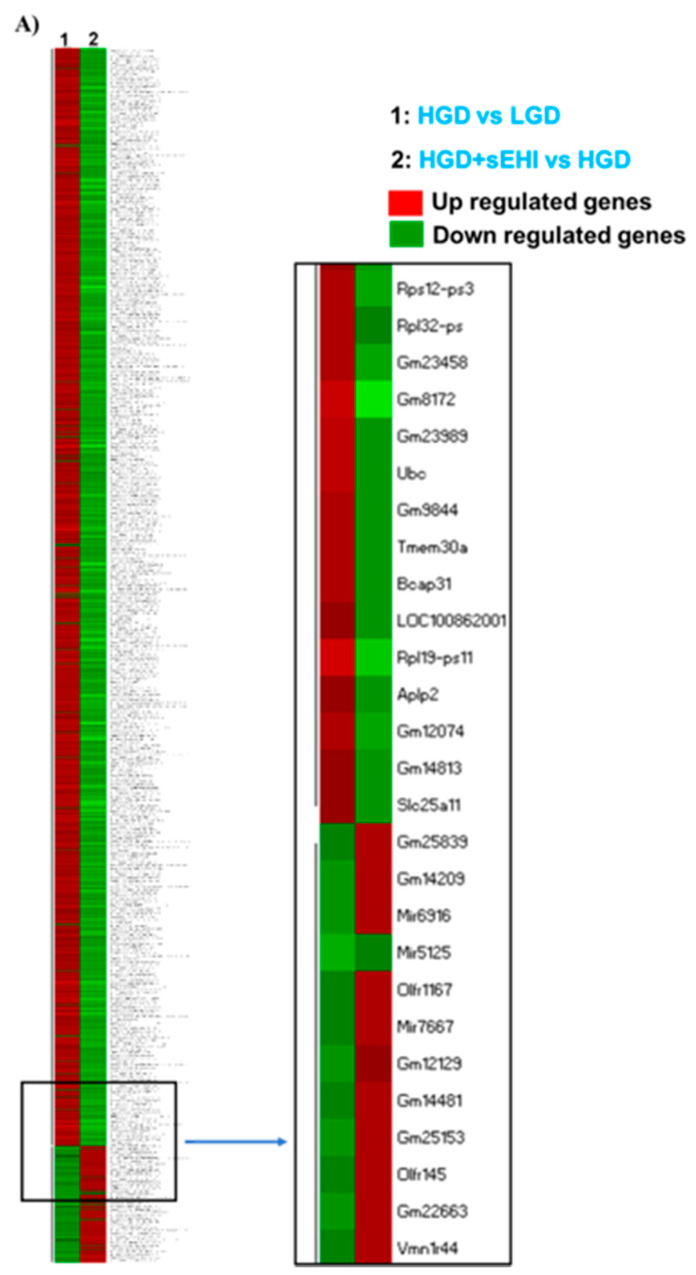

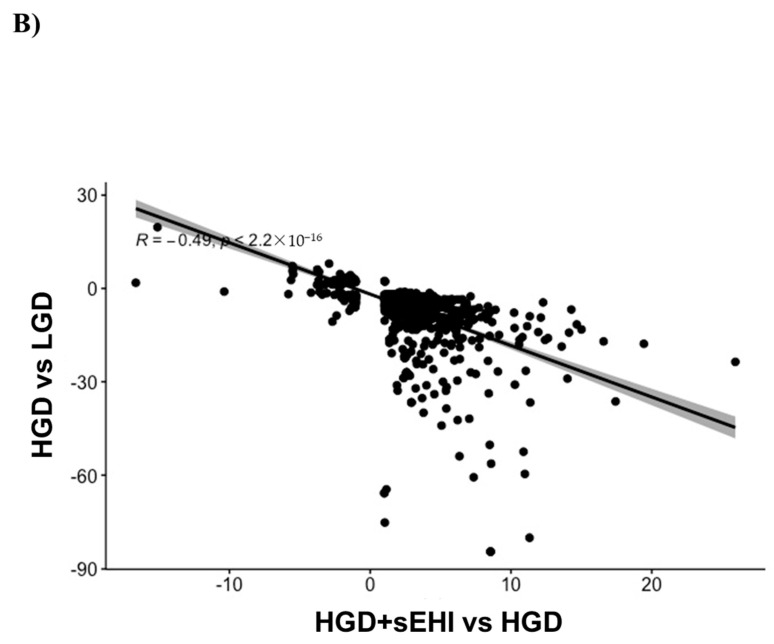
The effect of the high glycemic diet with and without soluble epoxide hydrolase inhibitor (sEHI) on differential gene expression in the hippocampal microvasculature. (**A**) Heat map showing individual differentially expressed genes (DEGs) in rows, and the two different experimental dietary comparison groups in columns, as follows: column 1: high glycemic diet compared to low glycemic diet (HGD vs LGD); column 2: HGD with soluble epoxide hydrolase inhibitor compared to without inhibitor (HGD+sEHI vs HGD). The data are shown for three biological replicates for each dietary group. Up-regulated genes are shown in red, and down-regulated genes in green. The rectangular box insert shows the list of genes with opposite gene expression patterns in the two comparisons (HGD vs. LGD compared to HGD+sEHI vs. HGD). (**B**) Pearson’s correlation analysis of the DEGs between the comparisons, HGD vs. LGD and HGD+sEHI vs. HGD.

Statistical analysis of microarray data showed that there were a larger number of DEGs (1701) for the comparison of the HGD+sEHI vs. HGD (Supplemental Table S6) than there were for the HGD vs. LGD comparison. The sEHI primarily down-regulated both protein-coding and non-coding DEGs in the HGD (Figure 7A, and Supplemental Figures S9 and S10). We then performed correlation analysis between the DEGs of the HGD+sEHI vs. HGD and the HGD vs. LGD and identified a highly significant negative correlation with the sEHI (Figure 7B). This suggests that the inhibitor counteracts the effects of the HGD on differential gene expression.

Most of the protein-coding genes down-regulated by the sEHI were involved in similar pathways (such as Alzheimer’s disease, oxidative phosphorylation, and fatty acid metabolism) activated by the HGD alone (Supplemental Figure S11), suggesting that the inhibitor may offset the effect of the HGD on differential gene expression by targeting similar pathways. We also found 17 TFs in common between the HGD with and without inhibitors such as NEUROG3 (Neurogenin 3), MECP2 (Methyl-CpG Binding Protein 2), KLF9 (Kruppel Like Factor 9), and PPARA (Supplemental Table S2). Target genes of these TFs were up-regulated by the HGD but down-regulated by the inhibitor (Supplemental Figure S12). These target genes were involved in neurological disease pathways (e.g., Alzheimer’s disease), oxidative phosphorylation (e.g., electron transport chain), apoptosis-related pathways (e.g., proteasome degradation), and cell signaling pathways (e.g., HF-1 signaling). 

A higher number of DE non-coding RNAs (171 miRNAs, 127 snoRNAs, and 80 lncRNAs) were modulated by the sEHI (Supplemental Tables S3, S4, and S5, respectively) than the HGD alone. Most of the DE miRNAs that were up-regulated by the HGD were down-regulated by the inhibitor by targeting 300 genes (Supplemental Figure S13) that were involved in apoptosis-related pathways (such as PTEN dependent cell cycle arrest and Ubiquitin mediated proteolysis) (Supplemental Figure S14) and pathways related to cell degradation and death. The majority of DE lncRNAs that were up-regulated by the HGD were down-regulated by the inhibitor and again targeted a greater number of genes (785) compared to the HGD alone (Supplemental Figure S15) and were mainly involved in cell signaling (Supplemental Figure S16). 

**Figure 8:**
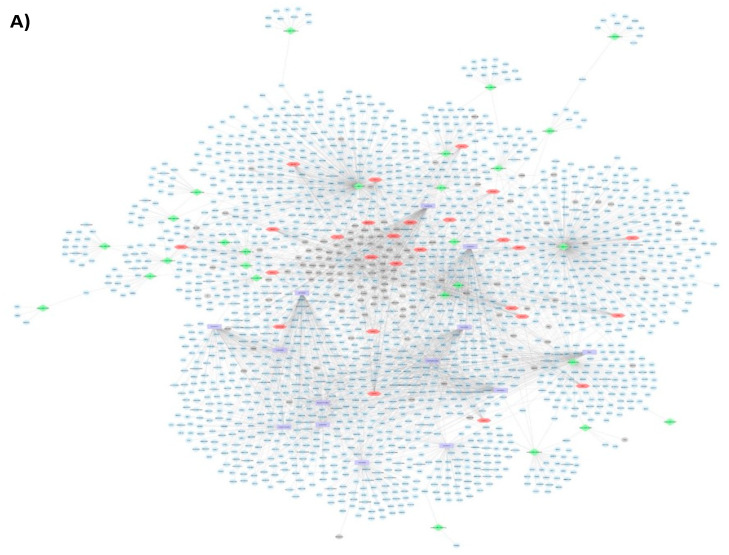

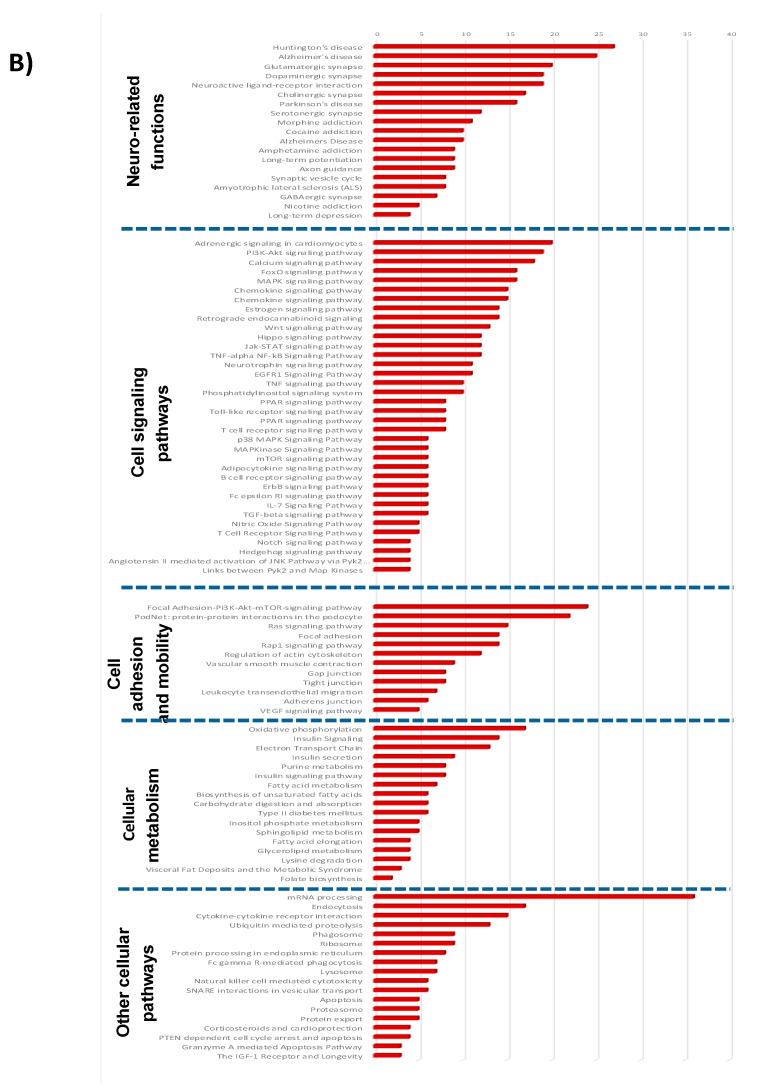
Effect of the high glycemic diet (HGD) with soluble epoxide hydrolase inhibitor (sEHI) on cellular pathways of differentially expressed protein-coding genes, miRNA targets, and LncRNA targets in the hippocampal microvasculature. (**A**) Network of differentially expressed protein-coding genes (grey circles), transcription factors (red hexagons), miRNAs (green diamonds) and their targets (blue circles), LncRNAs (purple rectangles) and their targets (blue circles) in the hippocampal microvessels for the high glycemic diet (HGD) with soluble epoxide hydrolase inhibitor (sEHI) when compared to HGD without sEHI. (**B**) Histogram of subset of relevant pathways of differentially expressed protein-coding genes, miRNA targets, and LncRNA targets in the hippocampal microvasculature for the HGD+sEHI vs HGD. The data are shown for three biological replicates for each dietary group. Statistically significant pathways (*p* < 0.05) were identified using Genetrial2 online database and are grouped by cellular function such as neuro-related, cell signaling, cell adhesion and mobility, cellular metabolism, and other.

We then performed an integrated analysis of all the identified DEGs, including mRNAs, miRNAs and their targets, lncRNAs and their targets, and the potential TFs. We obtained a large molecular network of interactions of the different types of RNAs modulated by the inhibitor (Figure 8A). Integrated pathway analysis of this molecular network (Figure 8B) revealed that the inhibitor regulated similar pathways as the HGD, namely neuro-related pathways (such as Alzheimer’s disease), cell signaling, cell adhesion (such as focal adhesion), and cellular metabolism, but once again, in opposite directions to the HGD. In toto, these data suggest that the inhibitor may counteract the effect of an HGD by robustly reversing the deleterious pattern of differential gene expression on cellular function and on the operative TFs, particularly by down-regulating the protein-coding and non-protein-coding DEGs that are up-regulated by the HGD.

### 3.3. Correlation of Genomics with Human Alzheimer’s Disease Data

**Figure 9:**
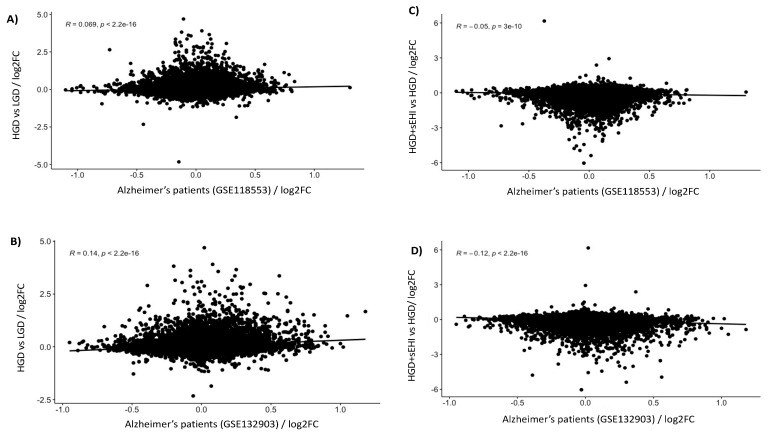
Correlation of the high glycemic diet (HGD) with and without inhibitor to Alzheimer’s disease. Pearson correlation analysis between the genes modulated by the HGD vs LGD and the gene expression profiles observed in patients with Alzheimer’s disease extracted from GEO database number (**A**) *GSE118553* and (**B**) *GSE132903*. Correlation between genes modulated by the HGD+sEHI vs LGD and patients with Alzheimer’s disease extracted from GEO database number (**C**) *GSE118553* and (**D**) *GSE132903*.

To evaluate the genomic modifications induced by the HGD on the development of neurodegenerative diseases, we analyzed the correlations between the genes modulated by the HGD and the genes associated with Alzheimer’s disease. We analyzed the available GEO datasets with GEO2R and differentially expressed mRNAs were correlated with those extracted from the analyzed publications using the R program. These analyses showed that the gene expression profile in the brain of patients with Alzheimer’s disease (GSE118553) was correlated, with high statistical significance, to the gene expression profile identified in our mice after the consumption of the HGD (Figure 9A). The gene expression profile of mice on the HGD in our study was also correlated with the gene expression profile obtained in another study (GSE132903) of brains of patients presenting with Alzheimer’s disease (Figure 9B). This analysis suggests that consumption of the HGD induces changes in the expression of genes in murine hippocampus that correlates with the development of Alzheimer’s disease in humans. Using the same approach, we also tested correlations between the genes modulated in our study by the HGD in the presence of the sEHI and the gene expression profiles obtained in both of the above noted genomic studies using the brains of patients with Alzheimer’s disease. Interestingly, we observed a negative correlation between the human AD data and the gene expression profile of our brain microvasculature for animals on the HGD with the sEHI (Figure 9C,D). These results suggest that the inhibitor can prevent, at least partially, changes in the expression of genes induced by the HGD that are associated with the development of Alzheimer’s disease in humans and can therefore exert a potentially protective effect against this neurological disorder.

### 3.4. Interaction of Effects of the Glycemic Diet and the Soluble Epoxide Hydrolase Inhibitor 

**Figure 10:**
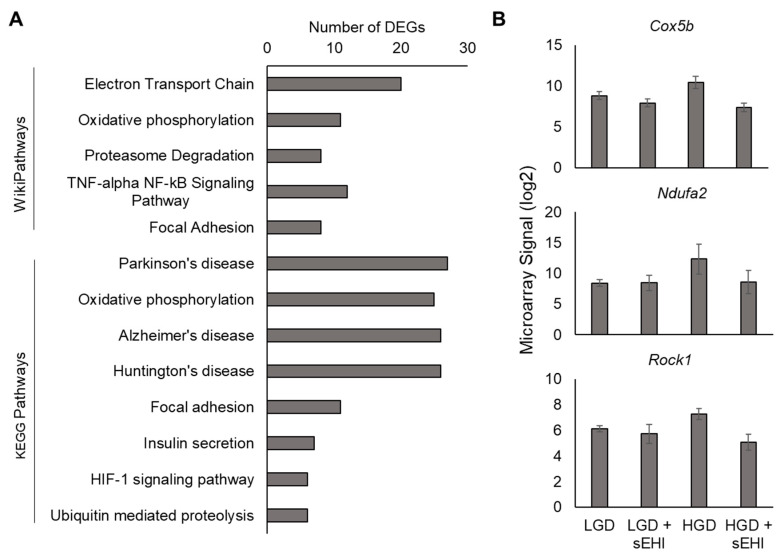
Analysis of DEGs with a significant interaction of diet and inhibitor. (**A**) Relevant enriched WikiPathways and KEGG Pathways for DEGs with an interaction between diet and inhibitor effects. (**B**) Signal plots of selected DEGs with an interaction between diet and inhibitor effects. Data shown are from three biological replicates (mean +/− standard deviation). The DEGs displayed are cytochrome c oxidase subunit Vb (Cox5b), NADH dehydrogenase (ubiquinone) 1 alpha subcomplex 2 (Ndufa2), and Rho-associated coiled-coil containing protein kinase 1 (Rock1).

In order to more clearly define how the sEHI modulated the response of hippocampal microvessels to an HGD, we used two-factor analysis to identify the DEGs with a significant interaction between the effects of the diet and the effects of the inhibitor. For this analysis, the LGD+sEHI group was included as it was necessary to use this additional control group for the two-factor analysis and to determine the delta fold change [(HGD+sEHI vs. LGD+sEHI) vs. (HGD vs. LGD)]. A positive delta fold change indicates a gene that is “more up-regulated” or “less down-regulated” by HGD in sEHI treated mice (compared to those with no inhibitor). A negative delta fold change indicates a gene that is “more down-regulated” or “less up-regulated” by an HGD in sEHI treated mice (compared to those with no inhibitor). We identified a total of 1543 DEGs (Supplemental Table S7) that had a significant interaction between diet and inhibitor, the majority of which had a negative delta fold change. In many cases, the direction of the regulation of the DEGs by the HGD was opposite in direction in the presence of the inhibitor when compared to the effects of the HGD on that same gene in the absence of the inhibitor. The DEGs with a negative delta fold change were diversified between RNA types, including coding, non-coding, multiple complex, and pseudogenes. The 583 DEGs with a positive delta fold change were primarily non-coding genes. Pathway analyses using the KEGG and WikiPathway databases for all of the coding DEGs (positive and negative delta fold change) demonstrated that the sEHI modulated the effects of the HGD for pathways involved in the electron transport chain, oxidative phosphorylation, focal adhesion, HIF-1 signaling pathway, and pathways active in neurological diseases (Alzheimer’s pathway) (Figure 10A). 

An additional finding of the two-factor analysis was that when the individual DEGs of the relevant pathways shown in Figure 10A (77 DEGs) were examined, it again became apparent that the sEHI reverted the gene expression profile of brain hippocampal microvascular endothelium of mice on the HGD to a profile similar to that of the LGD. Examples of three genes (*Rock1, Ndufa2, and Cox5b*) are shown in Figure 10B. These DEGs were selected because they were each represented in multiple relevant pathways including focal adhesion (*Rock1*), electron transport chain (*Ndufa2* and *Cox5b*), and oxidative phosphorylation (*Ndufa2* and *Cox5b*). 

## 4. Discussion

In this study, we characterized the effect of a high glycemic diet (HGD) on murine neurovascular function and cognition using hippocampal microvascular multiomics of mice exposed to high or low glycemic index diets. Utilizing similar techniques of large-scale transcriptome gene profiling, we have previously shown the effect of lipid stress and a Western diet (WD) on hippocampal microvascular injury and cognitive decline in male and female mice ApoE-/- and LDL-R-/- mice [8,9,10,11]. However, the molecular footprint of the glycemic component of the WD on the brain microvasculature, and whether and how the HGD may contribute to microvascular injury, was up to now unknown. A HGD is a risk factor for dementia [14] and is associated with poor cognitive performance [15,16], but whether the effect can be reversed has also been unknown. Given that in murine models of diabetes, soluble epoxide hydrolase inhibitors (sEHI) reduce neuroinflammation and cognitive decline [32,33,34], we also aimed to examine their role in our model. 

We used a relevant model of glycemic index and demonstrated the expected significant differences in weight, cholesterol, glucose, and insulin levels consistent with what has been published previously for these experimental models [19,63,64,65]. To our knowledge, the present study shows for the first time that the HGD has distinct differential effects on gene expression that are different from the LGD and characterized by up-regulation of differentially expressed protein-coding genes, transcription factors, as well as non-protein-coding genes (miRNAs, snoRNAs, and lncRNAs), that are involved in five major cellular pathways: neurodegenerative diseases (Alzheimer’s disease), endothelial cell function (focal adhesion), cell signaling (PPAR signaling, PI3K-Akt signaling), cellular metabolism (oxidative phosphorylation, electron transport chain), and other cellular pathways (mRNA processing, oxidative damage). Below we discuss our multi-omics integrative analysis for the focal adhesion and Alzheimer’s disease pathways to illustrate the complexity and characterize the molecular regulation in response to the HGD. 

Integrative analyses allowed us to identify the protein-coding genes, TFs, and the non-coding genes modulated by the HGD in the focal adhesion pathway. *Cdc42* (Cell Division Control Protein Homolog 42) is a protein-coding DEG up-regulated by the HGD and targeted by STAT3 (Signal Transducer and Activator of Transcription 3) transcription factor. *Cdc42* is up-regulated in aging endothelial cells via the focal adhesion pathway [66,67] and mediates the activation of proinflammatory genes [68]. STAT3 transcription factor is activated by oxidative stress [69,70] and is associated with neurovascular inflammation and Alzheimer’s disease [71]. The HGD also up-regulated differentially expressed miRNA *miR-1902* that targets *ITGB* (Integrin β). Activation of ITGB induces a reduction in endothelial barrier function by destabilizing intercellular junctions and abnormal remodeling of extracellular matrix (ECM) [72]. Another example of differentially expressed miRNAs up-regulated by the HGD is *miR-5125* and *miR-692* which target *PKC* (Protein Kinase C). PKC is associated with endothelial dysfunction, insulin resistance [73], and neuroinflammation [74]. The HGD also up-regulated differential expression of LncRNA *Gm6117* that targets *MLCK* (Myosin Light Chain Kinase). Activation of MLCK induces endothelial vascular hyperpermeability, microvascular barrier dysfunction, and inflammation [75]. Another interesting pathway activated by the HGD was the vascular endothelial growth factor (VEGF) signaling pathway. VEGF is associated with an increase in microvascular permeability by activating PKC [76], which then signals the activation of MLCK [75]. Taken together, this molecular cascade suggests that an HGD may promote endothelial cell dysfunction by microvascular hyper permeability and activating proinflammatory TFs, protein, and non-protein-coding genes. 

We also performed an integrated analysis of the Alzheimer’s disease pathway and identified TFs, protein-coding, and non-coding RNAs modulated by the HGD. Mitochondrial *complex IV* protein-coding DEG was up-regulated by the HGD and targeted by DNMT1 (DNA Methyl Transferase 1) transcription factor. Increased expression of *complex IV* has been observed in the hippocampus of AD patients [77], and DNMT1 expression is up-regulated in late-onset AD [78]. Alterations in mitochondrial function are also associated with diabetes [79]. Another example of a DEG up-regulated by the HGD was *LRP1* (LDLR-related protein). Expression of *LRP1* is increased in the hippocampus of AD patients [80], particularly in the microvasculature close to amyloid plaques in AD brains [81,82]. DE miRNA, *miR-5125* was activated by the HGD and is known to target *DKK1* (Dickkopf-1). Increased expression of *DKK1* has been detected in the plasma and brain of AD patients and AD transgenic mice [83,84]. Another target of *miR-5125* was *LPL* (Lipoprotein lipase). LPL accumulates in senile plaques [85], and its expression has been shown to be increased in the hippocampus of AD mice [86]. LncRNAs *Gm6117* and *Gm16339* were also up-regulated by the HGD and target *NMDAR* (N-methyl-D-aspartate receptor). An increase in NMDAR activity induces apoptosis and neurodegeneration in AD [87]. Yet another interesting pathway modulated by the HGD was the PI3K-Akt signaling pathway. Activation of the PI3K/Akt pathway promotes oxidative stress-mediated cell death [88]. Thus, our findings suggest that an HGD may be involved in previously defined Alzheimer’s disease-related pathways via deleterious effects on the brain microvasculature by promoting mitochondrial dysfunction, apoptosis, and neurodegeneration, and similar to the focal adhesion pathway, via up-regulation of pathway-associated TFs, protein-coding, and non-coding genes.

Our work with the soluble epoxide hydrolase inhibitor (sEHI) trans-4-[4-(3-adamantan-1-yl-ureido)-cyclohexyloxy]-benzoic acid (t-AUCB) showed several novel findings. sEH enzyme degrades anti-inflammatory and neuroprotective fatty acid epoxides (EETs) to biologically less active diols (DHETs) which are known to be involved in vascular cognitive impairment and Alzheimer’s disease [24,25]. In animal models of stroke, sEH inhibitors have been shown to be neuroprotective [27,28]. In our study, we show for the first time that the sEHI reversed the deleterious phenotype of the HGD by down-regulating the differentially expressed genes up-regulated by the HGD and their TFs, and also by differential expression of non-coding RNAs (miRNA, snoRNA, and lncRNA) and that this proceeds by targeting the same pathways up-regulated by the HGD. When looking at specific DEGs targeted by the inhibitor, we identified interesting DEGs. For example, the presence of the sEHI down-regulated the expression of *Cdc42*, and the deletion of *Cdc42* is known to have protective effects against chronic inflammation in endothelial cells [67]. The sEHI also down-regulated the expression of the genes targeted by the PPAR (Peroxisome Proliferator-Activated Receptor) transcription factor, which is known to play a protective role against neuroinflammation in AD [89]. Furthermore, the sEHI down-regulated DE miRNAs, *miR-5125,* and *miR-692*, and their targets, *DKK1* and *PKC*, respectively. Inhibition of DKK1 has been shown to improve cognitive impairment [90] and protect against neurotoxicity [91]. PKC inhibition also protects against vascular injury of the blood–brain barrier [74]. Additionally, the sEHI also down-regulated DE lncRNA *Gm6117* and its target, *MLCK*. MLCK inhibitors play a protective role against vascular hyperpermeability and microvascular dysfunction [75]. These findings indicate that the sEHI served to protect against the neurotoxicity, endothelial- and neuro-inflammation, and blood– brain barrier permeability injury associated with the HGD. 

Furthermore, our correlation analysis of the DEGs modulated by the HGD and inhibitor with the gene expression profiles obtained from the brains of Alzheimer’s disease patients showed that the HGD is positively correlated, and the inhibitor is negatively correlated with the genes associated with Alzheimer’s disease.

Our 2-factor interactive analysis served to further identify DEGs having a significant interaction between diet and the soluble epoxide hydrolase inhibitor. These DEGs were involved in Alzheimer’s disease, electron transport chain, oxidative phosphorylation, and focal adhesion. The sEHI also was shown to restore the gene expression profile of hippocampal microvessels of mice on the HGD to a profile similar to that of the LGD, including for genes such as *Rock1* (Rho-associated protein kinase 1), which was increased by an HGD and decreased by the sEHI. *ROCK1* is activated in Alzheimer’s disease, and reduction of *ROCK1* protects against AD by depleting amyloid-β levels in the brain [92]. In vitro exposure of brain microvascular endothelial cells to EETs, which generally increase after inhibition of sEH, has been shown to inhibit rho kinase (ROCK) activity [93]. Taken together, our molecular results suggest that the soluble epoxide hydrolase inhibitor may offset the effect of an HGD by robustly reversing the deleterious pattern of differential gene expression to that similar to an LGD and likely thereby protecting against vascular injury, neuroinflammation, and neurodegeneration. Furthermore, the sEHI was associated with improved cognition by down-regulating the TFs, protein-coding, and non-protein-coding DEGs that were up-regulated by the HGD. 

## 5. Conclusions and Clinical Implications

In alignment with our hypothesis, our results show that an HGD has deleterious effects on differential gene expression of brain microvessels in memory centers in the brain by up-regulating the protein-coding and non-coding genes involved in alterations of mitochondrial function, oxidation, inflammation, and microvascular dysfunction. These processes play an important role in the pathophysiology of dementia. We also showed that inhibition of sEH protects against neuroinflammation, apoptosis, vascular injury, and cognitive decline by down-regulating the DEGs up-regulated by an HGD. The clinical role of sEHIs has been reviewed [22]. In clinical trials, sEHIs play a protective role in hypertension [94], and in animal models, they demonstrate neuroprotection in stroke [27,28], cerebral hypoperfusion, and in diabetes type 1 and type 2 [29,30,31,32,33]. Our studies show that sEHIs may also be promising therapeutic targets in the microvascular endothelial dysfunction that accompanies Alzheimer’s and vascular dementias.

## Data Availability

We have deposited the microarray data in GEO and the accession number is GSE185057.

## Supplementary Materials

The following are available online at https://www.mdpi.com/article/10.3390/nu13113913/s1, Figure S1: Volcano plot of differential gene expression changes in hippocampal microvessels for the high glycemic diet (HGD) compared to the low glycemic diet (LGD), Figure S2: Distribution of differentially expressed RNAs in hippocampal microvessels for the high glycemic diet (HGD) when compared to the low glycemic diet (LGD), Figure S3: Histogram of differentially expressed protein coding genes pathways in hippocampal microvessels for the high glycemic diet (HGD) when compared to the low glycemic diet (LGD), Figure S4. Target gene networks of differentially expressed transcription factors (TFs) in hippocampal microvessels for the high glycemic diet (HGD) compared to the low glycemic diet (LGD), Figure S5. Target gene networks of differentially expressed miRNAs in hippocampal microvessels with the high glycemic diet (HGD) compared to the low glycemic diet (LGD), Figure S6: Histogram of differentially expressed miRNA targets pathways in hippocampal microvessels with the high glycemic diet (HGD) when compared to the low glycemic diet (LGD), Figure S7. Target gene networks of differentially expressed LncRNAs in hippocampal microvessels with the high glycemic diet (HGD) compared to the low glycemic diet (LGD), Figure S8: Histogram of differentially expressed lncRNA targets pathways in hippocampal microvessels with the high glycemic diet (HGD) when compared to the low glycemic diet (LGD), Figure S9: Volcano plot of differential gene expression changes in hippocampal microvessels for the high glycemic diet (HGD) with soluble epoxide hydrolase inhibitor (sEHI) compared to without sEHI treatment, Figure S10: Distribution of differentially expressed RNAs in hippocampal microvessels from the high glycemic diet (HGD) with soluble epoxide hydrolase inhibitor (sEHI) compared to without sEHI treatment, Figure S11: Histogram of protein coding differentially expressed genes pathways in hippocampal microvessels for the high glycemic diet (HGD) with soluble epoxide hydrolase inhibitor (sEHI) compared to without sEHI treatment, Figure S12: Target gene networks of differentially expressed transcription factors (TFs) in hippocampal microvessels for the high glycemic diet (HGD) with and without soluble epoxide hydrolase inhibitor (sEHI), Figure S13: Target gene networks of differentially expressed miRNAs in hippocampal microvessels for the high glycemic diet (HGD) with and without soluble epoxide hydrolase inhibitor (sEHI), Figure S14: Histogram of differentially expressed miRNA targets pathways in hippocampal microvessels for the HGD with soluble epoxide hydrolase inhibitor (sEHI) compared to without sEHI treatment, Figure S15: Target gene networks of differentially expressed LncRNAs in hippocampal microvessels for the high glycemic diet (HGD) with and without soluble epoxide hydrolase inhibitor (sEHI), Figure S16: Histogram of differentially expressed lncRNA targets pathways in hippocampal microvessels for the high glycemic diet (HGD) with soluble epoxide hydrolase inhibitor (sEHI) compared to without the sEHI; Table S1: Differentially expressed genes for the high glycemic diet (HGD) compared to the low glycemic diet (LGD), Table S2: Top 30 transcription factors (TFs) differentially expressed by a high glycemic diet (HGD) with and without soluble epoxide hydrolase inhibitor (sEHI) in hippocampal microvascular endothelium, Table S3: Effect of the high glycemic diet (HGD) with and without soluble epoxide hydrolase inhibitor (sEHI) on the expression of microRNAs (miRNAs) in hippocampal microvessels, Table S4: Effect of the high glycemic diet (HGD) with and without soluble epoxide hydrolase inhibitor (sEHI) on the expression of long non-coding RNAs (lncRNAs) in hippocampal microvessels, Table S5: Effect of the high glycemic diet (HGD) with and without soluble epoxide hydrolase inhibitor (sEHI) on the expression of small nucleolar RNAs (snoRNAs) in hippocampal microvessels, Table S6: Differentially expressed genes for the high glycemic diet (HGD) with soluble epoxide hydrolase inhibitor (sEHI) compared to without sEHI, Table S7: Differentially expressed genes with a significant interaction of diet and inhibitor.
