# Supplementary material for: Inhibition of Soluble Epoxide Hydrolase Is Protective against the Multiomic Effects of a High Glycemic Diet on Brain Microvascular Inflammation and Cognitive Dysfunction
Source: Nutrients. 2021 Nov 1;13(11):3913. doi: 10.3390/nu13113913 (PMC8622784; doi:10.3390/nu13113913)
Supplement: Supplementary file 1 [file nutrients-13-03913-s001.zip › nutrients-1425072-supplementary.pdf]

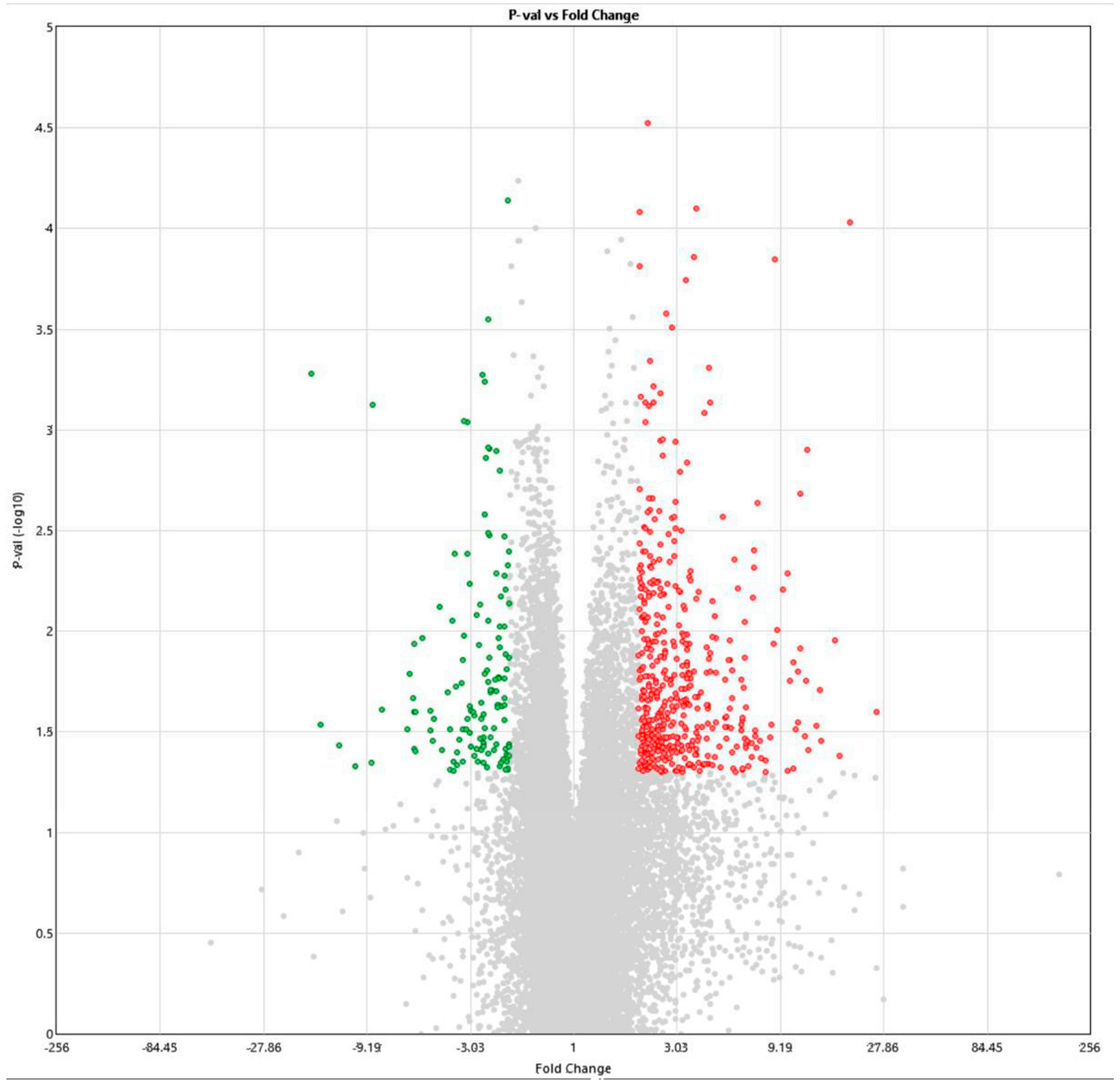

Supplement Figure S1: Volcano plot of differential gene expression changes in hippocampal microvessels for the high glycemic diet (HGD) compared to the low glycemic diet (LGD). The transcriptome of hippocampus microvessels from the high glycemic diet (HGD) compared to low glycemic diet (LGD). The data are shown for three biological replicates for each dietary group. The x-axis specifies the fold-changes (FC), and the y-axis specifies the negative logarithm to the base 10 of the p-values. Filtering criteria for differentially expressed genes (DEGs) is FC = ±2.0 and p-value < 0.05. Red and green dots represent probe sets for transcripts expressed at significantly higher or lower levels than LGD, respectively.

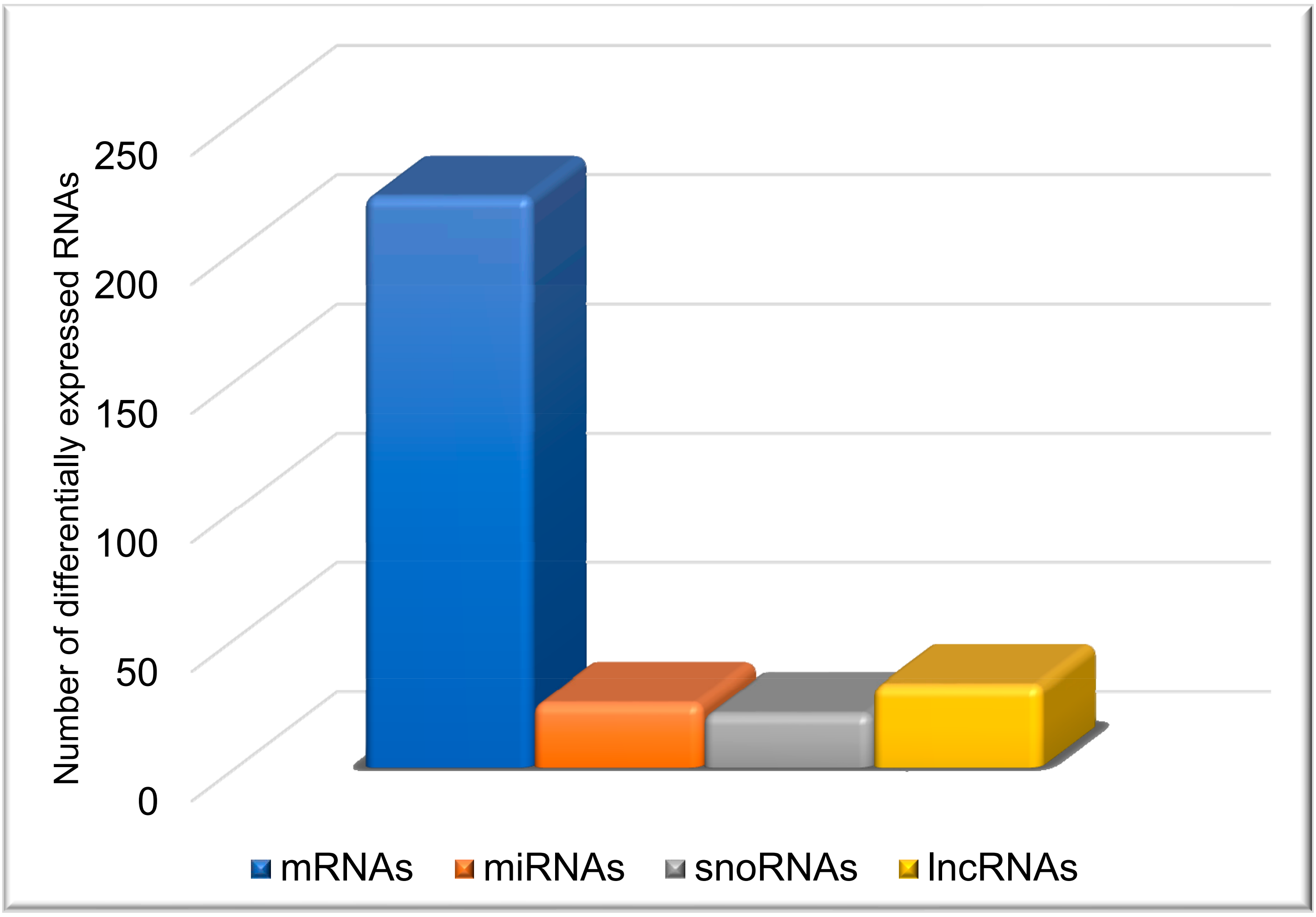

Supplemental Figure S2: Distribution of differentially expressed RNAs in hippocampal microvessels for the high glycemic diet (HGD) when compared to the low glycemic diet (LGD). Number of differentially expressed protein coding (messenger RNAs=mRNAs, blue) and non-protein coding RNAs (microRNAs=miRNAs, orange; small nucleolar RNA=snoRNAs, grey; and long non-coding RNA=lncRNAs, yellow) in hippocampal microvessels from the high glycemic diet (HGD) compared to low glycemic diet (LGD). The data are shown for three biological replicates for each dietary group.

Supplemental Figure S3

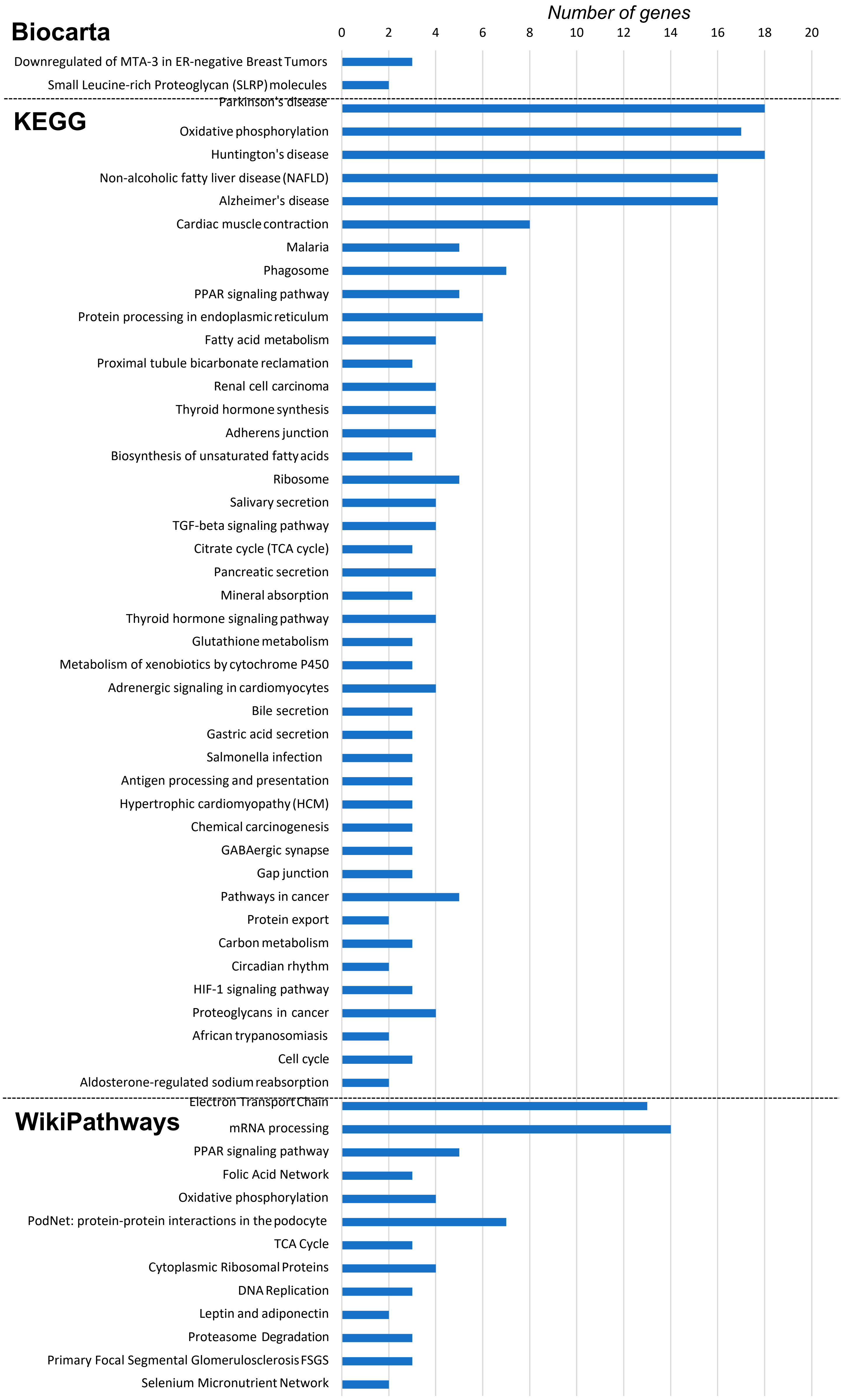

Supplemental Figure S3: Histogram of differentially expressed protein coding genes pathways in hippocampal microvessels for the high glycemic diet (HGD) when compared to the low glycemic diet (LGD). Significant cellular pathways ($p<0.05$) of differentially expressed protein coding genes in hippocampus microvessels from the high glycemic diet (HGD) compared to low glycemic diet (LGD). The data are shown for three biological replicates for each dietary group. Biocarta, KEGG and WikiPathways were identified using Genetrial2 online database.

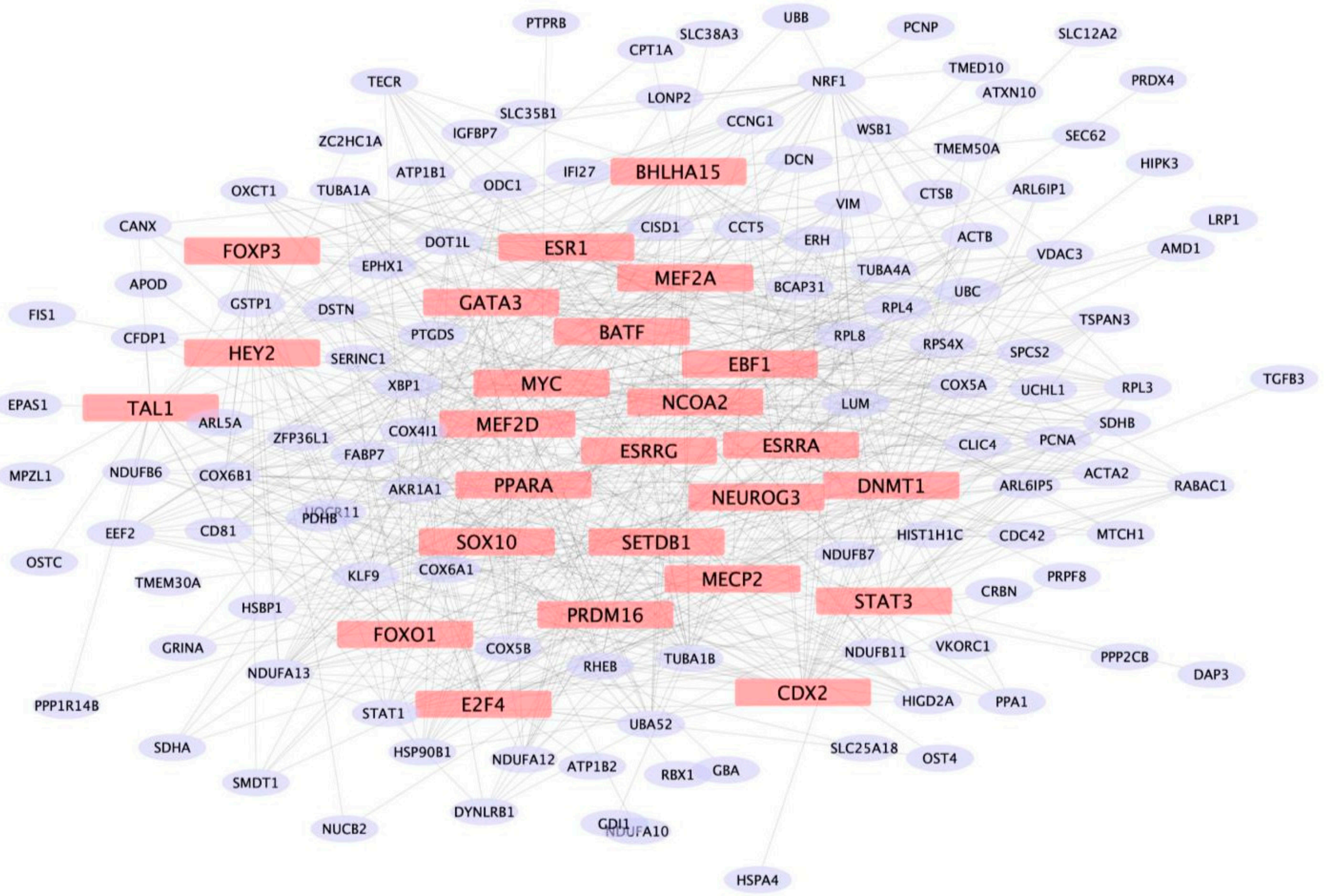

Supplemental Figure S4. Target gene networks of differentially expressed transcription factors (TFs) in hippocampal microvessels for the high glycemic diet (HGD) compared to the low glycemic diet (LGD). The network of interactions between differentially expressed transcription factors (TFs, pink) and their target genes (purple) of the high glycemic diet (HGD) compared to the low glycemic diet (LGD). The data are shown for three biological replicates for each dietary group. TFs and their targets were identified using Enrichr database.

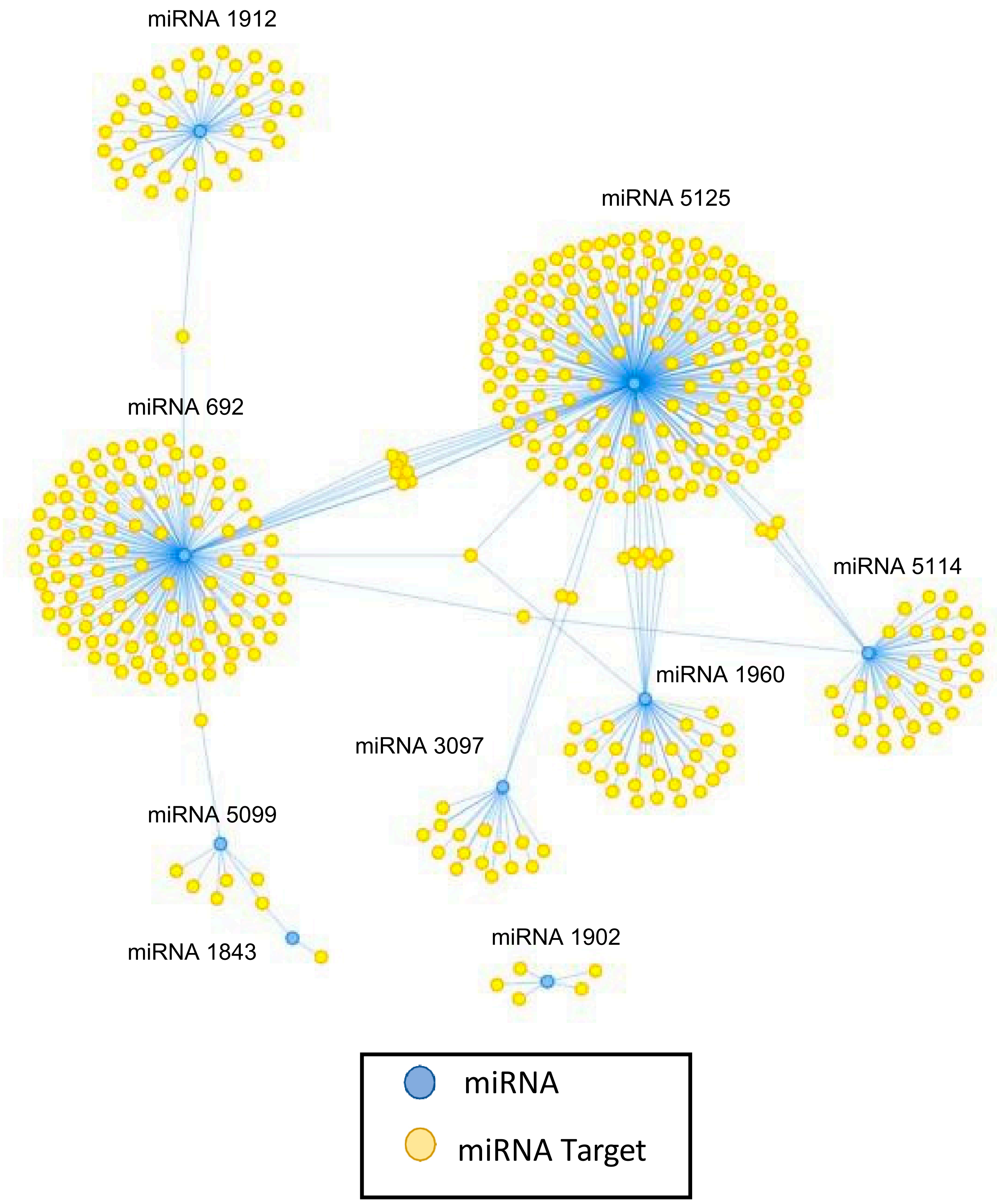

Supplemental Figure S5. Target gene networks of differentially expressed miRNAs in hippocampal microvessels with the high glycemic diet (HGD) compared to the low glycemic diet (LGD). The network of interactions between differentially expressed miRNAs (blue circles) and their target genes (yellow circles) of the high glycemic diet (HGD) compared to the low glycemic diet (LGD). The data are shown for three biological replicates for each dietary group. miRNA targets were identified using Mienturnet database.

Supplemental Figure S6

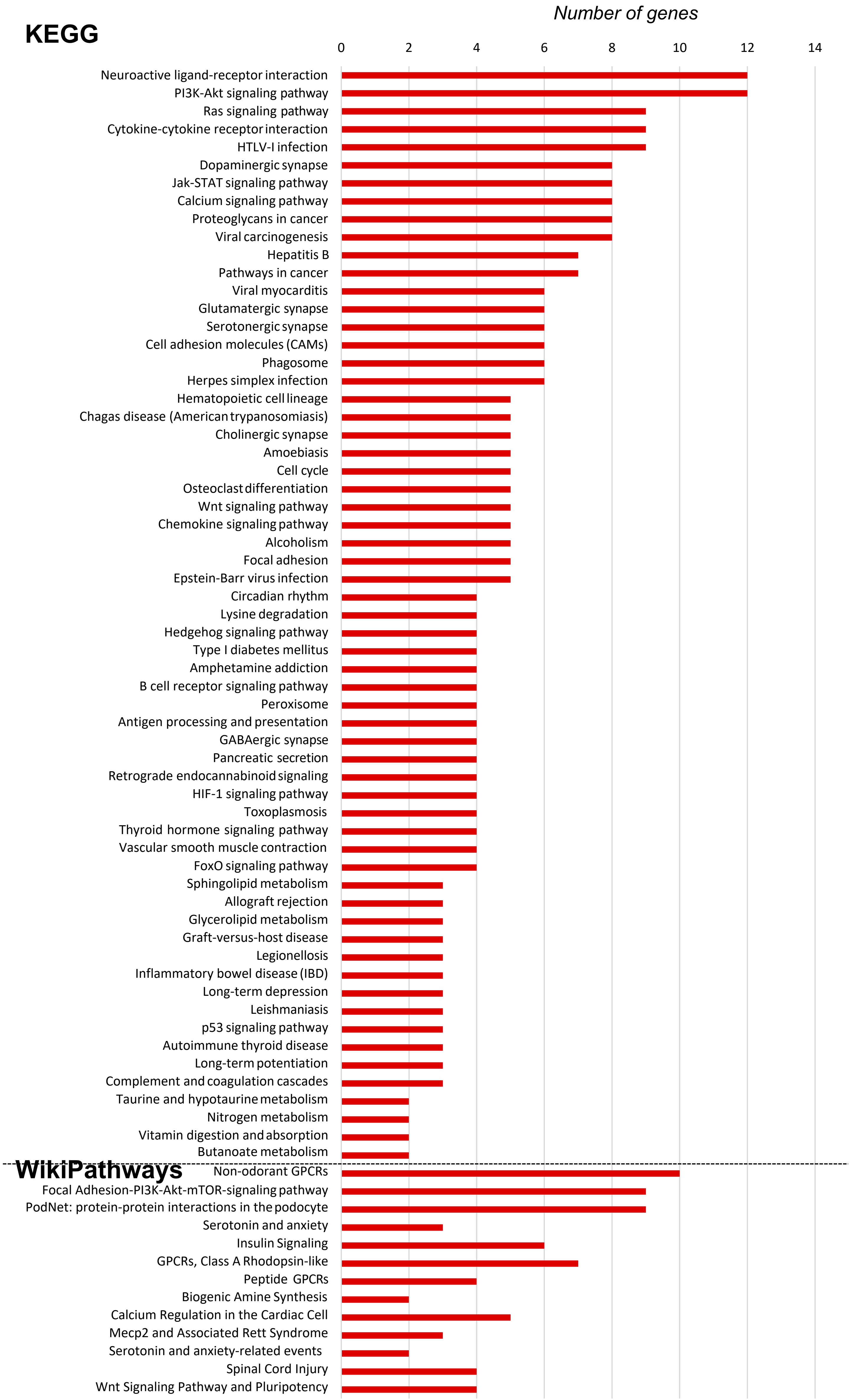

Supplemental Figure S6: Histogram of differentially expressed miRNA targets pathways in hippocampal microvessels with the high glycemic diet (HGD) when compared to the low glycemic diet (LGD). Significant cellular pathways ($p<0.05$) of differentially expressed miRNA target genes in hippocampus microvessels from the high glycemic diet (HGD) compared to low glycemic diet (LGD). KEGG and WikiPathways were identified using Genetrial2 online database. The data are shown for three biological replicates for each dietary group.

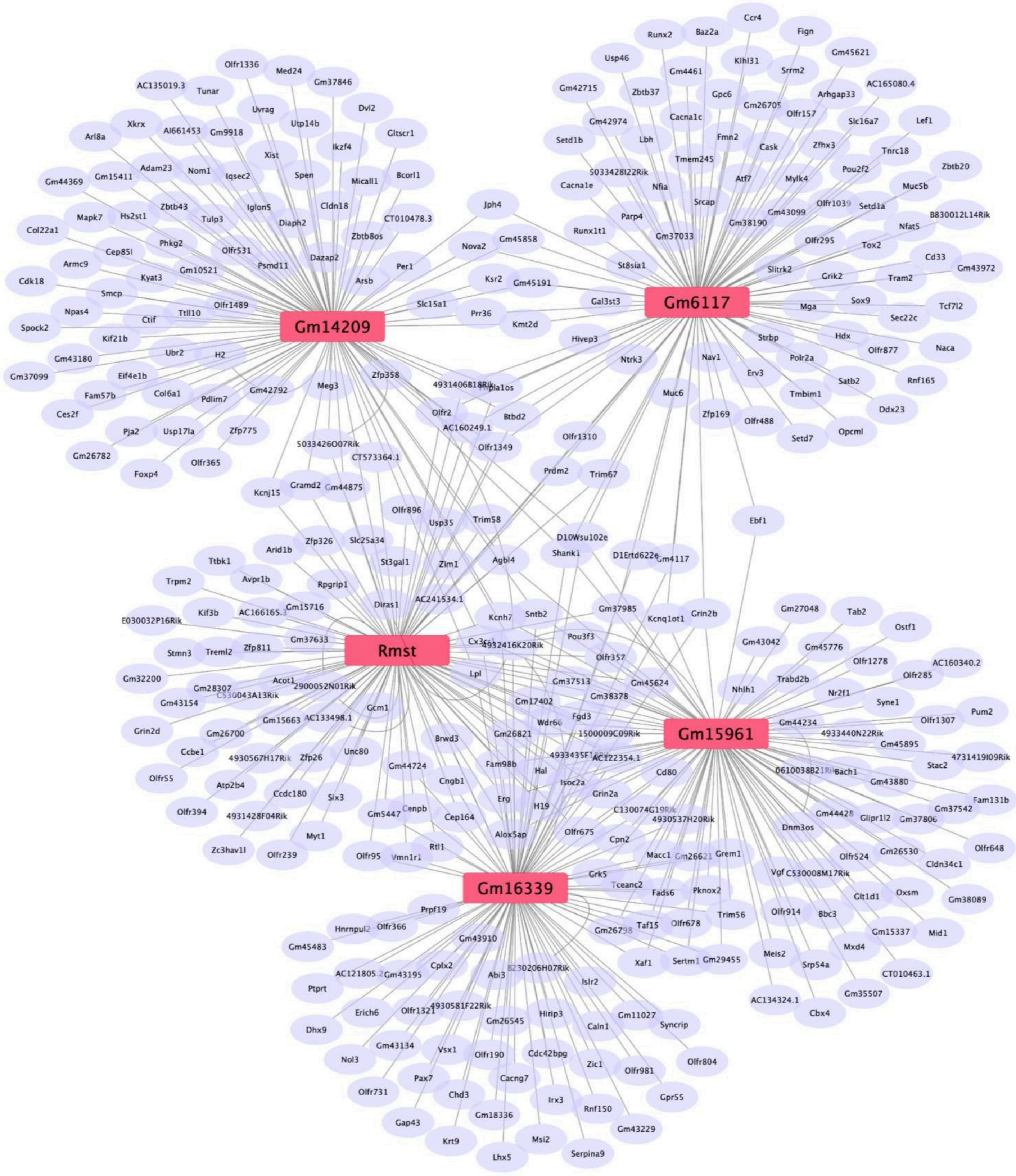

Supplemental Figure S7. Target gene networks of differentially expressed LncRNAs in hippocampal microvessels with the high glycemic diet (HGD) compared to the low glycemic diet (LGD). The network of interactions between differentially expressed lncRNAs (red boxes) and their target genes (purple circles) of the high glycemic diet (HGD) compared to the low glycemic diet (LGD). The data are shown for three biological replicates for each dietary group. LncRNA targets were identified using LncRRIsearch and Rtools CBRC databases.

Supplemental Figure S8

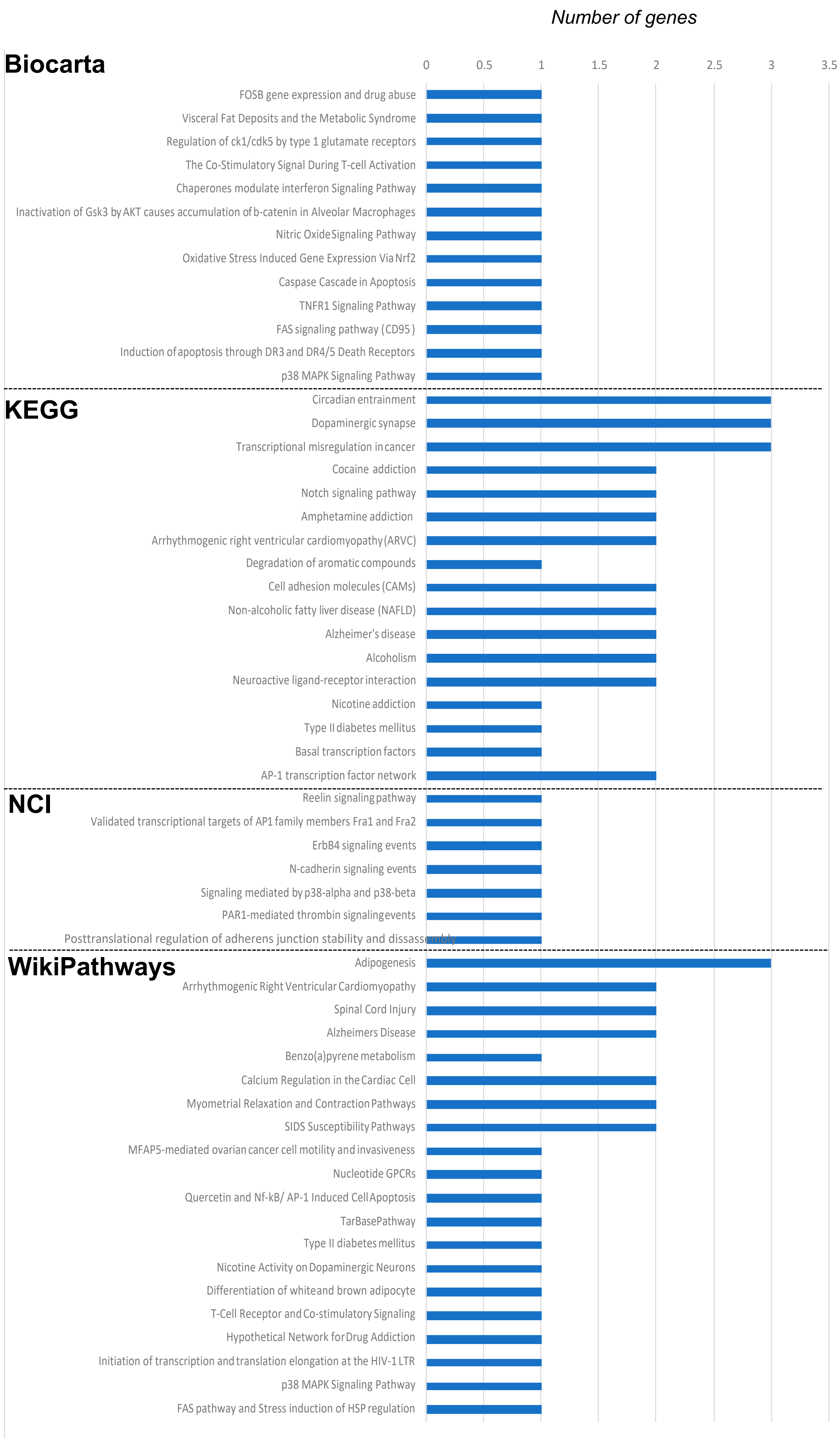

Supplemental Figure S8: Histogram of differentially expressed lncRNA targets pathways in hippocampal microvessels with the high glycemic diet (HGD) when compared to the low glycemic diet (LGD). Significant cellular pathways ($p<0.05$) of differentially expressed lncRNA target genes in hippocampus microvessels from the high glycemic diet (HGD) compared to low glycemic diet (LGD). The data are shown for three biological replicates for each dietary group. Biocarta, KEGG, NCI and WikiPathways were identified using Genetrial2 online database.

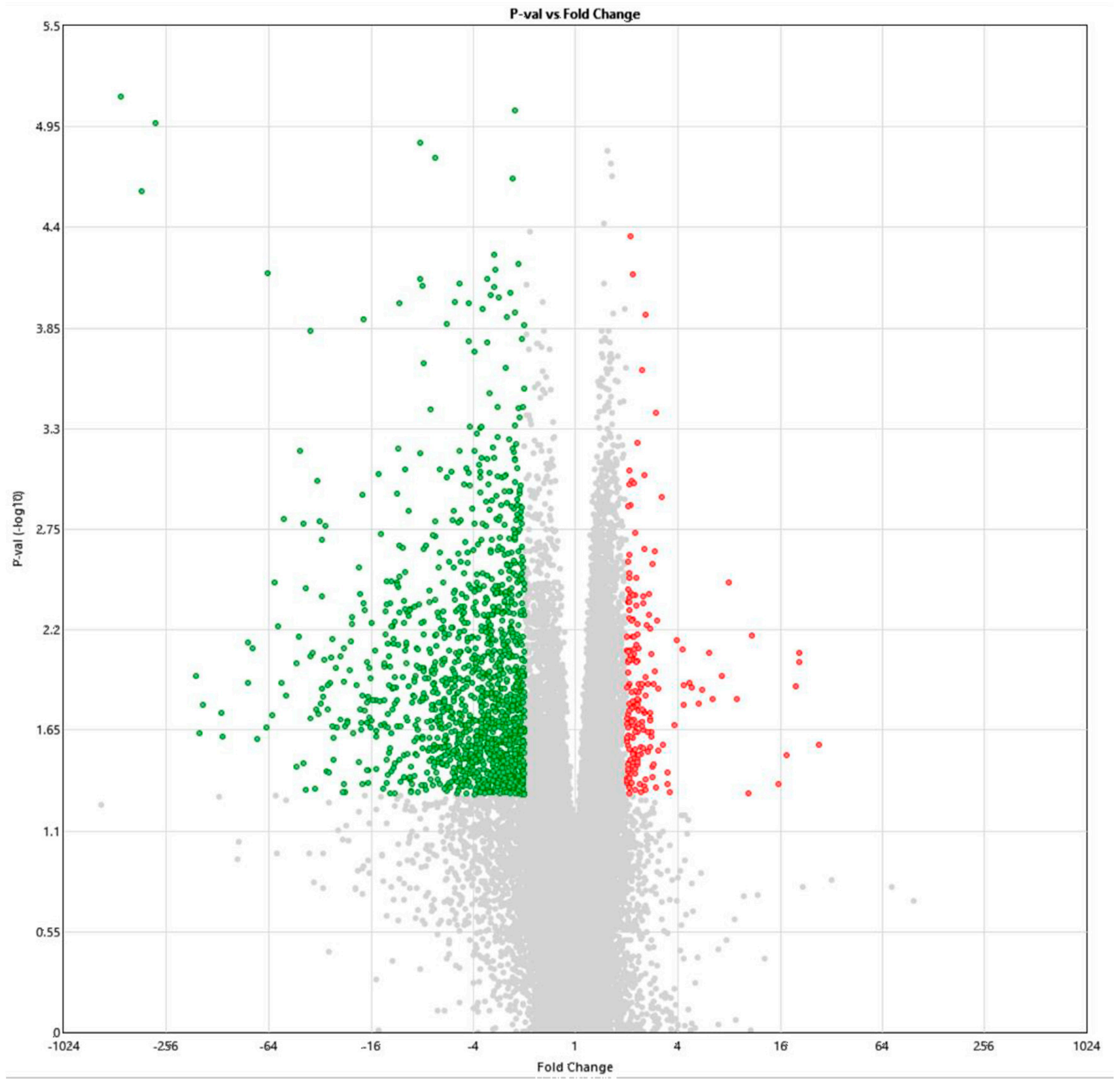

Supplement Figure S9: Volcano plot of differential gene expression changes in hippocampal microvessels for the high glycemic diet (HGD) with soluble epoxide hydrolase inhibitor (sEHI) compared to without sEHI treatment. The transcriptome of hippocampus microvessels from the high glycemic diet (HGD) with soluble epoxide hydrolase inhibitor (sEHI) compared to without sEHI treatment. The data are shown for three biological replicates for each dietary group. The x-axis specifies the fold-changes (FC), and the y-axis specifies the negative logarithm to the base 10 of the p-values. Filtering criteria for differentially expressed genes (DEGs) is FC = ±2.0 and p-value < 0.05. Red and green dots represent probe sets for transcripts expressed at significantly higher or lower levels than the HGD without sEHI treatment, respectively.

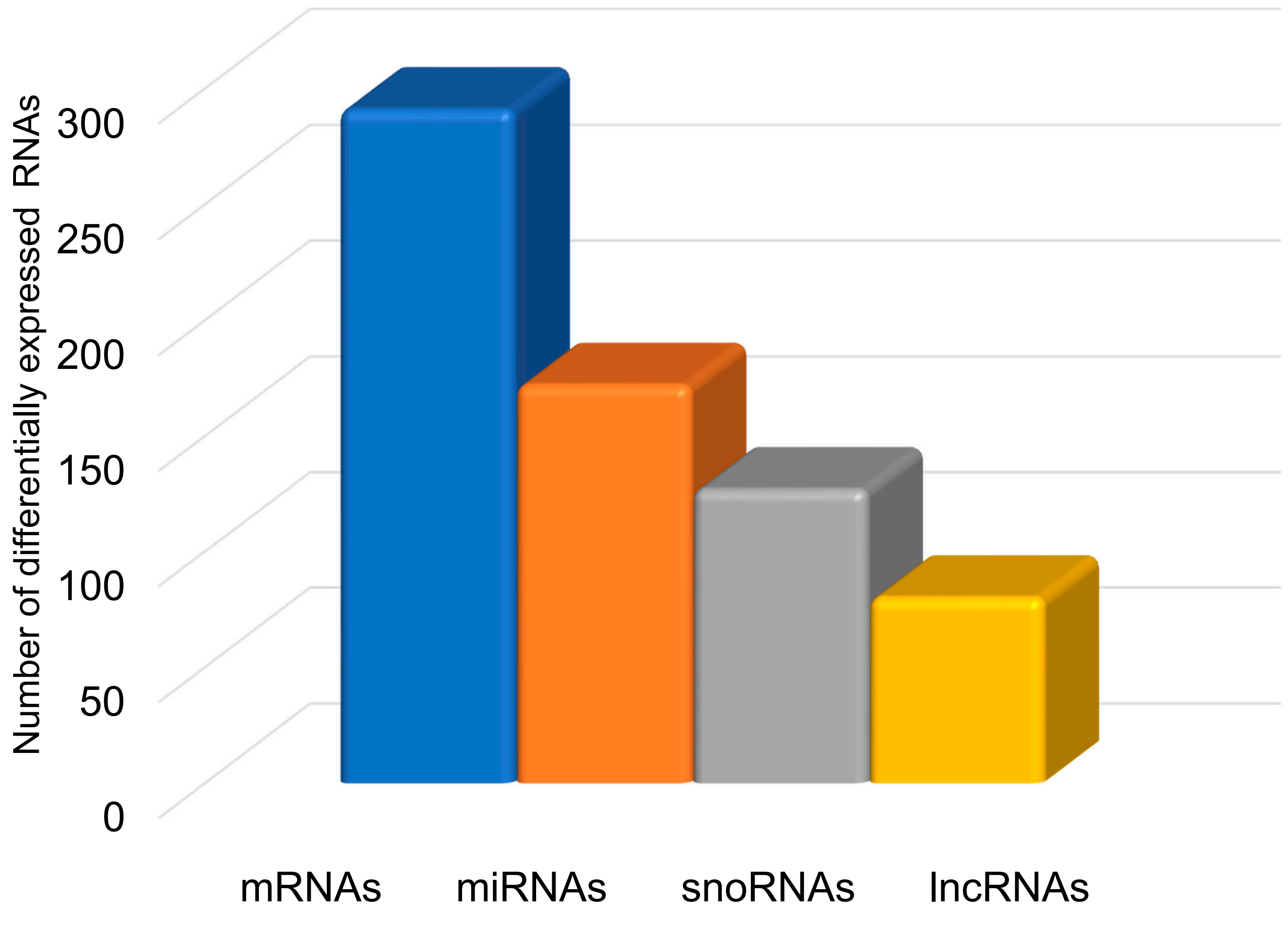

Supplemental Figure S10: Distribution of differentially expressed RNAs in hippocampal microvessels for the high glycemic diet (HGD) with soluble epoxide hydrolase inhibitor (sEHI) compared to without sEHI treatment. Number of differentially expressed protein coding (messenger RNAs=mRNAs, blue) and non-protein coding RNAs (microRNAs=miRNAs, orange; small nucleolar RNA=snoRNAs, grey; and long non-coding RNA=lncRNAs, yellow) in hippocampal microvessels from the high glycemic diet (HGD) with soluble epoxide hydrolase inhibitor (sEHI) compared to without sEHI treatment. The data are shown for three biological replicates for each dietary group.

Supplemental Figure S11

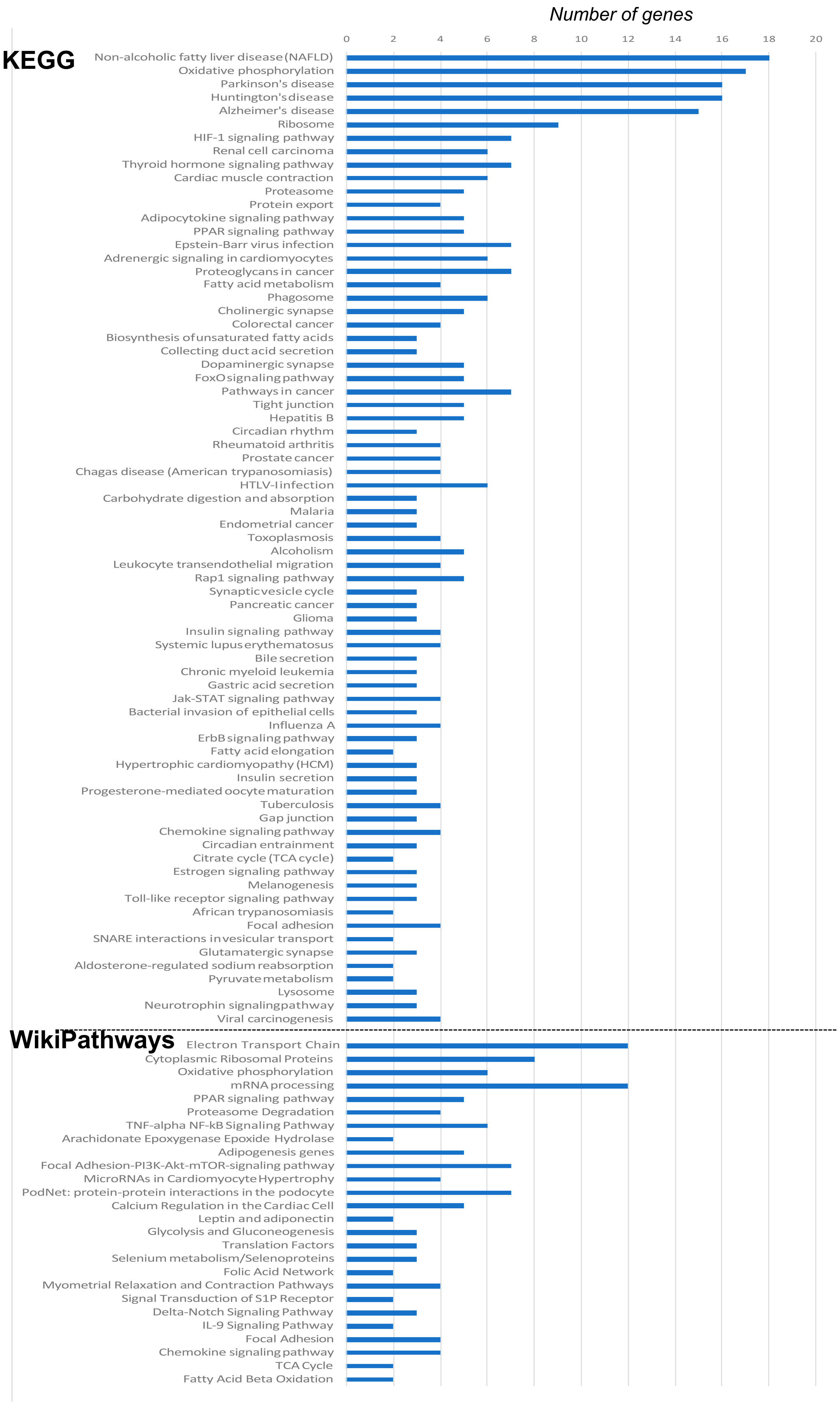

Supplemental Figure S11: Histogram of protein coding differentially expressed genes pathways in hippocampal microvessels for the high glycemic diet (HGD) with soluble epoxide hydrolase inhibitor (sEHI) compared to without sEHI treatment. Significant cellular pathways ($p<0.05$) of differentially expressed protein coding genes in hippocampus microvessels from the high glycemic diet (HGD) with soluble epoxide hydrolase inhibitor (sEHI) compared to without sEHI treatment. The data are shown for three biological replicates for each dietary group. KEGG and WikiPathways were identified using Genetrial2 online database.

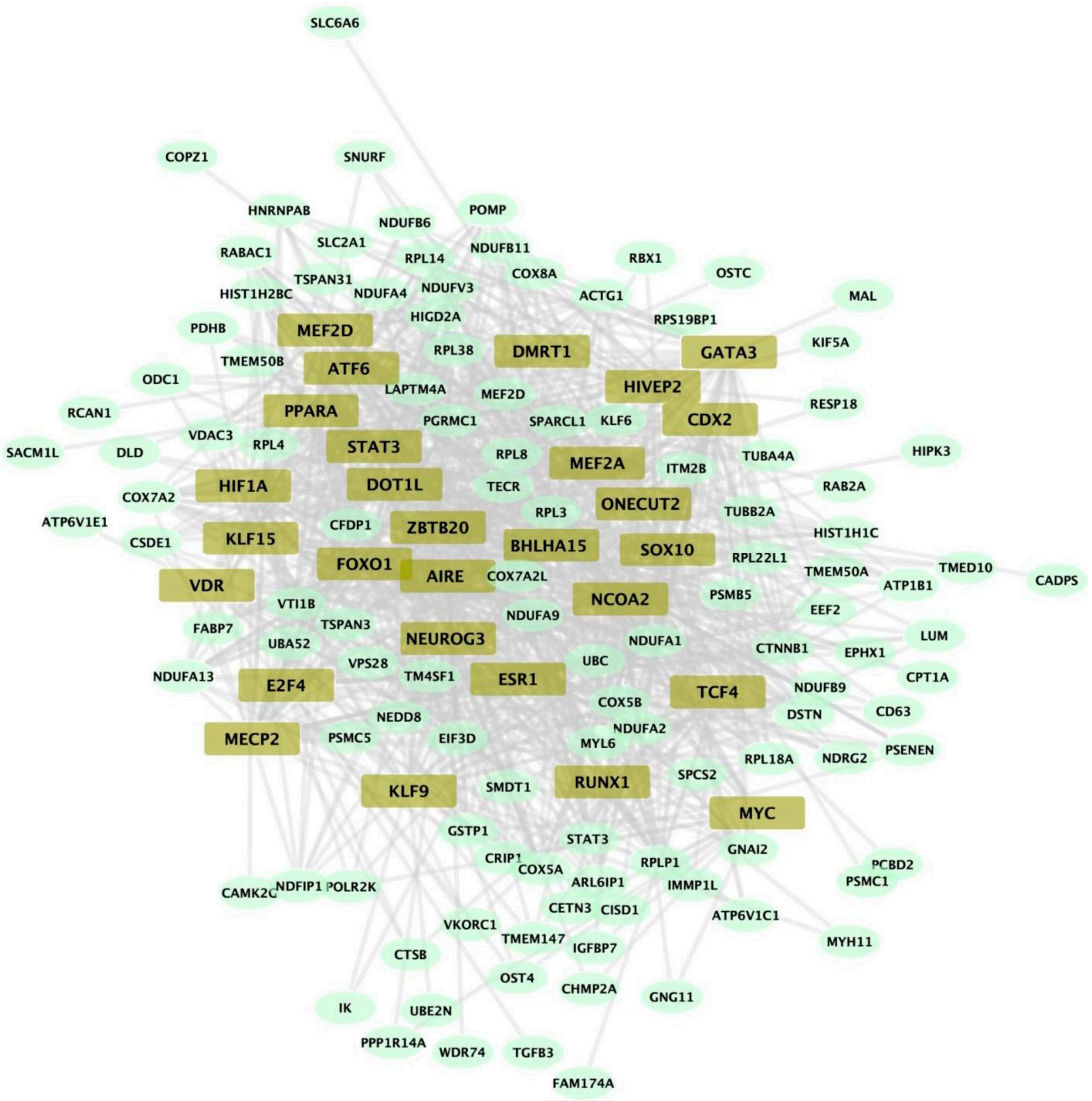

Supplemental Figure S12. Target gene networks of differentially expressed transcription factors (TFs) in hippocampal microvessels for the high glycemic diet (HGD) with and without soluble epoxide hydrolase inhibitor (sEHI). The network of interactions between differentially expressed transcription factors (TFs, yellow) and their target genes (green) of the high glycemic diet (HGD) with and without soluble epoxide hydrolase inhibitor (sEHI). The data are shown for three biological replicates for each dietary group. TFs and their targets were identified using Enrichr database.

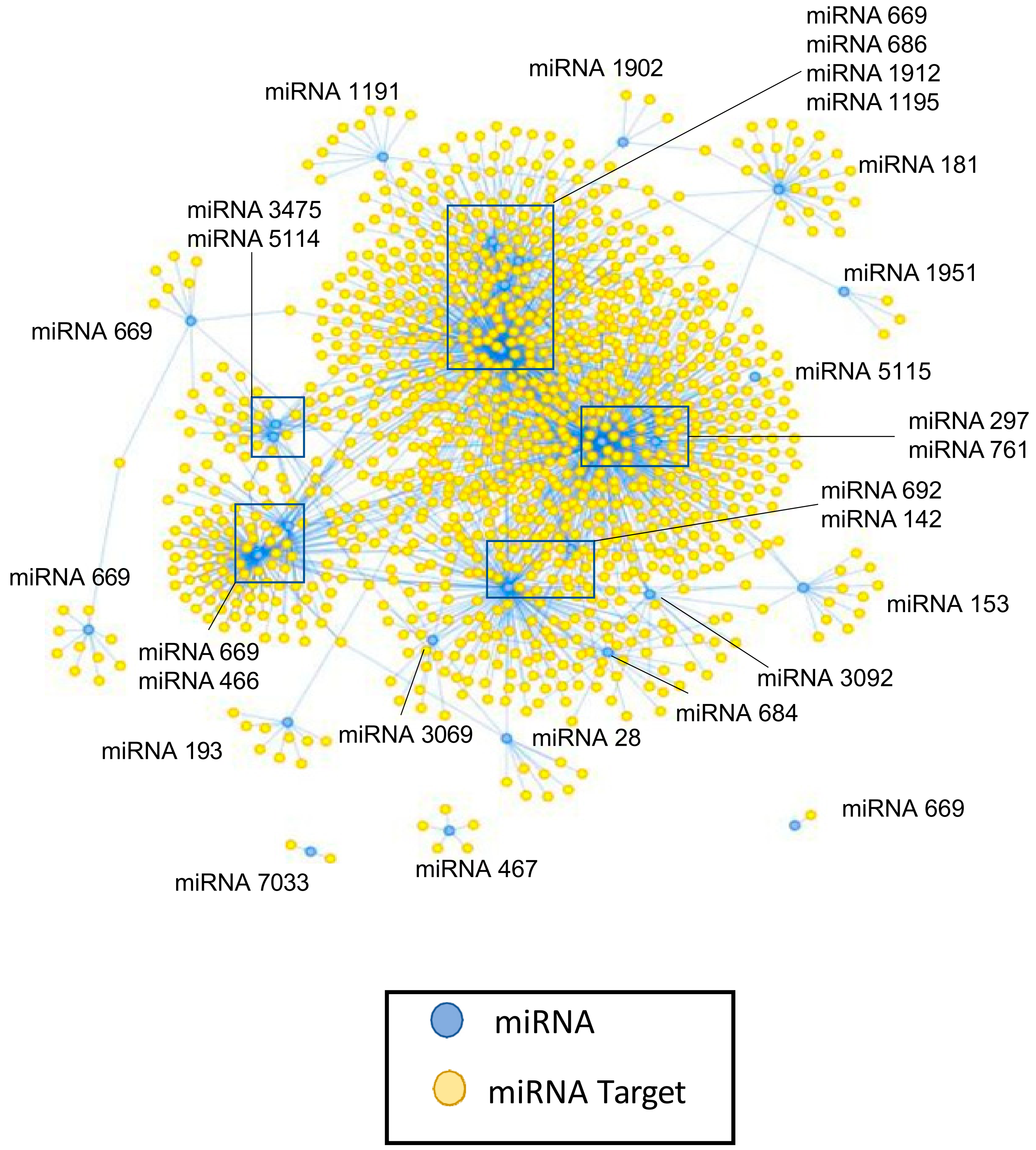

Supplemental Figure S13. Target gene networks of differentially expressed miRNAs in hippocampal microvessels for the high glycemic diet (HGD) with and without soluble epoxide hydrolase inhibitor (sEHI). The network of interactions between differentially expressed miRNAs (blue circles) and their target genes (yellow circles) of the high glycemic diet (HGD) with soluble epoxide hydrolase inhibitor (sEHI) when compared to HGD without inhibitor. The data are shown for three biological replicates for each dietary group. miRNA targets were identified using Mienturnet database.

Supplemental Figure S14

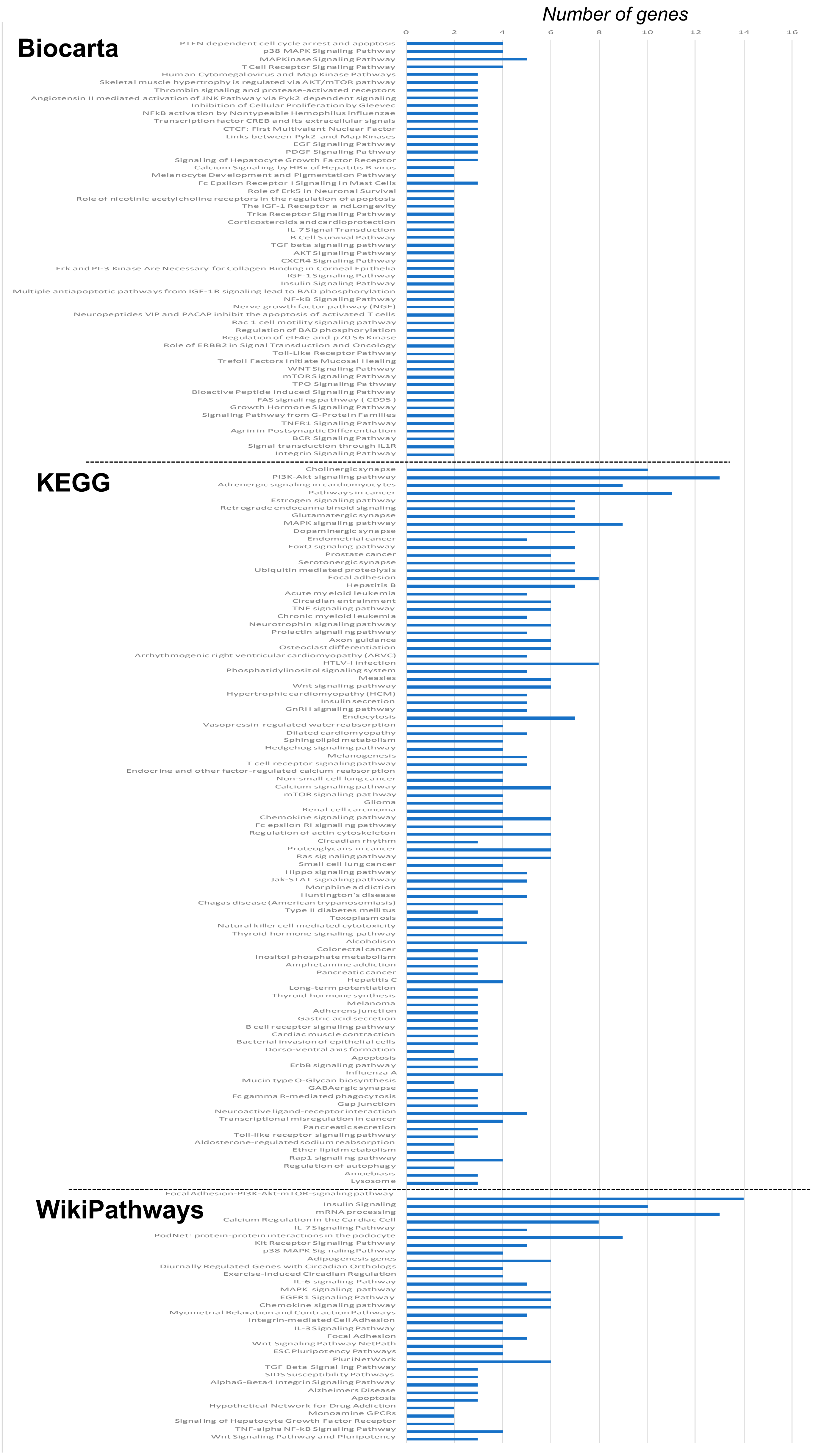

Supplemental Figure S14: Histogram of differentially expressed miRNA targets pathways in hippocampal microvessels for the high glycemic diet (HGD) with soluble epoxide hydrolase inhibitor (sEHI) compared to without sEHI treatment. Significant cellular pathways ($p<0.05$) of differentially expressed miRNA target genes in hippocampus microvessels of the high glycemic diet (HGD) with soluble epoxide hydrolase inhibitor (sEHI) compared to without sEHI treatment. The data are shown for three biological replicates for each dietary group. Biocarta, KEGG and WikiPathways were identified using Genetrial2 online database.

Supplemental Figure S15

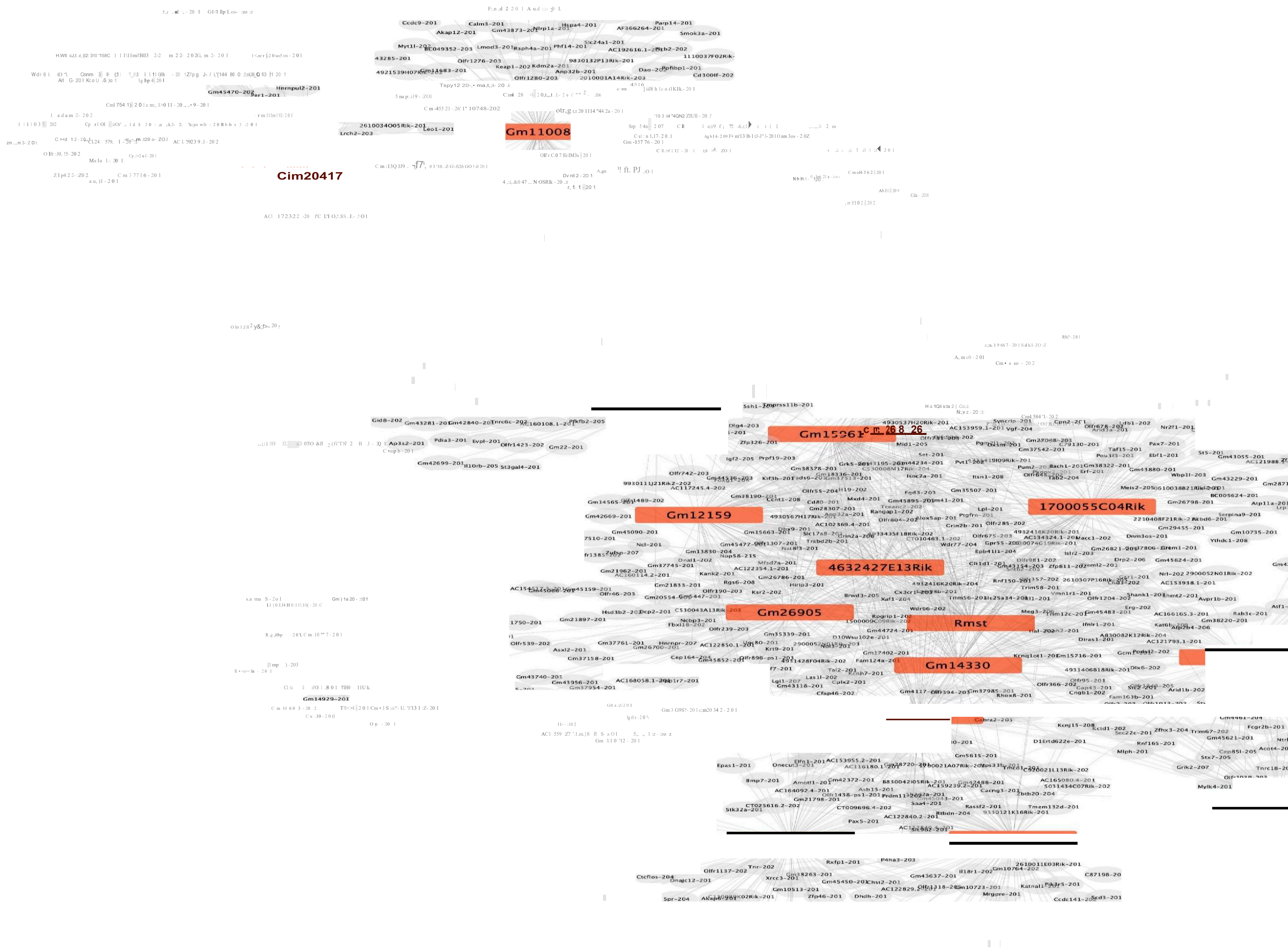

Supplemental Figure S15. Target gene networks of differentially expressed LncRNAs in hippocampal microvessels for the high glycemic diet (HGD) with and without soluble epoxide hydrolase inhibitor (sEHI). The network of interactions between differentially expressed lncRNAs (red boxes) and their target genes (purple circles) of the high glycemic diet (HGD) with soluble epoxide hydrolase inhibitor (sEHI) when compared to HGD without inhibitor. The data are shown for three biological replicates for each dietary group. LncRNA targets were identified using LncRRIsearch and Rtools CBRC databases.

Supplemental Figure S16

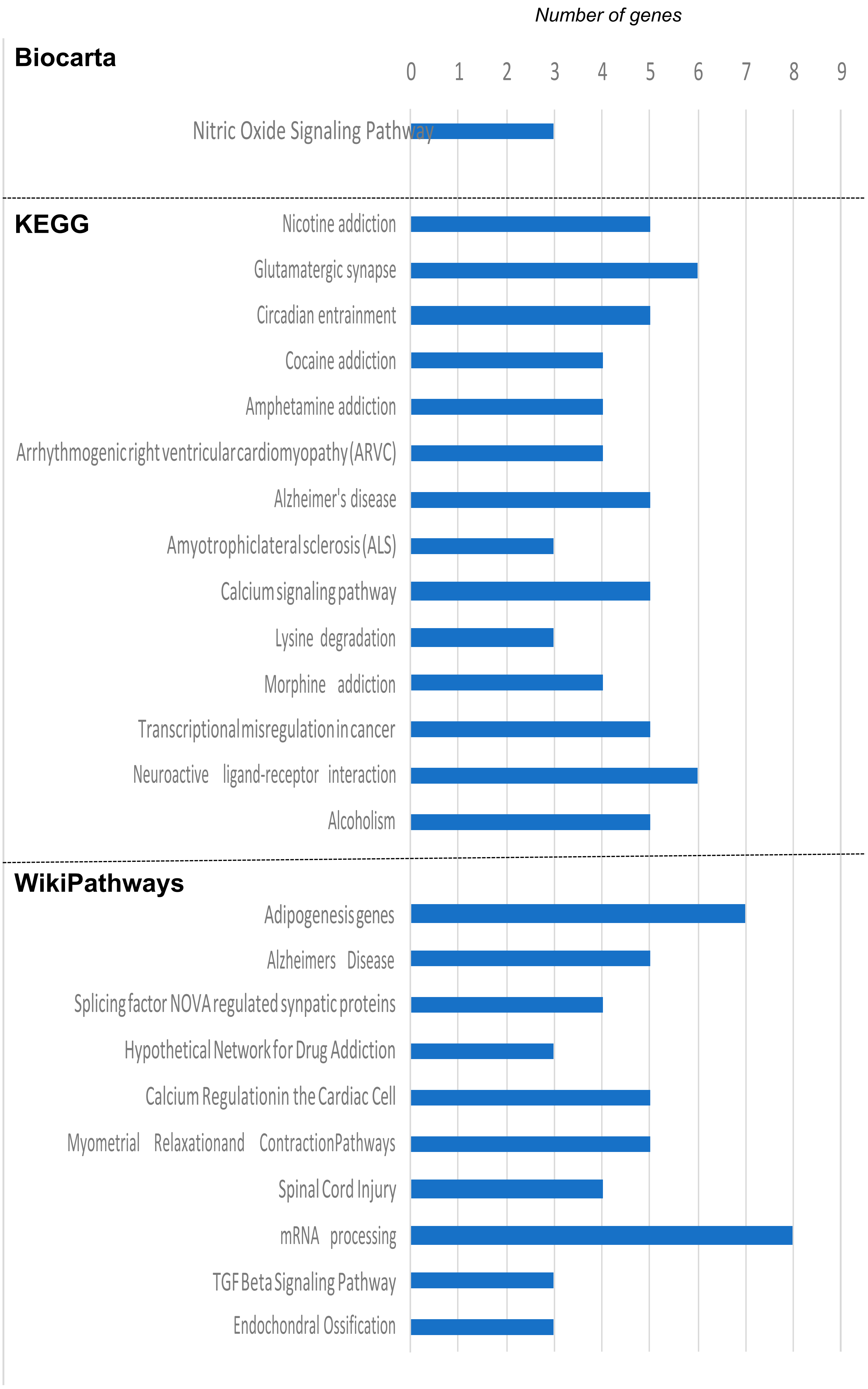

Supplemental Figure S16: Histogram of differentially expressed lncRNA targets pathways in hippocampal microvessels for the high glycemic diet (HGD) with soluble epoxide hydrolase inhibitor (sEHI) compared to without sEHI treatment. Significant cellular pathways ($p<0.05$) of differentially expressed lncRNA target genes in hippocampus microvessels of the high glycemic diet (HGD) with soluble epoxide hydrolase inhibitor (sEHI) compared to without sEHI treatment. The data are shown for three biological replicates for each dietary group. Biocarta, KEGG and WikiPathways were identified using Genetrial2 online database.

Table S1: Differentially expressed genes for the high glycemic diet (HGD) compared to low glycemic diet (LGD).

Gene Symbol	Description	Fold Change
Ppp1r14b	protein phosphatase 1, regulatory (inhibitor) subunit 1	2.21
Gm6117; RP23-192D5.	predicted gene 6117 [Source:MGI Symbol;Acc:MGI:	-2.02
		3.73
Fam205a2; Gm10600	family with sequence similarity 205, member A2; pre	2.02
Gm24400	predicted gene, 24400 [Source:MGI Symbol;Acc:MG	19.44
Gm19738	PREDICTED: predicted gene, 19738, transcript varia	3.65
Gm7381	predicted gene 7381 [Source:MGI Symbol;Acc:MGI:	8.66
Dnajc4	DnaJ (Hsp40) homolog, subfamily C, member 4	2.03
Gm7497	predicted gene 7497 [Source:MGI Symbol;Acc:MGI:	3.35
Gm19767	PREDICTED: predicted gene, 19767 (Gm19767), mi	2.69
Gm24783	predicted gene, 24783 [Source:MGI Symbol;Acc:MG	-2.5
Brms1l	breast cancer metastasis-suppressor 1-like	2.87
Jag1	jagged 1	2.28
Snord16a	small nucleolar RNA, C/D box 16A	4.28
Gm23098	predicted gene, 23098	-16.65
Gm11037	predicted gene 11037 [Source:MGI Symbol;Acc:MGI	-2.66
		-2.6
		2.34
		2.55
		2.05
Gm16209	predicted gene 16209 [Source:MGI Symbol;Acc:MGI	4.3
Rab24	RAB24, member RAS oncogene family	2.36
Dctn2	dynactin 2	2.17
		-8.67
Gm4804	predicted gene 4804 [Source:MGI Symbol;Acc:MGI:	2.24
		4.09
Mir5125	microRNA 5125	-3.25
		-3.12
Atp2b4; Mir6903	ATPase, Ca++ transporting, plasma membrane 4; mic	2.15
Gm12428	predicted gene 12428 [Source:MGI Symbol;Acc:MGI	2.59
Dynlrb1	dynein light chain roadblock-type 1	2.52
Gm1866	predicted gene 1866 [Source:MGI Symbol;Acc:MGI:	2.97
		-2.49
		-2.47
Higd2a	HIG1 domain family, member 2A	12.28
Gm24621	predicted gene, 24621 [Source:MGI Symbol;Acc:MG	-2.3
Uck2	uridine-cytidine kinase 2	2.59
		-2.58
Eef2	eukaryotic translation elongation factor 2	3.37
		-2.21
Gm3608	predicted gene 3608 [Source:MGI Symbol;Acc:MGI:	3.14

		2.03
Gm22289	predicted gene, 22289 [Source:MGI Symbol;Acc:MG	11.37
Cd248	CD248 antigen, endosialin	2.23
Slc18b1	solute carrier family 18, subfamily B, member 1	2.33
Cox6a1	cytochrome c oxidase subunit VIa polypeptide 1	2.98
Hist1h2an	histone cluster 1, H2an	7.16
Ndufa10	NADH dehydrogenase (ubiquinone) 1 alpha subcomp	2.26
		2.51
Gba	glucosidase, beta, acid	2.2
		-2.61
Gm10171	predicted gene 10171	4.98
		2.95
		2.95
Hist2h4	histone cluster 2, H4	2.86
Gm12074	predicted gene 12074 [Source:MGI Symbol;Acc:MGI	2.39
Gm13050	predicted gene 13050 [Source:MGI Symbol;Acc:MGI	2.14
Gm17756	PREDICTED: predicted gene, 17756 (Gm17756), m	2.17
Cox5b	cytochrome c oxidase subunit Vb	2.99
LOC100861862	PREDICTED: uncharacterized LOC100861862 (LOC	3.16
Lonp2	lon peptidase 2, peroxisomal	2.27
		-2.51
LOC100861832	PREDICTED: uncharacterized LOC100861832 (LOC	2.77
		-2.47
		-2.1
Apoa1bp	apolipoprotein A-I binding protein	2.95
Plxnc1	plexin C1	2.03
Gm5612	predicted gene 5612 [Source:MGI Symbol;Acc:MGI:	2.53
Gm14292	predicted gene 14292 [Source:MGI Symbol;Acc:MGI	6.89
Epm2aip1	EPM2A (laforin) interacting protein 1	2.1
AY036118	cDNA sequence AY036118	2.15
Ranbp2-ps2	RAN binding protein 2, pseudogene 2	-2.01
Gm13132	predicted gene 13132 [Source:MGI Symbol;Acc:MGI	-3.6
		-3.15
13298; Fam205a4; Gm2	predicted gene 13298 (Gm13298), mRNA.; family wi	2.28
LOC100503279	PREDICTED: uncharacterized LOC100503279 (LOC	2.94
Hivep1	human immunodeficiency virus type I enhancer bindi	2.5
Ndufa13	NADH dehydrogenase (ubiquinone) 1 alpha subcomp	5.6
Rngtt	RNA guanylyltransferase and 5-phosphatase; RNA gu	2.35
Gm19974	PREDICTED: predicted gene, 19974 (Gm19974), mi	2.81
Slc38a3	solute carrier family 38, member 3	2.04
		-2.02
Stk39	serine/threonine kinase 39	2.28
Slc35b1	solute carrier family 35, member B1	2.3
Hist1h2aj	histone cluster 1, H2aj	6.89

Gm1974	PREDICTED: predicted gene 1974 (Gm1974), misc R	2.03
Gm11367	predicted gene 11367 [Source:MGI Symbol;Acc:MGI	3.49
Gm12816	predicted gene 12816	2.08
		9.98
Mir6928	microRNA 6928	-2.29
Mir5099	microRNA 5099	-2.11
Mir5099	microRNA 5099	-2.11
LOC100861650	PREDICTED: uncharacterized LOC100861650, trans	3.47
Lrp1	low density lipoprotein receptor-related protein 1	2.02
Prpf8	pre-mRNA processing factor 8	3.51
Gatad1	GATA zinc finger domain containing 1	2.35
Gm13862	predicted gene 13862 [Source:MGI Symbol;Acc:MGI	2.48
Crry-ps	complement receptor related protein, pseudogene [So	2.43
Gm7204	predicted pseudogene 7204 [Source:MGI Symbol;Acc	2.05
Ppa1	pyrophosphatase (inorganic) 1	2.28
Vim	vimentin	2.32
Gm9625	predicted gene 9625 [Source:MGI Symbol;Acc:MGI:	2.75
		-3.06
Cdc40	cell division cycle 40	2.07
		2.99
Pcna-ps2	proliferating cell nuclear antigen pseudogene 2 [Sourc	2.55
Zfp36l1	zinc finger protein 36, C3H type-like 1	2.04
Gm10233	predicted pseudogene 10233 [Source:MGI Symbol;Ac	5.79
Ankmy2	ankyrin repeat and MYND domain containing 2	2.16
Gm26361	predicted gene, 26361 [Source:MGI Symbol;Acc:MG	-2.07
		9.39
		3.08
Eif5al3-ps	eukaryotic translation initiation factor 5A-like 3, pseu	3.15
Hist1h2af	histone cluster 1, H2af	3.84
Scp2-ps2	sterol carrier protein 2, pseudogene 2 [Source:MGI Sy	2.35
Mlc1	megalencephalic leukoencephalopathy with subcortic	2.23
Gm2174	predicted gene 2174 [Source:MGI Symbol;Acc:MGI:	2.64
Gm22930	predicted gene, 22930	2.61
Gstp1	glutathione S-transferase, pi 1	2.52
		-2.17
Dap3	death associated protein 3	2.05
Wdr74	WD repeat domain 74	2.23
Cox5a	cytochrome c oxidase subunit Va	6.85
LOC100862246	PREDICTED: uncharacterized LOC100862246 (LOC	3.73
Pnmal1	PNMA-like 1	2.14
Gm6444	predicted gene 6444 [Source:MGI Symbol;Acc:MGI:	4.41
Gm27313	predicted gene, 27313 [Source:MGI Symbol;Acc:MG	-2.01
Oxct1	3-oxoacid CoA transferase 1	2.13
m15724; RP23-246B24	predicted gene 15724; novel pseudogene	-2.74

Gm11675	predicted gene 11675 [Source:MGI Symbol;Acc:MGI	3.26
Gm15530	predicted gene 15530 [Source:MGI Symbol;Acc:MGI	-4.2
Ndufb7	NADH dehydrogenase (ubiquinone) 1 beta subcomple	2.75
Nlk	nemo like kinase	2.04
Peg3os	Peg3 opposite strand	2.02
Ndufb6	NADH dehydrogenase (ubiquinone) 1 beta subcomple	3.27
		2.12
Pcna	proliferating cell nuclear antigen	2.46
		-2.85
Snapc1	small nuclear RNA activating complex, polypeptide 1	2.1
		4.55
Gm8927	predicted gene 8927 [Source:MGI Symbol;Acc:MGI:	2.09
Nucb2	nucleobindin 2	2.21
2900011O08Rik	RIKEN cDNA 2900011O08 gene	2.59
Mir8095	microRNA 8095	-3.67
Ighv5-12-4	Ighv5-12-4 immunoglobulin heavy variable 5-12-4	-2.5
		6.25
Rheb	Ras homolog enriched in brain	2.15
Gabra3	gamma-aminobutyric acid (GABA) A receptor, subun	2.45
Gm7286	predicted gene 7286 [Source:MGI Symbol;Acc:MGI:	3.1
Gm14049	predicted gene 14049 [Source:MGI Symbol;Acc:MGI	-2.2
		-2.11
Vkorc1	vitamin K epoxide reductase complex, subunit 1	8.9
Cisd1	CDGSH iron sulfur domain 1	2.64
Gmps	guanine monophosphate synthetase; guanine monphos	2.09
Slc12a2	solute carrier family 12, member 2	2.48
Sepw1	selenoprotein W, muscle 1	3.2
Gm13573	predicted gene 13573 [Source:MGI Symbol;Acc:MGI	3.37
Slc25a18	solute carrier family 25 (mitochondrial carrier), memb	2.4
		3.25
		-3.26
Rabac1	Rab acceptor 1 (prenylated)	4.41
		-5.06
Ranbp2-ps8	RAN binding protein 2, pseudogene 8 [Source:MGI S	-2.23
		4.61
Gabbr2	gamma-aminobutyric acid (GABA) B receptor, 2; Syn	2.14
Sdhb	succinate dehydrogenase complex, subunit B, iron sul	2.18
		2.22
Snord14e	small nucleolar RNA, C/D box 14E	16.58
Rpl18-ps2	ribosomal protein L18, pseudogene 2; 60S ribosomal	5.33
Ost4	oligosaccharyltransferase 4 homolog (S. cerevisiae)	2.43
Uchl1	ubiquitin carboxy-terminal hydrolase L1	2.89
Clic4	chloride intracellular channel 4 (mitochondrial)	2.36
Gm11511	predicted gene 11511 [Source:MGI Symbol;Acc:MGI	3.21

LOC100862216	PREDICTED: uncharacterized LOC100862216, trans	2.63
Gm12481	predicted gene 12481	3.45
Gstp2	glutathione S-transferase, pi 2	2.24
Acta2	actin, alpha 2, smooth muscle, aorta	3.33
LOC100862063	PREDICTED: uncharacterized LOC100862063 (LOC	2.23
		-5.55
Resp18	regulated endocrine-specific protein 18	8.51
		-2.77
		2.96
Gm13396	predicted gene 13396 [Source:MGI Symbol;Acc:MGI	-2.21
Cd81	CD81 antigen	4.17
Gm17383	predicted gene, 17383 [Source:MGI Symbol;Acc:MG	11.34
Tgfb3	transforming growth factor, beta 3	2.17
Sdha	succinate dehydrogenase complex, subunit A, flavopr	2.3
Tuba1b	tubulin, alpha 1B	3.38
		2.66
Atp1b2	ATPase, Na+/K+ transporting, beta 2 polypeptide	2.67
Gm10273	predicted pseudogene 10273 [Source:MGI Symbol;Ac	4.31
		2.08
		-2.07
Gm25732	predicted gene, 25732 [Source:MGI Symbol;Acc:MG	2.01
LOC100862384	PREDICTED: uncharacterized LOC100862384 (LOC	2.52
Abhd3	abhydrolase domain containing 3	2.26
Tsn	translin	2.64
		-2.01
Dcn	decorin	6.26
		-2.47
Atp5b	ATP synthase, H+ transporting mitochondrial F1 com	3
		4.29
Atp5l2-ps	ATP synthase, H+ transporting, mitochondrial FO co	5.26
Gm19496	PREDICTED: predicted gene, 19496 (Gm19496), mi	5.34
		-3.29
Ctsb	cathepsin B	2.44
Kif5a	kinesin family member 5A	3.39
		10.62
Gm10051	predicted pseudogene 10051	3.21
BC002163	NADH dehydrogenase Fe-S protein 5 pseudogene; cD	3.36
Cct5	chaperonin containing Tcp1, subunit 5 (epsilon)	2.33
Hipk3; Mir1902	homeodomain interacting protein kinase 3; microRNA	2.11
Nol4	nucleolar protein 4	2.11
Gm16238	predicted gene 16238 [Source:MGI Symbol;Acc:MGI	2.07
		-2.04
Rassf3	Ras association (RalGDS/AF-6) domain family memb	2.09
		-2.54

		2.66
Hist1h2al	histone cluster 1, H2al	2.66
Gm16378	predicted gene 16378 [Source:MGI Symbol;Acc:MGI	5.47
Syn1	synapsin I	2.02
Scd2; Mir5114	stearoyl-Coenzyme A desaturase 2; microRNA 5114	4.34
		11.08
Gm12254	predicted gene 12254 [Source:MGI Symbol;Acc:MGI	3.62
Spcs2	signal peptidase complex subunit 2 homolog (S. cerev	4.57
Clk3	CDC-like kinase 3	2.63
Rpsa-ps12	ribosomal protein SA, pseudogene 12	3.4
Gm14150	predicted gene 14150	4.14
Gm25581	predicted gene, 25581 [Source:MGI Symbol;Acc:MG	-2.59
Mir5125	microRNA 5125	-5.81
Tmem30a	transmembrane protein 30A	3.47
Babam1	BRISC and BRCA1 A complex member 1	2.94
Gm5931	predicted pseudogene 5931 [Source:MGI Symbol;Acc	3.2
Lancl1	LanC (bacterial lantibiotic synthetase component C)-l	2.21
Gm12460	predicted gene 12460 [Source:MGI Symbol;Acc:MGI	2.17
Lin7a	lin-7 homolog A (C. elegans)	2.33
m14209; RP23-464H11	predicted gene 14209 [Source:MGI Symbol;Acc:MGI	-2.23
Gm12967	predicted gene 12967 [Source:MGI Symbol;Acc:MGI	2.04
Gm14388	predicted gene 14388 [Source:MGI Symbol;Acc:MGI	2.7
Gm25153	predicted gene, 25153 [Source:MGI Symbol;Acc:MG	-2.23
Hist1h1c	histone cluster 1, H1c	3.39
		-2.11
Tmed10	transmembrane emp24-like trafficking protein 10 (yea	3.49
Gm19494	PREDICTED: predicted gene, 19494, transcript varia	5.09
		-2.32
		2.03
Rpl28-ps1	ribosomal protein L28, pseudogene 1	2.82
Gm17541	predicted gene, 17541 [Source:MGI Symbol;Acc:MG	6.03
Rps4x	ribosomal protein S4, X-linked; ribosomal protein S4,	10.22
Gm19475	PREDICTED: predicted gene, 19475 (Gm19475), m	2.8
Gm15920	predicted gene 15920 [Source:MGI Symbol;Acc:MGI	12.12
LOC100861805	PREDICTED: uncharacterized LOC100861805 (LOC	2.4
Gm16339; RP24-369B4.	predicted gene 16339 [Source:MGI Symbol;Acc:MGI	-2.49
Gm20775	predicted gene, 20775 [Source:MGI Symbol;Acc:MG	2.42
		-3.33
Gm9294	predicted pseudogene 9294	2.83
Sgpp2	sphingosine-1-phosphate phosphotase 2	2.24
Gm11273	predicted gene 11273	3.51
Gm24590	predicted gene, 24590 [Source:MGI Symbol;Acc:MG	2.59
		-3.53
Rmst	rhabdomyosarcoma 2 associated transcript (non-codin	2.24

		6.22
		3.4
Gm12618	predicted gene 12618	3.27
Pcdh9	protocadherin 9; Synthetic construct Mus musculus cl	3.16
Trappc11	trafficking protein particle complex 11	2.34
Rpl4	ribosomal protein L4	14.13
Gm6055	predicted gene 6055	2.49
		-2.45
		-2.41
m3893; 4933409K07Ri	predicted gene 3893; RIKEN cDNA 4933409K07 gen	2.11
Crbn	cereblon; cereblon (Crbn), transcript variant 2, mRNA	2.12
Mir7053	microRNA 7053	-2.31
		3.92
		-3.87
Zc2hc1a	zinc finger, C2HC-type containing 1A	2.33
		-2.45
LOC100862313	PREDICTED: uncharacterized LOC100862313, trans	2.43
Mpzl1	myelin protein zero-like 1	2.31
		3
Msl2	male-specific lethal 2 homolog (Drosophila); male-sp	2.3
Gm14176	predicted gene 14176	2.08
Amd2; Amd1	S-adenosylmethionine decarboxylase 2; S-adenosylme	2.43
Vamp1	vesicle-associated membrane protein 1	2.08
Arl6ip5	ADP-ribosylation factor-like 6 interacting protein 5	2.08
Rpl28-ps3	ribosomal protein L28, pseudogene 3	3.77
Ifi27	interferon, alpha-inducible protein 27	2.13
Gm5514	predicted gene 5514 [Source:MGI Symbol;Acc:MGI:	3.67
Mir6412	microRNA 6412	3.01
		2.45
		-2.1
Gm15665	predicted gene 15665 [Source:MGI Symbol;Acc:MGI	-5.62
Ubc; Uba52	ubiquitin C; ubiquitin A-52 residue ribosomal protein	5.5
Sec62	SEC62 homolog (S. cerevisiae)	2.07
Gm14165	predicted gene 14165 [Source:MGI Symbol;Acc:MGI	2.26
Gm24588	predicted gene, 24588 [Source:MGI Symbol;Acc:MG	2.82
Gm10221	predicted gene 10221 [Source:MGI Symbol;Acc:MGI	3.34
Cnot4	CCR4-NOT transcription complex, subunit 4	2.21
Pts	6-pyruvoyl-tetrahydropterin synthase	3.23
LOC100862094	PREDICTED: uncharacterized LOC100862094 (LOC	3.28
Mir3097	microRNA 3097	2.66
		3.17
Scarna3a; Mir1843b	small Cajal body-specific RNA 3A; microRNA 1843b	-2.7
		3.43
		-2.28

Gm23448	predicted gene, 23448 [Source:MGI Symbol;Acc:MG	-2.23
		-2.1
Rps2-ps13	ribosomal protein S2, pseudogene 13	4.21
Gdi1	guanosine diphosphate (GDP) dissociation inhibitor 1	3.45
Gm25635	predicted gene, 25635 [Source:MGI Symbol;Acc:MG	4.17
Gm23510	predicted gene, 23510 [Source:MGI Symbol;Acc:MG	-3.07
Gm6822	predicted pseudogene 6822	3.03
		-2.15
Rpl19-ps12	ribosomal protein L19, pseudogene 12	3.27
Actb	actin, beta	2.62
Gm16399	predicted pseudogene 16399 [Source:MGI Symbol;Ac	2.89
		-2.26
Ptgds	prostaglandin D2 synthase (brain)	2.15
Rab7	RAB7, member RAS oncogene family	6.38
Gm5436	predicted pseudogene 5436 [Source:MGI Symbol;Acc	2.22
Tmem50a	transmembrane protein 50A	5.35
		2.16
LOC100861642	PREDICTED: uncharacterized LOC100861642 (LOC	4.22
Gm5265	predicted pseudogene 5265 [Source:MGI Symbol;Acc	3.76
Gm20432	predicted gene 20432 [Source:MGI Symbol;Acc:MGI	2
Gm15961; RP23-455J6.	predicted gene 15961 [Source:MGI Symbol;Acc:MGI	2.58
		-7.83
Traj59	T cell receptor alpha joining 59	-3.01
		-4.66
Rbx1	ring-box 1	2.89
Gm17428	predicted gene, 17428 [Source:MGI Symbol;Acc:MG	-5.48
		2.52
		-2.93
Mir1912	microRNA 1912	25.94
Gm10181	predicted gene 10181 [Source:MGI Symbol;Acc:MGI	-5.52
Ppp2cb	protein phosphatase 2 (formerly 2A), catalytic subunit	2.72
Ephx1	epoxide hydrolase 1, microsomal	2.17
		-2.63
Wsb1	WD repeat and SOCS box-containing 1	2.48
		-2.89
Gm16354	predicted gene 16354 [Source:MGI Symbol;Acc:MGI	3.24
		5.16
		2.48
		2.57
Gm8648	predicted gene 8648 [Source:MGI Symbol;Acc:MGI:	5.06
Cfdp1	craniofacial development protein 1	2.11
Rpl3	ribosomal protein L3	6.09
Ube2nl	ubiquitin-conjugating enzyme E2N-like [Source:MGI	2.41
		-3.14

		2.71
		-4.46
Gm14604; RP23-260P9.	predicted gene 14604; novel pseudogene	-2.68
Rpl8	ribosomal protein L8	6.14
Bcap31	B cell receptor associated protein 31	2.32
		2.62
Cpt1a	carnitine palmitoyltransferase 1a, liver	2.24
Cds2	CDP-diacylglycerol synthase (phosphatidate cytidylyl	2.07
		-2.1
Mtmr6	myotubularin related protein 6	2.14
Gm25188	predicted gene, 25188 [Source:MGI Symbol;Acc:MG	11.15
Ubb; Gm1821	ubiquitin B; ubiquitin pseudogene	4.61
Prdx4	peroxiredoxin 4	2.9
Tuba1a	tubulin, alpha 1A	3.19
Gm7363	predicted gene 7363 [Source:MGI Symbol;Acc:MGI:	4.19
Mir692-1	microRNA 692-1	6.04
Gm17604	predicted gene, 17604 [Source:MGI Symbol;Acc:MG	-15.11
Gm10224	predicted pseudogene 10224	2.88
Gm4017; Gm10157	predicted gene 4017 [Source:MGI Symbol;Acc:MGI:	8.36
Akr1a1	aldo-keto reductase family 1, member A1 (aldehyde r	2.61
Gm16470	predicted pseudogene 16470 [Source:MGI Symbol;Ac	2.65
Gm5777	predicted gene 5777 [Source:MGI Symbol;Acc:MGI:	2.82
Gm9166	predicted gene 9166 [Source:MGI Symbol;Acc:MGI:	2.48
Raph1	Ras association (RalGDS/AF-6) and pleckstrin homol	2.24
		2.57
Gm1821	predicted gene 1821 (Gm1821), non-coding RNA.; ub	13.6
Arl5a	ADP-ribosylation factor-like 5A	2.16
		2.48
Cox4i1	cytochrome c oxidase subunit IV isoform 1	2.2
Fis1	fission 1 (mitochondrial outer membrane) homolog (y	2.11
Gm4479	predicted gene 4479 [Source:MGI Symbol;Acc:MGI:	3.79
Gm6293	predicted pseudogene 6293	3.27
Gm5471	predicted pseudogene 5471	5.07
Ndufb11	NADH dehydrogenase (ubiquinone) 1 beta subcomple	5.18
Tspan3	tetraspanin 3	3.97
Gm13249	predicted gene 13249	2.54
Gm22043	predicted gene, 22043 [Source:MGI Symbol;Acc:MG	-2.36
		4.44
Mir1960	microRNA 1960	-2.61
Gm19774	PREDICTED: predicted gene, 19774 (Gm19774), m	5.38
Gm15013	predicted gene 15013 [Source:MGI Symbol;Acc:MGI	3.32
		-5.96
Gm27626	predicted gene, 27626 [Source:MGI Symbol;Acc:MG	10.89
Mir6352	microRNA 6352	-3.3

Gm2531	predicted gene 2531 [Source:MGI Symbol;Acc:MGI:	-3.78
Gm25559	predicted gene, 25559 [Source:MGI Symbol;Acc:MG	-3.19
2410015M20Rik	RIKEN cDNA 2410015M20 gene	2.33
		2.59
Cops3	COP9 (constitutive photomorphogenic) homolog, sub	2.46
Apod	apolipoprotein D	7.04
Rpl10-ps6	ribosomal protein L10, pseudogene 6	6.03
		-4.65
Rbm4	RNA binding motif protein 4	2.09
Hspa4	heat shock protein 4	2.08
Rpl32-ps	ribosomal protein L32, pseudogene [Source:MGI Sym	2.27
		-3.04
Gm6428	predicted pseudogene 6428 [Source:MGI Symbol;Acc	3.71
Gm27248	predicted gene 27248 [Source:MGI Symbol;Acc:MGI	2.21
Gm6374	predicted gene 6374	2.18
LOC100862193	PREDICTED: uncharacterized LOC100862193 (LOC	2.26
Ptprb	protein tyrosine phosphatase, receptor type, B	2.33
Rgs7bp	regulator of G-protein signalling 7 binding protein	2.47
Gm13680	predicted gene 13680 [Source:MGI Symbol;Acc:MGI	11.94
LOC100862107	PREDICTED: uncharacterized LOC100862107 (LOC	2.4
Gm4987	predicted gene 4987 [Source:MGI Symbol;Acc:MGI:	2.01
Gm12231	predicted gene 12231	7.02
Atp1b1	ATPase, Na+/K+ transporting, beta 1 polypeptide	3.3
		2.91
Tuba4a	tubulin, alpha 4A	2.76
Vdac3	voltage-dependent anion channel 3	8.21
Canx	calnexin	2.22
Gm11628	predicted gene 11628 [Source:MGI Symbol;Acc:MGI	2.62
Prkag1	protein kinase, AMP-activated, gamma 1 non-catalyti	3.84
		2.7
Maf	avian musculoaponeurotic fibrosarcoma (v-maf) AS4	2.17
Gm8508	predicted gene 8508 [Source:MGI Symbol;Acc:MGI:	3.07
Gm14670	predicted gene 14670 [Source:MGI Symbol;Acc:MGI	-2.44
		-2.64
Gm11539	predicted gene 11539 [Source:MGI Symbol;Acc:MGI	3.02
Grina	glutamate receptor, ionotropic, N-methyl D-aspartate-	5.12
Gm3695	predicted gene 3695 [Source:MGI Symbol;Acc:MGI:	2.4
Gm22501	predicted gene, 22501 [Source:MGI Symbol;Acc:MG	2.36
Gm19491	PREDICTED: predicted gene, 19491 (Gm19491), mi	2.55
		2.09
Rpl9-ps1	ribosomal protein L9, pseudogene 1	2.68
Odc1	ornithine decarboxylase, structural 1	4.17
		-2.73
Rpl10-ps1	ribosomal protein L10, pseudogene 1	6.3

Gm13436	predicted gene 13436	2.41
Gm7502	predicted gene 7502 [Source:MGI Symbol;Acc:MGI:	2.25
Gm24983	predicted gene, 24983 [Source:MGI Symbol;Acc:MG	-3.41
		3.6
Gm12328	predicted gene 12328 [Source:MGI Symbol;Acc:MGI	6.46
		2.1
		2.1
Gm22760; Gm12318	predicted gene, 22760 [Source:MGI Symbol;Acc:MG	14.29
Gm22358	predicted gene, 22358 [Source:MGI Symbol;Acc:MG	7.39
Epas1	endothelial PAS domain protein 1	3.75
		-4.53
Derl1	Der1-like domain family, member 1	2.32
Gm12726	predicted gene 12726 [Source:MGI Symbol;Acc:MGI	2.47
		2.56
Gm25594	predicted gene, 25594 [Source:MGI Symbol;Acc:MG	6.76
Rpl21-ps12	ribosomal protein L21, pseudogene 12 [Source:MGI S	3.07
		-2.63
Hba-a2; Hba-a1	hemoglobin alpha, adult chain 2; hemoglobin alpha, a	2.56
Gm4735	predicted gene 4735	6.34
		-2.01
		-2.29
Gm13413	predicted gene 13413 [Source:MGI Symbol;Acc:MGI	3.16
Cox6b1	cytochrome c oxidase, subunit VIb polypeptide 1	2.47
		2.79
Larp4	La ribonucleoprotein domain family, member 4; La ri	3.56
D8Ertd738e	DNA segment, Chr 8, ERATO Doi 738, expressed	2.51
		2.17
		-12.34
		-12.34
		-12.34
		-12.34
		-12.34
Hsp90b1	heat shock protein 90, beta (Grp94), member 1	3.4
Sv2b	synaptic vesicle glycoprotein 2 b	2.3
m14734; RP23-272G10	predicted gene 14734; novel pseudogene	-2.62
Pcnp	PEST proteolytic signal containing nuclear protein	2.92
		2.08
Arl6ip1	ADP-ribosylation factor-like 6 interacting protein 1	5.76
		-3.03
		-2.01
Ostc	oligosaccharyltransferase complex subunit	4.77
Gm5863	predicted gene 5863 [Source:MGI Symbol;Acc:MGI:	2.17
Ccng1	cyclin G1	2.59
		2.65

Eif1-ps1	eukaryotic translation initiation factor 1, pseudogene 1	6.36
		2.72
Hsbp1	heat shock factor binding protein 1	7.13
		-2.09
Snord82	small nucleolar RNA, C/D box 82	-5.52
Gm13882	predicted gene 13882 [Source:MGI Symbol;Acc:MGI	2.16
Gm12424	predicted gene 12424 [Source:MGI Symbol;Acc:MGI	-2.7
		2.94
Ncam1	neural cell adhesion molecule 1	2.36
		-2.85
Gm4883	predicted gene 4883	3.73
		-4.1
Elf1	E74-like factor 1; E74-like factor 1 (Elf1), mRNA.	2.71
Gm6969	predicted pseudogene 6969 [Source:MGI Symbol;Acc	3.22
		-2.71
Atp6v0e	ATPase, H+ transporting, lysosomal V0 subunit E	3.46
		-2.35
Cdc42	cell division cycle 42	2.84
Gm6316; EG622339	predicted pseudogene 6316 [Source:MGI Symbol;Acc	2.05
Gm13797	predicted gene 13797 [Source:MGI Symbol;Acc:MGI	12.42
Dstn	destrin	3.17
Pgm2l1	phosphoglucomutase 2-like 1	2.18
Slc9a9	solute carrier family 9 (sodium/hydrogen exchanger),	2.46
Mir8115	microRNA 8115	-5.46
		-3.48
Pdhb	pyruvate dehydrogenase (lipoamide) beta	2.32
Gm6768	predicted gene 6768 [Source:MGI Symbol;Acc:MGI:	5.45
Gm12666	predicted gene 12666	2.88
Gm20349	PREDICTED: predicted gene, 20349 (Gm20349), mi	3.01
Gm16412	predicted pseudogene 16412 [Source:MGI Symbol;Ac	2.88
Ndufa12	NADH dehydrogenase (ubiquinone) 1 alpha subcomp	2.18
Abhd2	abhydrolase domain containing 2	2.13
Trf	transferrin	2.06
Gm12129	predicted gene 12129 [Source:MGI Symbol;Acc:MGI	-2.51
Lum	lumican	2.28
		2.64
LOC100861804	PREDICTED: uncharacterized LOC100861804, trans	2.17
		3.73
Gm12922	predicted gene 12922 [Source:MGI Symbol;Acc:MGI	2.23
		-2.04
Gm19976	PREDICTED: predicted gene, 19976, transcript varia	2.18
Rnaset2a; Rnaset2b	ribonuclease T2A; ribonuclease T2B (Rnaset2b), mR	3.68
Rpl18-ps1	ribosomal protein L18, pseudogene 1	17.43
Gm12944	predicted gene 12944 [Source:MGI Symbol;Acc:MGI	4.22

		-2.91
		-2.01
		-2.01
		-2.01
Gm16416	predicted gene 16416 [Source:MGI Symbol;Acc:MGI	4.56
		6.76
Dctn4	dynactin 4	2.14
Gm13226	predicted gene 13226 [Source:MGI Symbol;Acc:MGI	2.71
Tecr	trans-2,3-enoyl-CoA reductase	2.59
Gm2546	predicted gene 2546 [Source:MGI Symbol;Acc:MGI:	3.31
Gm5560	predicted pseudogene 5560	2.8
Atxn10	ataxin 10	2.33
		7.48
		-2.13
Vkorc1l1	vitamin K epoxide reductase complex, subunit 1-like	2.47
Gm5921	predicted gene 5921 [Source:MGI Symbol;Acc:MGI:	2.67
Mtch1	mitochondrial carrier homolog 1 (C. elegans)	2.08
Gm14284	predicted gene 14284 [Source:MGI Symbol;Acc:MGI	7.88
Gkn3	gastrokine 3	2.26
		-3.65
		-2.8
		2.12
Cep70	centrosomal protein 70	-2.05
		-3.3
Gm14148	predicted gene 14148 [Source:MGI Symbol;Acc:MGI	2.24
Gm7887	predicted gene 7887 [Source:MGI Symbol;Acc:MGI:	3.81
Gm25660	predicted gene, 25660 [Source:MGI Symbol;Acc:MG	-2.63
Gm17047	predicted gene 17047 [Source:MGI Symbol;Acc:MGI	-2.19
Uqcr11	ubiquinol-cytochrome c reductase, complex III subuni	2.04
		-8.75
Gm14946	predicted gene 14946 [Source:MGI Symbol;Acc:MGI	-2.19
Uck2	uridine-cytidine kinase 2	4.28
Rpl19-ps4; Gm27506	ribosomal protein L19, pseudogene 4 [Source:MGI Sy	3.46
Fads2	fatty acid desaturase 2	2.37
Gm6829	predicted pseudogene 6829 [Source:MGI Symbol;Acc	4.18
Erh	enhancer of rudimentary homolog (Drosophila)	2.89
Igfbp7	insulin-like growth factor binding protein 7	2.36
		4.07
		3.52
		-3.52
		2.14
Gm19933	PREDICTED: predicted gene, 19933, transcript varia	3.43
Dhx9	DEAH (Asp-Glu-Ala-His) box polypeptide 9	2.15
Smdt1	single-pass membrane protein with aspartate rich tail	4.44

Fabp7	fatty acid binding protein 7, brain	4
Gm22757; Gm13875	predicted gene, 22757 [Source:MGI Symbol;Acc:MG	6.55
Gm25856	predicted gene, 25856 [Source:MGI Symbol;Acc:MG	-10.37
Gm5621	predicted gene 5621	2.39
		-2.21
Gm22663	predicted gene, 22663 [Source:MGI Symbol;Acc:MG	-2.53
Gm11969	predicted gene 11969 [Source:MGI Symbol;Acc:MGI	2.56
Gm8724	predicted pseudogene 8724 [Source:MGI Symbol;Acc	3.1
		2.28
Gm8399; Gm25241	predicted gene 8399 [Source:MGI Symbol;Acc:MGI:	3.1
Gm26300; Gm11285	predicted gene, 26300 [Source:MGI Symbol;Acc:MG	4.8
Gapdh-ps14	glyceraldehyde-3-phosphate dehydrogenase, pseudoge	2.25
Gm22303	predicted gene, 22303 [Source:MGI Symbol;Acc:MG	3.64
		2.44
		3.21
Gm5559	predicted gene 5559	2.01
		-2.02
Gm27684	predicted gene, 27684 [Source:MGI Symbol;Acc:MG	6.12
Gm13433	predicted gene 13433 [Source:MGI Symbol;Acc:MGI	5.56
		10.53
Gm4575	predicted gene 4575 [Source:MGI Symbol;Acc:MGI:	2.56
Atp5l-ps1	ATP synthase, H+ transporting, mitochondrial F0 com	6.14
		-2.03
		-2.07
Gm10198	predicted gene 10198 [Source:MGI Symbol;Acc:MGI	-3.76
Gm11185	predicted gene 11185 [Source:MGI Symbol;Acc:MGI	2.18
		2.2
Gm16418	predicted pseudogene 16418 [Source:MGI Symbol;Ac	2.66
		9.96
		2.54
		3.86
Gm11172	predicted gene 11172 [Source:MGI Symbol;Acc:MGI	-3.63
Gm5844	predicted gene 5844 [Source:MGI Symbol;Acc:MGI:	2.96
Tdrd3	tudor domain containing 3	2.11
Rpl14-ps1	ribosomal protein L14, pseudogene 1 [Source:MGI Sy	3.03
Napb	N-ethylmaleimide sensitive fusion protein attachment	2.57
		7.81
Serinc1	serine incorporator 1	5.7
Gm10073	predicted pseudogene 10073 [Source:MGI Symbol;Ac	3.23

Table S2. Top 30 transcription factors (TFs) differentially expressed by a high glycemic diet (HGD) with and without soluble epoxide hydrolase inhibitor (sEHI) in hippocampal microvascular endothelium.

HGD vs LGD			HGD+sEHI vs HGD		
**Top 30 TFs**	**TF Name**	**P-value**	**Top 30 TFs**	**TF Name**	**P-value**
MEF2A	Myocyte Enhancer Factor 2A	1.2E-25	MEF2D	Myocyte Enhancer Factor 2D	5.7E-24
MEF2D	Myocyte Enhancer Factor 2D	1.9E-25	MECP2	Methyl-CpG Binding Protein 2	3.1E-23
MECP2	Methyl-CpG Binding Protein 2	2.6E-22	MEF2A	Myocyte Enhancer Factor 2A	4.5E-22
CDX2	Caudal-type homeobox 2	5.2E-19	GATA3	GATA Binding Protein 3	2.4E-19
GATA3	GATA Binding Protein 3	8.1E-19	CDX2	Caudal Type Homeobox 2	1.0E-18
NCOA2	Nuclear Receptor Coactivator 2	2.0E-16	TCF4	Transcription Factor 4	1.9E-17
PPARA	Peroxisome Proliferator Activated Receptor Alpha	1.4E-15	NEUROG3	Neurogenin 3	3.7E-17
ESRRG	Estrogen Related Receptor Gamma	3.8E-15	E2F4	E2F Transcription Factor 4	1.1E-16
SETDB1	SET Domain Bifurcated Histone Lysine Methyltransferase 1	1.6E-14	ZBTB20	Zinc Finger And BTB Domain Containing 20	3.6E-16
BHLHA15	Basic Helix-Loop-Helix Family Member A15	2.3E-14	FOXO1	Forkhead Box O1	1.6E-15
STAT3	Signal Transducer And Activator Of Transcription 3	3.0E-14	KLF9	Kruppel Like Factor 9	2.2E-15
BATF	Basic Leucine Zipper ATF-Like Transcription Factor	6.3E-14	PPARA	Peroxisome Proliferator Activated Receptor Alpha	9.5E-15
SOX10	SRY-Box Transcription Factor 10	7.1E-14	HIF1A	Hypoxia Inducible Factor 1 Subunit Alpha	3.2E-14
MYC	MYC Proto-Oncogene, BHLH Transcription Factor	1.7E-13	BHLHA15	Basic Helix-Loop-Helix Family Member A15	4.9E-14
DNMT1	DNA Methyltransferase 1	2.1E-13	VDR	Vitamin D Receptor	1.1E-13
ESR1	Estrogen Receptor 1	2.2E-13	ESR1	Estrogen Receptor 1	1.5E-13
NEUROG3	Neurogenin 3	3.1E-13	MYC	MYC Proto-Oncogene, BHLH Transcription Factor	2.2E-13
EBF1	EBF Transcription Factor 1	3.3E-13	DOT1L	DOT1 Like Histone	2.3E-13

				Lysine Methyltransferase	
E2F4	E2F Transcription Factor 4	4.5E-13	KLF15	Kruppel Like Factor 15	1.6E-12
HEY2	Hes Related Family BHLH Transcription Factor With YRPW Motif 2	4.5E-13	RUNX1	RUNX Family Transcription Factor 1	1.8E-12
FOXP3	Forkhead Box P3	5.2E-13	NCOA2	Nuclear Receptor Coactivator 2	1.9E-12
TAL1	TAL BHLH Transcription Factor 1, Erythroid Differentiation Factor	5.5E-13	SOX10	SRY-Box Transcription Factor 10	2.2E-12
PRDM16	PR/SET Domain 16	5.5E-13	HIVEP2	HIVEP Zinc Finger 2	3.4E-12
ESRRA	Estrogen Related Receptor Alpha	7.4E-13	STAT3	Signal Transducer And Activator Of Transcription 3	7.8E-12
FOXO1	Forkhead Box O1	8.1E-13	ONECUT2	One Cut Homeobox 2	8.4E-12
XBP1	X-Box Binding Protein 1	8.6E-13	MEF2D	Myocyte Enhancer Factor 2D	1.6E-11
DOT1L	DOT1 Like Histone Lysine Methyltransferase	8.8E-13	ATF6	Activating Transcription Factor 6	2.2E-11
STAT1	Signal Transducer And Activator Of Transcription 1	1.1E-12	DMRT1	Doublesex And Mab-3 Related Transcription Factor 1	2.4E-11
KLF9	Kruppel Like Factor 9	1.4E-12	STAT3	Signal Transducer And Activator Of Transcription 3	3.0E-11
NRF1	Nuclear Respiratory Factor 1	2.3E-12	AIRE	Autoimmune Regulator	3.3E-11

Table S3. Effect of the high glycemic diet (HGD) with and without soluble epoxide hydrolase inhibitor (sEHI) on the expression of microRNAs (miRNAs) in hippocampal microvessels.

HGD vs LGD		HGD+sEHI vs HGD					
Gene Symbol	Fold Change	Gene Symbol	Fold Change	Gene Symbol	Fold Change	Gene Symbol	Fold Change
Mir1912	25.94	Gm25911	-472.71	Gm23019	-10.24	Mir669b	-4.63
Mir1960	-2.61	Gm24245	-295.98	Gm23415	-4.54	Mir669e	-14.98
Mir3097	2.66	Gm24270	-295.98	Mir6917	2.32	Mir684-1	-172.48
Mir5099	-2.11	Gm25732	-6.68	Gm23882	-7.85	Mir684-1	-157.28
Mir5125	-3.25	Gm24187	-361.17	Mir7667	2.29	Mir684-1	-157.28
Mir6352	-3.3	Gm23388	-64.54	Gm23086	-3.3	Mir684-1	-157.28
Mir6412	3.01	Mir669b	-3.15	Gm22610	-23.14	Mir684-1	-157.28
Mir692-1	6.04	Mir6338	2.47	Gm27530	2.1	Mir684-1	-157.28
Mir6928	-2.29	Gm27626	-52.49	Gm24369	-2.61	Mir684-1	-23.22
Mir7053	-2.31	Mir6412	-4.87	Gm25063	-12.6	Mir684-1	-12.92
Mir8095	-3.67	Gm22735	-3.52	Gm25000	-12.27	Mir684-2	-122.24
Mir8115	-5.46	Gm22930	-2.81	Gm26050	-3.82	Mir692-2	-9.57
Gm22930	2.61	Gm22694	-2.51	Gm22232	-3.3	Mir6935	-4.69
Gm24588	2.82	Gm24093	-2.56	Gm25040	-8.71	Mir6903	-3.03
Gm24590	2.59	Gm24811	-6.44	Gm22243	-3.27	Mir7090	-2.54
Gm25559	-3.19	Mir692-1	-12.13	Mir466f-4	-8.96	Mir5131	-2.05
Gm25594	6.76	Gm23053	-4.23	Gm23368	-5.59	Mir1902	-2.02
Gm25732	2.01	Gm22800	-3.08	Gm24026	-5.18	Mir761	-2.76
Gm27313	-2.01	Gm23566	-7.29	Mir1191	-28.06	Mir686	-11.16
Gm27626	10.89	Gm23051	-3.34	Gm27722	4.66	Mir3069	-2.33
Mir6903	2.15	Gm23190	-8.94	Mir6989	-19.52	Mir5114	-5.72
Mir1902	2.11	Mir669c	2.7	Gm25738	-4.36	Mir1291	-9.51
Mir5125	-5.81	Gm23489	-3.41	Gm26308	2.42	Gm23992	-3.49
Mir1843b	-2.7	Gm23154	-20.66	Gm26250	-2.24	Mir6388	2.19
Mir5114	4.34	Mir7033	2.07	Gm22201	2.12	Gm27989	-4.61
		Mir3092	3	Gm25693	-3.26	Mir193b	2.04
		Mir669m-2	-3.26	Gm26053	-18.06	Mir153	3.46
		Mir1951	2.21	Gm23045	-3.02	Gm23164	-2.28
		Gm27529	-33.75	Gm23728	-13.47	Gm27910	-6.87
		Gm22030	-5.74	Gm27684	-14.95	Gm23189	-2.15

	Gm27459	-6.46	Gm27935	-5.91	Gm25708	-2.15
	Mir1195	-3.59	Gm26469	-6.14	Gm25959	-2.15
	Gm23487	-4.05	Mir7053	2.07	Gm24332	-2.15
	Gm24588	-2.83	Gm25591	-2.83	Gm23802	-2.15
	Gm25233	-2.18	Gm24710	-3.38	Gm22903	-2.15
	Gm25355	-2.25	Gm24135	-3.38	Gm25571	-2.15
	Gm23064	-2.25	Gm24586	-4.47	Gm23049	-2.15
	Gm23631	-3.23	Gm23798	-2.15	Gm24370	-2.15
	Gm25986	-2.06	Gm25070	-2.15	Gm24159	-2.15
	Mir297-1	-14.89	Gm22475	-2.15	Gm25299	-2.15
	Gm22852	-2.47	Gm23400	-2.15	Gm28019	-2.72
	Gm23876	-3.41	Mir7063	2.99	Gm22189	-2.63
	Gm22117	-2.51	Mir6916	2.06	Gm27839	-6.54
	Gm27731	-2.92	Gm24541	2.59	Mir6386	2.57
	Gm23785	-2.08	Mir1912	-23.61	Gm22230	-2.08
	Mirlet7a-2	2.76	Mir142	2.52	Gm24111	2.01
	Gm22802	-3.16	Mir28c	4.3	Gm23374	-16.71
	Mir3475	3.04	Mir297a-2	-12.2		
	Mir466j	-3.66	Mir467c	-2.87		
	Gm22712	-8.71	Mir5115	-5.97		
	Mir6993	2.35	Gm23700	-2.03		
	Gm26231	-4.45	Mir669a-4	-2.09		
	Gm22234	-4.45	Mir669a-5	-2.09		
	Gm27450	-2.31	Mir669a-6	-2.09		
	Gm23880	2.32	Mir669a-7	-2.09		
	Gm27704	-11.55	Mir669a-8	-2.09		
	Gm22990	-2.76	Mir669a-9	-2.09		
	Gm23215	-2.21	Mir669a-10	-2.09		
	Mir181d	2.83	Mir669a-11	-2.09		
	Gm26463	-2.72	Mir669a-12	-2.09		
	Gm24975	-2.72	Gm27341	-4.43		
	Gm25100	-2.72	Gm23989	-2.96		

Table S4. Effect of the high glycemic diet (HGD) with and without soluble epoxide hydrolase inhibitor (sEHI) on the expression of long non-coding RNAs (lncRNAs) in hippocampal microvessels.

HGD vs LGD		HGD+sEHI vs HGD					
Gene Symbol	Fold Change	Gene Symbol	Fold Change	Gene Symbol	Fold Change	Gene Symbol	Fold Change
Gm6117; RP23-192D5.2	-2.02	1110032A03Rik	-2.07	Gm24139	-2	LOC100862193	-2.7
Gm19738	3.65	1700020I14Rik	-2.43	Gm25722	2.18	LOC100862198	-2.02
Gm19767	2.69	1700055C04Rik	2.01	Gm26414	2.17	LOC100862216	-8
Gm23098	-16.65	4632427E13Rik	-2.41	Gm26581; RP23-58L22.8	2.19	LOC100862227	-4.74
LOC100861862	3.16	A130040M12Rik	-2.5	Gm26818	-3.34	LOC100862246	-4.48
LOC100861832	2.77	DLG2-AS1_2	2.41	Gm26826	2.28	LOC100862257	-3.9
LOC100503279	2.94	Gm11008; RP23-83O23.1	-5.89	Gm26904	-3.87	LOC100862313	-7.1
Gm19974	2.81	Gm12159	2.36	Gm26905	-6.93	LOC100862318	-3.27
Gm1974	2.03	Gm14330; AC124590.1	2.06	Gm9753	2.43	LOC100862384	-3.6
LOC100861650	3.47	Gm15961; RP23-455J6.4	-2.56	Gm9794	-9.05	Mir28c	4.3
LOC100862246	3.73	Gm19295	-5.86	LOC100503279	-5.4	Mir5115	-5.97
Peg3os	2.02	Gm19425	-6.31	LOC100861642	-7.61	Peg3os	-2.28
LOC100862216	2.63	Gm19453	-8.51	LOC100861650	-3.7	PRINS	2.23
LOC100862063	2.23	Gm19474	-3.56	LOC100861675	-2.37	Rmst	-2.13

LOC100862384	2.52	Gm19491	-4.3	LOC100861724	2.59	Rny3	-65.75
Gm19496	5.34	Gm19494	-11.17	LOC100861762	-2.26	Rpl14-ps1	-3.81
Gm14209; RP23-464H11.2	-2.23	Gm19496	-6.31	LOC100861804	-2.44		
Gm19494	5.09	Gm19595	-31.12	LOC100861805	-2.58		
LOC100861805	2.4	Gm19660	-4.58	LOC100861832	-2.64		
Gm16339; RP24-369B4.1	-2.49	Gm19738	-8.16	LOC100861833	-6.59		
Rmst	2.24	Gm19767	-3.32	LOC100861852	-2.57		
LOC100862313	2.43	Gm19831	-31.16	LOC100861862	-3.04		
LOC100862094	3.28	Gm19868	-11.73	LOC100861882	-11.19		
LOC100861642	4.22	Gm19886	-2.96	LOC100861939	-2.33		
Gm15961; RP23-455J6.4	2.58	Gm19933	-4.2	LOC100861967	-2.07		
LOC100862193	2.26	Gm19974	-2.38	LOC100862063	-2.91		
LOC100862107	2.4	Gm19976	-5.24	LOC100862073	-2.12		
Gm19491	2.55	Gm20077	-6.79	LOC100862081	-3.14		
Gm20349	3.01	Gm20265	2.22	LOC100862086	-12.89		
LOC100861804	2.17	Gm20417; RP24-458F14.3	-4.91	LOC100862094	-7.85		
Gm19976	2.18	Gm22701	-2.14	LOC100862107	-2.04		
Gm19933	3.43	Gm23221	2.13	LOC100862145	-2.22		

Table S5. Effect of the high glycemic diet (HGD) with and without soluble epoxide hydrolase inhibitor (sEHI) on the expression of small nucleolar RNAs (snoRNAs) in hippocampal microvessels.

HGD vs LGD		HGD+sEHI vs HGD					
Gene Symbol	Fold Change	Gene Symbol	Fold Change	Gene Symbol	Fold Change	Gene Symbol	Fold Change
Gm22043	-2.36	Snord116l1	-27.05	Gm25428	2.36	Gm24658	-22.19
Gm22289	11.37	Snora34	-9.51	Gm25128	-5.89	Gm23359	-22.19
Gm22303	3.64	Snord116l2	-27.05	Snord14e	-17.03	Gm24742	-22.19
Gm22358	7.39	Gm24400	-17.8	Gm22631	-31.72	Gm23696	-22.19
Gm22501	2.36	Gm22289	-36.67	Gm24233	2.31	Gm26094	-22.19
Gm22663	-2.53	Gm24921	2.52	Gm23089	-28.78	Gm23511	-4.04
Gm23448	-2.23	Gm24336	-2.94	Gm24711	-28.78	Gm23141	-3.13
Gm23510	-3.07	Gm22748	-4.35	Gm26188	-28.78	Gm23119	-5.54
Gm24400	19.44	Snord16a	-3.55	Gm25839	2.77	Gm25074	-22.79
Gm24621	-2.3	Gm25138	-2.03	Gm22173	-32.86	Gm23308	2.47
Gm24783	-2.5	Gm22584	-20.81	Gm24691	2.05	Snord116l1	-27.05
Gm24983	-3.41	Gm24913	2.6	Gm26502	-36.66	Snord116l2	-27.05
Gm25153	-2.23	Gm26365	-3.59	Gm23313	-36.66	Snord116	-27.05
Gm25188	11.15	Gm23862	-21.04	Gm23446	-36.66	Snord116l1	-27.05
Gm25581	-2.59	Gm24518	-8.64	Gm25407	2.14	Snord116l2	-27.05
Gm25635	4.17	Gm24079	2.2	Gm22358	-9.84	Snord116	-27.05
Gm25856	-10.37	Gm23407	2.14	Gm26032	-21.9	Snord116l1	-27.05
Gm26361	-2.07	Gm24264	-16.79	Gm22046	-21.9	Snord116l2	-27.05
Snord14e	16.58	Gm22131	-13.3	Gm25471	-21.9	Snora34	-9.51
Snord16a	4.28	Gm25157	-16.4	Gm25210	-21.9	Snord116l1	-27.05
Snord82	-5.52	Gm22941	-16.4	Gm25350	-22.19	Scarna13	-2.33
		Gm22258	-16.4	Gm22128	-22.19	Gm25474	-7.06
		Gm22851	-25.95	Gm26246	-22.19	Gm22047	-7.06
		Gm23953	-7.43	Gm25597	-22.19	Gm23619	-7.06
		Gm26332	-7.43	Gm26201	-22.19	Snora30	-6.12
		Gm22863	-7.43	Gm22812	-22.19	Gm22188	-20.29
		Gm24618	-7.43	Gm26433	-22.19	Gm25188	-12.16
		Gm22786	2.88	Gm23357	-22.19	Gm26097	-4.78
		Gm25944	-6.97	Gm22110	-22.19	Gm26411	2.03
		Gm25615	-9.56	Gm23724	-22.19	Scarna9	-2.93
		Snord82	5.59	Gm23549	-22.19	Gm23260	2.41
		Gm24729	2.27	Gm26223	-22.19	Gm25635	-3.88
		Gm23767	-16.71	Gm23524	-22.19	Gm23458	-3.79
		Gm24298	-2.26	Gm26136	-22.19	Gm23322	2.64
		Gm22776	-12.48	Snord92	-2.65	Gm24463	-5.25
		Gm24008	2.11	Gm22297	2.89	Gm23316	2.21
		Gm25506	-2.81	Gm22868	2.18	Gm26170	2.24

	Gm22510	-3.06	Gm22303	-3.94	Gm24926	-2.94
	Gm26390	-3.06	Gm23622	2.23	Gm22632	-2.94
	Gm26336	-3.06	Gm22501	-2.39	Gm22252	-2.05
	Snord42a	-7.19	Gm26202	-10.66	Snord17	-2.02
	Gm25759	-2.59	Gm26358	2.54		
	Gm24689	2.02	Gm25224	3.56		

Table S6: Differentially expressed genes for the high glycemic diet (HGD) with soluble epoxide hydrolase inhibitor (sEHI) compared to without sEHI.

Gene Symbol	Description	Fold Change
Gm25911	predicted gene, 25911 [Source:MGI Symbol;Acc:MGI:	-472.71
Pcyox1	prenylcysteine oxidase 1	-2.27
Gm24245	predicted gene, 24245 [Source:MGI Symbol;Acc:MGI:	-295.98
Gm24270	predicted gene, 24270 [Source:MGI Symbol;Acc:MGI:	-295.98
Gm19738	PREDICTED: predicted gene, 19738, transcript varian	-8.16
Gm25732	predicted gene, 25732 [Source:MGI Symbol;Acc:MGI:	-6.68
Myl9	myosin, light polypeptide 9, regulatory	-2.33
Gm24187	predicted gene, 24187 [Source:MGI Symbol;Acc:MGI:	-361.17
		2.12
		-3.03
Copb2	coatomer protein complex, subunit beta 2 (beta prime)	-2.17
Gm19886	PREDICTED: predicted gene, 19886 (Gm19886), mis	-2.96
Gm23388	predicted gene, 23388 [Source:MGI Symbol;Acc:MGI:	-64.54
LOC100862148	PREDICTED: uncharacterized LOC100862148 (LOC	2.18
Cox5b	cytochrome c oxidase subunit Vb	-8.17
Gm19767	PREDICTED: predicted gene, 19767 (Gm19767), mis	-3.32
Gm3608	predicted gene 3608 [Source:MGI Symbol;Acc:MGI:38	-4.8
LOC100862216	PREDICTED: uncharacterized LOC100862216, transcript variant 1 (LOC100862216), miscRNA.; PREDICTED: uncharacterized LOC100862216, transcript variant 2 (LOC100862216), miscRNA.	-8
Atp2b4; Mir6903	ATPase, Ca++ transporting, plasma membrane 4; micr	-3.03
1700020I14Rik	RIKEN cDNA 1700020I14 gene	-2.43
Mir669b	microRNA 669b	-3.15
Nfe2l1	nuclear factor, erythroid derived 2,-like 1	-2.85
Apoa1bp	apolipoprotein A-I binding protein	-5.11
Gm7381	predicted gene 7381 [Source:MGI Symbol;Acc:MGI:36	-10.83
Rab24	RAB24, member RAS oncogene family	-4.27
Gm14870	predicted gene 14870 [Source:MGI Symbol;Acc:MGI:3	-3.55
Dnajc4	DnaJ (Hsp40) homolog, subfamily C, member 4	-2.26
		2.58
Rplp0	ribosomal protein, large, P0	-2.55
Gm24400	predicted gene, 24400 [Source:MGI Symbol;Acc:MGI:	-17.8
Cox6a1	cytochrome c oxidase subunit VIa polypeptide 1	-5.72
Gm11400	predicted gene 11400 [Source:MGI Symbol;Acc:MGI:3	-2.02
Gm22289	predicted gene, 22289 [Source:MGI Symbol;Acc:MGI:	-36.67
Gm9103	predicted gene 9103 [Source:MGI Symbol;Acc:MGI:36	-2.07
Dynlrb1	dynein light chain roadblock-type 1	-4.25
AY036118	cDNA sequence AY036118	-3.33
Gm7497	predicted gene 7497 [Source:MGI Symbol;Acc:MGI:36	-3.93
		-7.87

Capza1	capping protein (actin filament) muscle Z-line, alpha 1	-2.59
Mir6338	microRNA 6338	2.47
Gm14450	predicted gene 14450 [Source:MGISymbol;Acc:MGI:3	-2.01
Ndufa9	NADH dehydrogenase (ubiquinone) 1 alpha subcomple	-3.22
Eif2b5	eukaryotic translation initiation factor 2B, subunit 5 eps	-2.05
		-2.87
Myh11	myosin, heavy polypeptide 11, smooth muscle	-2.17
LOC100862313	PREDICTED: uncharacterized LOC100862313, transcript variant 1 (LOC100862313), miscRNA.; PREDICTED: uncharacterized LOC100862313, transcript variant 2 (LOC100862313), miscRNA.	-7.1
		2.97
Zfand3	zinc finger, AN1-type domain 3	-2.15
Ezh1	enhancer of zeste 1 polycomb repressive complex 2 sub	-2.27
		-4.16
LOC100862384	PREDICTED: uncharacterized LOC100862384 (LOC	-3.6
Gm14418	predicted gene 14418 [Source:MGISymbol;Acc:MGI:3	-3.64
Gm12074	predicted gene 12074 [Source:MGISymbol;Acc:MGI:3	-3.8
		-2.9
Brms1l	breast cancer metastasis-suppressor 1-like	-2.48
		2.33
Puf60	poly-U binding splicing factor 60	-2.23
Gm6054	predicted gene 6054 [Source:MGISymbol;Acc:MGI:36	-3.07
Gm13050	predicted gene 13050 [Source:MGISymbol;Acc:MGI:3	-2.34
Eif3s6-ps1	eukaryotic translation initiation factor 3, subunit 6, pse	-11.05
Psmc1	protease (prosome, macropain) 26S subunit, ATPase 1;	-3.93
Gm11675	predicted gene 11675 [Source:MGISymbol;Acc:MGI:3	-4.8
Apod	apolipoprotein D	-41.9
		-2.31
Gm11273	predicted gene 11273	-8.22
Psmc5	protease (prosome, macropain) 26S subunit, ATPase 5	-3.59
		-3.03
LOC100861762	PREDICTED: uncharacterized LOC100861762 (LOC	-2.26
Gm9625	predicted gene 9625 [Source:MGISymbol;Acc:MGI:37	-3.32
Ndufv3	NADH dehydrogenase (ubiquinone) flavoprotein 3	-2.27
Trf	transferrin	-3.72
		-4.41
Gm5967	predicted gene 5967 [Source:MGISymbol;Acc:MGI:36	-2.2
Hba-a2; Hba-a1	hemoglobin alpha, adult chain 2; hemoglobin alpha, adult chain 1 (Hba-a1), mRNA.; hemoglobin alpha, adult chain 1; Synthetic construct Mus musculus clone IMAGE:100062289, MGC:190450 hemoglobin alpha, adult chain 2 (Hba-a2) mRNA, encodes complete protein.	-10.08

Acta2	actin, alpha 2, smooth muscle, aorta	-6.33
Gm14330; AC124590.1	predicted gene 14330 [Source:MGI Symbol;Acc:MGI:3	2.06
Ost4	oligosaccharyltransferase 4 homolog (S. cerevisiae)	-3.63
LOC100503279	PREDICTED: uncharacterized LOC100503279 (LOC	-5.4
		-4.23
		-3.2
		-14.52
Jag1	jagged 1	-2.56
Gm24921	predicted gene, 24921 [Source:MGI Symbol;Acc:MGI:	2.52
Nucb2	nucleobindin 2	-2.89
Gm1866	predicted gene 1866 [Source:MGI Symbol;Acc:MGI:30	-2.63
		-5.77
		-2.69
Amd2; Amd1	S-adenosylmethionine decarboxylase 2; S-adenosylmet	-3.65
Gm19774	PREDICTED: predicted gene, 19774 (Gm19774), mR	-32.91
		2.14
Ppp6c	protein phosphatase 6, catalytic subunit	-2.29
		2.2
		2.06
		-4.53
LOC100862318	PREDICTED: uncharacterized LOC100862318, transcript variant 1 (LOC100862318), miscRNA.; PREDICTED: uncharacterized LOC100862318, transcript variant 2 (LOC100862318), miscRNA.; PREDICTED: uncharacterized LOC100862318, transcript variant 3 (LOC100862318), miscRNA.	-3.27
LOC100862073	PREDICTED: uncharacterized LOC100862073 (LOC	-2.12
Eif5al3-ps	eukaryotic translation initiation factor 5A-like 3, pseud	-4.18
Nt5c	5,3-nucleotidase, cytosolic; 5',3'-nucleotidase, cytosolic	-2.14
Ndufa10	NADH dehydrogenase (ubiquinone) 1 alpha subcomple	-2.64
Gm12727	predicted gene 12727 [Source:MGI Symbol;Acc:MGI:3	-2.41
Ociad2	OCIA domain containing 2	-2.44
Gm14269	predicted gene 14269 [Source:MGI Symbol;Acc:MGI:3	-3.64
Gm14292	predicted gene 14292 [Source:MGI Symbol;Acc:MGI:3	-11.18
Gm12183	predicted gene 12183	-2.14
Hba-a2; Hba-a1	hemoglobin alpha, adult chain 2 (Hba-a2), mRNA.; hemoglobin alpha, adult chain 1; hemoglobin alpha, adult chain 2; hemoglobin alpha, adult chain 1, mRNA (cDNA clone MGC:57888 IMAGE:5684314), complete cds.; Synthetic construct Mus musculus clone IMAGE:100062289, MGC:190450 hemoglobin alpha, adult chain 2 (Hba-a2) mRNA, encodes complete protein.	-17.99

Gm24336	predicted gene, 24336 [Source:MGI Symbol;Acc:MGI:	-2.94
		3.2
Mrpl24	mitochondrial ribosomal protein L24	-2.2
Gm13573	predicted gene 13573 [Source:MGI Symbol;Acc:MGI:3	-3.1
Flna	filamin, alpha	-3.37
Tra2a	transformer 2 alpha homolog (Drosophila)	-2.52
		2.11
Gm17756	PREDICTED: predicted gene, 17756 (Gm17756), mR	-2.77
LOC100862145	PREDICTED: uncharacterized LOC100862145, transcript variant 1 (LOC100862145), miscRNA.; PREDICTED: uncharacterized LOC100862145, transcript variant 2 (LOC100862145), miscRNA.	-2.22
Rnf168	ring finger protein 168	-2.37
		-2.4
		2.04
Gm13298; Fam205a3;	predicted gene 13298 (Gm13298), mRNA.; family with sequence similarity 205, member A3 [Source:MGI Symbol;Acc:MGI:5434953]; predicted gene, 21598	-2.11
Gm13298; Fam205a2	predicted gene 13298 (Gm13298), mRNA.; family with sequence similarity 205, member A2 [Source:MGI Symbol;Acc:MGI:3701946]; predicted gene 13298	-2.11
LOC100861967	PREDICTED: uncharacterized LOC100861967, transcript variant 1 (LOC100861967), miscRNA.; PREDICTED: uncharacterized LOC100861967, transcript variant 2 (LOC100861967), miscRNA.; PREDICTED: uncharacterized LOC100861967, transcript variant 3 (LOC100861967), miscRNA.	-2.07
13298; Fam205a4; Gm2	predicted gene 13298 (Gm13298), mRNA.; family with sequence similarity 205, member A4 [Source:MGI Symbol;Acc:MGI:5434294]; predicted gene, 20938	-2.33
Eef2	eukaryotic translation elongation factor 2	-4.05
Atp6v1c1	ATPase, H+ transporting, lysosomal V1 subunit C1	-3.21
Gm5777	predicted gene 5777 [Source:MGI Symbol;Acc:MGI:3	-5.24
Rpl18-ps2	ribosomal protein L18, pseudogene 2; 60S ribosomal p	-9.58
Gm12226	predicted pseudogene 12226 [Source:MGI Symbol;Acc	-2.09
Gm4804	predicted gene 4804 [Source:MGI Symbol;Acc:MGI:3	-2.22
Commd3	COMM domain containing 3	-2.8
		-2.11
Gm27626	predicted gene, 27626 [Source:MGI Symbol;Acc:MGI:	-52.49
LOC100862063	PREDICTED: uncharacterized LOC100862063 (LOC	-2.91
Gm14387	predicted gene 14387	-4.73

Gm16399	predicted pseudogene 16399 [Source:MGI Symbol;Acc	-5.29
Ufd1l	ubiquitin fusion degradation 1 like	-2.81
		-2.24
Gm15621	predicted gene 15621 [Source:MGI Symbol;Acc:MGI:	-32.12
Ndufa13	NADH dehydrogenase (ubiquinone) 1 alpha subcomple	-6.76
Mir6412	microRNA 6412	-4.87
		-39.98
Gm22735	predicted gene, 22735	-3.52
Gm14322	predicted gene 14322	-3.92
Dnttip1	deoxynucleotidyltransferase, terminal, interacting prote	-2.06
		-2.62
Gm8730	predicted pseudogene 8730 [Source:MGI Symbol;Acc:	-3.39
Rps6-ps1	ribosomal protein S6, pseudogene 1 [Source:MGI Sym	-29.64
		-2.15
Amd-ps3	S-adenosylmethionine decarboxylase, pseudogene 3 [S	-2.31
Slc38a3	solute carrier family 38, member 3	-2.16
Olfr1324	olfactory receptor 1324	2.25
Slc35b1	solute carrier family 35, member B1	-2.26
		-14.01
Hist1h2an	histone cluster 1, H2an	-6.92
Peg3os	Peg3 opposite strand	-2.28
Gm10224	predicted pseudogene 10224	-6.73
Gm22930	predicted gene, 22930	-2.81
Gm9143	predicted gene 9143 [Source:MGI Symbol;Acc:MGI:3	-2.26
Gm15500	predicted pseudogene 15500	-2.45
Gm19831	PREDICTED: predicted gene, 19831, transcript variant 1 (Gm19831), miscRNA.; PREDICTED: predicted gene, 19831, transcript variant 2 (Gm19831), miscRNA.; PREDICTED: predicted gene, 19831, transcript variant 3 (Gm19831), miscRNA.; PREDICTED: predicted gene, 19831, transcript variant 4 (Gm19831), miscRNA.; PREDICTED: predicted gene, 19831 (Gm19831), miscRNA.	-31.16
Kif1b	kinesin family member 1B	-2.29
Ppa1	pyrophosphatase (inorganic) 1	-3.11
Ptprg	protein tyrosine phosphatase, receptor type, G; Synthetic construct Mus musculus clone IMAGE:100068354, MGC:195898 protein tyrosine phosphatase, receptor type, G (Ptprg) mRNA, encodes complete protein.	-3.56
LOC100862246	PREDICTED: uncharacterized LOC100862246 (LOC	-4.48
Gm11367	predicted gene 11367 [Source:MGI Symbol;Acc:MGI:	-3.75
LOC100861642	PREDICTED: uncharacterized LOC100861642 (LOC	-7.61

		-10.91
Srsf2	serine/arginine-rich splicing factor 2	-2.82
Gm15733	predicted gene 15733 [Source:MGI Symbol;Acc:MGI:	-2.08
Gm23935	predicted gene, 23935 [Source:MGI Symbol;Acc:MGI:	-10.36
Ifna11	interferon alpha 11	2.52
Cd63	CD63 antigen	-2.69
LOC100862094	PREDICTED: uncharacterized LOC100862094 (LOC	-7.85
LOC100862081	PREDICTED: uncharacterized LOC100862081 (LOC	-3.14
Ndufb7	NADH dehydrogenase (ubiquinone) 1 beta subcomplex	-4.01
Sumo1	small ubiquitin-like modifier 1; SMT3 suppressor of m	-2.89
		2.93
2310036O22Rik	RIKEN cDNA 2310036O22 gene	-2.18
Gm22748	predicted gene, 22748 [Source:MGI Symbol;Acc:MGI:	-4.35
Zfp938	zinc finger protein 938	-6.35
Gm6055	predicted gene 6055	-3.18
Gnai2	guanine nucleotide binding protein (G protein), alpha i	-2.37
Gm9385	predicted pseudogene 9385	-2.39
Gm16209	predicted gene 16209 [Source:MGI Symbol;Acc:MGI:	-4.87
Gm22694	predicted gene, 22694 [Source:MGI Symbol;Acc:MGI:	-2.51
		2.07
Slc18b1	solute carrier family 18, subfamily B, member 1	-2.63
Gm12821	predicted gene 12821 [Source:MGI Symbol;Acc:MGI:	-2.07
Pet100	PET100 homolog (S. cerevisiae)	-2.05
Ano1	anoctamin 1, calcium activated chloride channel	-2.04
Epm2aip1	EPM2A (laforin) interacting protein 1	-2.3
Txnl1	thioredoxin-like 1	-5.57
LOC100862227	PREDICTED: uncharacterized LOC100862227, transcript variant 1 (LOC100862227), miscRNA.; PREDICTED: uncharacterized LOC100862227, transcript variant 2 (LOC100862227), miscRNA.; PREDICTED: uncharacterized LOC100862227, transcript variant 3 (LOC100862227), miscRNA.	-4.74
		2.03
Carm1	coactivator-associated arginine methyltransferase 1	-2.27
Cds2	CDP-diacylglycerol synthase (phosphatidate cytidylyltr	-4.35
Gm13171	predicted gene 13171 [Source:MGI Symbol;Acc:MGI:	-2.53
Ogfrl1	opioid growth factor receptor-like 1	-2.11
		2.85
Gm24093	predicted gene, 24093	-2.56
Ndufs3	NADH dehydrogenase (ubiquinone) Fe-S protein 3	-2.43
Gm24811	predicted gene, 24811 [Source:MGI Symbol;Acc:MGI:	-6.44
Nsmce2	non-SMC element 2 homolog (MMS21, S. cerevisiae)	-2.05
Rab7	RAB7, member RAS oncogene family	-18.91

Gm19976	PREDICTED: predicted gene, 19976, transcript variant 6 (Gm19976), miscRNA.; PREDICTED: predicted gene, 19976, transcript variant 8 (Gm19976), miscRNA.	-5.24
Pcna	proliferating cell nuclear antigen	-2.45
Nrd1; Mir761	nardilysin, N-arginine dibasic convertase, NRD convert	-2.76
		-10.09
Larp7	La ribonucleoprotein domain family, member 7; Synthetic construct Mus musculus clone IMAGE:100016333, MGC:184518 La ribonucleoprotein domain family, member 7 (Larp7) mRNA, encodes complete protein.	-2.07
Rock1	Rho-associated coiled-coil containing protein kinase 1; Synthetic construct Mus musculus clone IMAGE:100062871, MGC:190980 Rho-associated coiled-coil containing protein kinase 1 (Rock1) mRNA, encodes complete protein.	-4.52
Snord16a	small nucleolar RNA, C/D box 16A	-3.55
		2.06
Gm12632	predicted gene 12632 [Source:MGI Symbol;Acc:MGI:3	-2.3
Gm16412	predicted pseudogene 16412 [Source:MGI Symbol;Acc	-3.83
Gm15644	predicted gene 15644 [Source:MGI Symbol;Acc:MGI:3	-3.32
Gm11336	predicted gene 11336 [Source:MGI Symbol;Acc:MGI:3	-2.24
Gm12428	predicted gene 12428 [Source:MGI Symbol;Acc:MGI:3	-2.12
Gm5514	predicted gene 5514 [Source:MGI Symbol;Acc:MGI:36	-10.91
		2.06
Gm17919	predicted gene, 17919 [Source:MGI Symbol;Acc:MGI:	2.28
Gm2174	predicted gene 2174 [Source:MGI Symbol;Acc:MGI:37	-2.47
March6	membrane-associated ring finger (C3HC4) 6	-2.07
Vamp1	vesicle-associated membrane protein 1	-3.12
Snapc1	small nuclear RNA activating complex, polypeptide 1	-2.12
Mir692-1	microRNA 692-1	-12.13
Gm6065	predicted gene 6065 [Source:MGI Symbol;Acc:MGI:36	-12.87
Ighv1-63	immunoglobulin heavy variable V1-63	7.99
Mir5115	microRNA 5115 (Mir5115), microRNA.	-5.97
Fam213b	family with sequence similarity 213, member B	-2.21
LOC100861882	PREDICTED: uncharacterized LOC100861882, transc	-11.19
Gm15427	predicted pseudogene 15427	-59.61
		-3.8
Npm3-ps1	nucleoplasmin 3, pseudogene 1	-2.01
Gm6887	predicted gene 6887 [Source:MGI Symbol;Acc:MGI:36	-2.7
Gm23053	predicted gene, 23053 [Source:MGI Symbol;Acc:MGI:	-4.23
Gm19494	PREDICTED: predicted gene, 19494, transcript varian	-11.17
Tsn	translin	-3.45

Glrx3; Gm12669	glutaredoxin 3; glutaredoxin 3 pseudogene	-2.88
		-2.99
Psmb5; Mir686	proteasome (prosome, macropain) subunit, beta type 5;	-11.16
		-4.13
Cd248	CD248 antigen, endosialin	-2.38
Gm14165	predicted gene 14165 [Source:MGISymbol;Acc:MGI:3	-3.76
Gm5558	predicted gene 5558 [Source:MGISymbol;Acc:MGI:36	-38.62
LOC100861650	PREDICTED: uncharacterized LOC100861650, transc	-3.7
		2.02
Slc2a1	solute carrier family 2 (facilitated glucose transporter),	-4.03
Gm22800	predicted gene, 22800 [Source:MGI Symbol;Acc:MGI:	-3.08
Ndufb6	NADH dehydrogenase (ubiquinone) 1 beta subcomplex	-2.86
Nol4	nucleolar protein 4	-2.43
210418O10Rik; Gm172	RIKEN cDNA 2210418O10 gene [Source:MGI Symbo	-2
Gm14046	predicted gene 14046 [Source:MGISymbol;Acc:MGI:3	-5.16
Gm23566	predicted gene, 23566 [Source:MGI Symbol;Acc:MGI:	-7.29
Atp2a2	ATPase, Ca++ transporting, cardiac muscle, slow twitc	-3.95
LOC100861805	PREDICTED: uncharacterized LOC100861805 (LOC	-2.58
2900011O08Rik	RIKEN cDNA 2900011O08 gene	-2.9
Lancl1	LanC (bacterial lantibiotic synthetase component C)-lik	-2.79
Actb	actin, beta	-5.2
		2.1
Ldhb	lactate dehydrogenase B	-2.88
Gm16335	predicted gene 16335 [Source:MGISymbol;Acc:MGI:3	-18.59
Idh3g	isocitrate dehydrogenase 3 (NAD+), gamma	-2.96
Ighv5-12-4	Ighv5-12-4 immunoglobulin heavy variable 5-12-4	2.7
		-4.69
Gm14401	predicted gene 14401 [Source:MGISymbol;Acc:MGI:3	-2.15
Lonp2	lon peptidase 2, peroxisomal	-2.18
		2.26
		2.16
		-2.38
Ppp1r14a	protein phosphatase 1, regulatory (inhibitor) subunit 14	-2.6
Gm19595	PREDICTED: predicted gene, 19595 (Gm19595), mis	-31.12
		2.5
		2.07
Gm10221	predicted gene 10221 [Source:MGISymbol;Acc:MGI:3	-4.42
Gm23051	predicted gene, 23051	-3.34
LOC100861675	PREDICTED: uncharacterized LOC100861675 (LOC	-2.37
Kif5b	kinesin family member 5B	-3.33
Hist1h2af	histone cluster 1, H2af	-5.16
Gm6969	predicted pseudogene 6969 [Source:MGI Symbol;Acc:	-6.68
Gm10243	predicted gene 10243	-10.28
LOC100861832	PREDICTED: uncharacterized LOC100861832 (LOC	-2.64

Spata31d1b	spermatogenesis associated 31 subfamily D, member 1	2.06
Bnip2	BCL2/adenovirus E1B interacting protein 2	-2.6
Brcc3	BRCA1/BRCA2-containing complex, subunit 3	-2.37
		2.04
Prpf8	pre-mRNA processing factor 8	-3.88
Gm12254	predicted gene 12254 [Source:MGI Symbol;Acc:MGI:3	-7.23
		2.49
Hspa4	heat shock protein 4	-3.43
Ndufa1	NADH dehydrogenase (ubiquinone) 1 alpha subcomple	-17.79
Gm11263	predicted gene 11263 [Source:MGI Symbol;Acc:MGI:3	-12.45
		-2.07
LOC100862086	PREDICTED: uncharacterized LOC100862086 (LOC	-12.89
Cdr1	cerebellar degeneration related antigen 1	-8.42
Gm6023	predicted gene 6023 [Source:MGI Symbol;Acc:MGI:36	-2.29
Gm26826	predicted gene, 26826 [Source:MGI Symbol;Acc:MGI:	2.28
Qrich1	glutamine-rich 1	-2.82
Gm14513	predicted gene 14513 [Source:MGI Symbol;Acc:MGI:3	-3.05
Pcna-ps2	proliferating cell nuclear antigen pseudogene 2 [Source	-2.49
Ptprb	protein tyrosine phosphatase, receptor type, B	-4.76
Gm14416	predicted gene 14416	-4.56
Gm23190	predicted gene, 23190 [Source:MGI Symbol;Acc:MGI:	-8.94
Atp1a2	ATPase, Na+/K+ transporting, alpha 2 polypeptide	-3.97
Gm6341	predicted pseudogene 6341 [Source:MGI Symbol;Acc:	-10.14
m20417; RP24-458F14.	predicted gene 20417 [Source:MGI Symbol;Acc:MGI:5	-4.91
Gm11478	predicted gene 11478 [Source:MGI Symbol;Acc:MGI:3	-9.4
		2.07
Rpl3	ribosomal protein L3	-9.5
Gm25138	predicted gene, 25138 [Source:MGI Symbol;Acc:MGI:	-2.03
Gm15720	predicted gene 15720 [Source:MGI Symbol;Acc:MGI:3	-17.51
Cd81	CD81 antigen	-5.08
Crbn	cereblon; cereblon (Crbn), transcript variant 2, mRNA.	-2.64
Plpp3	phospholipid phosphatase 3	-3.04
Ybx1	Y box protein 1	-2.9
Ypel5	yippee-like 5 (Drosophila)	-2.09
Gm16200	predicted gene 16200 [Source:MGI Symbol;Acc:MGI:3	-13.16
Gm4883	predicted gene 4883	-6.02
		-6.45
Tardbp	TAR DNA binding protein	-3.85
Gm5564	predicted gene 5564 [Source:MGI Symbol;Acc:MGI:36	-9.77
Gm9166	predicted gene 9166 [Source:MGI Symbol;Acc:MGI:36	-4.54
Gm10020	predicted pseudogene 10020	-10.97
Slc12a2	solute carrier family 12, member 2	-2.67
Sept4	septin 4; septin 4 (Sept4), nuclear gene encoding mitoc	-2.48
		-2.31

Gm19660	PREDICTED: predicted gene, 19660, transcript varian	-4.58
Gstp1	glutathione S-transferase, pi 1	-2.7
Rpl9-ps1	ribosomal protein L9, pseudogene 1	-3.29
Mir669c	microRNA 669c	2.7
Anxa6	annexin A6	-2.09
Tgfb3	transforming growth factor, beta 3	-3.09
LOC100861862	PREDICTED: uncharacterized LOC100861862 (LOC	-3.04
Plxnc1	plexin C1	-2.02
		-3.46
Tecr	trans-2,3-enoyl-CoA reductase	-3.81
Gm23489	predicted gene, 23489	-3.41
		-9.67
Gm14421	predicted gene 14421 [Source:MGI Symbol;Acc:MGI:	-3.25
		-6.43
		-6.43
Gm23154	predicted gene, 23154 [Source:MGI Symbol;Acc:MGI:	-20.66
Mir7033	microRNA 7033	2.07
Psmd7	proteasome (prosome, macropain) 26S subunit, non-AT	-4.67
Olfr1167	olfactory receptor 1167	2.16
Stk39	serine/threonine kinase 39	-2.7
Hist1h2aj	histone cluster 1, H2aj	-6
		2.12
Mir3092	microRNA 3092	3
Vdac3	voltage-dependent anion channel 3	-7.88
Tm4sf1	transmembrane 4 superfamily member 1	-2.37
Mir669m-2	microRNA 669m-2	-3.26
Gm2214	predicted gene 2214 [Source:MGI Symbol;Acc:MGI:3	-11.64
Canx	calnexin	-4.36
		-8.14
Gm23019	predicted gene, 23019	-10.24
Gm23415	predicted gene, 23415 [Source:MGI Symbol;Acc:MGI:	-4.54
Cox7a2	cytochrome c oxidase subunit VIIa 2	-15.96
Gm22584	predicted gene, 22584 [Source:MGI Symbol;Acc:MGI:	-20.81
Mir6917	microRNA 6917	2.32
Gm24913	predicted gene, 24913 [Source:MGI Symbol;Acc:MGI:	2.6
Atp5g3	ATP synthase, H+ transporting, mitochondrial F0 comp	-2.16
Gm5449	predicted pseudogene 5449 [Source:MGI Symbol;Acc:	-3.18
Gm12922	predicted gene 12922 [Source:MGI Symbol;Acc:MGI:	-3.22
Gm16409	predicted gene 16409 [Source:MGI Symbol;Acc:MGI:	-56.32
Gm19474	PREDICTED: predicted gene, 19474, transcript varian	-3.56
Oaz1-ps	ornithine decarboxylase antizyme 1, pseudogene [Sour	-12.08
Gm14326	predicted gene 14326	-11.89
Gm10273	predicted pseudogene 10273 [Source:MGI Symbol;Acc	-5.28
Ptgds	prostaglandin D2 synthase (brain)	-3.44

Hsp90ab1	heat shock protein 90 alpha (cytosolic), class B membe	-6.05
Gm8865	predicted gene 8865 [Source:MGI Symbol;Acc:MGI:36	-5.39
Ccnd2	cyclin D2	-2.42
Gm16073	predicted gene 16073 [Source:MGI Symbol;Acc:MGI:3	-2.38
		-6.23
Ighv10-1	immunoglobulin heavy variable 10-1	2.74
Gm19974	PREDICTED: predicted gene, 19974 (Gm19974), mis	-2.38
Gm5566	predicted pseudogene 5566	-2.76
		-3.12
Uchl1	ubiquitin carboxy-terminal hydrolase L1	-3.33
Gm26365	predicted gene, 26365 [Source:MGI Symbol;Acc:MGI:	-3.59
Gm11836	predicted gene 11836	-4.54
Vim	vimentin	-2.42
Gm5160	predicted gene 5160 [Source:MGI Symbol;Acc:MGI:36	-3.38
Arl8b	ADP-ribosylation factor-like 8B	-4.8
Pitpnb	phosphatidylinositol transfer protein, beta	-2.32
Sdha	succinate dehydrogenase complex, subunit A, flavoprot	-2.35
m11008; RP23-83O23.	predicted gene 11008 [Source:MGI Symbol;Acc:MGI:3	-5.89
Adipor2	adiponectin receptor 2	-2.72
Cdk19	cyclin-dependent kinase 19	-5.55
Srsf7	serine/arginine-rich splicing factor 7	-2.64
Wdr74	WD repeat domain 74	-2.19
Gm23882	predicted gene, 23882 [Source:MGI Symbol;Acc:MGI:	-7.85
Gm11518	predicted gene 11518 [Source:MGI Symbol;Acc:MGI:3	-13.17
Gm10250	predicted pseudogene 10250 [Source:MGI Symbol;Acc	-6.59
Mir7667	microRNA 7667	2.29
Ndufs4	NADH dehydrogenase (ubiquinone) Fe-S protein 4	-6.66
		10.86
Gm6822	predicted pseudogene 6822	-3.17
Gpi1	glucose phosphate isomerase 1; glucose phosphate isom	-2.04
Micu2	mitochondrial calcium uptake 2	-2.36
		2.21
		2.17
Gm5848	PREDICTED: predicted pseudogene 5848 (Gm5848),	-4.07
Gm3208	predicted gene 3208 [Source:MGI Symbol;Acc:MGI:37	2.01
Gm8172	predicted pseudogene 8172 [Source:MGI Symbol;Acc:	-42.33
Gm5915	predicted pseudogene 5915	-4.87
Cops3	COP9 (constitutive photomorphogenic) homolog, subu	-3.25
Atp5a1	ATP synthase, H+ transporting, mitochondrial F1 comp	-2.7
Tuba1b	tubulin, alpha 1B	-4.16
Gm6139	predicted gene 6139	-5.9
BC002163	NADH dehydrogenase Fe-S protein 5 pseudogene; cDN	-3.77
Kif5a	kinesin family member 5A	-4.34
Crip1	cysteine-rich protein 1 (intestinal)	-3.12

Gm11539	predicted gene 11539 [Source:MGI Symbol;Acc:MGI:3	-3.23
Pcdh9	protocadherin 9; Synthetic construct Mus musculus clo	-3.07
D8Ertd738e	DNA segment, Chr 8, ERATO Doi 738, expressed	-11.87
Gm6265	predicted pseudogene 6265	-27
Gm11223	predicted gene 11223	-2.98
Calm3	calmodulin 3	-2.14
Atp5b	ATP synthase, H+ transporting mitochondrial F1 comp	-3.18
		3.91
Camk2g	calcium/calmodulin-dependent protein kinase II gamma	-2.28
Gm14403	predicted gene 14403	-4.33
Gm23862	predicted gene, 23862 [Source:MGI Symbol;Acc:MGI:	-21.04
Gcnt2	glucosaminyl (N-acetyl) transferase 2, I-branching enzy	-2.5
Gm6644	predicted gene 6644	-2.66
LOC100862001	PREDICTED: uncharacterized LOC100862001 (LOC	-2.11
Eef1b2	eukaryotic translation elongation factor 1 beta 2	-2.54
		-84.83
Gm12231	predicted gene 12231	-9.87
Rpl35a-ps2	ribosomal protein L35A, pseudogene 2	-12.82
Mertk	c-mer proto-oncogene tyrosine kinase; Synthetic constr	-2.1
Gm23086	predicted gene, 23086 [Source:MGI Symbol;Acc:MGI:	-3.3
Gm10123; Gm13493	predicted pseudogene 10123 [Source:MGI Symbol;Acc	-11.7
Srp9	signal recognition particle 9	-2.2
Serbp1	serpine1 mRNA binding protein 1	-2.66
Vti1b	vesicle transport through interaction with t-SNAREs 1E	-3.16
Scd1	stearoyl-Coenzyme A desaturase 1	-2.09
		2.29
		-11.15
Rpl27-ps1	ribosomal protein L27, pseudogene 1 [Source:MGI Sy	-9.94
LOC100861833	PREDICTED: uncharacterized LOC100861833 (LOC	-6.59
Prpf18	PRP18 pre-mRNA processing factor 18 homolog (yeas	-2.5
		2.35
Gm8566	predicted pseudogene 8566 [Source:MGI Symbol;Acc:	-80.01
Gm12517	predicted gene 12517 [Source:MGI Symbol;Acc:MGI:3	-3.89
Gm22610	predicted gene, 22610	-23.14
Gm24518	predicted gene, 24518 [Source:MGI Symbol;Acc:MGI:	-8.64
Gm19453	PREDICTED: predicted gene, 19453 (Gm19453), mis	-8.51
Gm20265	PREDICTED: predicted gene, 20265 (Gm20265), mis	2.22
Tspan3	tetraspanin 3	-7.24
		4.26
Gm19491	PREDICTED: predicted gene, 19491 (Gm19491), mis	-4.3
		-4.26
Gm27530	predicted gene, 27530 [Source:MGI Symbol;Acc:MGI:	2.1
Hnrnpa2b1	heterogeneous nuclear ribonucleoprotein A2/B1; hetero	-2.48
Gm24369	predicted gene, 24369 [Source:MGI Symbol;Acc:MGI:	-2.61

Rpl18a-ps1	ribosomal protein L18A, pseudogene 1	-15.24
Klf6	Kruppel-like factor 6	-3.01
Gm11810	predicted gene 11810	-10.06
		2
Gm4479	predicted gene 4479 [Source:MGI Symbol;Acc:MGI:37	-5.56
		-3.05
Mal	myelin and lymphocyte protein, T cell differentiation p	-4.77
Myl6	myosin, light polypeptide 6, alkali, smooth muscle and	-3.26
Slc6a20a	solute carrier family 6 (neurotransmitter transporter), m	-4.82
Gm14481	predicted gene 14481 [Source:MGI Symbol;Acc:MGI:3	2.04
Hipk3; Mir1902	homeodomain interacting protein kinase 3; microRNA	-2.02
Gm14429	predicted gene 14429 [Source:MGI Symbol;Acc:MGI:3	-2.8
Dcn	decorin	-7.16
Gm10198	predicted gene 10198 [Source:MGI Symbol;Acc:MGI:3	6.08
Rpl14-ps1	ribosomal protein L14, pseudogene 1 [Source:MGI Sy	-5.07
Zfp931	zinc finger protein 931	-3.24
Gm12778	predicted gene 12778 [Source:MGI Symbol;Acc:MGI:3	-35.28
		20.61
		20.61
		20.61
		20.61
		20.61
		20.61
		-2.46
		2.78
Gm24079	predicted gene, 24079 [Source:MGI Symbol;Acc:MGI:	2.2
		-3.1
Gm11060	predicted gene 11060 [Source:MGI Symbol;Acc:MGI:3	-2.38
		-3.12
Olfr692	olfactory receptor 692	2.09
Gm10080	predicted gene 10080 [Source:MGI Symbol;Acc:MGI:3	-4.94
Gm10015	predicted gene 10015	-3.95
Rpl35a-ps7	ribosomal protein L35A, pseudogene 7	-24.39
Gm13144	predicted gene 13144	-2.3
Gm13862	predicted gene 13862 [Source:MGI Symbol;Acc:MGI:3	-2.23
		-3.47
Gm25063	predicted gene, 25063 [Source:MGI Symbol;Acc:MGI:	-12.6
Rpl18-ps1	ribosomal protein L18, pseudogene 1	-36.34
LOC100862193	PREDICTED: uncharacterized LOC100862193 (LOC	-2.7
Gm10275	predicted pseudogene 10275 [Source:MGI Symbol;Acc	-2.49
Rplp1	ribosomal protein, large, P1	-27.51
m14944; RP23-302B23	predicted gene 14944; histocompatibility 2, class II anti	-2.46
Tmed10	transmembrane emp24-like trafficking protein 10 (yeas	-3.78
Tuba1a	tubulin, alpha 1A	-3.92

E030024N20Rik	RIKEN cDNA E030024N20 gene [Source:MGI Symbo	-5.89
		-3.94
Scp2-ps2	sterol carrier protein 2, pseudogene 2 [Source:MGI Sy	-3.56
Rps19bp1	ribosomal protein S19 binding protein 1	-2.06
Clk3	CDC-like kinase 3	-2.79
Vps28	vacuolar protein sorting 28 (yeast)	-4.09
Akap8l	A kinase (PRKA) anchor protein 8-like	-2.08
		2.06
Gm23407	predicted gene, 23407 [Source:MGI Symbol;Acc:MGI:	2.14
Gm10171	predicted gene 10171	-6.06
Ptn	pleiotrophin	-3.13
		2.21
Gm17571	predicted gene, 17571 [Source:MGI Symbol;Acc:MGI:	-29.99
Rpl3-ps2	ribosomal protein L3, pseudogene 2	-5.87
Gm14391; Gm6710	predicted gene 14391; predicted gene 6710	-2.02
		2.03
Gm14732	predicted gene 14732 [Source:MGI Symbol;Acc:MGI:	2.31
Gtf3c6	general transcription factor IIIC, polypeptide 6, alpha	-4.93
Gm24264	predicted gene, 24264 [Source:MGI Symbol;Acc:MGI:	-16.79
Gm14327	predicted gene 14327	-2.84
Ubc; Uba52	ubiquitin C; ubiquitin A-52 residue ribosomal protein f	-3.58
		2.28
		20.82
		20.82
		20.82
		20.82
		20.82
Ncl	nucleolin	-2.76
Gm14150	predicted gene 14150	-5.13
		-6.71
		2.02
Rpl35a-ps5	ribosomal protein L35A, pseudogene 5	-9.86
Gm26904	predicted gene, 26904 [Source:MGI Symbol;Acc:MGI:	-3.87
Tmod2	tropomodulin 2	-2.9
Gm14279	predicted gene 14279	-44.08
Gm11631	predicted gene 11631 [Source:MGI Symbol;Acc:MGI:	-13.31
Wsb1	WD repeat and SOCS box-containing 1	-3.87
		-6.15
Rpl8	ribosomal protein L8	-8.63
Cisd1	CDGSH iron sulfur domain 1	-2.78
Igfbp7	insulin-like growth factor binding protein 7	-4.28
Gm22131	predicted gene, 22131 [Source:MGI Symbol;Acc:MGI:	-13.3
Rpl29; Gm8210	ribosomal protein L29; predicted pseudogene 8210	-6.84
Gm16355	predicted gene 16355 [Source:MGI Symbol;Acc:MGI:	-2.95

Pnmal1	PNMA-like 1	-2.25
Gm25000	predicted gene, 25000 [Source:MGI Symbol;Acc:MGI:	-12.27
		-2.03
Gm26050	predicted gene, 26050 [Source:MGI Symbol;Acc:MGI:	-3.82
Rpl18a	ribosomal protein L18A	-8.01
Gm25157	predicted gene, 25157 [Source:MGI Symbol;Acc:MGI:	-16.4
Gm22941	predicted gene, 22941 [Source:MGI Symbol;Acc:MGI:	-16.4
Gm22258	predicted gene, 22258 [Source:MGI Symbol;Acc:MGI:	-16.4
Gm10108	predicted pseudogene 10108 [Source:MGI Symbol;Acc	-7.87
Rplp0-ps1	ribosomal protein, large, P0, pseudogene 1	-2.25
Cfdp1	craniofacial development protein 1	-2.5
Gm22851	predicted gene, 22851 [Source:MGI Symbol;Acc:MGI:	-25.95
Gm12038	predicted gene 12038 [Source:MGI Symbol;Acc:MGI:3	-8.32
		-4.53
Ube2l3	ubiquitin-conjugating enzyme E2L 3	-3.71
Gm3934	PREDICTED: predicted gene 3934 (Gm3934), mRNA	-2.78
Rpl28-ps1	ribosomal protein L28, pseudogene 1	-2.74
Gm15846	predicted gene 15846 [Source:MGI Symbol;Acc:MGI:3	-5.32
Ifi27	interferon, alpha-inducible protein 27	-2.29
Gm11249	predicted gene 11249 [Source:MGI Symbol;Acc:MGI:3	-6.7
		-2.3
Etohi1	ethanol induced 1	-3.95
		-7
Rps6-ps3	ribosomal protein S6, pseudogene 3 [Source:MGI Sym	-21.51
Gm6444	predicted gene 6444 [Source:MGI Symbol;Acc:MGI:36	-4
Spry2	sprouty homolog 2 (Drosophila)	-2.06
Gm12481	predicted gene 12481	-3.19
Gng11	guanine nucleotide binding protein (G protein), gamma	-2.13
Gm16021	predicted gene 16021 [Source:MGI Symbol;Acc:MGI:3	-7.82
Gm6767	predicted gene 6767 [Source:MGI Symbol;Acc:MGI:36	-5.19
		-2.43
Gm14537	predicted gene 14537 [Source:MGI Symbol;Acc:MGI:3	2.91
		-3.55
		-3.24
		-2.09
		2.03
Gm13772	predicted gene 13772 [Source:MGI Symbol;Acc:MGI:3	-7.08
Hsp90b1	heat shock protein 90, beta (Grp94), member 1	-4.09
Gm8842	PREDICTED: predicted gene 8842, transcript variant	-2.02
Polr2f	polymerase (RNA) II (DNA directed) polypeptide F	-2.68
Sod1	superoxide dismutase 1, soluble	-6.86
D10Jhu81e	DNA segment, Chr 10, Johns Hopkins University 81 ex	-2.13
Elovl4	elongation of very long chain fatty acids (FEN1/Elo2, S	-2.17
Ik	IK cytokine	-5.17

Atp6ap2	ATPase, H+ transporting, lysosomal accessory protein	-2.82
Gm22232	predicted gene, 22232 [Source:MGI Symbol;Acc:MGI:	-3.3
Gm10181	predicted gene 10181 [Source:MGI Symbol;Acc:MGI:	7.22
Gm14288; Gm14440	predicted gene 14288; predicted gene 14440 (Gm1444(	-2.94
Gm6285	predicted gene 6285 [Source:MGI Symbol;Acc:MGI:3	-2.01
Gm25040	predicted gene, 25040 [Source:MGI Symbol;Acc:MGI:	-8.71
Gm22243	predicted gene, 22243 [Source:MGI Symbol;Acc:MGI:	-3.27
Mir684-1	microRNA 684-1 (Mir684-1), microRNA.; microRNA	-172.48
Gm5921	predicted gene 5921 [Source:MGI Symbol;Acc:MGI:3	-3.7
Slc38a2	solute carrier family 38, member 2	-2.05
Zmat2	zinc finger, matrin type 2	-2.24
Msl2	male-specific lethal 2 homolog (Drosophila); male-spe	-3.02
		-10.43
LOC100861724	PREDICTED: uncharacterized LOC100861724 (LOC	2.59
Gm14296	predicted gene 14296	-2.16
LOC432823	similar to hypothetical protein MGC37588, mRNA (cD	-11.59
Pitpna	phosphatidylinositol transfer protein, alpha	-2.21
Pcbd2	pterin 4 alpha carbinolamine dehydratase/dimerization	-3.21
		-4.3
		-3.55
Rpl31-ps20	ribosomal protein L31, pseudogene 20	-4.8
H2afz	H2A histone family, member Z	-16.2
Gm23953	predicted gene, 23953 [Source:MGI Symbol;Acc:MGI:	-7.43
Gm26332	predicted gene, 26332 [Source:MGI Symbol;Acc:MGI:	-7.43
Gm22863	predicted gene, 22863 [Source:MGI Symbol;Acc:MGI:	-7.43
Gm24618	predicted gene, 24618 [Source:MGI Symbol;Acc:MGI:	-7.43
Rpl27-ps2	ribosomal protein L27, pseudogene 2	-7.67
Rps2-ps13	ribosomal protein S2, pseudogene 13	-5.79
		-4.91
Rpl15-ps3	ribosomal protein L15, pseudogene 3	-3.14
Gm16111	predicted gene 16111 [Source:MGI Symbol;Acc:MGI:	-2.13
Mir466f-4	microRNA 466f-4 [Source:MGI Symbol;Acc:MGI:378	-8.96
Gm23368	predicted gene, 23368 [Source:MGI Symbol;Acc:MGI:	-5.59
Pdxk	pyridoxal (pyridoxine, vitamin B6) kinase	-4.36
Raph1	Ras association (RalGDS/AF-6) and pleckstrin homolo	-2.77
Prkag1	protein kinase, AMP-activated, gamma 1 non-catalytic	-5.27
Ctsb	cathepsin B	-2.54
Gm24026	predicted gene, 24026 [Source:MGI Symbol;Acc:MGI:	-5.18
Gm11361	predicted pseudogene 11361 [Source:MGI Symbol;Acc	-6.11
Sparcl1	SPARC-like 1	-4.63
Smdt1	single-pass membrane protein with aspartate rich tail 1	-8.87
		-2.16
Gm5451	predicted gene 5451	-23.3
Gm8991	predicted pseudogene 8991 [Source:MGI Symbol;Acc:	-2.52

Mir1191	microRNA 1191	-28.06
Gm10029	predicted gene 10029	-53.93
Rpl31-ps14	ribosomal protein L31, pseudogene 14	-6.82
Scd2; Mir5114	stearoyl-Coenzyme A desaturase 2; microRNA 5114	-5.72
Gm27722	predicted gene, 27722 [Source:MGI Symbol;Acc:MGI:	4.66
Gm14288; Gm14440	predicted gene 14288 (Gm14288), mRNA.; predicted g	-2.06
Rpl35a-ps4	ribosomal protein 35A, pseudogene 4	-84.44
Rpl35a-ps6	ribosomal protein L35A, pseudogene 6	-84.44
Rpl35a-ps3	ribosomal protein L35A, pseudogene 3	-84.44
Olfr1055	olfactory receptor 1055	-4.06
Gm22786	predicted gene, 22786 [Source:MGI Symbol;Acc:MGI:	2.88
Gm25944	predicted gene, 25944 [Source:MGI Symbol;Acc:MGI:	-6.97
Gm13433	predicted gene 13433 [Source:MGI Symbol;Acc:MGI:	-5.78
Copz1	coatomer protein complex, subunit zeta 1	-2.88
		-3.28
Gm12468	predicted gene 12468	-2.26
Olfr1274-ps	olfactory receptor 1274, pseudogene	2.35
Maf	avian musculoaponeurotic fibrosarcoma (v-maf) AS42	-2.22
		2.68
		2.45
		2.06
		4.29
m3893; 4933409K07Ri	predicted gene 3893; RIKEN cDNA 4933409K07 gene	-2.04
Gm6311	predicted gene 6311 [Source:MGI Symbol;Acc:MGI:3	-12.63
Gm8203	predicted pseudogene 8203	-28.99
Gm14399	predicted gene 14399	-5.78
100862170; 4933409K0	PREDICTED: uncharacterized LOC100862170 (LOC	-3.18
Mir6989	microRNA 6989	-19.52
		-2.09
		2.05
Gm17604	predicted gene, 17604 [Source:MGI Symbol;Acc:MGI:	19.68
Gatc	glutamyl-tRNA(Gln) amidotransferase, subunit C; glut	-5.16
Gm25615	predicted gene, 25615 [Source:MGI Symbol;Acc:MGI:	-9.56
Gm6238	predicted pseudogene 6238	-2.25
Gm25738	predicted gene, 25738	-4.36
1700055C04Rik	RIKEN cDNA 1700055C04 gene	2.01
Gm26308	predicted gene, 26308 [Source:MGI Symbol;Acc:MGI:	2.42
		-3.1
Rpl5-ps1	ribosomal protein L5, pseudogene 1	-11.5
Hprt	hypoxanthine guanine phosphoribosyl transferase	-3.58
		4.85
Gm12366	predicted gene 12366 [Source:MGI Symbol;Acc:MGI:	-5.29
		-2.31
Gm12666	predicted gene 12666	-2.2

		-11.84
Gm8420	predicted gene 8420 [Source:MGI Symbol;Acc:MGI:36	-2.77
		3.05
Tspan31	tetraspanin 31	-3.5
2410015M20Rik	RIKEN cDNA 2410015M20 gene	-2.63
		-11.99
Psma7	proteasome (prosome, macropain) subunit, alpha type 7	-2.32
Itm2b	integral membrane protein 2B	-30.93
Akr1a1	aldo-keto reductase family 1, member A1 (aldehyde red	-2.7
Gm10073	predicted pseudogene 10073 [Source:MGI Symbol;Acc	-6.75
Gm27219	predicted gene 27219 [Source:MGI Symbol;Acc:MGI:5	-4.58
Prdx4	peroxiredoxin 4	-3.75
Snord82	small nucleolar RNA, C/D box 82	5.59
Gm26250	predicted gene, 26250 [Source:MGI Symbol;Acc:MGI:	-2.24
		2.24
Gm6293	predicted pseudogene 6293	-3.61
		2.35
Gm6712	predicted gene 6712 [Source:MGI Symbol;Acc:MGI:36	-5.59
Gm24729	predicted gene, 24729 [Source:MGI Symbol;Acc:MGI:	2.27
Dld	dihydrolipoamide dehydrogenase	-2.01
Gm22201	predicted gene, 22201 [Source:MGI Symbol;Acc:MGI:	2.12
Rpl31-ps17	ribosomal protein L31, pseudogene 17	-4.93
Far1	fatty acyl CoA reductase 1	-2.09
Gm17150	predicted gene 17150 [Source:MGI Symbol;Acc:MGI:4	-6.89
Gm6109	PREDICTED: predicted gene 6109 (Gm6109), mRNA	-6.54
Rps4x	ribosomal protein S4, X-linked; ribosomal protein S4,	-7.79
		-2.92
Rpl17-ps4	ribosomal protein L17, pseudogene 4	-3.05
Gm11930	predicted gene 11930 [Source:MGI Symbol;Acc:MGI:3	-3.5
		-3.29
		-4.97
Gm25693	predicted gene, 25693 [Source:MGI Symbol;Acc:MGI:	-3.26
Cct5	chaperonin containing Tcp1, subunit 5 (epsilon)	-3.15
Gm13246	predicted gene 13246 [Source:MGI Symbol;Acc:MGI:3	-2.82
4632427E13Rik	RIKEN cDNA 4632427E13 gene	-2.41
Sar1b	SAR1 gene homolog B (S. cerevisiae)	-2.07
Nedd4	neural precursor cell expressed, developmentally down	-4.11
Gm12728	predicted gene 12728 [Source:MGI Symbol;Acc:MGI:3	-3.34
Rps15-ps2	ribosomal protein S15, pseudogene 2	-2.78
		2.57
Flrt2	fibronectin leucine rich transmembrane protein 2	-2.06
Gm10247	predicted gene 10247 [Source:MGI Symbol;Acc:MGI:3	-50.28
Gm9794	PREDICTED: predicted pseudogene 9794 (Gm9794),	-9.05
Gm26053	predicted gene, 26053	-18.06

Srsf11	serine/arginine-rich splicing factor 11; serine/arginine-r	-2.15
Rpl31-ps1	ribosomal protein L31, pseudogene 1 [Source:MGI Sy	-3.59
		-30.57
Gm11969	predicted gene 11969 [Source:MGI Symbol;Acc:MGI:3	-2.29
		-2.52
Gm12328	predicted gene 12328 [Source:MGI Symbol;Acc:MGI:3	-8.84
Gm23767	predicted gene, 23767 [Source:MGI Symbol;Acc:MGI:	-16.71
Rps6-ps2	ribosomal protein S6, pseudogene 2 [Source:MGI Sym	-2.02
Atp6v1e1	ATPase, H+ transporting, lysosomal V1 subunit E1	-3.51
Rpl19-ps12	ribosomal protein L19, pseudogene 12	-3.92
Resp18	regulated endocrine-specific protein 18	-7.6
Gm12341	predicted gene 12341 [Source:MGI Symbol;Acc:MGI:3	-3.1
Rps2-ps10	ribosomal protein S2, pseudogene 10 [Source:MGI Sym	-8.01
Rpl10a-ps1	ribosomal protein L10A, pseudogene 1	-7.58
Rpl31-ps11	ribosomal protein L31, pseudogene 11	-5.21
Slc6a6	solute carrier family 6 (neurotransmitter transporter, tau	-2.3
Ppargc1a	peroxisome proliferative activated receptor, gamma, co	-2.12
Gm24298	predicted gene, 24298 [Source:MGI Symbol;Acc:MGI:	-2.26
Cltb	clathrin, light polypeptide (Lcb)	-2.19
Gm17428	predicted gene, 17428 [Source:MGI Symbol;Acc:MGI:	6.35
Gm23045	predicted gene, 23045 [Source:MGI Symbol;Acc:MGI:	-3.02
Dnajc19	DnaJ (Hsp40) homolog, subfamily C, member 19	-2.88
Actg1; Mir6935	actin, gamma, cytoplasmic 1; microRNA 6935	-4.69
Mff	mitochondrial fission factor	-2.09
		8.92
Gm16199	predicted gene 16199 [Source:MGI Symbol;Acc:MGI:3	-5.9
		2.31
Ssfa2	sperm specific antigen 2	-2.53
Gm23728	predicted gene, 23728	-13.47
Gm20091	predicted gene, 20091	-11.19
Gm14813	predicted gene 14813 [Source:MGI Symbol;Acc:MGI:3	-2.07
2010107E04Rik	RIKEN cDNA 2010107E04 gene	-15.63
LOC100861939	PREDICTED: uncharacterized LOC100861939, transc	-2.33
Gm25428	predicted gene, 25428 [Source:MGI Symbol;Acc:MGI:	2.36
Gm12350	predicted gene 12350 [Source:MGI Symbol;Acc:MGI:3	-8.01
Gm27684	predicted gene, 27684 [Source:MGI Symbol;Acc:MGI:	-14.95
1110004F10Rik	RIKEN cDNA 1110004F10 gene	-2.14
Gm27935	predicted gene, 27935 [Source:MGI Symbol;Acc:MGI:	-5.91
Gm26581; RP23-58L22.	predicted gene, 26581 [Source:MGI Symbol;Acc:MGI:	2.19
Sdhb	succinate dehydrogenase complex, subunit B, iron sulfu	-2.14
Rpl4	ribosomal protein L4	-14.21
Gm4853	predicted pseudogene 4853	-3.65
Gm2389	predicted pseudogene 2389 [Source:MGI Symbol;Acc:	-8.22
Gm25128	predicted gene, 25128 [Source:MGI Symbol;Acc:MGI:	-5.89

Rps12-ps26	ribosomal protein S12, pseudogene 26 [Source:MGI Sy	-5.25
Gm14405	predicted gene 14405 (Gm14405), non-coding RNA.; p	-2.71
Tmem30	transmembrane protein 30A	-3.55
a Rap2a	RAS related protein 2a	-2.04
		-6.35
Gm11971	predicted gene 11971 [Source:MGI Symbol;Acc:MGI:3	-9.21
Snord14e	small nucleolar RNA, C/D box 14E	-17.03
Gm26469	predicted gene, 26469 [Source:MGI Symbol;Acc:MGI:	-6.14
Ndufa4	NADH dehydrogenase (ubiquinone) 1 alpha subcomple	-6.43
Snrpd2	small nuclear ribonucleoprotein D2	-2.5
Mir7053	microRNA 7053	2.07
Gm14288; Gm14435	predicted gene 14288 (Gm14288), mRNA.; predicted g	-2.32
Serinc1	serine incorporator 1	-11.63
Gm11172	predicted gene 11172 [Source:MGI Symbol;Acc:MGI:3	5.33
Gm22631	predicted gene, 22631 [Source:MGI Symbol;Acc:MGI:	-31.72
		-5.92
Gm25591	predicted gene, 25591 [Source:MGI Symbol;Acc:MGI:	-2.83
Gm13841; Rpl29	predicted gene 13841; ribosomal protein L29, mRNA (	-6.93
Gm24710	predicted gene, 24710 [Source:MGI Symbol;Acc:MGI:	-3.38
Gm24135	predicted gene, 24135 [Source:MGI Symbol;Acc:MGI:	-3.38
Gm13252	predicted gene 13252 [Source:MGI Symbol;Acc:MGI:3	-3.57
Mir28c	microRNA 28c (Mir28c), microRNA.	4.3
430; Gm14434; 0610010	predicted gene 14308 (Gm14308), mRNA.; predicted g	-4.69
Gm26818	predicted gene, 26818 [Source:MGI Symbol;Acc:MGI:	-3.34
Gm24233	predicted gene, 24233 [Source:MGI Symbol;Acc:MGI:	2.31
Gm8841	PREDICTED: predicted gene 8841 (Gm8841), mRNA	-7.06
Rpl3-ps1	ribosomal protein L3, pseudogene 1	-2.81
P4hb	prolyl 4-hydroxylase, beta polypeptide	-2.82
		-2.68
Mir684-1	microRNA 684-1 (Mir684-1), microRNA.; microRNA	-157.28
Mir684-1	microRNA 684-1 (Mir684-1), microRNA.; microRNA	-157.28
Mir684-1	microRNA 684-1 (Mir684-1), microRNA.; microRNA	-157.28
Mir684-1	microRNA 684-1 (Mir684-1), microRNA.; microRNA	-157.28
Mir684-1	microRNA 684-1 (Mir684-1), microRNA.; microRNA	-157.28
Selk	selenoprotein K	-2.54
Gm24586	predicted gene, 24586 [Source:MGI Symbol;Acc:MGI:	-4.47
Akt3	thymoma viral proto-oncogene 3	-3.09
Gm5844	predicted gene 5844 [Source:MGI Symbol;Acc:MGI:36	-2.33
Derl1	Der1-like domain family, member 1	-2.35
Gm14412	predicted gene 14412 [Source:MGI Symbol;Acc:MGI:3	-5.45
Gm15483	predicted gene 15483	-3.58
Gm16100	predicted gene 16100 [Source:MGI Symbol;Acc:MGI:3	-2.13
Gm10260	predicted gene 10260 [Source:MGI Symbol;Acc:MGI:3	-5.68
Cox4i1	cytochrome c oxidase subunit IV isoform 1	-2.51

Fxyd1	FXYD domain-containing ion transport regulator 1	-2.45
Ube2n	ubiquitin-conjugating enzyme E2N	-2.14
Rpl29-ps5	ribosomal protein L29, pseudogene 5 [Source:MGI Sy	-3.05
Mir1951	microRNA 1951	2.21
		-2.09
Gm23221	predicted gene, 23221	2.13
Gm27529	predicted gene, 27529 [Source:MGI Symbol;Acc:MGI:	-33.75
Rpl27a-ps2	ribosomal protein L27A, pseudogene 2 [Source:MGI S	-2.17
Gm17541	predicted gene, 17541 [Source:MGI Symbol;Acc:MGI:	-7.08
Gm20391	predicted gene 20391 [Source:MGI Symbol;Acc:MGI:5	2.46
Gm14419	predicted gene 14419	-5.8
Gabra3	gamma-aminobutyric acid (GABA) A receptor, subunit	-2.16
Rpl31-ps16	ribosomal protein L31, pseudogene 16	-6.09
Gm23089	predicted gene, 23089 [Source:MGI Symbol;Acc:MGI:	-28.78
Gm24711	predicted gene, 24711 [Source:MGI Symbol;Acc:MGI:	-28.78
Gm26188	predicted gene, 26188 [Source:MGI Symbol;Acc:MGI:	-28.78
		-2.43
Pik3r1	phosphatidylinositol 3-kinase, regulatory subunit, polyp	-2.16
Gm1840	predicted gene 1840 [Source:MGI Symbol;Acc:MGI:30	-12.07
Gm22030	predicted gene, 22030 [Source:MGI Symbol;Acc:MGI:	-5.74
Tmem147	transmembrane protein 147	-6.72
		-2.97
		-3.02
Gm5787	predicted gene 5787 [Source:MGI Symbol;Acc:MGI:38	-2.15
Gm1821	predicted gene 1821 (Gm1821), non-coding RNA.; ubi	-18.68
Gm12159	predicted gene 12159	2.36
Gm7502	predicted gene 7502 [Source:MGI Symbol;Acc:MGI:36	-2.36
Gm14630	predicted gene 14630 [Source:MGI Symbol;Acc:MGI:3	-2.21
Gm24139	predicted gene, 24139	-2
Higd2a	HIG1 domain family, member 2A	-4.49
Ube2k	ubiquitin-conjugating enzyme E2K	-2.01
Gm17268	predicted gene, 17268 [Source:MGI Symbol;Acc:MGI:	2.49
Gm26983	predicted gene, 26983 [Source:MGI Symbol;Acc:MGI:	-2.22
		2.07
Gm11942	predicted gene 11942 [Source:MGI Symbol;Acc:MGI:3	-17.52
Gm8927	predicted gene 8927 [Source:MGI Symbol;Acc:MGI:36	-2.94
		-2.05
Mrps36-ps1	mitichondrial ribosomal protein S36, pseudogene 1 [So	-5.97
Trappc11	trafficking protein particle complex 11	-2.69
Abhd3	abhydrolase domain containing 3	-2.97
Rpl30-ps11	ribosomal protein L30, pseudogene 11	-5.42
Layn	layilin	-3.24
Gm19475	PREDICTED: predicted gene, 19475 (Gm19475), mR	-3.6
Mir684-2	microRNA 684-2; microRNA 684-2 (Mir684-2), micro	-122.24

Rpl31-ps10	ribosomal protein L31, pseudogene 10	-4.82
Rpl15-ps2	ribosomal protein L15, pseudogene 2	-5.27
Gm25839	predicted gene, 25839 [Source:MGI Symbol;Acc:MGI:	2.77
Gm13436	predicted gene 13436	-2.5
Mir142	microRNA 142	2.52
Pomp	proteasome maturation protein	-12.72
Rnaset2a; Rnaset2b	ribonuclease T2A; ribonuclease T2B (Rnaset2b), mRN	-4.81
Gm22173	predicted gene, 22173 [Source:MGI Symbol;Acc:MGI:	-32.86
Gm24691	predicted gene, 24691 [Source:MGI Symbol;Acc:MGI:	2.05
Dstn	destrin	-3.47
Gm10540	predicted gene 10540 [Source:MGI Symbol;Acc:MGI:3	-4.16
Pcnp	PEST proteolytic signal containing nuclear protein	-3.24
Gm20430	predicted gene 20430 [Source:MGI Symbol;Acc:MGI:5	-60.67
Gm10913	predicted pseudogene 10913	-4.21
		-3.3
Gm10343	predicted gene 10343 [Source:MGI Symbol;Acc:MGI:3	-6.26
Polb	polymerase (DNA directed), beta	-2.73
Gm15961; RP23-455J6.	predicted gene 15961 [Source:MGI Symbol;Acc:MGI:3	-2.56
Eif4h	eukaryotic translation initiation factor 4H	-4.68
Ctnnb1; Mir7090	catenin (cadherin associated protein), beta 1; microRN	-2.54
Gm9174	predicted pseudogene 9174 [Source:MGI Symbol;Acc:	-5.44
Rpl9-ps4	ribosomal protein L9, pseudogene 4 [Source:MGI Sym	-16.48
Gm27459	predicted gene, 27459 [Source:MGI Symbol;Acc:MGI:	-6.46
Sparc	secreted acidic cysteine rich glycoprotein	-2.1
Gm6747	PREDICTED: predicted gene 6747, transcript variant	-9.87
Hagh	hydroxyacyl glutathione hydrolase	-2.01
Rpl9-ps7	ribosomal protein L9, pseudogene 7	-9.88
Gm12704	predicted gene 12704 [Source:MGI Symbol;Acc:MGI:3	-2.25
		2.75
		-2.23
		-2.62
Rpl19-ps4; Gm27506	ribosomal protein L19, pseudogene 4 [Source:MGI Sy	-5.27
Gm8062	predicted pseudogene 8062 [Source:MGI Symbol;Acc:	-3.26
Ephx1	epoxide hydrolase 1, microsomal	-3.1
		-2.3
Grin3a	glutamate receptor ionotropic, NMDA3A; glutamate re	-2.21
		2.01
Gm26502	predicted gene, 26502 [Source:MGI Symbol;Acc:MGI:	-36.66
Gm23313	predicted gene, 23313 [Source:MGI Symbol;Acc:MGI:	-36.66
Gm23446	predicted gene, 23446 [Source:MGI Symbol;Acc:MGI:	-36.66
Gm20094	PREDICTED: predicted gene, 20094 (Gm20094), mR	-8.46
		-4.75
Cpt1a	carnitine palmitoyltransferase 1a, liver	-3.3
Rpl27-ps3	ribosomal protein L27, pseudogene 3 [Source:MGI Sy	-24.31

Rpl19	ribosomal protein L19	-3.91
Atxn10	ataxin 10	-2.67
Mir1195	microRNA 1195	-3.59
Gm25407	predicted gene, 25407 [Source:MGI Symbol;Acc:MGI:	2.14
		2.21
Gm10039	predicted pseudogene 10039	-6.32
Tdrd3	tudor domain containing 3	-2.52
Gmps	guanine monophosphate synthetase; guanine monphosp	-2.15
Gm10217	predicted gene 10217 [Source:MGI Symbol;Acc:MGI:	-6.62
Rps18-ps3	ribosomal protein S18, pseudogene 3 [Source:MGI Sy	-7.17
LOC100862257	PREDICTED: uncharacterized LOC100862257 (LOC	-3.9
Gm5481	PREDICTED: predicted gene 5481 (Gm5481), mRNA	-15.98
Utrn	utrophin	-2.91
		-3.83
Gm22358	predicted gene, 22358 [Source:MGI Symbol;Acc:MGI:	-9.84
		2.75
Gm19353	predicted gene, 19353 [Source:MGI Symbol;Acc:MGI:	-2.92
Mettl5	methyltransferase like 5	-2.31
Gm26032	predicted gene, 26032 [Source:MGI Symbol;Acc:MGI:	-21.9
Gm22046	predicted gene, 22046 [Source:MGI Symbol;Acc:MGI:	-21.9
Gm25471	predicted gene, 25471 [Source:MGI Symbol;Acc:MGI:	-21.9
Gm25210	predicted gene, 25210 [Source:MGI Symbol;Acc:MGI:	-21.9
m21811; 2610005L07R	predicted gene, 21811; cadherin 11 pseudogene	-3.25
		2.44
		2.14
Gm23487	predicted gene, 23487	-4.05
Gm24588	predicted gene, 24588 [Source:MGI Symbol;Acc:MGI:	-2.83
Gm25233	predicted gene, 25233	-2.18
Gm5786	predicted pseudogene 5786 [Source:MGI Symbol;Acc:	-7.48
Gm10254	predicted gene 10254 [Source:MGI Symbol;Acc:MGI:	-2.11
Gm5457	predicted pseudogene 5457 [Source:MGI Symbol;Acc:	-16.89
Hbb-bt; Hbb-b2; Hbb-b1	hemoglobin, beta adult t chain; hemoglobin, beta adult	-2.53
		2.08
Hist1h2al	histone cluster 1, H2al	-2.48
Gm25350	predicted gene, 25350 [Source:MGI Symbol;Acc:MGI:	-22.19
Gm22128	predicted gene, 22128 [Source:MGI Symbol;Acc:MGI:	-22.19
Gm26246	predicted gene, 26246 [Source:MGI Symbol;Acc:MGI:	-22.19
Gm25597	predicted gene, 25597 [Source:MGI Symbol;Acc:MGI:	-22.19
Gm26201	predicted gene, 26201 [Source:MGI Symbol;Acc:MGI:	-22.19
Gm22812	predicted gene, 22812 [Source:MGI Symbol;Acc:MGI:	-22.19
Gm26433	predicted gene, 26433 [Source:MGI Symbol;Acc:MGI:	-22.19
Gm23357	predicted gene, 23357 [Source:MGI Symbol;Acc:MGI:	-22.19
Gm22110	predicted gene, 22110 [Source:MGI Symbol;Acc:MGI:	-22.19
Gm23724	predicted gene, 23724 [Source:MGI Symbol;Acc:MGI:	-22.19

Gm23549	predicted gene, 23549 [Source:MGI Symbol;Acc:MGI:	-22.19
Gm26223	predicted gene, 26223 [Source:MGI Symbol;Acc:MGI:	-22.19
Gm23524	predicted gene, 23524 [Source:MGI Symbol;Acc:MGI:	-22.19
Gm26136	predicted gene, 26136 [Source:MGI Symbol;Acc:MGI:	-22.19
Gm24658	predicted gene, 24658 [Source:MGI Symbol;Acc:MGI:	-22.19
Gm23359	predicted gene, 23359 [Source:MGI Symbol;Acc:MGI:	-22.19
Gm24742	predicted gene, 24742 [Source:MGI Symbol;Acc:MGI:	-22.19
Gm23696	predicted gene, 23696 [Source:MGI Symbol;Acc:MGI:	-22.19
Gm26094	predicted gene, 26094 [Source:MGI Symbol;Acc:MGI:	-22.19
Gm11425	predicted gene 11425	-5.05
Gm23511	predicted gene, 23511 [Source:MGI Symbol;Acc:MGI:	-4.04
		-18.35
Gm14431; Gm8898	predicted gene 14431 (Gm14431), transcript variant 2,	-2.6
14431; Gm8898; Gm42	predicted gene 14431; predicted gene 8898 (Gm8898),	-2.6
Rpl31-ps13	ribosomal protein L31, pseudogene 13	-4.27
Gm8662	predicted gene 8662 [Source:MGI Symbol;Acc:MGI:36	-6.22
Gm23141	predicted gene, 23141 [Source:MGI Symbol;Acc:MGI:	-3.13
Rps12-ps21	ribosomal protein S12, pseudogene 22	-3.56
Rps2-ps6	ribosomal protein S2, pseudogene 6 [Source:MGI Sym	-13.42
Olfr145	olfactory receptor 145	2.01
Gm12396	predicted gene 12396	-6.44
		2.36
		-4.36
Gm26414	predicted gene, 26414	2.17
Gm23119	predicted gene, 23119 [Source:MGI Symbol;Acc:MGI:	-5.54
Eif3d	eukaryotic translation initiation factor 3, subunit D	-4.27
Gm4294	predicted gene 4294 [Source:MGI Symbol;Acc:MGI:37	-5.53
Gm2004	predicted gene 2004	-4.71
Gm13680	predicted gene 13680 [Source:MGI Symbol;Acc:MGI:3	-14.05
Gm11510	predicted gene 11510 [Source:MGI Symbol;Acc:MGI:3	-4.24
Pgrmc1	progesterone receptor membrane component 1	-8.77
Gm12460	predicted gene 12460 [Source:MGI Symbol;Acc:MGI:3	-2.14
Gm14411	predicted gene 14411	-4.43
Gkn3	gastrokine 3	-2.29
Prelid1	PRELI domain containing 1	-2.28
		3.84
Gm15779	predicted gene 15779 [Source:MGI Symbol;Acc:MGI:3	-2.9
		-2.67
Gm25074	predicted gene, 25074 [Source:MGI Symbol;Acc:MGI:	-22.79
Gm23308	predicted gene, 23308 [Source:MGI Symbol;Acc:MGI:	2.47
Atp1b1	ATPase, Na+/K+ transporting, beta 1 polypeptide	-2.76
Ndufa2	NADH dehydrogenase (ubiquinone) 1 alpha subcomple	-13.19
Eif1-ps1	eukaryotic translation initiation factor 1, pseudogene 1	-10.68
Gm14391; Gm6710	predicted gene 14391 (Gm14391), transcript variant 1,	-2.7

PRINS	PRINS RNA	2.23
Fis1	fission 1 (mitochondrial outer membrane) homolog (ye	-3.2
Gatm	glycine amidinotransferase (L-arginine:glycine amidino	-2.69
Gm13094	predicted gene 13094 [Source:MGI Symbol;Acc:MGI:3	-11.24
Gatad1	GATA zinc finger domain containing 1	-2.21
Gm5453	predicted gene 5453	-3.35
Kcna1	potassium voltage-gated channel, shaker-related subfam	-3.12
116l1; Snord116l2; Sno	small nucleolar RNA, C/D box 116-like 1 (Snord116l1	-27.05
116l1; Snord116l2; Sno	small nucleolar RNA, C/D box 116-like 1 (Snord116l1	-27.05
116l1; Snord116l2; Sno	small nucleolar RNA, C/D box 116-like 1 (Snord116l1	-27.05
116l1; Snord116l2; Sno	small nucleolar RNA, C/D box 116-like 1 (Snord116l1	-27.05
116l1; Snord116l2; Sno	small nucleolar RNA, C/D box 116-like 1 (Snord116l1	-27.05
116l1; Snord116l2; Sno	small nucleolar RNA, C/D box 116-like 1 (Snord116l1	-27.05
116l1; Snord116l2; Sno	small nucleolar RNA, C/D box 116-like 1 (Snord116l1	-27.05
116l1; Snord116l2; Sno	small nucleolar RNA, C/D box 116-like 1; small nucle	-27.05
116l1; Snord116l2; Sno	small nucleolar RNA, C/D box 116-like 1 (Snord116l1	-27.05
Snord116l2	small nucleolar RNA, C/D box 116-like 2	-27.05
Snord116l1	small nucleolar RNA, C/D box 116-like 1	-27.05
		-2.86
Rny3	RNA, Y3 small cytoplasmic (associated with Ro protei	-65.75
Rny3	RNA, Y3 small cytoplasmic (associated with Ro protei	-65.75
Gm25474	predicted gene, 25474 [Source:MGI Symbol;Acc:MGI:	-7.06
Gm22047	predicted gene, 22047 [Source:MGI Symbol;Acc:MGI:	-7.06
Gm23619	predicted gene, 23619 [Source:MGI Symbol;Acc:MGI:	-7.06
Cdc42	cell division cycle 42	-3.46
Gm11628	predicted gene 11628 [Source:MGI Symbol;Acc:MGI:3	-2.74
Gm15795	predicted gene 15795 [Source:MGI Symbol;Acc:MGI:3	-6.99
Gm11625	predicted gene 11625 [Source:MGI Symbol;Acc:MGI:3	-3.59
		-12.77
		-12.77
		-12.77
		-12.77
		-12.77
Gm19496	PREDICTED: predicted gene, 19496 (Gm19496), mis	-6.31
Hist1h1c	histone cluster 1, H1c	-2.59
		-25.5
		-25.5
Ywhae	tyrosine 3-monooxygenase/tryptophan 5-monooxygena	-2.25
Pias1	protein inhibitor of activated STAT 1	-5.78
Gm14407	predicted gene 14407	-2.12
		2.04
Smt3h2-ps	SMT3 suppressor of mif two 3 homolog 2, pseudogene	-16.16
Ddhd1; Mir5131	DDHD domain containing 1; microRNA 5131; DDHD	-2.05
Gm17257	predicted gene, 17257 [Source:MGI Symbol;Acc:MGI:	-2.01

Snora30	small nucleolar RNA, H/ACA box 30	-6.12
		-4.39
s12-ps22; LOC1010557	ribosomal protein S12, pseudogene 22 [Source:MGI Sy	-3
Gm25355	predicted gene, 25355 [Source:MGI Symbol;Acc:MGI:	-2.25
Gm23064	predicted gene, 23064 [Source:MGI Symbol;Acc:MGI:	-2.25
Gm23631	predicted gene, 23631 [Source:MGI Symbol;Acc:MGI:	-3.23
Atp5l2-ps	ATP synthase, H+ transporting, mitochondrial FO com	-3.45
Gm22701	predicted gene, 22701	-2.14
Gm16380	predicted pseudogene 16380 [Source:MGI Symbol;Acc	-2.34
Gm13137	predicted gene 13137 [Source:MGI Symbol;Acc:MGI:3	-2.63
Gm19933	PREDICTED: predicted gene, 19933, transcript varian	-4.2
Gm25986	predicted gene, 25986 [Source:MGI Symbol;Acc:MGI:	-2.06
Smt3h2-ps2	SMT3 suppressor of mif two 3 homolog 2, pseudogene	-8.48
Gm7117; Gm10275	predicted pseudogene 7117 [Source:MGI Symbol;Acc:	-18.42
Slc38a11	solute carrier family 38, member 11	-2.6
		2.5
Gm14287	predicted gene 14287 [Source:MGI Symbol;Acc:MGI:3	-5.17
		-2.11
Txn1	thioredoxin 1	-2.06
Gm8444	predicted gene 8444 [Source:MGI Symbol;Acc:MGI:36	-2.01
Mir297-1	microRNA 297-1	-14.89
Gm14325	predicted gene 14325	-3.82
		-2.13
Gm22852	predicted gene, 22852 [Source:MGI Symbol;Acc:MGI:	-2.47
Gm27248	predicted gene 27248 [Source:MGI Symbol;Acc:MGI:5	-2.48
Slc25a11	solute carrier family 25 (mitochondrial carrier oxogluta	-2.28
Gm6576	predicted gene 6576 [Source:MGI Symbol;Acc:MGI:36	-4.36
Rps15a-ps5	ribosomal protein S15A, pseudogene 5	-2.01
Tmem50b	transmembrane protein 50B	-5.37
Atp5l-ps1	ATP synthase, H+ transporting, mitochondrial F0 comp	-3.57
		2.77
		-4.55
		2.64
9430002A10Rik	RIKEN cDNA 9430002A10 gene	-3.07
		2.23
Gm23876	predicted gene, 23876 [Source:MGI Symbol;Acc:MGI:	-3.41
		2.32
Sep15	selenoprotein	-2.43
Pdhb	pyruvate dehydrogenase (lipoamide) beta	-2.41
Gm22188	predicted gene, 22188 [Source:MGI Symbol;Acc:MGI:	-20.29
		-2.29
Naa38	N(alpha)-acetyltransferase 38, NatC auxiliary subunit	-2.29
Gm12482	predicted gene 12482 [Source:MGI Symbol;Acc:MGI:3	-2.12
Rpl31-ps7	ribosomal protein L31, pseudogene 7	-2.24

Vkorc1l1	vitamin K epoxide reductase complex, subunit 1-like 1	-3.6
Gm25188	predicted gene, 25188 [Source:MGI Symbol;Acc:MGI:	-12.16
Gm14388	predicted gene 14388 [Source:MGI Symbol;Acc:MGI:3	-2.33
Dhx9	DEAH (Asp-Glu-Ala-His) box polypeptide 9	-2.46
Atp5h	ATP synthase, H+ transporting, mitochondrial F0 comp	-2.1
		-163.45
Gm6863	predicted gene 6863 [Source:MGI Symbol;Acc:MGI:36	-4.86
Pik3r3	phosphatidylinositol 3 kinase, regulatory subunit, polyp	-2.61
Gm7286	predicted gene 7286 [Source:MGI Symbol;Acc:MGI:36	-2.59
Gm9159	predicted gene 9159 [Source:MGI Symbol;Acc:MGI:36	-2.58
		2.04
Gm4596	predicted gene 4596 [Source:MGI Symbol;Acc:MGI:37	-2.16
		-3.39
Gm14295	predicted gene 14295	-4.67
Gm13202	predicted gene 13202	-5.79
		2.78
Gm5139	predicted gene 5139 [Source:MGI Symbol;Acc:MGI:37	-2.71
		-4.9
Gm22751; Gm12164	predicted gene, 22751 [Source:MGI Symbol;Acc:MGI:	-2.72
Cltc	clathrin, heavy polypeptide (Hc)	-3.87
n-R5s158	nuclear encoded rRNA 5S 158 [Source:MGI Symbol;A	2.09
Rpl19-ps11	ribosomal protein L19, pseudogene 11	-14.96
Fads2	fatty acid desaturase 2	-3.83
Gm12715	predicted gene 12715	-2.26
Ubb; Gm1821	ubiquitin B; ubiquitin pseudogene	-7.92
		-118.95
Gm15393	predicted gene 15393 [Source:MGI Symbol;Acc:MGI:3	-3.6
Gm9703	predicted gene 9703 [Source:MGI Symbol;Acc:MGI:37	-8.55
Rpl28-ps3	ribosomal protein L28, pseudogene 3	-3.76
Gm11450	predicted gene 11450 [Source:MGI Symbol;Acc:MGI:3	-26.48
Qk	quaking	-3.41
Gm16418	predicted pseudogene 16418 [Source:MGI Symbol;Acc	-3.74
Gm5561	predicted gene 5561 [Source:MGI Symbol;Acc:MGI:38	-3.82
Gm25722	predicted gene, 25722	2.18
		2.01
Pten	phosphatase and tensin homolog	-3.28
Rps12-ps19	ribosomal protein S12, pseudogene 19 [Source:MGI Sy	-7.2
Gm7206	predicted pseudogene 7206 [Source:MGI Symbol;Acc:	-6.5
Gm15745	predicted gene 15745 [Source:MGI Symbol;Acc:MGI:3	-2.95
		-2.47
		-4.26
Rps6-ps4	ribosomal protein S6, pseudogene 4	-75.19
Csde1	cold shock domain containing E1, RNA binding	-2.36
Gm17511	predicted gene, 17511	-3.16

Gm26097	predicted gene, 26097 [Source:MGI Symbol;Acc:MGI:	-4.78
Gm10051	predicted pseudogene 10051	-3.04
Rabac1	Rab acceptor 1 (prenylated)	-3.69
Gm4184	PREDICTED: predicted gene 4184 (Gm4184), mRNA	-4.77
Drg1	developmentally regulated GTP binding protein 1	-7.94
Gm22117	predicted gene, 22117 [Source:MGI Symbol;Acc:MGI:	-2.51
Gm27731	predicted gene, 27731 [Source:MGI Symbol;Acc:MGI:	-2.92
Rnf169	ring finger protein 169	-2.17
Gm8129	predicted pseudogene 8129	-5.7
Gm8648	predicted gene 8648 [Source:MGI Symbol;Acc:MGI:36	-5.85
Gm10576	predicted gene 10576	-4.91
Gm5210	predicted gene 5210 [Source:MGI Symbol;Acc:MGI:36	-2.56
Ptp4a2	protein tyrosine phosphatase 4a2	-2.66
Gm14436; Gm8923	predicted gene 14436; predicted gene 8923	-2.14
Snrpd3	small nuclear ribonucleoprotein D3	-3.65
		-2.84
Mbip	MAP3K12 binding inhibitory protein 1	-2.46
Rps8-ps1	ribosomal protein S8, pseudogene 1 [Source:MGI Sym	-5.94
Pllp	plasma membrane proteolipid	-2.04
Rpl14-ps1	ribosomal protein L14, pseudogene 1	-3.81
Gm17383	predicted gene, 17383 [Source:MGI Symbol;Acc:MGI:	-8.95
Mpzl1	myelin protein zero-like 1	-2.24
Gm14420	predicted gene 14420	-2.19
434; 0610010B08Rik; G	predicted gene 14308 (Gm14308), mRNA.; predicted g	-2.35
Bloc1s1	biogenesis of lysosomal organelles complex-1, subunit	-2.87
		-2.04
		2.02
		-2.16
		-2.2
Itfg1	integrin alpha FG-GAP repeat containing 1	-4.83
Atp6ap1	ATPase, H+ transporting, lysosomal accessory protein	-2.84
LOC100861804	PREDICTED: uncharacterized LOC100861804, transc	-2.44
Gm13750	predicted gene 13750 [Source:MGI Symbol;Acc:MGI:3	-2.72
Irf2bp2	interferon regulatory factor 2 binding protein 2	-2.12
Rpl30-ps5	ribosomal protein L30, pseudogene 5	-2.36
Gm13249	predicted gene 13249	-2.61
Gm15900	predicted gene 15900 [Source:MGI Symbol;Acc:MGI:3	-2.59
Fam107a	family with sequence similarity 107, member A; family	-3.82
Gpr37l1	G protein-coupled receptor 37-like 1	-2.02
Gm7936	predicted pseudogene 7936 [Source:MGI Symbol;Acc:	-3.1
Cox6b1	cytochrome c oxidase, subunit VIb polypeptide 1	-2.35
Cnot4	CCR4-NOT transcription complex, subunit 4	-2.37
		-3.68
Hnrnpa3; Gm6793	heterogeneous nuclear ribonucleoprotein A3; heterogen	-4.95

Gm20563	predicted gene, 20563 [Source:MGI Symbol;Acc:MGI:	-3.28
Gm5908	predicted gene 5908	-4.24
Rps19-ps9	ribosomal protein S19, pseudogene 9	-3.33
		26.99
Rps12-ps20	ribosomal protein S12, pseudogene 20	-3.33
Gm22527	predicted gene, 22527 [Source:MGI Symbol;Acc:MGI:	3.25
Gm14393	predicted gene 14393	-3.62
		-2.54
		-2.45
Rpl22l1	ribosomal protein L22 like 1	-6.78
Gpcpd1	glycerophosphocholine phosphodiesterase GDE1 homo	-3.8
		-4.11
Gm11808	predicted gene 11808 [Source:MGI Symbol;Acc:MGI:3	-3.97
Tceb1	transcription elongation factor B (SIII), polypeptide 1	-5.53
		-4.3
Gm19295	PREDICTED: predicted gene, 19295 (Gm19295), mis	-5.86
Gm23785	predicted gene, 23785 [Source:MGI Symbol;Acc:MGI:	-2.08
Gm26411	predicted gene, 26411 [Source:MGI Symbol;Acc:MGI:	2.03
		-2.39
Tgoln1; Tgoln2	trans-golgi network protein; trans-golgi network protei	-4.63
Scarna9	small Cajal body-specific RNA 9	-2.93
Mirlet7a-2	microRNA let7a-2	2.76
Gm15459	predicted gene 15459	-3.12
Gm15796	predicted gene 15796 [Source:MGI Symbol;Acc:MGI:3	-3.59
430; Gm14434; 061001	predicted gene 14308 (Gm14308), mRNA.; predicted g	-6.23
Gm15481	predicted gene 15481 [Source:MGI Symbol;Acc:MGI:3	-3.25
Gm13394	predicted gene 13394	-6.41
Gm23260	predicted gene, 23260 [Source:MGI Symbol;Acc:MGI:	2.41
Rnf115	ring finger protein 115	-2.03
Epas1	endothelial PAS domain protein 1	-5.88
Gm22802	predicted gene, 22802 [Source:MGI Symbol;Acc:MGI:	-3.16
		-4.23
Gm16089	predicted gene 16089 [Source:MGI Symbol;Acc:MGI:3	-3.74
Gm20077	PREDICTED: predicted gene, 20077 (Gm20077), mis	-6.79
Gm20545	predicted gene 20545 [Source:MGI Symbol;Acc:MGI:5	-3.65
Gm25635	predicted gene, 25635 [Source:MGI Symbol;Acc:MGI:	-3.88
Gm23458	predicted gene, 23458 [Source:MGI Symbol;Acc:MGI:	-3.79
Gm10705	predicted gene 10705	-8
Gm5879	predicted gene 5879	-3.81
Gm12755	predicted gene 12755 [Source:MGI Symbol;Acc:MGI:3	-12.31
Gm5805	predicted gene 5805 [Source:MGI Symbol;Acc:MGI:36	-3.32
Gm12669	predicted gene 12669	-3.62
		-6.82
		2.52

		2.52
		2.52
		2.52
		2.52
		2.52
		2.52
		2.52
		2.52
		2.52
		2.52
		2.52
		2.52
		2.52
		2.52
		2.52
		2.52
		2.52
Gm6768	predicted gene 6768 [Source:MGI Symbol;Acc:MGI:3	-6.07
		-7.26
Arl6ip1	ADP-ribosylation factor-like 6 interacting protein 1	-6.69
Rps25-ps1	ribosomal protein S25, pseudogene 1	-4.44
Dph3b-ps	DPH3B, KTI11 homolog B (S. cerevisiae), pseudogene	2.06
Cyc1	cytochrome c-1	-2.94
Lum	lumican	-2.41
Psme2b	protease (prosome, macropain) activator subunit 2B	-3.12
		2.23
Cycs	cytochrome c, somatic	-2.22
Fabp7	fatty acid binding protein 7, brain	-5.04
Gm7308	predicted pseudogene 7308 [Source:MGI Symbol;Acc:	-7.39
Tmem184c	transmembrane protein 184C	-2.28
Psmd11	proteasome (prosome, macropain) 26S subunit, non-AT	-2.08
Gm26905	predicted gene, 26905 [Source:MGI Symbol;Acc:MGI:	-6.93
Gm11281	predicted gene 11281	-2.78
Gm29376	predicted gene 29376	-4.98
		-3.33
		-5.23
Mir3475	microRNA 3475	3.04
Prpf38a	PRP38 pre-mRNA processing factor 38 (yeast) domain	-2.09
Srp54a	signal recognition particle 54A	-4.43
Gm23322	predicted gene, 23322 [Source:MGI Symbol;Acc:MGI:	2.64
Uqcrq	ubiquinol-cytochrome c reductase, complex III subunit	-5.61
Mir466j	microRNA 466j [Source:MGI Symbol;Acc:MGI:37833	-3.66
Uqcr11	ubiquinol-cytochrome c reductase, complex III subunit	-3.18
		-3.05

432; Gm14434; 061001	predicted gene 14308; predicted gene 14430; predicted	-2.16
Pcdh7	protocadherin 7	-3.8
1110032A03Rik	RIKEN cDNA 1110032A03 gene	-2.07
Gm5559	predicted gene 5559	-3.08
		-3.28
Gm14409	predicted gene 14409 [Source:MGI Symbol;Acc:MGI:3	-4.95
Sepw1	selenoprotein W, muscle 1	-3.11
Gm12033	predicted gene 12033	-2.87
Gm22776	predicted gene, 22776 [Source:MGI Symbol;Acc:MGI:	-12.48
		-5.52
Gm11196	predicted gene 11196 [Source:MGI Symbol;Acc:MGI:3	-2
Rpl14; Rpl14-ps1	ribosomal protein L14; ribosomal protein L14, pseudog	-2.69
Rbx1	ring-box 1	-2.78
Gm11511	predicted gene 11511 [Source:MGI Symbol;Acc:MGI:3	-2.52
DLG2-AS1_2	DLG2 antisense RNA 1 conserved region 2	2.41
Gm6374	predicted gene 6374	-2.22
Gm5138	predicted gene 5138 [Source:MGI Symbol;Acc:MGI:37	-4.91
m14434; 0610010B08R	predicted gene 14308 (Gm14308), mRNA.; predicted g	-3
Rab2a	RAB2A, member RAS oncogene family	-2.25
Gm2606	predicted pseudogene 2606 [Source:MGI Symbol;Acc:	-4.49
Sept7	septin 7	-2.29
Mrgpra2b	MAS-related GPR, member A2B	2.18
Rpl27a-ps1	ribosomal protein L27A, pseudogene 1 [Source:MGI S	-2.74
Gm4835	predicted pseudogene 4835 [Source:MGI Symbol;Acc:	-3.19
Gm22712	predicted gene, 22712	-8.71
Atp1b2	ATPase, Na+/K+ transporting, beta 2 polypeptide	-2.55
Cetn3	centrin 3	-4.16
Rpl30-ps1	ribosomal protein L30, pseudogene 1	-5.26
Gm12338	predicted gene 12338	-9.84
59; Gm17081; LOC1008	predicted pseudogene 5859 [Source:MGI Symbol;Acc:	-3.86
430; Gm14434; 0610010	predicted gene 14308 (Gm14308), mRNA.; predicted g	-3.64
Ndufb9	NADH dehydrogenase (ubiquinone) 1 beta subcomplex	-5.8
Rpl19-ps1	ribosomal protein L19, pseudogene 1	-10.25
Arrdc3	arrestin domain containing 3	-2.86
Mir6993	microRNA 6993	2.35
		17.33
Rpl31-ps22	ribosomal protein L31, pseudogene 22	-3.69
		-8.6
Rps13-ps1	ribosomal protein S13, pseudogene 1	-3.75
Gm7363	predicted gene 7363 [Source:MGI Symbol;Acc:MGI:36	-5.95
Apoo; Apoo-ps	apolipoprotein O; apolipoprotein O, pseudogene	-2.64
Gm6467	predicted gene 6467 [Source:MGI Symbol;Acc:MGI:36	-2.53
Gm5560	predicted pseudogene 5560	-4.41
		2.32

Rs5-8s1	5.8S ribosomal RNA	-3
		-2.85
Psma3	proteasome (prosome, macropain) subunit, alpha type 3	-6.17
Gm26231	predicted gene, 26231 [Source:MGI Symbol;Acc:MGI:	-4.45
Gm22234	predicted gene, 22234 [Source:MGI Symbol;Acc:MGI:	-4.45
Hist1h2bc	histone cluster 1, H2bc	-5.01
Gm12671	predicted gene 12671 [Source:MGI Symbol;Acc:MGI:3	-4.15
Morf4l1	mortality factor 4 like 1	-3.89
		-3.64
		-4.02
Gm3756	predicted gene 3756 [Source:MGI Symbol;Acc:MGI:37	-4.35
		-2.81
		-2.1
Arl5a	ADP-ribosylation factor-like 5A	-2.44
Vmn1r44	vomeronasal 1 receptor 44	2.22
		-7.05
Nae1	NEDD8 activating enzyme E1 subunit 1	-2.12
		-2.64
Rps19-ps7	ribosomal protein S19, pseudogene 7	-2.12
		-2.5
		-4.38
s12-ps23; LOC1008621	ribosomal protein S12, pseudogene 23 [Source:MGI Sy	-4.62
Rmst	rhabdomyosarcoma 2 associated transcript (non-coding	-2.13
Scai	suppressor of cancer cell invasion	-3.01
		-6.37
Tmem47	transmembrane protein 47	-2.9
Gm11688	predicted gene 11688 [Source:MGI Symbol;Acc:MGI:3	-2.55
Gm24008	predicted gene, 24008 [Source:MGI Symbol;Acc:MGI:	2.11
Gm14036	predicted gene 14036 [Source:MGI Symbol;Acc:MGI:3	-5.46
Rps27l	ribosomal protein S27-like	-2.87
Gm15151	predicted gene 15151 [Source:MGI Symbol;Acc:MGI:3	-26.72
Gm14946	predicted gene 14946 [Source:MGI Symbol;Acc:MGI:3	2.32
Psma2	proteasome (prosome, macropain) subunit, alpha type 2	-3.13
Usmg5	upregulated during skeletal muscle growth 5	-10.9
Tubb2a	tubulin, beta 2A class IIA	-3.19
		-12.86
Gm27450	predicted gene, 27450 [Source:MGI Symbol;Acc:MGI:	-2.31
		-3.86
Pts	6-pyruvoyl-tetrahydropterin synthase	-3.83
Rps18-ps1	ribosomal protein S18, pseudogene 1	-4.2
		-2.91
Gm16378	predicted gene 16378 [Source:MGI Symbol;Acc:MGI:3	-3.68
		-3.51
Gm25506	predicted gene, 25506 [Source:MGI Symbol;Acc:MGI:	-2.81

Wnk1	WNK lysine deficient protein kinase 1	-2.14
Serinc3	serine incorporator 3	-2.42
		-7.28
LOC100861852	PREDICTED: uncharacterized LOC100861852, transc	-2.57
Mtch1	mitochondrial carrier homolog 1 (C. elegans)	-2.38
		2.19
		-2.03
Gm12346	predicted gene 12346 [Source:MGI Symbol;Acc:MGI:3	-2.26
Snord92	small nucleolar RNA, C/D box 92	-2.65
n-R5s205	nuclear encoded rRNA 5S 205 [Source:MGI Symbol;A	2.21
Psenen	presenilin enhancer 2 homolog (C. elegans)	-3.79
		-2.67
		2.06
Rpl31-ps23	ribosomal protein L31, pseudogene 23	-2.53
Ndufa12	NADH dehydrogenase (ubiquinone) 1 alpha subcomple	-2.32
Rpl36a-ps3	ribosomal protein L36A, pseudogene 3	-2.42
Gm23880	predicted gene, 23880 [Source:MGI Symbol;Acc:MGI:	2.32
Nedd8	neural precursor cell expressed, developmentally down	-4.09
Vkorc1	vitamin K epoxide reductase complex, subunit 1	-6.85
Gm12335	predicted gene 12335 [Source:MGI Symbol;Acc:MGI:3	-18.92
Ostc	oligosaccharyltransferase complex subunit	-6.46
Gm8858	predicted gene 8858 [Source:MGI Symbol;Acc:MGI:36	-4.22
		2.17
Rtcb	RNA 2,3-cyclic phosphate and 5-OH ligase; RNA 2',3'-	-4.83
Gm27704	predicted gene, 27704 [Source:MGI Symbol;Acc:MGI:	-11.55
		-2.54
Gm5641	predicted gene 5641 [Source:MGI Symbol;Acc:MGI:36	-39.96
Gm22990	predicted gene, 22990 [Source:MGI Symbol;Acc:MGI:	-2.76
Gm3272	predicted pseudogene 3272	-4.16
Ndrg2	N-myc downstream regulated gene 2	-6.13
Ndufb11	NADH dehydrogenase (ubiquinone) 1 beta subcomplex	-5.74
Il6st	interleukin 6 signal transducer	-2.14
Gm7324	predicted gene 7324 [Source:MGI Symbol;Acc:MGI:36	-3.06
Gm15719	predicted gene 15719 [Source:MGI Symbol;Acc:MGI:3	-4.09
Laptm4a	lysosomal-associated protein transmembrane 4A	-3.48
		2.03
		-2.21
		2.1
Gm22297	predicted gene, 22297 [Source:MGI Symbol;Acc:MGI:	2.89
		2.3
Gm4459	predicted gene 4459 [Source:MGI Symbol;Acc:MGI:37	-2.01
		2.03
Hspe1	heat shock protein 1 (chaperonin 10)	-3.09
Snrpn; Snurf	small nuclear ribonucleoprotein N; SNRPN upstream r	-2.07

Actg-ps1; Gm23812	actin, gamma, pseudogene 1 [Source:MGI Symbol;Acc	-6.1
Rora	RAR-related orphan receptor alpha	-2.26
Gm23215	predicted gene, 23215 [Source:MGI Symbol;Acc:MGI:	-2.21
Gm14088	predicted gene 14088 [Source:MGI Symbol;Acc:MGI:3	-4.06
Gm15487; Atp6v0c	predicted gene 15487; predicted gene 15487 [Source:M	-3.04
Gm5944	predicted gene 5944 [Source:MGI Symbol;Acc:MGI:36	-2.66
		-4.24
Gm9844	predicted pseudogene 9844 [Source:MGI Symbol;Acc:	-2.4
Gm7808	predicted pseudogene 7808 [Source:MGI Symbol;Acc:	-2.38
Gm12960	predicted gene 12960	-2.21
Gm6344	predicted gene 6344 [Source:MGI Symbol;Acc:MGI:37	-5.73
		-4.84
LOC100862107	PREDICTED: uncharacterized LOC100862107 (LOC	-2.04
Gm22868	predicted gene, 22868 [Source:MGI Symbol;Acc:MGI:	2.18
Mir181d	microRNA 181d	2.83
		-44.21
Gm15920	predicted gene 15920 [Source:MGI Symbol;Acc:MGI:3	-9.37
Gm26463	predicted gene, 26463 [Source:MGI Symbol;Acc:MGI:	-2.72
Gm24975	predicted gene, 24975 [Source:MGI Symbol;Acc:MGI:	-2.72
Gm25100	predicted gene, 25100 [Source:MGI Symbol;Acc:MGI:	-2.72
Gm28019	predicted gene, 28019 [Source:MGI Symbol;Acc:MGI:	-2.72
		-2.72
Gm3617	predicted gene 3617 [Source:MGI Symbol;Acc:MGI:37	-6.77
Hnrnpab	heterogeneous nuclear ribonucleoprotein A/B	-2.35
Napb	N-ethylmaleimide sensitive fusion protein attachment p	-2.79
Zfp97	zinc finger protein 97; Synthetic construct Mus muscul	-2.13
		-2.04
Gm24463	predicted gene, 24463 [Source:MGI Symbol;Acc:MGI:	-5.25
Gm19868	PREDICTED: predicted gene, 19868 (Gm19868), mis	-11.73
		-2.7
Gm23316	predicted gene, 23316 [Source:MGI Symbol;Acc:MGI:	2.21
Gm22189	predicted gene, 22189 [Source:MGI Symbol;Acc:MGI:	-2.63
A130040M12Rik	RIKEN cDNA A130040M12 gene (A130040M12Rik),	-2.5
Rps19-ps5	ribosomal protein S19, pseudogene 5	-4.04
		-2.12
		2.04
Mpp6	membrane protein, palmitoylated 6 (MAGUK p55 subf	-2.32
Mir669e	microRNA 669e	-14.98
Ghitm	growth hormone inducible transmembrane protein	-4.39
Gm23992	predicted gene, 23992 [Source:MGI Symbol;Acc:MGI:	-3.49
		-2.02
Gm5426	predicted pseudogene 5426 [Source:MGI Symbol;Acc:	-2.61
Rpl38-ps2	ribosomal protein L38, pseudogene 2	-5
Gm10959	predicted gene 10959 [Source:MGI Symbol;Acc:MGI:3	-4.38

Gm15843	predicted gene 15843 [Source:MGI Symbol;Acc:MGI:3	-2.91
Gm26170	predicted gene, 26170 [Source:MGI Symbol;Acc:MGI:	2.24
		-2.42
Mir6388	microRNA 6388	2.19
Irs2	insulin receptor substrate 2	-3.69
Gm9836	predicted pseudogene 9836 [Source:MGI Symbol;Acc:	-16.73
		-2.4
Dram2	DNA-damage regulated autophagy modulator 2; VDNA	-2.6
Gm27989	predicted gene, 27989 [Source:MGI Symbol;Acc:MGI:	-4.61
Gm10180	predicted gene 10180 [Source:MGI Symbol;Acc:MGI:3	-4.83
Gm3699	predicted gene 3699 [Source:MGI Symbol;Acc:MGI:37	-4.83
Tes3-ps	testis derived transcript 3, pseudogene [Source:MGI Sy	-16.77
		2.16
Rps12-ps3	ribosomal protein S12, pseudogene 3 [Source:MGI Sym	-4.02
Gm2546	predicted gene 2546 [Source:MGI Symbol;Acc:MGI:37	-5.07
Amfr	autocrine motility factor receptor	-2.07
Ndfip1	Nedd4 family interacting protein 1	-2.49
		-29.16
		-4.59
		-2.32
Gm22510	predicted gene, 22510 [Source:MGI Symbol;Acc:MGI:	-3.06
Gm26390	predicted gene, 26390 [Source:MGI Symbol;Acc:MGI:	-3.06
Gm26336	predicted gene, 26336 [Source:MGI Symbol;Acc:MGI:	-3.06
Rpl31-ps15	ribosomal protein L31, pseudogene 15	-3.34
Gm7336	predicted gene 7336 [Source:MGI Symbol;Acc:MGI:36	-4.66
Rpl34-ps1	ribosomal protein L34, pseudogene 1	-2.75
Mir193b	microRNA 193b	2.04
Rcan1	regulator of calcineurin 1	-2.08
Gm10232	predicted pseudogene 10232	-2.04
Chmp2a	charged multivesicular body protein 2A	-2.47
Mir297a-2	microRNA 297a-2	-12.2
Mir153	microRNA 153	3.46
Cox7a2l	cytochrome c oxidase subunit VIIa polypeptide 2-like	-2.84
Gm22303	predicted gene, 22303 [Source:MGI Symbol;Acc:MGI:	-3.94
Gm10263	predicted gene 10263 [Source:MGI Symbol;Acc:MGI:3	-3.82
Rps8-ps4	ribosomal protein S8, pseudogene 4 [Source:MGI Sym	-6.63
		-2.05
Gm6382	predicted gene 6382 [Source:MGI Symbol;Acc:MGI:36	-2.28
Gm7589	predicted gene 7589	-2.53
		-5.31
Mbnl2	muscleblind-like 2	-2.64
Gm13162	predicted pseudogene 13162 [Source:MGI Symbol;Acc	-3.83
Gm11951	predicted gene 11951 [Source:MGI Symbol;Acc:MGI:3	-4.09
Gm13378	predicted gene 13378 [Source:MGI Symbol;Acc:MGI:3	-7.51

		-2.3
		-2.67
		-28.15
Srp54b; Srp54a	signal recognition particle 54B; signal recognition parti	-3.29
		-2.02
Ighv7-1	immunoglobulin heavy variable 7-1 [Source:MGI Sym	2.09
Gm23622	predicted gene, 23622 [Source:MGI Symbol;Acc:MGI:	2.23
Gm5239	predicted pseudogene 5239 [Source:MGI Symbol;Acc:	-2.01
Gm22501	predicted gene, 22501 [Source:MGI Symbol;Acc:MGI:	-2.39
Shfm1	split hand/foot malformation (ectrodactyly) type 1	-3.21
Gm20899; Gapdh-ps15	predicted gene, 20899; glyceraldehyde-3-phosphate deh	-4.2
Uqcrc2	ubiquinol cytochrome c reductase core protein 2	-2.59
Gm10443	predicted pseudogene 10443	-2.71
Gm17538	predicted gene, 17538 [Source:MGI Symbol;Acc:MGI:	-3.17
Tmem50a	transmembrane protein 50A	-5.17
		-12.82
		-12.82
Tuba4a	tubulin, alpha 4A	-3.14
Gm23700	predicted gene, 23700 [Source:MGI Symbol;Acc:MGI:	-2.03
Mir669a-4	microRNA 669a-4	-2.09
Mir669a-5	microRNA 669a-5	-2.09
Mir669a-6	microRNA 669a-6	-2.09
Mir669a-7	microRNA 669a-7	-2.09
Mir669a-8	microRNA 669a-8	-2.09
Mir669a-9	microRNA 4669a-9	-2.09
Mir669a-10	microRNA 669a-10	-2.09
Mir669a-11	microRNA 669a-11	-2.09
Mir669a-12	microRNA 669a-12	-2.09
Dync1h1	dynein cytoplasmic 1 heavy chain 1	-2.75
Ncam1	neural cell adhesion molecule 1	-2.56
LOC100862198	PREDICTED: uncharacterized LOC100862198 (LOC	-2.02
		-2.43
Gm27341	predicted gene, 27341 [Source:MGI Symbol;Acc:MGI:	-4.43
		-2.59
Gm24926	predicted gene, 24926 [Source:MGI Symbol;Acc:MGI:	-2.94
Gm22632	predicted gene, 22632 [Source:MGI Symbol;Acc:MGI:	-2.94
Adm	adrenomedullin	-2.24
Gm10182	predicted pseudogene 10182 [Source:MGI Symbol;Acc	-8.93
Gm23989	predicted gene, 23989 [Source:MGI Symbol;Acc:MGI:	-2.96
Gm8756	predicted gene 8756 [Source:MGI Symbol;Acc:MGI:3	-2.74
Slc25a5	solute carrier family 25 (mitochondrial carrier, adenine	-2.2
Gm13007	predicted gene 13007 [Source:MGI Symbol;Acc:MGI:	-2.67
Nell2	NEL-like 2	-2.19
LOC100861725	PREDICTED: uncharacterized LOC100861725 (LOC	-3.69

		2.82
Gm22252	predicted gene, 22252 [Source:MGI Symbol;Acc:MGI:	-2.05
		-5.14
Gm10420	predicted gene 10420	-3.97
Atp1a3	ATPase, Na+/K+ transporting, alpha 3 polypeptide (At	-2.04
Snora34; Mir1291	small nucleolar RNA, H/ACA box 34; microRNA 1291	-9.51
Gm5863	predicted gene 5863 [Source:MGI Symbol;Acc:MGI:36	-2.57
Gm13363	predicted gene 13363 [Source:MGI Symbol;Acc:MGI:3	-10.25
Gm14760	predicted gene 14760	-4.77
Pip4k2a	phosphatidylinositol-5-phosphate 4-kinase, type II, alph	-3.63
Phpt1	phosphohistidine phosphatase 1	-2.32
Olfr1424	olfactory receptor 1424	2.06
		-2.55
Gdi1	guanosine diphosphate (GDP) dissociation inhibitor 1	-2.39
		-2.1
Mir692-2	microRNA 692-2	-9.57
Dctn4	dynactin 4	-2.61
		2.01
Snord42a	small nucleolar RNA, C/D box 42A	-7.19
Gm27839	predicted gene, 27839 [Source:MGI Symbol;Acc:MGI:	-6.54
Mir6386	microRNA 6386	2.57
Eif4g2	eukaryotic translation initiation factor 4, gamma 2	-3.74
Fkbp7	FK506 binding protein 7	-2.18
Csnk1a1	casein kinase 1, alpha 1	-2.38
Gpbp1	GC-rich promoter binding protein 1	-3.23
Gm5582	predicted gene 5582 [Source:MGI Symbol;Acc:MGI:36	-9.48
Gm22230	predicted gene, 22230	-2.08
		-2.1
Odc1	ornithine decarboxylase, structural 1	-3.45
Gm2026	predicted gene 2026 [Source:MGI Symbol;Acc:MGI:37	-4.35
Gm9013	predicted gene 9013 [Source:MGI Symbol;Acc:MGI:36	-2.85
Atp6v0e	ATPase, H+ transporting, lysosomal V0 subunit E	-3.06
Gm11849	predicted gene 11849 [Source:MGI Symbol;Acc:MGI:3	-2.7
Gm12372	predicted gene 12372 [Source:MGI Symbol;Acc:MGI:3	-4.83
Gm24111	predicted gene, 24111 [Source:MGI Symbol;Acc:MGI:	2.01
Gm25759	predicted gene, 25759 [Source:MGI Symbol;Acc:MGI:	-2.59
		2.01
		-2.04
Gsk3b	glycogen synthase kinase 3 beta	-2.57
Gm24689	predicted gene, 24689 [Source:MGI Symbol;Acc:MGI:	2.02
Slc7a11	solute carrier family 7 (cationic amino acid transporter,	-4.5
Ap3d1	adaptor-related protein complex 3, delta 1 subunit	-2.08
Gm26202	predicted gene, 26202 [Source:MGI Symbol;Acc:MGI:	-10.66
Srp54b; Srp54c	signal recognition particle 54B (Srp54b), mRNA.; sign	-3.02

		-5.36
		-4.3
Gm9432	predicted gene 9432 [Source:MGI Symbol;Acc:MGI:36	-2.27
		-4.95
Rps13-ps4	ribosomal protein S13, pseudogene 4	-4.25
Gm4575	predicted gene 4575 [Source:MGI Symbol;Acc:MGI:37	-3.75
		-2.08
		2
Mir684-1; Gm13430	microRNA 684-1 (Mir684-1), microRNA.; predicted g	-12.92
		-3.41
Rpl30-ps8	ribosomal protein L30, pseudogene 8 [Source:MGI Sy	-4.74
		-2.44
Gm4518	predicted gene 4518 [Source:MGI Symbol;Acc:MGI:37	-2.89
Gm23374	predicted gene, 23374 [Source:MGI Symbol;Acc:MGI:	-16.71
Rps12-ps9	ribosomal protein S12, pseudogene 9	-5.56
		2.14
		-2.81
		-2.43
Gm19425	PREDICTED: predicted gene, 19425 (Gm19425), mis	-6.31
Gm10053	predicted gene 10053 [Source:MGI Symbol;Acc:MGI:3	-17.98
Fam174a	family with sequence similarity 174, member A	-2.03
		2.37
Rpl38	ribosomal protein L38	-3.7
Gm23164	predicted gene, 23164 [Source:MGI Symbol;Acc:MGI:	-2.28
Gm13413	predicted gene 13413 [Source:MGI Symbol;Acc:MGI:3	-3.24
		-2.35
		-2.6
		3.48
		15.71
Gm8355	predicted pseudogene 8355 [Source:MGI Symbol;Acc:	-9.17
Mir684-1; Gm10241	microRNA 684-1; predicted pseudogene 10241	-23.22
Fam168a	family with sequence similarity 168, member A	-2.18
Gm14853	predicted gene 14853 [Source:MGI Symbol;Acc:MGI:3	-4.4
Rps19-ps4	ribosomal protein S19, pseudogene 4	-4.7
Cadps	Ca2+-dependent secretion activator	-2.34
		-2.63
Gm10145	predicted gene 10145	-7.78
Wdr47	WD repeat domain 47	-2.06
		-8.1
Rpl30-ps10	ribosomal protein L30, pseudogene 10	-4.41
Gm27910	predicted gene, 27910 [Source:MGI Symbol;Acc:MGI:	-6.87
Rpl9-ps6	ribosomal protein L9, pseudogene 6 [Source:MGI Sym	-2.65
		-3.34
Gm14305	predicted gene 14305	-5.69

Rps19-ps8	ribosomal protein S19, pseudogene 8	-2.64
Atp1b3	ATPase, Na+/K+ transporting, beta 3 polypeptide	-4.19
Dirc2	disrupted in renal carcinoma 2 (human)	-2.45
Gm26358	predicted gene, 26358 [Source:MGI Symbol;Acc:MGI:	2.54
Mrpl50	mitochondrial ribosomal protein L50	-2.81
		-5.77
Mir669b	microRNA 669b	-4.63
		-2.3
Mir467c	microRNA 467c	-2.87
Gm23189	predicted gene, 23189 [Source:MGI Symbol;Acc:MGI:	-2.15
Gm25708	predicted gene, 25708 [Source:MGI Symbol;Acc:MGI:	-2.15
Gm25959	predicted gene, 25959 [Source:MGI Symbol;Acc:MGI:	-2.15
Gm24332	predicted gene, 24332 [Source:MGI Symbol;Acc:MGI:	-2.15
Gm23802	predicted gene, 23802 [Source:MGI Symbol;Acc:MGI:	-2.15
Gm22903	predicted gene, 22903 [Source:MGI Symbol;Acc:MGI:	-2.15
Gm25571	predicted gene, 25571 [Source:MGI Symbol;Acc:MGI:	-2.15
Gm23049	predicted gene, 23049 [Source:MGI Symbol;Acc:MGI:	-2.15
Gm24370	predicted gene, 24370 [Source:MGI Symbol;Acc:MGI:	-2.15
Gm24159	predicted gene, 24159 [Source:MGI Symbol;Acc:MGI:	-2.15
Gm25299	predicted gene, 25299 [Source:MGI Symbol;Acc:MGI:	-2.15
Gm23798	predicted gene, 23798 [Source:MGI Symbol;Acc:MGI:	-2.15
Gm25070	predicted gene, 25070 [Source:MGI Symbol;Acc:MGI:	-2.15
Gm22475	predicted gene, 22475 [Source:MGI Symbol;Acc:MGI:	-2.15
Gm23400	predicted gene, 23400 [Source:MGI Symbol;Acc:MGI:	-2.15
Gm27038	predicted gene, 27038 [Source:MGI Symbol;Acc:MGI:	-2.45
Cox8a	cytochrome c oxidase subunit VIIIa	-5.47
Gm2574	predicted pseudogene 2574	-3.8
Lrrc58	leucine rich repeat containing 58	-2.61
Gm12587	predicted gene 12587 [Source:MGI Symbol;Acc:MGI:[illegible]	-2.63
Mir7063	microRNA 7063	2.99
Cox5a	cytochrome c oxidase subunit Va	-4.79
Mir6916	microRNA 6916	2.06
Gm14017	predicted gene 14017 [Source:MGI Symbol;Acc:MGI:[illegible]	-3.25
Atp5l-ps2	ATP synthase, H+ transporting, mitochondrial F0 comp	-2.97
Scarna13; Mir3069	small Cajal body-specific RNA 1; microRNA 3069; mi	-2.33
Sdpr	serum deprivation response	-2.25
Gm6517	predicted gene 6517 [Source:MGI Symbol;Acc:MGI:3[illegible]	-3.51
Polr2k	polymerase (RNA) II (DNA directed) polypeptide K	-2.48
		-2.51
Tpt1-ps5	tumor protein, translationally-controlled, pseudogene 5	-2.49
Gm8783	predicted pseudogene 8783 [Source:MGI Symbol;Acc:	-13.55
Gm15801	predicted gene 15801 [Source:MGI Symbol;Acc:MGI:[illegible]	-13.55
Sacm1l	SAC1 (suppressor of actin mutations 1, homolog)-like	-2.04
		-5.88

Gm2962	predicted pseudogene 2962 [Source:MGI Symbol;Acc:	-34.01
Spcs2	signal peptidase complex subunit 2 homolog (S. cerevi	-3.85
Gm12912	predicted gene 12912 [Source:MGI Symbol;Acc:MGI:3	-2.08
Rpl38-ps1	ribosomal protein L38, pseudogene 1	-7.63
Gm16354	predicted gene 16354 [Source:MGI Symbol;Acc:MGI:3	-4.89
Gm12529	predicted gene 12529 [Source:MGI Symbol;Acc:MGI:3	-2.64
Gm14989	predicted gene 14989	-2.35
		2.25
		-38.84
Rplp1-ps1	ribosomal protein, large, P1, pseudogene 1	-2.92
Gm24541	predicted gene, 24541 [Source:MGI Symbol;Acc:MGI:	2.59
Gm21961	predicted gene, 21961 [Source:MGI Symbol;Acc:MGI:	-2.16
Gm14414	predicted gene 14414 [Source:MGI Symbol;Acc:MGI:3	-9.28
Gm7618	predicted pseudogene 7618 [Source:MGI Symbol;Acc:	-3.05
		-3.53
Elf1	E74-like factor 1; E74-like factor 1 (Elf1), mRNA.	-2.49
		-2.55
Glns-ps1	glutamine synthetase pseudogene 1	-4.93
Gm16470	predicted pseudogene 16470 [Source:MGI Symbol;Acc	-2.91
Aplp2	amyloid beta (A4) precursor-like protein 2	-2.13
Smap2	small ArfGAP 2	-3.19
Immp1l	IMP1 inner mitochondrial membrane peptidase-like (S.	-5.72
Mef2a	myocyte enhancer factor 2A	-3.65
Grm3	glutamate receptor, metabotropic 3	-3.4
		-2.62
Gm25224	predicted gene, 25224 [Source:MGI Symbol;Acc:MGI:	3.56
Gm7887	predicted gene 7887 [Source:MGI Symbol;Acc:MGI:36	-3.76
		-3.09
Smt3h2-ps4	SMT3 suppressor of mif two 3 homolog 2, pseudogene	-22.7
		-8.52
Gm14148	predicted gene 14148 [Source:MGI Symbol;Acc:MGI:3	-3.16
Gm15198	predicted gene 15198 [Source:MGI Symbol;Acc:MGI:3	-9.35
Mir1912	microRNA 1912	-23.61
Gm9753	PREDICTED: predicted gene 9753 (Gm9753), miscR	2.43
Sfxn1	sideroflexin 1	-3.16
		-3.2
1810022K09Rik	RIKEN cDNA 1810022K09 gene	-2.43
Gm4660	predicted gene 4660 [Source:MGI Symbol;Acc:MGI:37	-2.52
		10.35
		10.35
		10.35
		10.35
		10.35
820431F20Rik; Gm2109	RIKEN cDNA 6820431F20 gene, mRNA (cDNA clone	-2.2

Nutf2-ps2; Nutf2-ps1	nuclear transport factor 2, pseudogene 2; nuclear transp	-11.56
Gm12469	predicted gene 12469 [Source:MGI Symbol;Acc:MGI:	-4.39
		-2.42
		-2.78
Gm6316; EG622339	predicted pseudogene 6316 [Source:MGI Symbol;Acc:	-3.42
Rps16-ps2	ribosomal protein S16, pseudogene 2	-12.29
Snord17	small nucleolar RNA, C/D box 17	-2.02
Gm13352	predicted gene 13352	-4.95
Gm13728	predicted gene 13728 [Source:MGI Symbol;Acc:MGI:	2.08
Mapk1ip1l	mitogen-activated protein kinase 1 interacting protein 1	-2.26
Sf3b1	splicing factor 3b, subunit 1	-2.19
Gm13921	predicted gene 13921 [Source:MGI Symbol;Acc:MGI:	-2.08
		-2.02
Ranbp9	RAN binding protein 9	-3.92

Table S7: Differentially expressed genes with a significant interaction of diet and inhibitor.

Gene Symbol	LGD average intensity (log2)	LGD+sEHI average intensity (log2)	HGD average intensity (log2)	HGD+sEHI average intensity (log2)	Delta Fold Change
Gm24357	5.45	10.08	7.45	4.1	-251.56
Gm27529	9.53	11.81	12.6	7.52	-164.38
Gm22631	10.14	11.65	12.58	7.59	-90.4
Gm27684	5.15	7.61	7.77	3.86	-82.02
Gm22289	6.76	7.88	10.27	5.07	-79.42
Gm11450	7.07	8.54	10.54	5.81	-73.28
Rpl18-ps1	11.17	12.07	15.29	10.11	-67.96
Rpl19-ps11	7.58	9.7	10.78	6.87	-64.81
Gm25074	10.06	11.49	12.12	7.61	-61.18
Gm16409	11.38	11.48	14.48	8.67	-60.23
Gm8172	11.66	12.02	14.29	8.89	-54.17
Rps6-ps1	8.92	9.76	11.55	6.66	-53.1
Gm22759; Gm14251	14.1	16.78	17.81	14.79	-52.05
Gm8203	8.2	9.02	12.01	7.15	-51.16
Gm22188	10.31	11.64	11.85	7.51	-50.88
Gm23089	10.42	11.24	11.67	6.83	-50.73
Gm24711	10.42	11.24	11.67	6.83	-50.73
Gm26188	10.42	11.24	11.67	6.83	-50.73
Gm5558	10.25	10.64	12.69	7.42	-50.59
Gm23313	9.71	10.11	11.26	6.07	-48.12
Gm23446	9.71	10.11	11.26	6.07	-48.12
Gm26502	9.71	10.11	11.26	6.07	-48.12
Gm27626	12.04	11.89	15.49	9.77	-47.13
Gm22851	8.33	9.19	10.49	5.8	-46.98
Gm22760; Gm12318	11.23	14	15.07	12.3	-46.32
Gm23862	10.36	11.49	12.21	7.81	-46.28
Gm22110	10.77	11.77	12.06	7.59	-44.59
Gm22128	10.77	11.77	12.06	7.59	-44.59
Gm22812	10.77	11.77	12.06	7.59	-44.59
Gm23357	10.77	11.77	12.06	7.59	-44.59
Gm23359	10.77	11.77	12.06	7.59	-44.59
Gm23524	10.77	11.77	12.06	7.59	-44.59
Gm23549	10.77	11.77	12.06	7.59	-44.59
Gm23696	10.77	11.77	12.06	7.59	-44.59
Gm23724	10.77	11.77	12.06	7.59	-44.59
Gm24658	10.77	11.77	12.06	7.59	-44.59
Gm24742	10.77	11.77	12.06	7.59	-44.59

Gm25350	10.77	11.77	12.06	7.59	-44.59
Gm25597	10.77	11.77	12.06	7.59	-44.59
Gm26094	10.77	11.77	12.06	7.59	-44.59
Gm26136	10.77	11.77	12.06	7.59	-44.59
Gm26201	10.77	11.77	12.06	7.59	-44.59
Gm26223	10.77	11.77	12.06	7.59	-44.59
Gm26246	10.77	11.77	12.06	7.59	-44.59
Gm26433	10.77	11.77	12.06	7.59	-44.59
Gm22046	10.75	11.76	12.03	7.58	-44.11
Gm25210	10.75	11.76	12.03	7.58	-44.11
Gm25471	10.75	11.76	12.03	7.58	-44.11
Gm26032	10.75	11.76	12.03	7.58	-44.11
Gm22426	9.97	12.07	13.95	10.6	-43.64
Apod	9.4	9.44	12.21	6.82	-43.06
Rpl19-ps1	7.68	9.45	10.54	7.18	-34.98
Gm22131	6.39	7.76	7.97	4.24	-34.55
Mir682	14.49	16.48	17.7	14.58	-34.34
Gm25247	16.65	17.63	19.22	15.1	-34.28
Gm22584	10.51	11.22	11.12	6.74	-33.97
Gm17571	15.24	15.42	17.61	12.71	-33.88
Rps24-ps3	10.41	11.18	13.55	9.32	-31.99
Gm13680	6.97	8.15	10.55	6.74	-31.84
	7.94	9.83	11.34	8.24	-31.76
Gm23154	11.5	12.1	13.9	9.53	-31.16
Gm24264	9.6	10.49	11.04	6.97	-31.05
Gm1821	12.81	13.54	16.58	12.36	-30.84
	9.64	9.27	11.7	6.38	-30.81
Uck2	4.94	7.96	7.04	5.17	-29.5
Gm23767	8.91	9.69	9.69	5.62	-28.56
Gm2546	13.8	16.25	15.52	13.18	-27.72
Snord14e	3.33	4.03	7.38	3.29	-27.63
Gm20302; Gm22758; Gm14173	13.57	15.09	16.86	13.6	-27.5
Rpl18a-ps1	10.3	11.11	13.38	9.45	-26.76
Gm10157	5.24	6.83	8.31	5.15	-26.72
Gm15427	14.03	12.81	17.49	11.59	-25.65
Gm15621	12.74	12.35	14.43	9.42	-24.6
Hsbp1	7.68	10.93	10.52	9.16	-24.25
Rab7	9.29	9.65	11.97	7.73	-24.22
Mir1191	16.71	16.49	18.2	13.39	-24.1
Gm25188	3.59	4.52	7.06	3.46	-23.32
Rps4x	6.08	7.66	9.43	6.47	-23.27
	8.57	11.32	12.04	10.32	-22.27

Vdac3	6	7.48	9.04	6.06	-21.91
Cox7a2	10.45	10.89	14.11	10.12	-21.64
Gm22610	11.11	11	13.69	9.15	-21.52
	7.37	8.96	10.6	7.78	-21.32
Gm12755	7.06	7.78	9.4	5.78	-20.25
	4.18	5.02	7.59	4.11	-20.03
Higd2a	5.22	7.38	8.84	6.68	-20
Rpl4	10.34	10.83	14.16	10.33	-19.98
Gm6136	11.22	12.72	14.15	11.34	-19.88
H2afz	7.67	7.92	10.63	6.61	-19.28
Gm25594	6.21	8.03	8.96	6.56	-18.82
Mir6989	7.9	7.78	9.11	4.82	-17.95
Gm22757; Gm13875	10.47	12.82	13.18	11.38	-17.79
Rps6-ps3	11.63	11.31	13.01	8.58	-17.34
Eif1-ps1	6.12	6.82	8.79	5.37	-17.31
Gm22358	3.77	4.58	6.66	3.36	-17.19
Gm15720	12.49	12.44	12.95	8.82	-16.86
Gm11971	9.56	10.42	11.89	8.69	-16.75
Rpl8	8.7	9.64	11.32	8.2	-16.64
	3.87	7.06	5.31	4.45	-16.63
Gm25911	15.81	10.95	17.92	9.04	-16.19
	13.67	14.87	16.9	14.09	-16.12
Gm2389	5.44	6.36	7.28	4.24	-15.59
Hist1h2an	4.86	6.02	7.7	4.91	-15.46
Gm12328	9.99	10.77	12.68	9.54	-15.14
Gm4613	12.3	13.41	15.09	12.27	-15.13
Rpl38-ps1	10.09	11.07	12.22	9.29	-15.1
Gm27935	5.87	7.19	7.35	4.79	-14.73
Gm24400	4.3	4.02	8.58	4.43	-14.69
Gm4575	9.03	10.99	10.39	8.48	-14.58
Gm1840	8.24	8.5	10.04	6.44	-14.4
Gm19496	5.64	6.82	8.06	5.4	-14.29
Gm22582	7.08	8.34	10.16	7.59	-14.25
Gm6341	6.98	7.46	9.28	5.94	-14.14
Gm22863	8.69	9.59	9.49	6.6	-13.91
Gm23953	8.69	9.59	9.49	6.6	-13.91
Gm24618	8.69	9.59	9.49	6.6	-13.91
Gm26332	8.69	9.59	9.49	6.6	-13.91
Gm2308	10.28	12.27	11.45	9.65	-13.74
Gm22776	8.5	8.62	9.54	5.9	-13.58
Gm5138	12.2	13.67	13.17	10.88	-13.57
Ndufa2	8.42	8.44	12.33	8.61	-13.4
Gm25615	9.03	9.49	9.86	6.6	-13.2

Gm10959	7.33	8.9	8.59	6.46	-13.06
Gm9081	6.71	8.54	8.12	6.25	-13.05
Gm22047	9.61	10.48	10.17	7.35	-12.98
Gm23619	9.61	10.48	10.17	7.35	-12.98
Gm25474	9.61	10.48	10.17	7.35	-12.98
Gm10327	10.88	13.11	12.16	10.7	-12.9
Slc6a20a	6.9	8.3	8.07	5.8	-12.73
Scd2; Mir5114	7.28	8.44	9.4	6.88	-12.73
Gm12286	11.11	12.79	12.09	10.12	-12.57
Gm8349	10.2	12.42	11.42	10	-12.56
Vps28	5.46	7.07	7.57	5.53	-12.51
Gm12231	6.5	6.84	9.32	6.01	-12.49
Rpl27-ps1	7.74	8.04	9.77	6.45	-12.24
Ubb; Gm1821	11.24	11.87	13.45	10.46	-12.22
	5.31	6.24	8.06	5.4	-12.15
Hsp90ab1	9.97	10.97	11.28	8.68	-12.11
Gm10171	6.46	7.45	8.78	6.18	-12.05
Gm5514	11.97	12.11	13.85	10.4	-12.04
Gm25944	8.58	9.36	9.68	6.88	-11.99
Gm16470	10.99	13.03	12.39	10.85	-11.97
Gm22548	14.18	16.44	14.86	13.56	-11.83
Gm7381	5.4	5.5	8.51	5.08	-11.59
Rpl37a	10.76	11.79	13.25	10.75	-11.57
Gm11263	8.67	8.56	10.47	6.83	-11.56
	9.62	11.12	11.56	9.54	-11.49
Gapdh-ps14	9.1	11.25	10.27	8.9	-11.47
Ndufb9	6.54	7.52	8.21	5.68	-11.44
Vkorc1	4.54	5.27	7.7	4.92	-11.34
Gm16209	5.21	6.43	7.32	5.03	-11.33
Gm12537	14.53	16.72	15.83	14.54	-11.21
Gm17383	6.79	7.1	10.29	7.13	-11.13
Gm3200	8.5	10.18	9.61	7.83	-11.05
	4.69	4.35	8.01	4.2	-11.03
Hist1h2aj	4.75	5.62	7.54	4.95	-10.97
Gm23728	10.3	9.99	12.72	8.97	-10.93
Gm12669	10.12	11.69	11.99	10.13	-10.78
Gm2445	9.57	11.26	10.67	8.95	-10.68
Gm17541	9.91	10.5	12.5	9.67	-10.68
D8Ertd738e	6.67	6.51	8	4.43	-10.6
Ndufb11	5.41	6.28	7.78	5.26	-10.55
Txnl1	6.19	7.1	7.45	4.97	-10.46
Gm19774	7.76	6.11	10.19	5.15	-10.46
Gm6065	7.53	7.2	9.13	5.44	-10.2
Gm3222	11.68	13.52	12.81	11.29	-10.19

	4.24	4.81	6.87	4.1	-10.17
Smdt1	6.03	6.22	8.18	5.04	-10.1
Glns-ps1	9.81	10.84	11.43	9.13	-10.03
	9.01	9.41	11.7	8.79	-9.9
Gm10108	9.13	9.44	11.05	8.07	-9.79
Rpl19-ps4; Gm27506	4.77	5.66	6.56	4.16	-9.76
Gm6316; EG622339	8.32	9.83	9.35	7.58	-9.75
Uqcrq	6.71	7.51	8.42	5.93	-9.74
Dcn	4.55	4.96	7.19	4.35	-9.57
Rpl18a	10.71	10.97	12.78	9.78	-9.57
	4.58	5.84	6.73	4.73	-9.53
	4.47	7.08	4.82	4.2	-9.46
Gm7286	7.17	9.04	8.8	7.43	-9.44
Ndufa13	7.25	7.73	9.74	6.98	-9.42
Rpl22l1	4.98	5.45	7.86	5.1	-9.42
Gm10359	14.67	16.63	15.9	14.65	-9.28
Eef2	6.79	7.97	8.55	6.53	-9.15
LOC100861862	4.2	5.78	5.86	4.25	-9.15
Epas1	8.23	8.86	10.14	7.58	-9.09
Mir3097	6.11	8.19	7.52	6.42	-9.09
Gm3534	9.35	11.52	10.58	9.58	-9.02
Gm24518	5.68	5.73	7.35	4.24	-8.95
Gm13292	14.28	16.05	15.35	13.95	-8.94
	11.64	13.53	13.46	12.19	-8.91
Selt	6.87	8.28	8.01	6.28	-8.84
Gm16378	4.74	5.96	7.19	5.31	-8.61
Gm22860	17.88	19	19.12	17.14	-8.59
Gm10273	8.23	8.93	10.34	7.94	-8.57
Hist1h2af	5.19	5.92	7.13	4.76	-8.56
Gpm6a	9.31	11.06	11.39	10.05	-8.55
Gm17068	8.16	9.53	9.44	7.72	-8.53
Gm4691; RP24-363O21.2	10.57	11.53	11.22	9.08	-8.5
Gm11518	7.15	6.5	8.02	4.3	-8.44
Kif5a	6.88	7.83	8.64	6.52	-8.41
Gm24187	19.22	13.79	19.75	11.26	-8.38
Rpl10a-ps1	10.04	10.18	11.96	9.03	-8.32
Gm13772	8.56	8.78	10.24	7.42	-8.25
Ndrg2	13.5	13.92	15.06	12.44	-8.23
Fam107a	10.86	11.97	12.2	10.26	-8.2
Gm10291	9.52	11.31	10.55	9.3	-8.2
Hba-a2; Hba-a1	9.55	9.25	10.9	7.57	-8.17

Gm12254	7.09	7.27	8.95	6.09	-8.15
Gm26300; Gm11285	8.33	9.58	10.59	8.82	-8.13
Gm10233	8.64	9.89	11.18	9.41	-8.11
Gm14292	6.23	5.76	9.01	5.53	-8.06
Gm6517	5.84	7.04	7.56	5.75	-8.04
	4.75	6.37	5.74	4.36	-8
Gm5559	12.81	14.19	13.82	12.2	-7.99
Rpl3	9.42	9.14	12.03	8.78	-7.83
Gm4609	14.5	16.1	15.63	14.26	-7.82
Gm12419	6.43	8.21	7.45	6.27	-7.82
LOC100861833	6.7	6.94	8.02	5.3	-7.82
Grina	6.27	7.02	8.63	6.43	-7.72
Gm27459	9.57	9.82	10.73	8.04	-7.66
Gm2606	12.45	13.22	13.36	11.19	-7.64
	6.67	8.45	8.03	6.88	-7.63
Rpl29; Gm8210	6.65	6.8	7.73	4.96	-7.62
Cd81	6.9	7.48	8.96	6.62	-7.58
Gm6293	4.36	5.43	6.08	4.22	-7.57
Gm13394	10.07	10.3	10.43	7.75	-7.56
Fads2	5.65	6.63	6.9	4.96	-7.55
Gm8756	9.11	10.57	9.8	8.35	-7.54
Gm6767	7.7	8.24	9.16	6.79	-7.52
	10.19	10.74	10.93	8.62	-7.31
Sepw1	6.5	7.72	8.18	6.54	-7.26
Gm24245	19.29	13.95	19.93	11.72	-7.26
Gm24270	19.29	13.95	19.93	11.72	-7.26
Gm5863	9.22	10.71	10.34	8.97	-7.22
Hba-a2; Hba-a1	11.24	9.91	12.83	8.66	-7.2
Papd4	4.69	5.85	6.28	4.6	-7.17
Gm11478	12.17	11.78	14.49	11.26	-7.17
Gm7363	7.04	7.31	9.11	6.54	-7.16
Rpl19	5.52	6.39	6.96	4.99	-7.15
Rpl27-ps2	5.03	4.93	7.02	4.08	-7.15
Gm7664	4.05	5.95	5.4	4.47	-7.13
Cox8a	9.4	9.78	11.02	8.57	-7.11
Gm25241	6.3	7.01	7.93	5.81	-7.1
Eif3s6-ps1	7.39	6.74	8.3	4.84	-7.06
Gm13841; Rpl29	6.75	6.76	8.11	5.32	-7.01
Actb	8.98	9.41	10.37	7.99	-6.99
Gm17081; LOC100862237	9.18	10.04	10.92	8.98	-6.99
Gm23019	14.18	13.62	15.59	12.23	-6.97
	9.98	12.01	11.88	11.12	-6.93

Rpl19-ps12	6.05	6.87	7.75	5.78	-6.93
LOC100861642	8.11	7.97	10.19	7.26	-6.89
Rnaset2b	5.32	5.83	7.2	4.93	-6.87
Gm10254	8.86	10.55	10.09	9.01	-6.8
Gm7336	12.32	12.86	13.06	10.84	-6.79
Gm14387	7.12	7.64	8.34	6.1	-6.76
Gm6747	7.67	7.11	9.18	5.88	-6.72
Rpl31-ps14	18.63	18.6	18.65	15.88	-6.7
Sod1	8.76	8.72	10.32	7.54	-6.67
Gm14111	10.94	12.19	12.1	10.61	-6.67
	4.82	5.73	6.54	4.72	-6.66
Gm13433	5.5	5.71	7.97	5.44	-6.66
Gm7618	8.85	9.97	10.01	8.4	-6.65
Pias1	5.52	5.72	7.3	4.77	-6.64
Gm23388	16.8	13.52	17	10.99	-6.64
Gm10243	8.64	8.01	10.64	7.28	-6.63
LOC100862257	5.06	5.82	6.43	4.46	-6.58
	5.92	6.93	8.12	6.43	-6.54
Rpsa-ps12	5.28	6.8	7.04	5.86	-6.5
Gm8648	7.27	7.42	9.61	7.06	-6.46
Gm23566	14.41	14.24	15.54	12.67	-6.46
Gm6428	5.93	6.79	7.81	6	-6.43
	3.52	3.57	6.16	3.54	-6.4
Gm7251	9.42	10.87	9.93	8.7	-6.38
Gm11185	8.48	10.36	9.6	8.82	-6.32
Gm26365	7.29	8.1	8.18	6.33	-6.31
Canx	9.86	10.38	11.01	8.88	-6.25
	15.63	16.23	16.47	14.43	-6.23
Gm11675	5.71	6.08	7.41	5.15	-6.2
Tspan31	6.97	7.77	7.35	5.54	-6.14
Gm12346	6.88	8.32	9.03	7.85	-6.14
LOC100862094	6.88	6.52	8.6	5.62	-6.12
Gm10020	7.8	6.95	10.12	6.67	-6.11
Gm19494	5.48	4.61	7.83	4.35	-6.1
Gm14760	12.59	12.94	13.05	10.79	-6.09
	12.15	13.39	13.39	12.03	-6.08
Gm5430	9.45	11.36	10.41	9.73	-5.98
Gm4335	7.25	8.72	8.47	7.36	-5.96
Gm12671	12.51	13.03	13.3	11.24	-5.96
Rpl18-ps2	7.52	6.83	9.93	6.67	-5.95
Rpsa-ps9	8.08	8.84	9.96	8.17	-5.91
	4.79	6.6	5.62	4.86	-5.91
Gm14150	5.8	6	7.85	5.49	-5.9
Pts	4.81	5.43	6.5	4.57	-5.88

Gm11808	8.54	9.1	9.98	7.99	-5.87
Gm13162	6.82	7.44	7.52	5.58	-5.87
Gm11367	5.55	6.19	7.35	5.45	-5.84
Rpl3-ps2	15.89	15.88	17.28	14.73	-5.83
Tuba1a	7.4	7.97	9.07	7.1	-5.82
	3.83	4.14	6.01	3.78	-5.81
Gm4883	8.96	8.91	10.86	8.28	-5.81
Acta2	6.79	6.66	8.52	5.86	-5.8
	7.97	9.88	9.91	9.29	-5.8
Gm13464	12.4	13.98	13.8	12.85	-5.8
Atp6ap1	5.82	6.84	6.93	5.43	-5.78
Gm12428	12.22	13.67	13.59	12.51	-5.78
Gm18859	7.75	9.23	8.59	7.54	-5.75
Gm6139	7.19	7.15	8.89	6.33	-5.74
Atxn10	6.27	7.37	7.5	6.08	-5.71
Gm20899; Gapdh-ps15	7.58	8.02	7.97	5.9	-5.68
	7.28	6.45	9.31	5.97	-5.67
Gm6444	4.78	5.28	6.92	4.92	-5.66
Rpl31-ps16	17.42	17.32	17.33	14.73	-5.65
Gm5944	8.29	9.37	8.93	7.52	-5.64
Pdxk	6.98	7.35	7.47	5.35	-5.63
Ndufb7	5.41	5.9	6.87	4.86	-5.62
Gm4654	11.74	13.37	12.47	11.62	-5.61
	13.78	15	15.24	13.98	-5.58
Gm13509	6.66	7.68	8.4	6.95	-5.56
Cox5a	4.99	5.2	7.77	5.51	-5.53
	4.37	4.7	6.4	4.26	-5.53
	5.67	6.94	5.73	4.53	-5.52
Gm23368	19.08	19.06	19.08	16.6	-5.51
Gm8129	10.8	10.75	12.35	9.84	-5.51
Gm5210	6.24	7.34	7.24	5.88	-5.49
Ptprb	7.54	7.74	8.76	6.51	-5.48
Gm15483	6.3	6.91	7.75	5.91	-5.48
Gm11273	6.72	6.13	8.53	5.49	-5.46
Zfp938	6.43	6.2	7.72	5.06	-5.43
Gm12372	5.77	5.94	6.94	4.67	-5.42
	3.74	4.56	5.24	3.63	-5.41
Psmb5; Mir686	7.01	5.96	8.73	5.25	-5.38
Eif5al3-ps	4.84	5.2	6.5	4.43	-5.34
	6.32	5.46	7.18	3.9	-5.34
Gm24269	11.94	12.94	13.07	11.66	-5.31
Gm10284	14.79	16.18	15.67	14.67	-5.24
Gm14046	8.21	8.23	8.58	6.22	-5.23

Gm14148	11.49	12.21	12.65	10.99	-5.22
Arl8b	6.44	6.55	7.95	5.69	-5.2
Gm13882	9.43	11.13	10.54	9.86	-5.19
Gm6863	11.35	11.44	12.36	10.08	-5.17
Cds2	7.79	8.04	8.85	6.73	-5.16
Tuba4a	5.01	5.73	6.48	4.83	-5.16
Gm5777	8.05	8.02	9.54	7.15	-5.14
Nedd4	8.15	8.46	8.82	6.78	-5.1
Gm11249	9.3	8.9	11.36	8.61	-5.09
Gm2004	8.83	8.94	10.02	7.78	-5.07
Gm26944; RP23-474B13.3	4.13	5.63	4.97	4.14	-5.07
Fis1	6.77	7.42	7.84	6.16	-5.03
Rpl31-ps11	17.08	17.03	17.03	14.64	-5.01
Clk3	5.73	6.58	7.13	5.65	-5
Gm17150	10.06	9.59	12.08	9.3	-4.99
Ubc; Uba52	9.72	10.2	12.18	10.34	-4.97
Clu	5.88	6.62	7.22	5.66	-4.95
Gm10224	7.02	6.57	8.55	5.8	-4.93
Ppp2cb	3.92	4.76	5.37	3.92	-4.9
	6.29	6.81	7.97	6.2	-4.88
Prpf8	5.43	5.76	7.24	5.28	-4.87
	9.38	9.47	10.37	8.19	-4.84
Gm10313	8.5	9.37	9.53	8.13	-4.84
	3.95	4.96	5.04	3.78	-4.81
Mal	7.79	7.8	7.73	5.47	-4.8
Gm19738	6.13	5.36	7.99	4.96	-4.8
Dusp6	4.95	6.77	5.68	5.24	-4.78
Gm2574	8.86	9.19	8.71	6.78	-4.77
Sparcl1	15.31	15.35	16.38	14.17	-4.76
Rpl31-ps17	16.17	16.12	16.12	13.81	-4.76
Ndufb6	5.47	6.2	7.18	5.66	-4.74
Gm24811	13.95	13.51	15.19	12.5	-4.74
Rpl31-ps20	15.37	15.35	15.39	13.13	-4.74
Atp1a2	10.16	10.4	11.74	9.75	-4.72
Prkag1	5.09	4.93	7.03	4.63	-4.72
Tuba1b	6.29	6.48	8.05	5.99	-4.72
Gm3608	5.62	5.6	7.27	5.01	-4.72
Gm15459	11.88	12.46	12.88	11.24	-4.65
Gm8927	9.44	10.1	10.5	8.95	-4.64
LOC100862170; 4933409K07Rik	7.2	7.74	8.61	6.94	-4.64
Eif4g2	7.09	7.4	8.32	6.42	-4.63
Grm3	7.52	7.97	8.57	6.81	-4.63

Npm1	8.59	9.35	10.04	8.59	-4.63
Glyr1	6.15	7.25	6.53	5.41	-4.62
Brms1l	4.77	5.66	6.29	4.98	-4.61
Plpp3	4.81	5.4	5.64	4.03	-4.59
	7.13	6.35	8.83	5.85	-4.59
2900011O08Rik	6.25	6.91	7.62	6.09	-4.58
Gm5564	8.14	7.05	9.08	5.79	-4.58
Gm14165	6.44	6.72	7.62	5.71	-4.57
	3.87	5.1	5.72	4.76	-4.54
	12.13	13.33	13.37	12.39	-4.53
Rpl28-ps3	8.72	8.99	10.64	8.73	-4.53
	4.41	5.17	5.4	3.98	-4.52
Gm1866	4.45	5.23	6.02	4.62	-4.52
Gm7497	5.34	5.54	7.08	5.11	-4.52
Apoa1bp	7.55	7.37	9.11	6.76	-4.51
Chst1	5.73	7.06	6.41	5.57	-4.51
BC002163	6.28	6.54	8.03	6.11	-4.5
Mpzl1	4.74	5.75	5.95	4.78	-4.5
Gm10736	6.47	7.18	7.23	5.77	-4.48
	5.19	6.68	6.06	5.38	-4.47
Aspn	5.42	6.39	6.32	5.14	-4.42
Gm16399	6.17	5.9	7.7	5.29	-4.38
Utrn	7.26	7.84	8.35	6.81	-4.37
	6.04	5.64	7.66	5.13	-4.36
Gm5265	5.56	6.35	7.47	6.14	-4.36
Gm7507	9.47	10.73	10.26	9.4	-4.35
Gm10221	9.4	9.38	11.15	9	-4.35
Ctnnb1; Mir7090	5.44	6.22	6.43	5.09	-4.34
Cox5b	8.82	7.9	10.4	7.37	-4.34
Ube2l3	6.16	6.39	7.68	5.79	-4.34
	5.47	6.55	6.12	5.09	-4.33
Actg1; Mir6935	6.88	6.76	8.18	5.95	-4.32
	3.73	4.55	5.03	3.75	-4.31
Mir6412	8.1	7.91	9.69	7.4	-4.3
Mobp	6.44	7.66	6.16	5.27	-4.3
Gm14399	8.11	7.68	9.48	6.95	-4.29
Gm9625	5.97	6.34	7.43	5.7	-4.28
Gm23141	6.71	7.16	7.55	5.91	-4.28
Gm17228	4.26	6.04	4.55	4.24	-4.26
Rabac1	5.88	6.08	8.02	6.14	-4.24
Pak1	9.73	10.92	10.85	9.96	-4.23
Rpl31-ps13	13.89	13.88	13.88	11.78	-4.23
Gm3695	6.89	8.16	8.15	7.35	-4.22
Igfbp7	6.68	6.66	7.92	5.82	-4.21

Gm19976	6.29	5.98	7.42	5.03	-4.21
Elf1	4.31	5.07	5.75	4.43	-4.2
Gm14777	7.93	9.42	8.67	8.09	-4.19
Gm10293	14.02	15.82	14.66	14.39	-4.19
Gm5139	6.93	7.55	7.83	6.39	-4.17
Ptgds	7.83	8.1	8.94	7.15	-4.16
Pten	5.49	5.83	6.68	4.96	-4.16
Gm23119	5.4	4.98	6.39	3.92	-4.16
Gm10015	6.14	6.21	7.48	5.5	-4.15
Rpl32-ps	7.93	9.23	9.11	8.36	-4.15
Gm8055	7.13	9.01	7.82	7.66	-4.13
Hspa4	6.62	6.88	7.68	5.9	-4.12
	4.9	5.66	6.67	5.4	-4.11
Rpl31-ps10	16.72	16.49	16.62	14.35	-4.11
	4.56	4.65	6.17	4.22	-4.1
Cct5	5.66	6.03	6.88	5.22	-4.09
Wsb1	5.35	5.43	6.66	4.71	-4.09
	6.13	6.96	6.78	5.58	-4.08
Gm25128	7.22	6.68	7.91	5.35	-4.04
	4.08	3.96	5.76	3.62	-4.03
Gm9844	7.42	8.16	8.32	7.06	-4.03
Gm14870	5.6	5.78	6.48	4.65	-4.02
Gm15484	6.95	8.5	7.84	7.39	-4
Gm27248	6.49	7.18	7.63	6.32	-4
Atp6v1e1	8.64	8.82	8.82	7.01	-3.99
Gm14130	8.8	10.1	9.64	8.95	-3.99
Slc2a1	10.11	10.09	10.48	8.47	-3.98
Atp1b2	5.01	5.65	6.43	5.08	-3.96
Tecr	7.48	7.53	8.86	6.92	-3.95
Gm12033	9.32	9.78	9.69	8.17	-3.95
Gm5566	5.06	5.57	5.96	4.49	-3.95
Gm13298; Fam205a4; Gm20938	4.7	5.46	5.89	4.67	-3.94
Cox6a1	7.81	7.27	9.39	6.87	-3.93
	4.13	4.57	5.54	4	-3.93
	4.71	4.74	6.17	4.24	-3.91
Cops3	5	5.26	6.3	4.6	-3.9
Snord16a	3.42	3.55	5.52	3.69	-3.9
Gm10126	8.36	9.68	9.27	8.63	-3.9
Gm23872	14.52	14.81	15.21	13.54	-3.9
Gm10051	8.25	8.61	9.93	8.32	-3.9
Gm16412	5.98	6	7.5	5.57	-3.9
Glrx3; Gm12669	8.2	8.63	8.95	7.42	-3.88

Gm13573	6.01	6.34	7.77	6.14	-3.88
Gm2383	6.77	8.43	7.38	7.08	-3.87
Gm13862	7.13	7.93	8.44	7.28	-3.87
Gm23053	16.92	16.79	18.12	16.04	-3.87
Tgfb3	4.82	5.11	5.94	4.31	-3.8
Tsn	5.03	5.17	6.44	4.65	-3.8
	14.8	15.65	15.61	14.54	-3.8
Abhd3	5.61	5.96	6.79	5.22	-3.79
Erh	5.32	5.95	6.85	5.55	-3.79
Kcna1	6.28	6.56	7.16	5.52	-3.78
	5.95	6.88	7.01	6.03	-3.78
Rpl28-ps1	9.29	9.75	10.78	9.33	-3.78
Crry-ps	5.26	6.21	6.55	5.58	-3.77
Ptprg	6.49	6.57	7.16	5.33	-3.77
Rab24	5.47	5.29	6.71	4.61	-3.77
	4.41	5	5.74	4.42	-3.77
Cisd1	3.95	4.38	5.35	3.87	-3.75
Gm14403	7.31	7.09	8.51	6.39	-3.73
Gm5787	6.54	7.33	7.42	6.31	-3.73
	5.11	6.48	6.22	5.71	-3.7
Pgam1-ps2	5.81	6.97	6.68	5.95	-3.69
Cd248	4.63	5.26	5.79	4.54	-3.68
Ncam1	5.22	5.75	6.46	5.11	-3.68
Pcna-ps2	4.7	5.26	6.05	4.73	-3.68
Pcnp	5.84	6.02	7.38	5.69	-3.67
Gm6807	5.35	5.93	6.22	4.93	-3.65
Gm16355	5.77	6.07	6.65	5.09	-3.64
Rgs7bp	5.66	7.39	6.96	6.83	-3.63
Trf	8.58	8.55	9.62	7.73	-3.63
Ntsr2	4.94	5.76	6	4.96	-3.62
Gm12070	6.36	6.88	6.48	5.14	-3.62
Gm8420	4.76	5.14	5.68	4.21	-3.61
Rpl15-ps3	6.15	6.35	6.99	5.34	-3.61
Arl5a	6.1	6.66	7.21	5.93	-3.6
Mir1195	15.56	15.56	15.57	13.72	-3.59
Gm11223	11.56	11.83	12.39	10.82	-3.59
Gm27731	7.88	8.18	8.51	6.97	-3.58
Ndufa10	5.82	6.26	7	5.6	-3.57
Gm6822	5.91	6.08	7.51	5.84	-3.57
Gm8318	6.52	8.09	6.95	6.69	-3.55
Dynlrb1	8.45	8.19	9.78	7.7	-3.54
Gm10540	11.56	11.33	12.29	10.24	-3.54
Gm12074	4.37	4.27	5.63	3.7	-3.54
Atp1a3	7.13	7.92	8.38	7.35	-3.53

Uqcrc2	5.74	6.18	6.98	5.61	-3.53
Ntan1	5.17	6.21	5.52	4.74	-3.52
Gm10480	9.43	10.15	10.47	9.38	-3.52
	9.38	9.21	9.42	7.45	-3.5
Gm12967	5.51	6.57	6.54	5.79	-3.5
Gm17420	12.27	13.04	13.22	12.18	-3.5
Gm11336	4.65	5.28	5.34	4.18	-3.46
Enpp5	5.1	5.98	5.68	4.78	-3.43
Oat	4.29	4.92	5.25	4.1	-3.43
	5.82	6.15	7.07	5.61	-3.43
Gm12481	6.11	6.21	7.9	6.23	-3.42
LOC100862081	7.2	7.32	8.32	6.67	-3.42
	4.41	5.06	5.32	4.21	-3.41
Rock1	6.14	5.73	7.26	5.08	-3.4
Rpl31-ps1	13.41	13.33	13.49	11.64	-3.39
Aff4	5.88	6.23	7.05	5.64	-3.38
Gm11557	7.01	8.27	7.74	7.25	-3.38
Hipk3; Mir1902	6.66	7.4	7.73	6.72	-3.37
Klf6	6.78	6.95	7.3	5.71	-3.37
	5.3	6.04	5.41	4.4	-3.37
Gm5844	8.04	8.57	9.6	8.39	-3.37
Fam205a2; Gm10600	4.8	5.58	5.81	4.85	-3.36
Kif5b	8	8.01	8.87	7.13	-3.35
Gm8730	6.53	6.51	7.3	5.54	-3.34
Mlc1	6.16	7.31	7.32	6.73	-3.33
Stk39	6.56	6.86	7.75	6.32	-3.32
	4.21	4.4	6.1	4.57	-3.32
Gm20775	5.1	5.85	6.37	5.39	-3.32
Atp1b1	8.43	8.69	10.15	8.69	-3.31
Pcna	4.95	5.37	6.24	4.95	-3.29
Gm12922	9.2	9.24	10.36	8.67	-3.29
Mtch1	7.24	7.7	8.29	7.04	-3.28
Dhx9	6.35	6.76	7.46	6.16	-3.26
	4.3	4.73	5.33	4.05	-3.26
Gm13171	4.58	4.94	5.54	4.2	-3.25
Gm14269	7.32	7.15	8.01	6.14	-3.25
Gm19767	4.73	4.7	6.15	4.42	-3.25
1500011B03Rik	4.99	5.82	5.89	5.03	-3.23
	5.26	5.36	6.59	4.99	-3.23
Gm13226	7.17	7.59	8.61	7.33	-3.23
Gm14388	3.88	4.35	5.32	4.1	-3.23
Gm26983	4.28	4.82	5.53	4.38	-3.23
Gm4518	13	13.16	13.5	11.97	-3.22

Gm6322	5.73	6.82	6.36	5.76	-3.22
Amd2; Amd1	6.79	6.6	8.07	6.2	-3.21
Tmem47	5.82	5.97	6.7	5.16	-3.21
	4.6	4.89	5.62	4.23	-3.21
Gm17756	4.31	4.53	5.43	3.96	-3.21
Ephx1	5.94	5.99	7.06	5.43	-3.2
Myh11	5.67	6.22	6.62	5.5	-3.19
Gm27038	4.75	5.13	5.45	4.15	-3.19
Gatad1	5.26	5.79	6.49	5.35	-3.18
Myl6	11.52	11.48	12.28	10.58	-3.17
	4.04	4.28	5.62	4.2	-3.17
Gm6054	6.97	7.01	7.77	6.16	-3.16
Gm11539	5.88	5.84	7.47	5.77	-3.16
Gm8100	8.59	9.56	8.93	8.24	-3.15
Cox4i1	6.15	6.48	7.29	5.96	-3.14
Pcdh9	5.26	5.29	6.92	5.3	-3.14
Ppa1	5.42	5.43	6.6	4.96	-3.14
Gm8738	4.51	5.21	5.83	4.87	-3.14
Gstp1	5.78	5.99	7.12	5.68	-3.12
Nucb2	5.95	6.06	7.09	5.56	-3.12
Atp2b4; Mir6903	5.99	6.03	7.1	5.5	-3.11
Ctsb	6.46	6.75	7.75	6.4	-3.11
Gm14322	7.88	7.55	8.66	6.69	-3.11
Gm12017	7.62	8.83	8.13	7.7	-3.11
Clic4	5.57	6.21	6.8	5.82	-3.09
Rpl32	6.44	7.17	7.55	6.66	-3.09
Uchl1	4.86	4.75	6.39	4.65	-3.09
LOC100862063	4.47	4.56	5.63	4.09	-3.09
Kif1a	6.61	7.31	7.73	6.8	-3.08
Gm3893; 4933409K07Rik	6.52	7.11	7.6	6.57	-3.08
Snx32	4.45	5.23	5.45	4.6	-3.08
Dctn2	4.35	5.03	5.47	4.53	-3.07
Gatm	6.98	7.17	7.46	6.03	-3.06
	4.47	4.74	5.59	4.24	-3.06
	4.34	4.35	5.67	4.07	-3.06
Napb	5.39	5.52	6.75	5.27	-3.05
	4.93	5.47	5.97	4.89	-3.05
Lancl1	5.26	5.39	6.4	4.92	-3.04
Nfe2l1	7.6	7.69	8.59	7.08	-3.04
	3.58	5.15	3.58	3.55	-3.04
Gm2897	4.89	5.83	5.17	4.51	-3.04
Gm9938	3.94	4.76	4.93	4.15	-3.04

Gm13298; Fam205a2	4.87	5.39	5.77	4.69	-3.04
Gm13298; Fam205a3; Gm21598	4.87	5.39	5.77	4.69	-3.04
	4.96	5.63	5.54	4.62	-3.03
Arxes1	3.94	4.43	4.94	3.84	-3.02
Atp5b	6.91	6.84	8.49	6.82	-3.02
Ndufa9	6.31	6.22	7.16	5.47	-3.02
Gm4462	6.79	7.45	7.32	6.39	-3.02
Gm23045	19.16	19.16	19.19	17.59	-3.02
Gm15975	4.01	5.19	4.63	4.22	-3.01
Med29	4.11	4.86	5.01	4.17	-3
Sv2b	5.91	6.77	7.11	6.38	-3
Tmod2	6.72	6.77	7.63	6.09	-3
Gm25233	5.65	6.11	6.38	5.26	-3
App	7.34	7.98	7.93	7	-2.98
Gm10408	4.42	5.77	4.5	4.28	-2.98
Raph1	4.54	4.64	5.71	4.23	-2.98
Gm14421	6.81	6.67	7.51	5.81	-2.96
Pik3r3	6.39	6.57	7.33	5.94	-2.95
	3.81	4.68	4.39	3.7	-2.95
A130040M12Rik	6.44	6.67	7.29	5.97	-2.94
Gdi1	9.65	9.94	11.43	10.18	-2.92
Ociad2	5.75	6	6.67	5.38	-2.91
Slc38a11	5.76	5.93	6.45	5.07	-2.91
	5.59	7.01	5.76	5.65	-2.91
Slc25a18	4.19	5.22	5.46	4.94	-2.9
	4.34	4.89	4.99	4.01	-2.9
Cdc37l1	5.63	6.25	6.05	5.13	-2.89
Ldhb	7.04	7.05	7.88	6.36	-2.89
Ppargc1a	5.06	5.51	5.99	4.9	-2.89
	5.91	6.77	6.68	6	-2.89
Maf	4.76	5.13	5.88	4.73	-2.88
Slc12a2	4.41	4.52	5.72	4.3	-2.88
	6.34	7.25	7.29	6.67	-2.88
Gm11511	3.52	3.71	5.2	3.87	-2.88
Psmc1	6.9	6.45	7.92	5.95	-2.87
Ppp6r3	5.73	6.34	6.36	5.44	-2.87
	4.57	5.46	4.97	4.34	-2.87
Oxct1	5.62	6.23	6.71	5.81	-2.86
Atp2a2	9.67	9.21	10.64	8.66	-2.86
Map1b	6.79	7.38	7.17	6.25	-2.86
Nrd1; Mir761	7.04	7.09	7.71	6.25	-2.86

Ptn	9.08	8.94	9.47	7.82	-2.86
Gm6134	5.56	6.24	6.46	5.62	-2.86
Myl12b	7.48	8.08	8.33	7.43	-2.85
Vim	4.6	4.83	5.82	4.54	-2.83
Gm6887	5.29	5.36	5.69	4.26	-2.83
Pnmal1	5.6	5.92	6.69	5.53	-2.82
Rpl37	9.52	10.9	10.46	10.34	-2.82
Gm10481	12.49	13	13.13	12.14	-2.82
	4.16	4.39	5.39	4.13	-2.81
Ost4	6.93	6.55	8.21	6.35	-2.8
Gmps	4.23	4.6	5.29	4.19	-2.79
Hivep1	4.83	5.4	6.15	5.25	-2.79
Sumo1	6.33	6.28	7.12	5.59	-2.79
Gm5612	6.63	7.39	7.96	7.25	-2.79
Gm19974	4.88	5.11	6.37	5.12	-2.79
	5.16	5.6	5.83	4.79	-2.78
	5.16	5.6	5.83	4.79	-2.78
Lrp1	5.93	6.43	6.94	5.97	-2.78
Gm4353	3.99	5.01	4.48	4.03	-2.78
Gm11849	7.08	7.12	7.79	6.36	-2.78
Atp5a1	6.94	6.98	7.54	6.11	-2.77
	4.4	4.3	5.49	3.91	-2.77
Camk2g	6.42	6.69	6.93	5.74	-2.76
Gsk3a	5.76	6.71	6.51	5.98	-2.76
	3.91	5.09	4.07	3.78	-2.76
Gm10566	13.56	13.72	13.85	12.55	-2.76
Carm1	4.79	5.07	5.75	4.57	-2.75
Micu2	5.02	5.23	5.99	4.75	-2.75
	4.17	4.75	4.89	4.02	-2.75
Gm5866	5.6	6.16	6.25	5.35	-2.75
Gm6055	6.08	5.87	7.4	5.73	-2.75
Gm9103	4.54	4.95	5.41	4.36	-2.75
Gm11824	6.25	6.84	6.92	6.05	-2.75
Cfdp1	5.94	6.07	7.02	5.7	-2.74
Gcnt2	5.02	5.16	5.67	4.35	-2.74
	5.93	6.61	6.78	6.02	-2.74
Psmb2	4.2	4.69	5.12	4.16	-2.73
Ube2n	5.11	5.47	6.07	4.97	-2.73
	3.94	4.71	4.54	3.87	-2.73
Gm4987	5.35	5.97	6.35	5.53	-2.73
Ap3d1	5.36	5.74	6.07	5.01	-2.72
Zfp931	5.61	5.36	6.77	5.08	-2.72
Flna	7.17	6.85	7.99	6.24	-2.71
Naa38	4.25	4.5	5.18	3.99	-2.71

	4.07	4.94	4.65	4.07	-2.71
Rngtt	4.47	4.98	5.7	4.78	-2.7
	5.09	5.19	5.96	4.63	-2.7
	3.96	4.95	4.3	3.86	-2.7
Cdc42bpa	5.96	6.49	6.59	5.7	-2.69
	3.7	4.72	4.66	4.26	-2.69
Babam1	5.54	5.76	7.09	5.89	-2.68
	5.56	6.89	5.43	5.33	-2.68
Pdhb	6.06	6.22	7.28	6	-2.68
Rnf187	5.37	5.85	6.37	5.43	-2.68
	4.23	4.83	5.13	4.3	-2.68
Gm14418	7.91	7.46	8.66	6.8	-2.68
Atrnl1	5.64	6.03	6.76	5.74	-2.67
Gabbr2	6.72	7.56	7.81	7.24	-2.67
Idh3g	7.19	7.04	7.64	6.07	-2.67
Gm10358	12.79	13.12	13.23	12.14	-2.67
Gm25591	12.29	12.21	12.02	10.52	-2.67
Ube2k	7	7.4	7.72	6.71	-2.66
Nol4	4.78	4.9	5.85	4.57	-2.65
Il6st	5.5	5.8	6.16	5.06	-2.64
D17Ertd648e	5.68	6.55	6.04	5.51	-2.62
Rap2a	4.51	4.87	5.35	4.33	-2.62
Cyr61	5.22	5.84	5.96	5.2	-2.61
Sept11	6.47	6.93	7.35	6.42	-2.61
Saraf	4.51	5.01	5.15	4.27	-2.61
Gm12727	5.9	6.01	6.72	5.45	-2.61
Cd63	8.53	8.48	9.03	7.6	-2.6
	5.96	6.68	6.73	6.08	-2.6
	4.55	4.42	5.87	4.35	-2.6
Fam205a1	4.42	5.02	5.03	4.26	-2.59
Ppp1r14b	5.38	5.85	6.52	5.63	-2.59
Gm4468	5.56	6.07	6.54	5.68	-2.58
	3.7	4	4.68	3.61	-2.57
Arntl	4.96	5.34	5.55	4.58	-2.56
Gm22930	5.45	5.32	6.84	5.35	-2.56
LOC100862384	4.82	4.33	6.16	4.31	-2.56
Bcan	5.87	6.35	6.41	5.54	-2.55
Capn7	6.92	7.26	7.33	6.33	-2.55
Tubg1	5.12	5.6	6.01	5.14	-2.55
	5.84	6.73	6.23	5.77	-2.55
Gm13050	5.07	5.19	6.17	4.94	-2.55
	6.97	7.06	8.12	6.87	-2.54
	3.97	5.48	4.13	4.29	-2.54
	3.63	4.77	3.99	3.78	-2.54

Hcn2	4.32	5.21	4.79	4.34	-2.53
Stub1; Mir3082	5.46	5.92	6.36	5.48	-2.53
Pdxp	3.67	4.12	4.46	3.58	-2.52
Rplp0	6.49	6.48	7.28	5.93	-2.52
Slc38a3	5.46	5.68	6.49	5.38	-2.52
	4.21	4.95	4.74	4.14	-2.52
Gm23064	16.52	16.68	17.26	16.09	-2.52
Gm25355	16.52	16.68	17.26	16.09	-2.52
Myl9	6.33	6.43	6.91	5.69	-2.51
Smarcc2	7.82	8.47	8.44	7.76	-2.51
Cwc15	6.1	6.47	6.77	5.82	-2.5
Rtn4	5.76	6.25	6.7	5.86	-2.5
	5.04	5.86	6.04	5.53	-2.5
Gm3934	5.78	5.62	6.66	5.18	-2.5
LOC100861805	5	4.95	6.26	4.89	-2.5
Kif1b	6.59	6.72	7.4	6.21	-2.49
Peg3os	6.79	6.92	7.81	6.62	-2.49
	7.51	7.83	7.91	6.91	-2.49
	8.31	7.57	8.73	6.67	-2.49
Gm4804	5.07	5.23	6.23	5.08	-2.49
Gm12912	6.3	6.56	6.79	5.73	-2.49
Gm6238	5.46	5.61	6.2	5.03	-2.49
Capza1	5.73	5.67	6.7	5.33	-2.48
Dnajc4	4.6	4.73	5.62	4.44	-2.48
Eef1a2	5.97	6.78	6.82	6.31	-2.48
Lonp2	4.88	5.07	6.07	4.94	-2.48
Ufd1l	4.96	4.78	5.87	4.38	-2.48
	4.38	4.62	5.43	4.37	-2.48
	3.39	4.46	3.9	3.66	-2.48
Gm16100	4.73	4.95	5.48	4.39	-2.48
Gm5436	5.76	5.99	6.91	5.82	-2.48
Rcor3	3.73	4.36	4.38	3.7	-2.47
Tspyl4	5.86	6.21	6.81	5.86	-2.47
Gm16238	5.42	5.77	6.46	5.51	-2.47
Lin7a	4.95	5.3	6.16	5.22	-2.46
Slc48a1	5.4	5.82	5.59	4.71	-2.46
Gm14944; RP23-302B23.2	5.02	5.02	6.14	4.84	-2.46
Mertk	5.38	5.61	6.09	5.03	-2.45
Sdhb	4.26	4.46	5.38	4.29	-2.45
	4.05	4.69	4.4	3.75	-2.44
Gm8412	4.56	4.85	5.28	4.28	-2.44
Cdh5	5.12	5.59	5.98	5.16	-2.43
Gm13149	5.02	5.56	5.88	5.14	-2.43

Wnk1	6.15	6.32	6.9	5.8	-2.42
	10.26	11.03	10.99	10.49	-2.42
Gm13695	4.04	4.84	4.57	4.09	-2.42
Amd-ps3	5.42	5.48	6.23	5.02	-2.42
Aplp1	6.41	7.02	6.99	6.33	-2.41
Sacm1l	4.57	4.8	5.22	4.19	-2.41
Gm14571	4.04	4.63	4.39	3.71	-2.41
Krit1	4.58	5	5.38	4.54	-2.4
Lynx1	3.91	4.35	4.57	3.75	-2.4
Gm12719	4.17	4.89	4.71	4.17	-2.4
Tnfaip1	5.03	5.39	5.96	5.07	-2.39
	4.29	4.94	4.9	4.29	-2.39
Gm13144	5.26	5.32	6.15	4.95	-2.39
LOC100861832	4.13	3.98	5.59	4.19	-2.39
Srsf7	5.85	5.7	6.39	4.99	-2.38
	7.53	7.87	8.34	7.43	-2.38
	5.34	4.51	6.32	4.24	-2.38
Gm12281	4.56	5.72	5	4.92	-2.38
Gm12460	6.97	7.12	8.08	6.98	-2.38
Eif1	7.07	7.38	7.9	6.96	-2.37
	14.72	14.69	15.51	14.23	-2.37
	3.92	4.34	4.81	3.98	-2.37
	5.14	6.08	5.32	5.01	-2.37
Agk	3.91	4.15	4.85	3.85	-2.36
Gm6612	4.56	4.8	5.53	4.53	-2.36
Gm8864	8.43	9.33	9.03	8.69	-2.36
Gm13693	4.02	4.72	4.5	3.96	-2.36
Gm13694	4.02	4.72	4.5	3.96	-2.36
Gm13696	4.02	4.72	4.5	3.96	-2.36
Gm13697	4.02	4.72	4.5	3.96	-2.36
Gm13698	4.02	4.72	4.5	3.96	-2.36
Fam195b	4.45	5.07	4.98	4.37	-2.35
Fads1	5.61	6.13	6.21	5.49	-2.35
Plxnc1	4.23	4.45	5.25	4.24	-2.35
Slc18b1	4.55	4.38	5.77	4.37	-2.35
	3.83	4.39	4.89	4.21	-2.35
LOC100862386	4.18	5.06	5.28	4.94	-2.35
Gm2163	5.87	6.47	6.75	6.12	-2.34
Gm12704	5.21	5.27	5.77	4.6	-2.34
Gm23215	13.93	14.02	14.22	13.08	-2.34
Map4k3	5.74	6.15	6.64	5.83	-2.33
	5.67	6.08	6.19	5.39	-2.32
Cdh2	5.52	6.12	6.28	5.68	-2.31
Gtf2i	5.72	6.16	6.03	5.26	-2.31

Tmem38a	5.82	6.19	5.98	5.14	-2.31
	5.9	6.67	6.73	6.3	-2.31
	8.16	9.25	9.23	9.11	-2.31
	4.86	4.91	5.29	4.13	-2.31
	5.82	5.62	6.08	4.67	-2.31
Gm12226	4.51	4.65	5.21	4.14	-2.31
Ndufv3	7.29	7.3	7.95	6.77	-2.3
Gm5473	9.59	9.82	9.48	8.51	-2.3
Mir669b	5.5	5.04	6.33	4.67	-2.29
	3.96	4.5	4.12	3.47	-2.29
Hist2h4	5.21	5.94	6.73	6.27	-2.28
Mrpl24	6.46	6.51	7.24	6.1	-2.28
Rpl6	5.3	5.95	5.94	5.41	-2.28
Gm6788	8.68	9.13	9.22	8.49	-2.28
Gm22694	10.31	10.17	10.63	9.3	-2.28
Pitpna	6.22	6.26	6.85	5.7	-2.27
	6.8	7.07	7.08	6.17	-2.27
Gm7985	8.23	9.04	8.82	8.45	-2.27
Larp4b	5.6	6.06	6.24	5.53	-2.26
March6	4.96	5.09	5.7	4.65	-2.26
Syn1	4.49	4.89	5.51	4.73	-2.26
Uso1	4.99	5.54	5.38	4.76	-2.26
	4.89	5.51	5.66	5.1	-2.26
	4.04	4.17	4.94	3.89	-2.26
Gm3715	4.98	5.82	5.19	4.86	-2.26
LOC100862073	6.68	6.77	7.46	6.38	-2.26
Rpl23a-ps4	4.17	4.54	4.99	4.18	-2.26
AK029949	5.46	6.09	6.21	5.66	-2.26
Bnip2	6.22	6.01	6.69	5.31	-2.25
Kif5c	6.65	7.06	7.18	6.41	-2.25
Mff	5.22	5.33	6.02	4.95	-2.25
Naa30	4.97	5.47	5.57	4.89	-2.25
Gm6644	5.19	4.94	5.87	4.46	-2.25
Gm4879	4.5	4.89	5.4	4.62	-2.25
Tmem59l	4.42	5.17	5.38	4.97	-2.24
Gm9143	7.72	7.7	8.26	7.08	-2.24
Gm14107	5.62	6.25	6.33	5.8	-2.24
Elovl4	4.28	4.32	5.13	4.02	-2.23
Gm25872	16.28	16.82	16.9	16.29	-2.23
Jag1	4.66	4.46	5.85	4.5	-2.22
Med30	4.1	4.55	4.91	4.22	-2.22
	3.52	4.77	3.76	3.86	-2.22
	4.07	4.48	5.11	4.37	-2.22
Gm12632	7.86	7.81	8.52	7.31	-2.22

Gm13545	6.15	6.33	6.76	5.79	-2.22
Gm19938	4.08	4.41	4.97	4.15	-2.22
Hey2	4.51	4.74	5.51	4.58	-2.21
Hbs1l	4.85	5.21	5.29	4.51	-2.21
Gm6245	3.76	4.09	4.86	4.05	-2.21
Efnb2	5.7	6.58	5.96	5.7	-2.2
Emsy	5.73	6.54	6.16	5.83	-2.2
	5.75	5.9	6.29	5.31	-2.2
	4.68	4.94	5.82	4.94	-2.2
Gm24108	11.29	11.89	11.71	11.17	-2.2
Rabl2	5.79	6.45	5.81	5.34	-2.19
Uck2	4.71	4.92	6.08	5.17	-2.19
Gm14639	5.36	5.88	5.58	4.97	-2.19
	7.85	8.15	9.25	8.42	-2.18
Actr2	8.96	9.34	8.78	8.04	-2.18
Gpi1	7.28	7.37	7.95	6.92	-2.18
Timm23	4.83	5.41	5.61	5.07	-2.18
Uqcrc1	5.06	5.45	6.02	5.28	-2.18
Gm13009	4.03	4.56	4.39	3.79	-2.18
Gm13436	4.3	4.1	5.57	4.25	-2.18
Dis3	4.68	4.87	5.41	4.48	-2.17
Pcyox1	5.73	5.66	6.64	5.45	-2.17
Clec1a	4.2	4.39	5.14	4.22	-2.16
Large	5.47	5.82	6.19	5.44	-2.16
Mrpl49	4.3	4.73	5.11	4.43	-2.16
	4.39	4.66	5	4.15	-2.16
Ighv3-5	3.37	4.3	3.59	3.41	-2.15
Atp5g3	5.82	5.81	6.32	5.21	-2.15
Cacybp	4.81	5.07	5.4	4.56	-2.15
Dap3	4.63	4.74	5.67	4.67	-2.15
Entpd1	5.01	5.2	5.77	4.86	-2.15
Fnta	4.39	4.71	5.2	4.41	-2.15
Ppp6c	5.46	5.37	6.04	4.85	-2.15
	4.6	5.39	5.13	4.81	-2.15
Gm6505	4.94	5.84	4.49	4.29	-2.15
Gm1974	4.47	4.6	5.49	4.52	-2.15
LOC100862145	6.29	6.25	7.1	5.96	-2.15
AY036118	8.55	7.91	9.65	7.92	-2.14
Kat6a	5.29	5.57	5.55	4.74	-2.14
Plekhb2	4.85	5.28	5.26	4.6	-2.14
Rsl24d1	5.31	5.95	5.7	5.24	-2.14
Tm4sf1	6.24	6.1	6.81	5.57	-2.14
Gm13345	4.19	5.29	4.14	4.14	-2.14
Cuta	4.78	5.19	5.48	4.8	-2.13

Ezh1	7.49	7.39	8.02	6.83	-2.13
Rpl37rt	7.65	8.04	8.17	7.46	-2.13
Rpl31-ps7	9.2	9.13	9.05	7.89	-2.13
Csrnp3	5.14	5.66	5.51	4.95	-2.12
Ncl	6.98	6.59	7.67	6.2	-2.12
Ogfrl1	5.38	5.39	5.94	4.87	-2.12
	10.41	10.7	10.71	9.91	-2.12
	4.03	4.7	4.55	4.14	-2.12
	5.03	5.01	5.44	4.33	-2.12
Gm14176	4.22	4.33	5.27	4.31	-2.12
Gm15981	4.57	4.98	4.94	4.27	-2.12
Gm24328	3.73	4.06	4.44	3.69	-2.12
Gm9386	5.65	6.61	5.93	5.81	-2.12
Rpl31-ps6	7.27	7.8	7.39	6.84	-2.12
Dusp11	6.1	6.56	6.18	5.56	-2.11
Gm3317; Gm3488	5.39	6.03	5.59	5.14	-2.11
Pgm2l1	5.99	6.78	7.11	6.83	-2.1
Vcl	4.84	5.21	5.27	4.57	-2.1
Gm14516	5.06	5.28	5.96	5.11	-2.1
Gm15531	4.88	5.52	5.53	5.1	-2.1
4933409K07Rik	5.52	6.16	6.21	5.78	-2.09
Anxa6	4.77	4.77	5.53	4.47	-2.09
Atp1a1	8.32	8.6	9.23	8.46	-2.09
Prkaa1	4.29	4.74	4.9	4.28	-2.09
Rnf25	4.75	5.3	5.34	4.83	-2.09
Sirt2	6.38	7	7.11	6.66	-2.09
Eprs	5.86	5.94	6.58	5.61	-2.08
Tnpo3	4.9	5.16	5.56	4.77	-2.08
Gm7927	3.57	4.33	4.15	3.85	-2.08
Gm12492	5.85	6.04	6.48	5.61	-2.08
Akap8l	7.22	7.21	7.73	6.67	-2.07
Rpl10; Snora70	4.62	4.97	5.54	4.84	-2.07
	4.34	5.26	4.96	4.83	-2.07
	4.99	5.37	5.95	5.28	-2.07
Gm12447	6.42	6.74	6.63	5.91	-2.07
Gm15378	3.83	4.52	4.18	3.81	-2.07
Gm19650	4.89	5.97	4.88	4.91	-2.07
Cbx3-ps7	6.12	6.36	6.31	5.51	-2.06
Gnai2	7.15	6.94	7.73	6.48	-2.06
Ncor1	7.15	7.44	7.3	6.55	-2.06
Ppp1r16b	6.15	6.83	6.41	6.05	-2.06
Gm16082	4.14	4.7	4.54	4.05	-2.06
Gm24139	18.46	18.5	18.46	17.45	-2.06
Prelid1	5.24	5.09	6.08	4.89	-2.05

Tnks2	5.72	5.94	6.31	5.5	-2.05
Rbfox2	5.56	5.83	6.11	5.36	-2.04
Zfp871	5.26	5.54	5.51	4.77	-2.04
	4.23	4.56	4.79	4.1	-2.04
Aldoc	6.58	7.02	7.42	6.83	-2.03
Gba	5.46	5.72	6.6	5.84	-2.03
Nsf	6.04	6.28	6.77	5.99	-2.03
	5.06	4.56	6.41	4.89	-2.03
	4.11	4.22	4.6	3.69	-2.03
Gm6736	4.18	4.75	4.39	3.93	-2.03
Agrn	5.91	6.03	6.45	5.56	-2.02
Clasp2	5.17	5.29	5.92	5.03	-2.02
Puf60	5.99	5.85	6.75	5.6	-2.02
Slc24a2	6.24	6.49	6.47	5.71	-2.02
LOC100862033	4.84	5.66	5.35	5.17	-2.02
	4.26	4.89	4.81	4.44	-2.01
Gm22194	4.85	4.9	5.37	4.42	-2.01
1700020I14Rik	6.38	6.1	6.97	5.69	-2
LOC100861762	4.38	4.21	5.21	4.04	-2
Olfr350	3.67	3.47	3.32	4.12	2
	4.11	3.63	3.71	4.23	2
	3.84	3.55	3.45	4.16	2
	4.16	3.74	3.98	4.56	2
	4.11	3.62	3.63	4.14	2
Gm10807	4.09	3.39	3.43	3.74	2
Rpl15-ps4	3.96	3.57	3.52	4.13	2
Serpina3d-ps	4.13	3.48	3.51	3.86	2
	5.05	4.61	4.65	5.21	2.01
	3.81	3.65	3.56	4.41	2.01
	3.74	3.66	3.5	4.42	2.01
	3.67	3.6	3.5	4.44	2.01
	4.16	3.68	3.61	4.13	2.01
	4.8	4.34	3.93	4.47	2.01
	4.11	3.55	3.39	3.83	2.01
	4.49	4.52	3.96	4.99	2.01
	4.05	3.74	3.69	4.38	2.01
	5.28	4.19	4.44	4.36	2.01
	4.26	4.07	3.81	4.63	2.01
	6.34	6.05	5.64	6.36	2.01
Gm24899; Gm26945; RP23-204I16.2	5.15	4.52	4.81	5.2	2.01
Mir6353	4.84	4.63	4.57	5.38	2.02
1700063A18Rik	4.23	3.82	3.63	4.23	2.02

	5.87	5.7	5.28	6.13	2.02
	3.95	3.65	3.69	4.4	2.02
	4.66	4.44	4.02	4.82	2.02
	4.23	3.61	3.45	3.84	2.02
Gm15135; AC123830.11	4.38	3.63	3.68	3.95	2.02
Mirlet7g	4.06	3.65	3.31	3.91	2.03
	3.84	3.78	3.46	4.42	2.03
	4.82	4.15	4.39	4.75	2.03
	4.63	3.97	4.11	4.46	2.03
	4.15	3.71	3.77	4.35	2.03
	4.82	4.14	4.05	4.39	2.03
	4.35	4.12	3.5	4.3	2.03
	4.51	4.17	4	4.68	2.03
	4.2	3.75	3.6	4.16	2.03
	3.98	3.72	3.72	4.48	2.03
	5.98	5.34	5.73	6.12	2.03
	4.21	4.06	3.83	4.7	2.03
	4.28	3.77	3.93	4.44	2.03
	3.87	3.49	3.48	4.12	2.03
Gm11417; RP23-397J5.2	5.65	4.97	4.88	5.22	2.03
Gm20543	4.13	3.65	3.53	4.08	2.03
Gm25418	6.23	6	5.68	6.47	2.03
	3.72	3.42	3.71	4.44	2.04
	4.98	4.61	4.59	5.25	2.04
	4.08	3.68	3.32	3.94	2.04
	4.46	4.14	4.15	4.85	2.04
	8.49	8.08	7.89	8.51	2.04
	5.78	4.66	4.99	4.9	2.04
Gm22791	4.63	4.44	4.28	5.11	2.04
Gm23421	4	3.63	3.65	4.31	2.04
Mir1947	5.14	4.34	4.34	4.58	2.05
	5.1	4.4	4.41	4.75	2.05
	4.24	3.83	3.98	4.61	2.05
	4.39	4.29	3.79	4.72	2.05
	4.01	3.77	3.29	4.09	2.05
Gm23125	4.08	3.94	3.95	4.84	2.05
Gm23274	5	4.61	4.49	5.14	2.05
Gm24113	4.06	3.71	3.53	4.23	2.05
Gm26361	4.98	4.33	3.93	4.32	2.05
Mir7081	6.83	6.36	6.01	6.59	2.06
	6.64	5.96	5.86	6.22	2.06
Svs3b	3.87	3.57	3.46	4.21	2.06

	4.15	3.98	3.79	4.65	2.06
	5.11	4.95	4.6	5.48	2.06
	5.1	4.26	4.42	4.63	2.06
	3.71	3.61	3.36	4.3	2.06
	4.97	4.03	4.19	4.3	2.06
Gm11882	4.24	3.73	3.81	4.34	2.06
Gm12909; RP23-339M2.1	4.06	3.85	3.49	4.33	2.06
Gm15168	4.35	3.65	3.53	3.88	2.06
Gm15488	3.76	3.63	3.46	4.37	2.06
Gm24490	4.17	3.4	3.75	4.02	2.06
Gm25838	4.39	4.05	3.76	4.46	2.06
	5.62	5.25	4.83	5.51	2.07
Gm10512	4.21	3.96	4.01	4.81	2.07
	3.99	3.64	3.6	4.3	2.07
	3.81	3.56	3.47	4.26	2.07
Gm11257	3.73	3.56	3.37	4.25	2.07
Gm12638	5.22	4.56	4.67	5.06	2.07
Gm15749; AC125183.7	4	3.75	3.4	4.2	2.07
	3.75	3.35	3.46	4.12	2.08
	3.95	3.74	3.8	4.64	2.08
	4.05	3.73	3.65	4.39	2.08
	6.26	5.51	5.45	5.75	2.08
Gm12584	4.2	3.79	3.43	4.08	2.08
Gm17739	4.33	3.69	3.77	4.18	2.08
Olfr248	4.04	3.45	3.5	3.97	2.09
Olfr1509	3.76	3.44	3.41	4.16	2.09
Olfr1506	4.05	3.75	3.67	4.44	2.09
	4.44	4.05	3.7	4.38	2.09
	4.51	3.7	4.31	4.57	2.09
	4.46	3.67	3.82	4.09	2.09
	4.19	3.84	3.57	4.29	2.09
	4.38	3.87	3.9	4.45	2.09
	6.42	6.02	5.72	6.37	2.09
	5.79	4.92	5.32	5.51	2.09
	4.54	3.9	3.76	4.18	2.09
Gm20471; RP24-485J8.2	4.07	3.42	3.55	3.96	2.09
Gm22257	3.91	3.48	3.45	4.09	2.09
Rhox7b	5.54	5.21	4.81	5.54	2.09
Ighv1-54	4.99	4.69	4.08	4.84	2.1
	3.88	3.51	3.79	4.48	2.1
	4.36	3.56	3.58	3.85	2.1

	3.84	3.76	3.72	4.71	2.1
	4.75	3.48	3.92	3.72	2.1
	4.42	3.79	4.04	4.48	2.1
	3.73	3.61	3.36	4.32	2.1
	3.9	3.77	3.79	4.73	2.1
	4.92	4.54	3.91	4.59	2.1
	4.89	4.51	4.33	5.01	2.1
Gm2930	4.18	3.83	3.83	4.54	2.1
Gm14999	4.31	3.88	3.72	4.35	2.1
Gm22764	4.75	4.29	4.37	4.98	2.1
Gm25776	4.57	4.18	3.67	4.35	2.1
Gm19935	4.68	4.57	4.18	5.14	2.1
LOC100861643	5.6	5.18	5.07	5.73	2.1
1700034J04Rik	3.83	3.37	3.29	3.9	2.1
Mir7036	5.5	4.81	4.76	5.15	2.11
	4.28	3.86	3.98	4.64	2.11
	3.83	3.45	3.43	4.13	2.11
	4.16	3.89	3.85	4.66	2.11
	3.98	3.57	3.48	4.15	2.11
	3.89	3.65	3.54	4.38	2.11
	4.22	4.18	3.83	4.86	2.11
Gm3513; AC113945.3	4.41	3.97	4.15	4.79	2.11
Mir6928	4.45	3.56	3.25	3.45	2.12
Mir7033	4.09	4.06	3.59	4.64	2.12
Olfr125	3.96	3.46	3.59	4.17	2.12
Olfr1373	4.21	3.73	3.77	4.38	2.12
	5.28	4.84	4.77	5.41	2.12
	4.27	4.04	3.47	4.34	2.12
	4.2	3.65	3.62	4.16	2.12
	7.15	6.77	6.57	7.27	2.12
	4.32	4.15	3.73	4.65	2.12
n-R5s205	4.52	4.58	3.81	4.95	2.12
Gm9457	8	7.44	7.52	8.05	2.12
Gm12951; RP23-276M23.2	4.87	4.33	4.55	5.11	2.12
Gm25334	3.91	3.72	3.6	4.5	2.12
Mir155	4.12	3.83	3.64	4.43	2.13
Wdyhv1	4.78	3.86	3.89	4.07	2.13
	6.53	6.2	5.66	6.42	2.13
	4.48	3.84	3.92	4.37	2.13
Gm12577	4.52	3.77	4.5	4.85	2.13
Gm22649	4.91	4.53	4.45	5.16	2.13
Gm25488	4.3	3.57	3.57	3.94	2.13

Ranbp2-ps9	4.86	4.21	4.05	4.49	2.13
Mir3086	4.26	3.57	3.38	3.79	2.14
3930402G23Rik	4.16	3.95	3.75	4.64	2.14
	3.81	3.72	3.37	4.37	2.14
	4.13	4.02	3.63	4.62	2.14
	4.4	4.18	3.96	4.84	2.14
Gm11680; RP23-173A8.2	4.26	3.83	3.86	4.53	2.14
Gm12195; RP23-319B15.6	4.76	4.33	4.21	4.88	2.14
	4.16	3.82	3.68	4.44	2.15
	3.64	3.3	3.34	4.1	2.15
	4.75	4.21	4.25	4.82	2.15
Gm11313	5.65	4.95	5.04	5.44	2.15
Gm25130	5.06	4.44	4.62	5.11	2.15
Ranbp2-ps2	4.67	4.17	3.66	4.26	2.15
	4.67	4.39	4.1	4.94	2.16
	3.69	3.27	3.27	3.96	2.16
Olfr719-ps	4.31	3.65	3.69	4.14	2.16
Prdx6-ps2	4.41	3.76	3.58	4.04	2.16
Gm27530	7.64	7.6	6.97	8.04	2.16
Mir7094-1	4.33	3.86	3.66	4.32	2.17
	5.06	4.16	4.22	4.44	2.17
	4.27	4.02	3.59	4.46	2.17
	4.3	3.96	3.82	4.6	2.17
Gm14330; AC124590.1	3.83	3.75	3.69	4.73	2.17
Gm15166	4.61	4.28	4.1	4.9	2.17
Gm25005	5.26	4.57	4.79	5.22	2.17
Gm26556; RP23-175C13.5	4.9	4.26	4.43	4.91	2.17
1700010B13Rik	4.47	4.29	3.83	4.76	2.17
5430401H09Rik	3.81	3.53	3.21	4.05	2.17
Olfr437	5.19	4.7	4.24	4.88	2.18
Olfr1030	3.85	3.4	3.41	4.08	2.18
Rnase2b	4.17	3.67	3.69	4.32	2.18
	3.92	3.52	3.65	4.38	2.18
	4	3.36	3.43	3.92	2.18
Gm12054; RP23-16B24.1	5.52	5.27	4.56	5.44	2.18
Magea2	5.12	4.44	4.58	5.02	2.19
	4.68	3.96	4.15	4.56	2.19
	3.66	3.4	3.3	4.18	2.19
	4.31	4.11	3.92	4.86	2.19

	4.71	3.62	4.11	4.15	2.19
	4.04	3.74	3.45	4.28	2.19
	4.47	4.17	3.75	4.58	2.19
	4.59	4.05	3.95	4.55	2.19
	4.15	3.7	3.65	4.34	2.2
	4.24	3.87	3.91	4.68	2.2
	6.56	5.42	5.93	5.93	2.2
	5.4	5.14	4.74	5.62	2.2
	4.55	3.92	4.01	4.52	2.2
Gm14398	9.96	9	9.18	9.36	2.2
	10.97	10.4	10.01	10.59	2.21
	10.97	10.4	10.01	10.59	2.21
Olfr145	3.75	3.61	3.62	4.63	2.21
	4.36	3.87	4.14	4.8	2.21
	3.84	3.57	3.41	4.28	2.21
	5.13	4.75	4.34	5.1	2.21
Gm15833	3.95	3.79	3.77	4.76	2.21
	3.91	4.13	2.88	4.24	2.22
1700109G15Rik	5.57	4.97	4.96	5.52	2.22
	7.73	7.47	6.86	7.76	2.22
	4.26	3.8	4.19	4.87	2.22
	4.08	3.96	3.47	4.5	2.22
	11.03	10.9	10.35	11.37	2.22
Gm15211	4.84	4.29	4.18	4.79	2.22
Gm23456	5.5	4.83	4.83	5.32	2.22
Gm24392	4.05	3.73	3.47	4.31	2.22
Gm24921	3.52	3.71	3.46	4.79	2.22
	4.26	3.48	3.57	3.95	2.23
	4.49	3.8	3.87	4.34	2.23
	3.79	3.36	3.44	4.17	2.23
	4.52	4.31	4	4.95	2.23
	4.41	3.79	3.85	4.38	2.23
Gm22578	4.11	3.57	3.93	4.56	2.23
Gm26187	4.15	3.9	3.89	4.8	2.23
	3.89	3.66	3.88	4.81	2.24
Spata31d1b	5.28	5.16	4.71	5.76	2.24
	4.29	3.63	3.74	4.23	2.24
	8.21	7.53	7.85	8.33	2.24
	5.24	4.75	4.64	5.31	2.24
	4.17	3.77	3.53	4.3	2.24
	5.01	4.24	4.26	4.65	2.24
Gm12740	4.47	3.99	3.95	4.62	2.24
Gm22713	4.87	4.65	4.45	5.4	2.24
	5.11	4.21	4.14	4.4	2.25

	4.25	3.52	3.55	3.99	2.25
Mir6917	6	6.04	5.14	6.35	2.26
	4.04	3.49	3.53	4.16	2.26
	4.79	3.92	3.82	4.12	2.26
	3.78	3.23	3.47	4.1	2.26
	7.06	6.95	7.12	8.19	2.26
	3.78	3.53	3.43	4.35	2.26
n-R5s52	4.2	3.46	3.81	4.25	2.26
Gm6254	11.17	10.08	10.18	10.27	2.26
Gm24124	5.07	4.51	4.48	5.09	2.26
Gm25819	4.8	4.11	4.41	4.89	2.26
Olfr734	4.36	4.1	3.43	4.35	2.27
	8.55	7.85	7.99	8.48	2.27
	4.44	3.6	3.67	4.02	2.27
	4.61	4.29	4.07	4.93	2.27
	4.38	3.78	3.76	4.34	2.27
	5.24	4.93	4.6	5.47	2.27
	4.09	3.84	3.71	4.64	2.27
	5.93	5.73	5.71	6.69	2.27
Gm26004	4.31	3.95	3.41	4.23	2.27
Gm10862	4.94	4.23	5.05	5.53	2.27
	4.86	3.96	4.17	4.45	2.28
	5	4.63	4.38	5.19	2.28
	10.93	11.03	9.74	11.03	2.28
	4.41	3.89	3.6	4.28	2.28
Mir7665	5.39	4.34	4.43	4.57	2.29
	5.24	4.86	4.62	5.44	2.29
	3.8	3.51	3.65	4.55	2.29
Gm26581; RP23-58L22.8	3.64	3.57	3.37	4.5	2.29
	5.76	5.35	5.07	5.86	2.3
	5.01	4.65	4.53	5.36	2.3
	4.16	3.64	3.58	4.25	2.3
	4.24	3.73	3.64	4.32	2.3
	4.53	3.84	4.09	4.6	2.3
	7.03	6.56	6.23	6.97	2.3
Gm11617	4.01	3.71	3.38	4.28	2.3
Gm24348	4.32	4.11	3.69	4.68	2.3
Gm26396	3.98	3.56	3.6	4.39	2.3
1700055C04Rik	3.55	3.35	3.4	4.4	2.31
	4.07	3.58	3.55	4.26	2.31
Gm12055; RP23-16B24.2	4.83	4.59	4.59	5.57	2.31
Gm24628	4.3	3.7	3.42	4.02	2.31

Mir6964	5.94	4.91	5.04	5.22	2.32
	6.19	5.52	5.14	5.68	2.32
Olfr102; Olfr100	4.52	3.85	3.84	4.38	2.32
Gm14345; Gm14346; Gm14351	4.06	3.46	3.77	4.39	2.32
	3.75	3.67	3.64	4.77	2.33
Gm19773	6.66	5.9	6.04	6.49	2.33
	6.68	5.78	6.09	6.42	2.34
	3.91	3.7	3.41	4.43	2.34
Gm15371	4.11	3.65	3.53	4.3	2.34
Gm27016; RP23-433F5.2	4.82	4.29	4.45	5.15	2.34
	4.14	3.83	3.72	4.64	2.35
	6.2	5.17	5.47	5.67	2.35
	4.57	4.08	4	4.74	2.35
	5.12	4.66	4.62	5.39	2.35
Gm15208; RP24-266P11.2	4.53	4.08	3.89	4.68	2.35
Gm22868	5.03	4.92	4.18	5.31	2.35
Ighv8-5	4.22	3.69	3.69	4.41	2.36
	4.2	3.98	3.57	4.58	2.36
	5.81	5.21	5.24	5.88	2.36
	3.9	3.49	3.36	4.19	2.36
	4.26	3.54	3.59	4.1	2.36
Mir5099	8.65	7.14	7.58	7.31	2.37
Mir5099	8.65	7.14	7.58	7.31	2.37
	4.47	4.08	3.87	4.73	2.37
	4.83	4.17	4.15	4.72	2.37
	4.18	3.63	3.55	4.25	2.37
	4.33	3.76	3.65	4.32	2.37
	4.78	4.07	3.66	4.2	2.37
	5.27	4.87	4.7	5.56	2.38
	4.27	3.7	3.68	4.36	2.38
	4.42	3.95	3.8	4.58	2.39
	4.16	3.68	3.47	4.25	2.39
	4.65	3.69	3.66	3.96	2.39
Gm10985	5.14	4.65	4.52	5.29	2.39
Gm25699	4.29	3.51	3.67	4.16	2.39
Gm27313	4.59	3.9	3.59	4.15	2.39
	5.37	5.07	4.88	5.85	2.4
	4.85	4.35	4.05	4.81	2.4
	4.71	4.39	4.05	5	2.4
	4.41	4.38	3.95	5.18	2.4

	4.25	3.87	3.72	4.61	2.41
	4.34	3.81	3.81	4.55	2.41
	6.68	6.73	5.78	7.11	2.41
	4.54	3.92	3.84	4.48	2.41
	5.33	4.1	4.4	4.44	2.41
	4.58	3.51	3.69	3.88	2.41
	4.76	3.82	4.27	4.6	2.41
Trav6-3	4.63	3.84	3.97	4.45	2.41
Mirlet7f-1	4.9	4.51	4.17	5.05	2.42
	4.09	3.76	3.83	4.78	2.42
	13.5	12.9	12.61	13.28	2.42
	4.87	4.03	3.99	4.42	2.42
Mir466f-1	6.36	5.44	6.25	6.61	2.43
	4.28	3.86	3.39	4.24	2.43
	5.12	4.21	4.6	4.97	2.43
	4.37	3.73	3.83	4.47	2.43
	6.75	5.8	5.11	5.44	2.43
Gm6161	9.83	8.92	8.86	9.23	2.43
Gm24008	4.97	4.76	4.42	5.5	2.43
Samd15	4.15	3.77	3.67	4.59	2.44
	3.98	3.74	3.35	4.4	2.44
Gm12060	5.12	4.01	4.57	4.75	2.45
	4.36	3.83	3.9	4.67	2.45
Dph3b-ps	4.29	4.03	3.79	4.83	2.46
Mir6987	4.54	3.85	3.85	4.46	2.46
	5.55	3.7	4.79	4.25	2.46
Gm24238	3.77	3.45	3.23	4.2	2.46
	4.99	4.42	4.29	5.02	2.47
	5.17	3.87	3.79	3.79	2.47
Mir3098	6.84	6.51	6.19	7.17	2.48
	3.86	3.58	3.44	4.47	2.48
	4.65	4.16	4	4.82	2.49
	3.8	3.67	3.28	4.47	2.49
Gm25407	4.72	4.5	3.83	4.93	2.49
Gm26414	4.19	4	3.51	4.63	2.49
Gm26847	5.35	5.02	5.09	6.07	2.49
Gm11116	4.44	3.53	3.86	4.27	2.5
Gm25938	4.9	4.24	4.03	4.69	2.5
	3.77	3.17	3.19	3.91	2.51
Olfr692	5.65	5.39	4.8	5.86	2.51
Olfr912	5.14	4.36	4.39	4.94	2.51
	4.75	4.12	4.61	5.31	2.51
	5.43	4.84	4.64	5.38	2.51
	4.03	3.56	3.41	4.27	2.51

Gm14481	4.47	4.17	4.25	5.28	2.51
	3.76	3.41	3.52	4.5	2.52
	4.5	3.92	3.99	4.74	2.52
	3.8	3.49	3.28	4.3	2.52
Gm14951; RP23-83P7.2	4.2	3.58	3.61	4.32	2.52
	3.71	3.69	3.47	4.79	2.53
	4.79	4.43	4.4	5.38	2.53
Gm24111	4.09	3.76	3.65	4.66	2.53
	4.18	3.83	3.56	4.55	2.54
	5.63	5.29	4.92	5.94	2.55
Gm13686; RP23-141N19.1	5.39	4.82	5.01	5.78	2.55
	7.8	6.49	6.29	6.34	2.56
	4.66	3.97	4.06	4.73	2.56
Gm14946	8	7.86	6.87	8.09	2.56
Olfr978	4.28	3.84	3.54	4.46	2.57
	4.39	3.92	3.92	4.8	2.57
Gm16171	4.31	3.79	3.89	4.73	2.57
	17.57	17.38	16.49	17.67	2.58
	5.46	4.93	4.91	5.74	2.58
	3.81	3.38	3.22	4.16	2.58
	4.51	4.11	3.94	4.9	2.58
Gm24291	5.51	4.83	5.15	5.84	2.58
Olfr959	4.19	3.61	3.55	4.35	2.59
	4.64	4.09	3.76	4.59	2.59
Gm23407	9.23	8.95	8.56	9.65	2.59
Mir5125	7.08	5.87	5.38	5.54	2.6
	8.39	7.67	7.67	8.33	2.6
Gm6117; RP23-192D5.2	4.61	3.84	3.59	4.21	2.6
Gm26741	3.93	3.45	3.49	4.39	2.6
	5.26	4.6	4.72	5.44	2.61
Gm22105	4.13	3.43	3.41	4.1	2.62
Gm27190	4.03	3.49	3.36	4.21	2.63
	4.86	4.33	3.74	4.62	2.64
	5.02	4.32	4.26	4.96	2.64
	5.34	4.93	4.65	5.64	2.64
	3.77	3.7	3.66	4.98	2.64
Mir5128	4.55	3.12	3.63	3.61	2.65
Mir1951	8.31	8.04	7.61	8.76	2.66
n-R5s202	4.92	3.95	4.25	4.7	2.66
Gm14731	4.51	3.89	3.93	4.73	2.66
	4.3	3.64	3.39	4.14	2.67

	4.44	3.86	4.04	4.88	2.67
	4.7	4.75	3.96	5.44	2.67
	4.41	3.7	3.88	4.6	2.68
	4.73	4.26	4.08	5.03	2.68
	4.06	3.66	3.67	4.69	2.68
Gm17919	3.82	3.59	3.44	4.63	2.68
Gm26826	4.16	3.93	3.44	4.63	2.68
Ranbp2-ps8	5.57	4.62	4.41	4.88	2.68
	4.12	3.59	3.47	4.37	2.69
Mir365-1	5.96	4.81	4.87	5.16	2.7
Mir3092	7.87	8.02	6.87	8.46	2.7
Gm11037	5.05	4	3.64	4.03	2.72
Olfr1113	4.07	3.19	3.58	4.15	2.73
	5	4.28	4.25	4.98	2.73
	7.26	5.93	6.53	6.65	2.73
Mir7078	6.58	5.76	5.79	6.43	2.74
n-R5s216	4.58	4.01	3.82	4.71	2.74
Gm24621	6.34	4.88	5.13	5.15	2.76
PRINS	4.14	3.83	3.45	4.61	2.76
Gm24079	4.8	4.47	4.12	5.26	2.77
Mir7667	4.08	3.8	3.99	5.19	2.79
	4.51	3.82	3.77	4.57	2.8
	5.27	4.86	5.02	6.09	2.8
Gm23753	5.49	4.89	4.55	5.44	2.8
Ifna11	4.13	3.97	3.76	5.09	2.81
Mir6338	4.43	4.24	4.04	5.35	2.81
	4.62	4.1	3.89	4.86	2.81
	13.59	13.57	12.36	13.83	2.81
Gm23000	6.75	6.15	6.15	7.04	2.81
Mir1961	4.6	3.85	3.73	4.47	2.82
Gm24691	4.42	3.96	3.57	4.6	2.82
Gm24913	4.2	4.09	3.9	5.28	2.82
Mir5108	7.45	6.73	6.74	7.52	2.83
	5.62	4.29	4.27	4.45	2.84
	4.69	4.34	4.4	5.56	2.84
	6.23	5.31	5.15	5.74	2.85
Gm26411	5.17	4.68	4.79	5.81	2.85
	4.68	3.85	3.68	4.37	2.86
	4.61	4.77	3.7	5.38	2.87
	7.85	6.95	6.78	7.4	2.87
	4.21	3.6	3.55	4.46	2.88
Gm22927	4.99	4.22	4.58	5.34	2.88
n-R5s151	5.81	5.19	4.78	5.7	2.89
Gm20917	4.6	3.79	3.77	4.5	2.89

	5.94	4.44	4.65	4.68	2.9
	4.61	3.74	3.73	4.4	2.9
Gm13018; RP23-416J8.1	5.88	4.61	5.38	5.66	2.93
	4.43	3.78	3.63	4.54	2.95
	5.77	5.3	5.14	6.24	2.97
Gm24783	5.27	4.34	3.95	4.59	2.98
Gm22779	4.83	4.18	4.28	5.21	2.99
Gm23448	5.02	4.05	3.86	4.47	2.99
	5.1	3.97	3.71	4.18	3.01
Gm16339; RP24-369B4.1	5.27	4.37	3.95	4.65	3.03
Gm23308	6.87	6.57	6.42	7.72	3.03
	4.64	3.42	3.37	3.77	3.07
	4.66	4.25	4.14	5.36	3.07
	4.67	3.63	3.78	4.37	3.1
Gm24233	5.1	4.68	4.37	5.58	3.1
	4.75	3.75	3.92	4.57	3.12
	7.28	6.36	6.14	6.86	3.13
	5.44	4.79	4.14	5.14	3.14
	4.41	3.8	4.23	5.27	3.14
Gm25153	5.43	4.74	4.27	5.23	3.14
Gm25722	5.5	4.97	4.75	5.88	3.14
	4.84	4.69	4.05	5.57	3.17
Gm26308	7.96	7.57	7.44	8.72	3.17
	7.65	7.06	6.29	7.36	3.18
Mir7094-2	4.57	3.77	3.64	4.52	3.21
Ighv5-12-4	7.48	7.22	6.16	7.59	3.23
Mir1960	5.04	4.46	3.65	4.77	3.24
Gm23737	6.18	5.59	5.09	6.21	3.25
Mir6916	6.12	5.45	5.05	6.09	3.28
	4.54	4.07	3.52	4.77	3.28
	4.25	3.24	3.44	4.15	3.28
Gm27490	5.87	5.26	5.09	6.21	3.3
	5.82	4.61	4.51	5.04	3.32
	6.86	5.65	5.72	6.25	3.33
Gm22663	10.95	10.23	9.61	10.63	3.33
	4.13	3.94	3.4	4.95	3.34
Gm23387	5.59	4.79	4.69	5.64	3.35
	7.63	6.92	6.57	7.62	3.4
	6.12	6.32	5.04	7.01	3.42
	5.5	3.66	3.89	3.83	3.44
	12.29	11.99	10.9	12.37	3.44
	4.58	4.16	3.7	5.06	3.45

Gm20391	5.17	4.68	4.29	5.59	3.47
Gm22786	4.88	4.6	4.24	5.76	3.52
Gm15724; RP23-246B24.3	7.92	6.94	6.46	7.31	3.55
	5	4.32	4.46	5.63	3.57
	4.98	4.18	4.1	5.14	3.6
Gm25160	5.98	4.86	5.27	6.01	3.64
Gm2531	6.7	4.98	4.78	4.95	3.7
Gm14209; RP23-464H11.2	5.36	4.39	4.2	5.14	3.74
Gm17268	13.88	13.29	13.48	14.8	3.75
Gm25428	4.95	4.26	4.53	5.76	3.78
Gm22507	7.21	5.74	5.97	6.43	3.81
Mir6388	5.01	4.21	4.25	5.38	3.82
Gm24541	5.82	5.25	4.48	5.85	3.85
	6.64	5.07	5.18	5.55	3.86
Gm23322	7.68	7.13	6.43	7.84	3.86
Mir511	5.21	4.19	4.28	5.21	3.87
Gm14604; RP23-260P9.1	6.74	5.98	5.32	6.53	3.92
Gm22527	4.65	4.35	3.76	5.46	3.99
Traj29	7.98	7.13	6.76	7.91	4.01
Gm25522	7.11	5.98	5.96	6.83	4.02
Mir7053	10.78	9.81	9.57	10.62	4.03
	5.69	4.81	4.71	5.85	4.04
LOC100861648	5.89	4.66	5.07	5.87	4.08
	4.97	3.58	3.78	4.43	4.1
	6.83	5.79	5.18	6.19	4.16
Gm25581	7.61	6.14	6.24	6.82	4.17
	5.6	4.65	4.83	5.94	4.19
	6.65	5.97	5.45	6.87	4.29
Gm26002	6.26	5.24	5.53	6.62	4.29
Gm23221	7.19	6.17	6.18	7.27	4.31
Gm22043	5.64	4.48	4.4	5.38	4.38
	5.06	4	4.05	5.13	4.42
	5.06	4	4.05	5.13	4.42
	5.06	4	4.05	5.13	4.42
	8.11	7.05	7.19	8.28	4.45
	5.08	3.72	3.79	4.58	4.46
Gm25660	9.33	8.37	7.94	9.15	4.51
	6.2	5.35	5.29	6.63	4.52
	6.2	5.35	5.29	6.63	4.52
	6.2	5.35	5.29	6.63	4.52
	6.2	5.35	5.29	6.63	4.52

	6.2	5.35	5.29	6.63	4.52
	6.2	5.35	5.29	6.63	4.52
	6.2	5.35	5.29	6.63	4.52
	6.2	5.35	5.29	6.63	4.52
	6.2	5.35	5.29	6.63	4.52
	6.2	5.35	5.29	6.63	4.52
	6.2	5.35	5.29	6.63	4.52
	6.2	5.35	5.29	6.63	4.52
	6.2	5.35	5.29	6.63	4.52
	6.2	5.35	5.29	6.63	4.52
	6.2	5.35	5.29	6.63	4.52
	6.2	5.35	5.29	6.63	4.52
	6.2	5.35	5.29	6.63	4.52
	6.2	5.35	5.29	6.63	4.52
Mir7047	7.55	6.08	6.36	7.08	4.53
Gm24088	6.36	4.81	5.7	6.35	4.6
	4.85	3.69	3.56	4.61	4.63
	4.79	3.63	4.13	5.18	4.64
	4.92	4.26	3.87	5.44	4.72
	11.31	10.25	10.17	11.38	4.85
Gm24032	7.15	6.14	6.01	7.3	4.91
Mir6930	12.03	11.09	11.07	12.54	5.29
	5.82	4.12	4.08	4.82	5.39
	12.31	10.69	10.6	11.44	5.51
Gm26121	6.24	4.76	4.74	5.74	5.57
Gm27722	5.74	5.43	4.64	6.86	5.77
	5.41	4.91	4.7	6.79	6.04
	10.36	9.27	8.55	10.05	6.04
Gm24878	6.19	4.58	4.69	5.73	6.26
Gm15665	6.44	5.18	3.95	5.37	6.44
Ighv1-63	6.25	6.39	4.71	7.7	7.26
Gm17428	19.38	19.16	16.92	19.59	7.37
	8.47	6.51	5.36	6.29	7.45
Mir8095	7.09	5.51	5.22	6.56	7.61
Gm10198	10.18	9.83	8.27	10.88	7.74
	11.47	10.63	9.25	11.53	8.66
Mir8115	9.96	9.05	7.51	9.72	8.68
	12.94	12.94	10.37	13.52	8.94
Snord82	7.59	6.79	5.13	7.61	9.71
Gm10181	15.19	14.72	12.73	15.58	9.97
	9.55	8.64	6.59	9.04	10.38
Gm11172	12.52	11.52	10.66	13.08	10.7
	6.76	5.18	5.36	7.44	12.66
Gm17604	14.73	14.65	10.81	15.11	20.84

Gm23098	7.36	3.84	3.3	4.17	20.99
	3.3	3.25	2.69	7.06	21.26
	3.3	3.25	2.69	7.06	21.26
	3.3	3.25	2.69	7.06	21.26
	3.3	3.25	2.69	7.06	21.26
	3.3	3.25	2.69	7.06	21.26
	3.3	3.25	2.69	7.06	21.26
	16.07	15.69	12.44	16.82	27.01
	16.07	15.69	12.44	16.82	27.01
	16.07	15.69	12.44	16.82	27.01
	16.07	15.69	12.44	16.82	27.01
	16.07	15.69	12.44	16.82	27.01
	7.33	6.23	5.75	9.72	33.66
	8.38	5.93	4.72	9.47	148.27
